# [4+2]-Cycloaddition to 5-Methylidene-Hydantoins and 5-Methylidene-2-Thiohydantoins in the Synthesis of Spiro-2-Chalcogenimidazolones

**DOI:** 10.3390/ijms24055037

**Published:** 2023-03-06

**Authors:** Dmitry E. Shybanov, Maxim E. Kukushkin, Yanislav S. Hrytseniuk, Yuri K. Grishin, Vitaly A. Roznyatovsky, Viktor A. Tafeenko, Dmitry A. Skvortsov, Nikolai V. Zyk, Elena K. Beloglazkina

**Affiliations:** Department of Chemistry, M. V. Lomonosov Moscow State University, 119991 Moscow, Russia

**Keywords:** spiro-compounds, hydantoins, 2-thiohydantoins, Diels–Alder reaction, [4+2]-cycloaddition, cytotoxicity, antibacterial activity

## Abstract

Novel hydantion and thiohydantoin-based spiro-compounds were prepared via theDiels–Alder reactions between 5-methylidene-hydantoins or 5-methylidene-2-thiohydantoins and 1,3-dienes (cyclopentadiene, cyclohexadiene, 2,3-dimethylbutadiene, isoprene). It was shown that the cycloaddition reactions proceed regioselectively and stereoselectively with the formation of exo-isomers in the reactions with cyclic dienes andthe less sterically hindered products in the reactions with isoprene. Reactions of methylideneimidazolones with cyclopentadiene proceed viaco-heating the reactants; reactions with cyclohexadiene, 2,3-dimethylbutadiene, and isoprene require catalysis by Lewis acids. It was demonstrated that ZnI_2_ is an effective catalyst in the Diels–Alder reactions of methylidenethiohydantoins with non-activated dienes. The possibility of alkylation and acylation of the obtained spiro-hydantoinsat the N(1)nitrogen atoms with PhCH_2_Cl or Boc_2_O and the alkylation of the spiro-thiohydantoinsat the S atoms with MeI or PhCH_2_Cl in high yields have been demonstrated. The preparativetransformation of spiro-thiohydantoins into corresponding spiro-hydantoinsin mild conditions by treating with 35% aqueous H_2_O_2_ or nitrile oxide has been carried out. The obtained compounds show moderate cytotoxicity in the MTT test on MCF7, A549, HEK293T, and VA13 cell lines. Some of the tested compounds demonstrated some antibacterial effect against *Escherichia coli* (*E. coli*) BW25113 DTC-pDualrep2 but were almost inactive against *E. coli* BW25113 LPTD-pDualrep2.

## 1. Introduction

Introducing conformationally rigid lipophilic moieties into a molecule is a common approach in the medicinal chemistry [1,2,3,4] that may lead to significant increases in biological activity [5,6,7,8]. One of the convenient methods for the synthesis of such conformationally rigid polycyclic derivatives is the Diels–Alder reaction of functionalized alkenes containing exocyclic C=C bonds with dienes. Dienesvariation makes it possible to fine-tune the structure of the resulting lipophilic framework [9,10,11], and the presence of a C=C double bond in the [4+2]-cycloaddition adduct opens the way for further modifications of the resulting molecules.

We have recently shown that spiro-2-chalcogenimidazolones, obtained by 1,3-dipolarcycloaddition reactions to 5-methylidene-substituted hydantoins, as well as their 2-thio- and 2-seleno-analogs, possess high cytotoxicity probably due to their ability to inhibit the p53-MDM2 proteins interaction [12,13,14]. However, there were no general methods for the synthesis of 5-methylidene-imidazol-4-onederivatives containing spiro-linked 5- and 6-membered rings described previously, and these compounds have not been previously studied for biological activity.

**Scheme 1:**
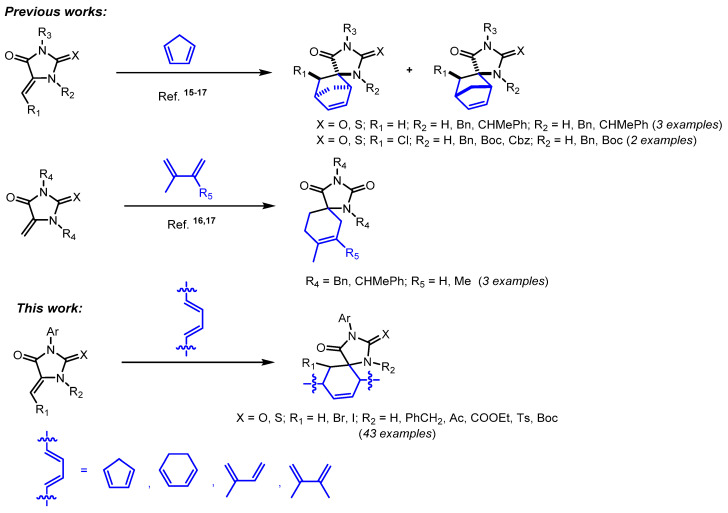
Diels–Alder reactions with methylenehydantoins and methylenethiohydantoins [15,16,17].

Diels–Alder reactions of 5-methylene-substituted imidazolones are described in the literature mainly asthe example of highly reactive cyclopentadiene [15,16,17] and as single examples of the reactions with isoprene and 2,3-dimethylbutadiene [16,17] (Scheme 1). In the present article, a systematic study of [4+2]-cycloaddition reactions of cyclopentadiene, cyclohexadiene, 2,3-dimethylbutadiene, and isoprene with 5-methylene-2-chalcogenimidazolone derivatives, both N-unsubstituted and containing substituents of various natures at nitrogen atoms, is carried out. In this work, we first demonstrated the possibility of methylidenethiohydantoins interaction with low active conjugated dienes, which makes it possible to obtain spirocyclic imidazolones containing various lipophilic frameworks. In addition, the possibility of post-synthetic transformations of the formed [4+2]-cycloaddition products using alkylating and acylating agents was demonstrated; these reactions can be an alternative method for the synthesis of those spiro-imidazolones that are formed in the reactions with dienes in low yields or as the inseparable diastereomers mixtures.

Various types of biological activities have been previously described for hydantoins and their spiro derivatives [18,19].In this case, if, for example, anticonvulsants require low toxicity, then inhibition of the growth of bacteria and tumor cells is determined by the cytotoxic effect. Therefore, for the initial assessment of the biological effect on cells of various types, a number of compounds synthesized in this work were tested on cytotoxicity and antibacterial activity on several human cell lines of various etiologies and several strains of *E. coli*.

## 2. Results and Discussion

### 2.1. Synthesis of Dienophiles

**Scheme 2:**
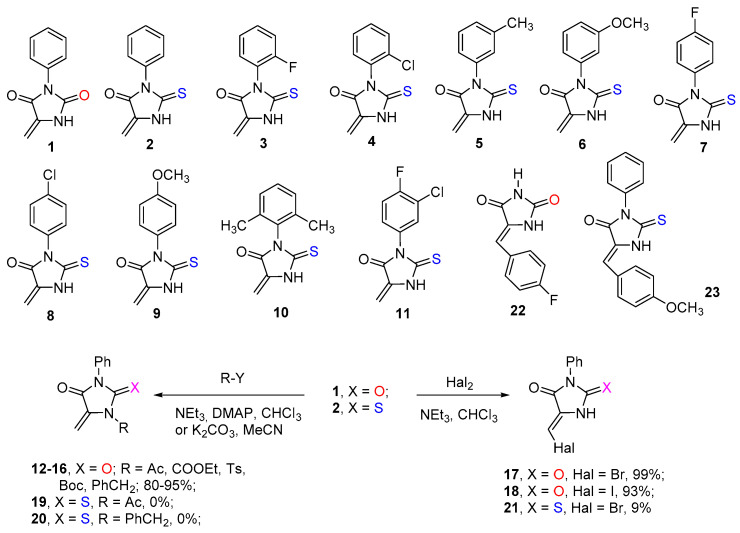
Starting dienophiles and synthesis of compounds **12**-**21** from methylideneimidazolones **1**,**2**.

**Scheme 3:**
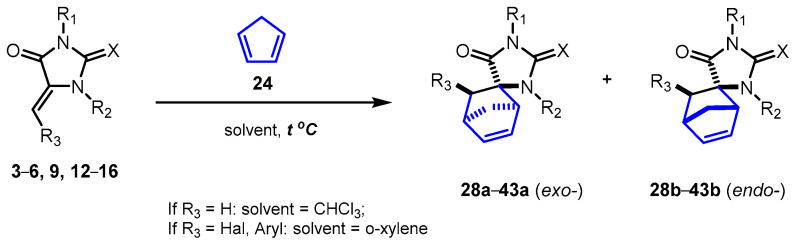
Reactions of methyleneimidazolones with cyclopentadiene.

Initial dienophiles **1**-**11**, **22,** and **23** were prepared according to previously described procedures [20,21,22,23]. Compounds **12**-**21**, containing both N(1)-substituted and N(1)-unsubstituted 2-chalcogenimidazolone moieties with exocyclic C=CH_2_, C=CHHal, and C=CHAr fragments in five positions, were synthesized according to Scheme 2. The substituents with different electronic and steric effects at the C=C double bond should significantly affect the reactivity of the dienophile, which can allow controlling the rate and selectivity of the Diels–Alder reaction with these substrates. It was found that methylidenehydantoin **1** gives high yields of alkylation and acylation products **12**-**16**, and its reaction with halogens (Br_2_, I_2_) in the presence of triethylamine leads to the corresponding halomethylidene-substituted imidazolones **17** and **18** as single isomers (Scheme 2). The configuration of the C=C double bond in compounds **17** and **18** was confirmed by ^1^H NMR spectra of the products of their reactions with cyclopentadiene (Scheme 3 and Supplementary Information).

Our attempts to introduce methylidenetiohydantoin **2** in the reactions with acetyl chloride and benzyl bromide under the same conditions were unsuccessful. We found that thiohydantoin **2** rapidly degrades in the presence of bases (NEt_3_, K_2_CO_3_), and its alkylation or acylation reactions are not formed even in trace amounts (according to ^1^H NMR spectra of reaction mixtures). When methylidenethiohydantoin **2** reacted with bromine at 0°C, resinification of the reaction mixture occurred, and the target halogen-substituted methylenethiohydantoin **21** could be isolated from the reaction mixture only in 9% yield (see Scheme 2 and Supplementary Information).

Next, we started studying the Diels–Alder reactions with dienophiles **1**-**23**. It should be noted that for each specific diene (cyclopentadiene **24**, cyclohexadiene **25**, 2,3-dimethylbutadiene **26**, isoprene **27**), the optimal reaction conditions turned out to be different and were selected depending on its reactivity.

### 2.2. Reactions of 5-Methylideneimidazolones 1-6, 9, 12-18, 22, and 23 with Cyclopentadiene

In our previous communication, we demonstrated the possibility of spiro-norbornene derivatives synthesis by the [4+2]-cycloaddition of highly active dienecyclopentadiene with methylideneimidazolones **1** and **2** at an eight-fold excess of the diene in refluxing chloroform [24]. Hydantoins **3**-**6**, **9,** and **12**-**16** were now introduced into the reactions with cyclopentadiene under the same conditions (Scheme 3, Table 1). In all cases, cycloaddition reactions proceeded in high yields with the predominant formation of the exo products **28a**-**43a**.

It should be noted that, unlike exo- and endo-products **29**-**33a** and **29**-**33b**, which do not contain unsubstituted nitrogen atoms, N(1)-unsubstituted compounds **28a**, **34a**-**39a,** and **28b**, **34b**-**39b** can be separated by column chromatography. Probably, the difference in the chromatographic mobility of the isomers is determined by the ability of the CONH and CSNH fragments of the molecules to form hydrogen bonds with the silica gel surface.

**Figure 1:**
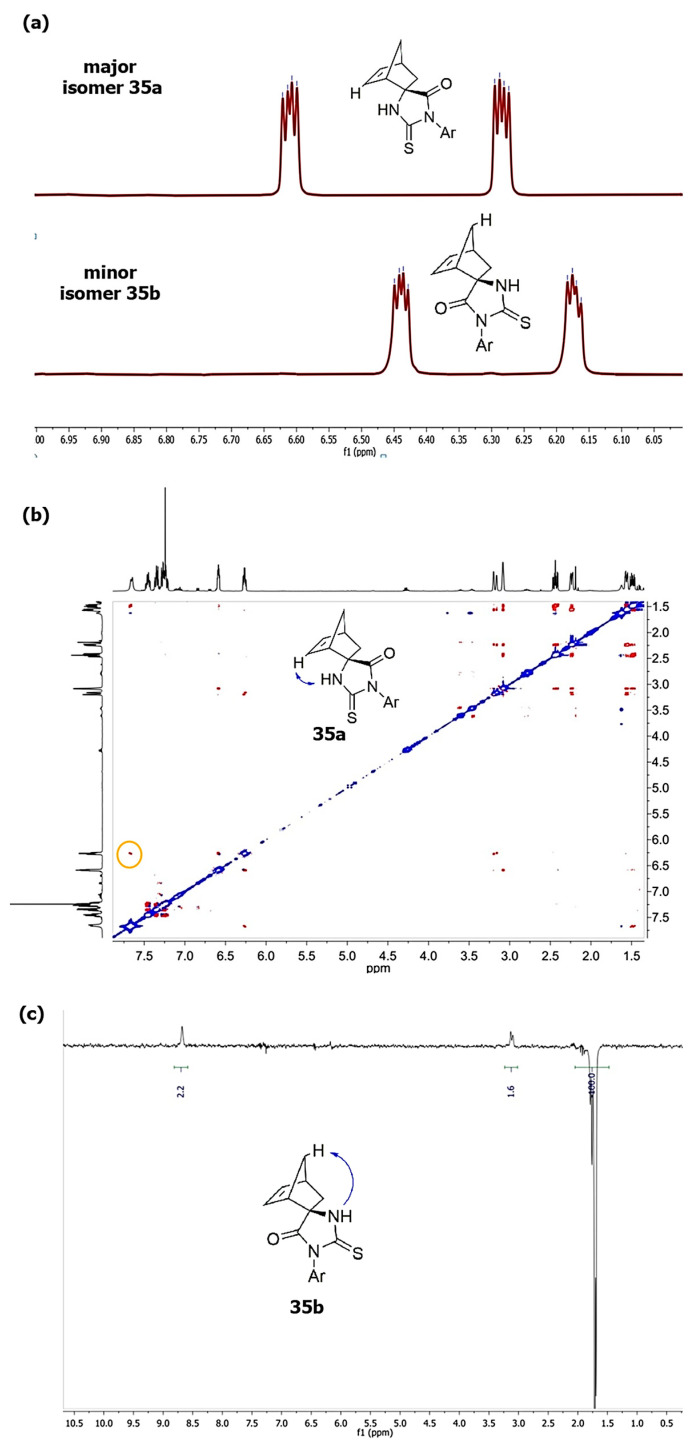
(**a**) Characteristic doublets of doublet set of HC=CH protons of compounds **35a** and **35b** in their ^1^H NMR spectra (CDCl_3_). (**b**) Characteristic correlations observed in ^1^H-^1^H NOESY NMR spectrum of the compounds **35a**. (**c**) ^1^H NOESY NMR spectrum of compound **35b**.

The structures of the resulting spiro-imidazolones **28**-**43** were determined using ^1^H and ^13^C NMR spectroscopy; for compounds **28** and **34,** their spectra correspond to those described in the literature [24]. According to ^1^H NMR spectroscopy data, exo- and endo-products **a** and **b** differ in chemical shifts of HC=CH protons of the norbornene framework: the characteristic doublets of the main products are shifted to a weaker field (at 6.20–6.60 ppm) relative to the signals of minor products (at 6.15–6.45 ppm) (Figure 1a), which is consistent with the literature data [15,17,24]. In addition, compounds **28**, **34**-**39** are characterized by a very large difference in the NH protons shifts of exo- and endo-isomers (>1 ppm in CDCl_3_), which can be explained by the influence of the anisotropy of the double C=C bond, which is quite close to the amide proton in isomer **a**.

The criterion for assigning exo- and endo-stereoisomers of compounds **28**, **34**-**39,** in addition to the chemical shifts of vinyl and NH protons, could be the NOESY spectra shown in the Supplementary Information for compounds **35a** and **35b**. In the NOESY spectrum of spiro-derivative **35a**, the interaction of the NH proton with the double bond proton, as well as with the proton of the nearest methylene group of the bicycle, is manifested, and there is no interaction with the proton at the C(7) atom of the norbornene framework (see Figure 1b). On the contrary, for the minor isomer **35b**, when the NH proton was irradiated, the Overhauser effect is observed for the proton at the C(7) atom of norbornene and is absent for the proton at the C=C double bond.

The diastereoselectivity of the cyclopentadiene Diels–Alder reaction with methylenehydantoins was found to be sensitive to the nature of the substituent at position 1 of the imidazolone moiety. Despite the increase in steric hindrance near the exocyclic double bond of the dienophile, the interaction of cyclopentadiene with hydantoins **12**-**15** containing electron-withdrawing Ac, COOEt, Ts, Boc substituents proceeded less selectively compared to N(1) unsubstituted hydantoin **1**. Such results may indicate that the electronic factors of the substituents have a stronger effect on the reactivity of the dienophile than the steric ones. The important role of the substituent in position 1 of the initial heterocycle on the reaction course is also confirmed by the fact that for the imidazolone **16** with a donor benzyl fragment, the yields of the cycloaddition product **33** are low, and only the isomers **33a** are formed (see Table 1).

Variation of the exocyclic chalcogen atom of the starting heterocycle does not affect the stereoselectivity of the Diels–Alder reaction (Table 1, compare products **28** and **34**). The same stereoselectivity of the Diels–Alder reaction is observed for thiohydantoins **2**-**6** and **9** with different substituents at N(3) atom. Apparently, the electronic properties of substituents in the C(2) and N(3) positions of the heterocycle have little effect on the reactivity of the dienophile.

An increase in steric hindrance at the C=C bond of methylideneimidazolone should hinder the [4+2]-cycloaddition reaction. Indeed, halomethylidenehydantoins **17**, **18** react with cyclopentadiene only when refluxed in ortho-xylene. In the course of these reactions, only exo-isomers **40a** and **41a** were formed in moderate yields. In the ^1^H NMR spectra of compounds **40a** and **41a**, in the region of about 4.7 ppm, there are doublets with *J* ~3.3 Hz, which confirms the spatial arrangement of the CHBr and CHI groups in the norbornene framework.

Imidazolones **22**, **23,** containing the bulkier structure and less acceptor compared to halogens aryl substituents at the double C=C bond, did not form the reaction products with cyclopentadiene under similar conditions even in trace amounts (according to ^1^H NMR spectroscopy of the reaction mixtures), probably for both electronic and steric reasons.

### 2.3. Reactions of 5-Methylideneimidazolones 1-3, 5, 6, 8-10 with Cyclohexadiene

Reactions of 5-methylideneimidazolones with dienes **25**-**27**, which are less reactive than cyclopentadiene, do not occur even when refluxing in toluene with a 20-fold excess of dienes. Under microwave irradiation, when methylidenehydantoin **1** was heated in benzene to 140°C with an excess of cyclohexadiene **25**, the formation of the target products only in trace amounts was detected. The reactions of cyclohexadiene with compounds **2**-**11** were successfully carried out by catalysis of the reaction with Lewis acids; BF_3_∙Et_2_O, AlCl_3,_ and ZnI_2_ were tested in the target reactions.

It was found that cyclohexadiene **25** rapidly decomposed in the presence of BF_3_∙Et_2_O, and no products of its Diels–Alder reaction were formed. In the presence of AlCl_3_, the decomposition of the diene also occurred, which accelerated upon heating the reaction mixture; however, the target product **44a** could be isolated if diene **25** was added slowly into a refluxing solution of dienophile **1** and Lewis acid.

**Scheme 4:**
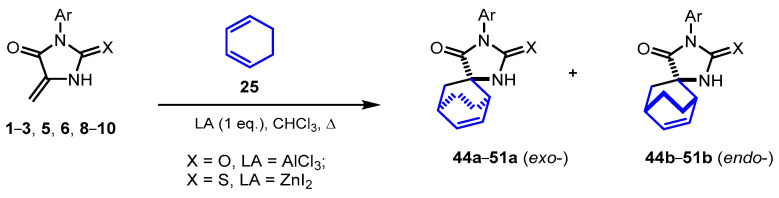
Reactions of methylidenethiohydantoins **1**-**3**, **5**, **6**, **8**-**10** with cyclohexadiene.

In the presence of 1 equivalent of ZnI_2_, cyclohexadiene did not react with 5-methylidenehydantoin **1** even when heated, but methylidenetiohydantoines **2**, **3**, **5**, **6**, **8**-**10** reacted with this diene under the same conditions to form the mixtures of diastereomeric products **45a**-**51a** and **45b**-**51b** in a ratio of ~3:1 (Scheme 4, Table 2), which could be separated by column chromatography.

It should be noted that the selectivity of the reaction is practically independent of the substituents in position 3 of the heterocycles **2**, **3**, **5**, **6**, **8**-**10**. In the presence of an excess of Lewis acid, the yields of the target products somewhat decreased, presumably due to the acceleration of the decomposition of the diene on the catalyst, and with a submolar amount of ZnI_2_, the formation of products slowed down.

Different results of the reaction of diene **25** with hydantoin **1** and thiohydantoins **2**, **3**, **5**, **6**, **8**-**10** in the presence of ZnI_2_ may be due to the fact that ZnI_2_ as a soft Lewis acid is predominantly coordinated to the sulfur atom of 2-thioimidazolone, and varying the chalcogen (O or S) strongly affects the efficiency of binding the dienophile to the Lewis acid.

The structures of spiro-imidazolones **44**-**51** were confirmed by ^1^H and ^13^C NMR spectroscopy data; the configuration of compound **46a** was determined via two-dimensional NMR techniques COSY and gHSQC (see Supplementary Information, Figures S67 and S68).

It may be noted that the ^1^H, ^13^C (and ^19^F NMR in cases where the compounds contained fluorine) spectra of all ortho-phenyl-N(3)-substituted spiro-imidazolones (for example, compounds **35**, **46,** and **51**, see Supplementary Information, Figures S23, S63, and S87 and similar) demonstrate two sets of signals. This can be explained by the hindered rotation around the single bond C(Ar)-N(imidazolone) of the imidazolone fragment and, consequently, the existence of each ortho-phenyl-substituted spiro-compounds as two atropisomers (axially chiral heterocyclic analogs of biaryl derivatives similar to those described for others ortho-substituted 2-thiohydantoins) [25,26,27]. The NMR spectra of these compounds at ambient temperatures do not indicate the presence of intramolecular dynamic processes, apparently due to the high barrier to internal rotation. Thus, weak line broadening in the ^19^F NMR spectrum of product **35a** is observed only at temperatures above 80 °C (Figure S30); this indicates that the rotational barrier is greater than 20 kcal/mol.

As in the reactions with cyclopentadiene, the isomeric exo- and endo-Diels–Alder products of the reactions of methylidenetiohydatoins **2**, **3**, **5**, **6**, **8**-**10** with cyclohexadiene can be distinguished by the chemical shifts of the protons of the CH=CH group: for the main isomers **a**, a characteristic doublet of doublets observed in the region of 6.62–6.34 ppm, and for minor products **b**, such doublet of doublets is present in the region of 6.48–6.30 ppm (in CDCl_3_).

**Figure 2:**
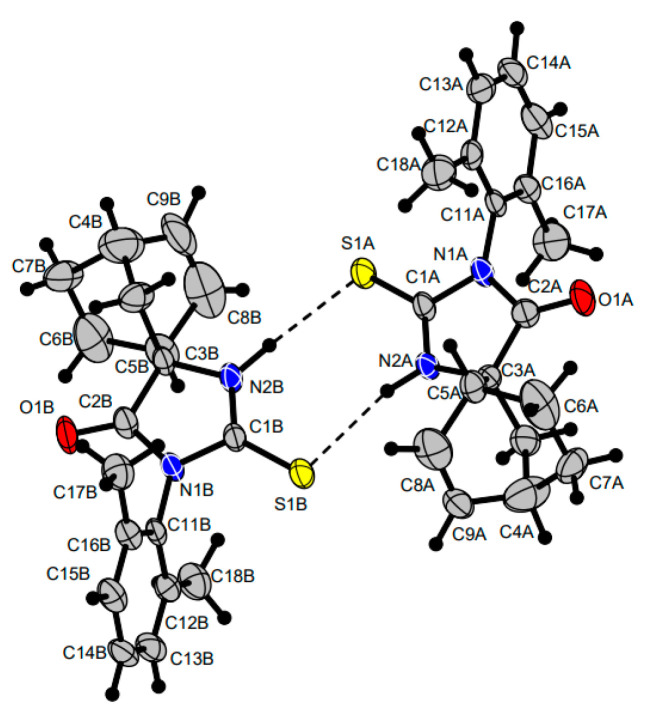
Molecular structure of compound **51a**. Thermal ellipsoids are given a 30% probability.

The structure of compound **51a** was additionally confirmed by the X-ray diffraction data. The crystal cell shown in Figure 2 includes two spiro-thiohydantoin molecules linked by NHS hydrogen bonds to form an eight-membered cycle. The dihedral angle between the planes of the cycles at the spiro junction is close to 90°; imidazolone five-membered rings are near the planar.

### 2.4. Reactions of 5-Methylideimidazolones 1-12 with 2,3-Dimethylbutadiene

**Scheme 5:**
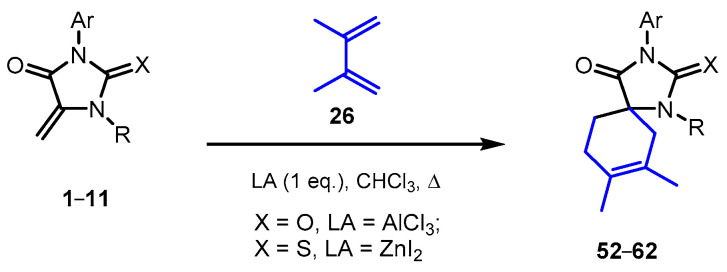
Reactions of methylideneimidazolones **1**-**12** with 2,3-dimethylbutadiene.

Reactions of methylideneimidazolones **1**-**12** with 2,3-dimethylbutadiene also require Lewis acids as the catalysis but can proceed in the presence of both AlCl_3_ and ZnI_2_ (Scheme 5, Table 3). The reactions of diene **26** with dienophiles **1** and **2** under the action of AlCl_3_ proceeded in moderate yields giving the compounds **52** and **53**.

As well as in reactions with cyclohexadiene, ZnI_2_ activates thiohydantoins **2**-**11** in the reactions with 2,3-dimethylbutadiene with the formation of target compounds **54**-**62** but does not catalyze the reactions with oxygen analogs, probably due to the selective coordination of the Lewis acid to the C=S bond of the starting heterocycle. A significant increase in the yield of thiohydantoin **53** when AlCl_3_ was replaced by the softer ZnI_2_ may be due to a decrease in the rate of the side process of diene polymerization under the action of the Lewis acid.

### 2.5. Reactions of 5-Methylideneimidazolones 1, 2, 4-6, 8, and 9 with Isoprene

**Scheme 6:**
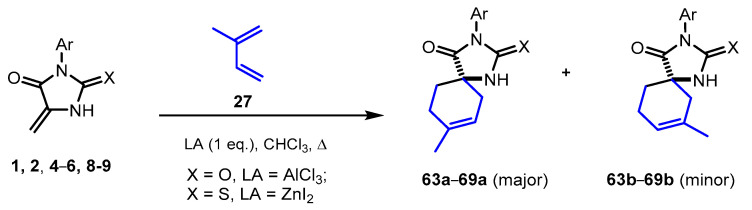
Reactions of methylideneimidazolones**1**, **2**, **4**-**6**, **8,** and **9** with isoprene.

Using isoprene **27**, the regioselectivity of the Diels–Alder reaction of methylideneimidazolones **1**, **2**, **4**-**6**, **8,** and **9** were studied. Boiling of hydantoin **1** mixture with a 10-fold excess of this diene in chloroform led to the formation of cycloaddition product **63a** in 70% yield and trace amounts of the minor regioisomer **63b**. Under similar conditions, thiohydantoins **2**, **4**-**6**, **8,** and **9** formed an inseparable mixture of regioisomers **64a**-**69a** and **64b**-**69b** in good yields in a ratio of ~87:13 (Scheme 6, Table 4 and Supplementary Information).

The structures of regioisomers were confirmed by^1^H NOESY1D,^1^H-^13^C HSQC, and^1^H-^13^C HMBC NMR spectroscopy data for the compounds **67a** and **67b** (see Supplementary Information, Figures S123–S125). In particular, the position of the methine proton in the six-membered ring of compound **67a** was confirmed by the presence of a cross peak in the HMBC spectrum, which is responsible for the vicinal interaction of this proton with the quaternary spiro-carbon.

The results obtained demonstrate that AlCl_3_ is an effective catalyst for the reactions of low-activity dienes with hydantoins and ZnI_2_ with thiohydantoins. At the same time, AlCl_3_ similarly catalyzes reactions with hydantoins and thiohydantoins (see Table 3). It may be supposed that the harder Lewis acid AlCl_3_ is coordinated to the N(1) atom of the starting imidazolone and, thus, catalyzes both reactions with hydantoins and thiohydantoins, while the softer Lewis acid ZnI_2_ is coordinated to the S atom and, thus, activates only thiohydantoins; however, exact proof of this assumption requires additional research.

### 2.6. Alkylation and Acylation of [4+2]-Cycloaddition Reaction Products

Studying the Diels–Alder reactions with methylideneimidazolones **1**-**18**, it was found that in the case of N(1)-unsubstituted dienophiles **1** and **2**, the resulting diastereomeric pair of spiroheterocycles can be successfully separated into individual isomers, while N(1)-substituted derivatives are formed either as an inseparable mixture of diastereomers or in extremely low yields (Table 1). Therefore, we studied the possibility of post-modification at the nitrogen atom of N(1)-unsubstituted Spiro derivatives **28a**, **34a**, **44a**, **45a**, **45b**, and **53**.

**Scheme 7:**
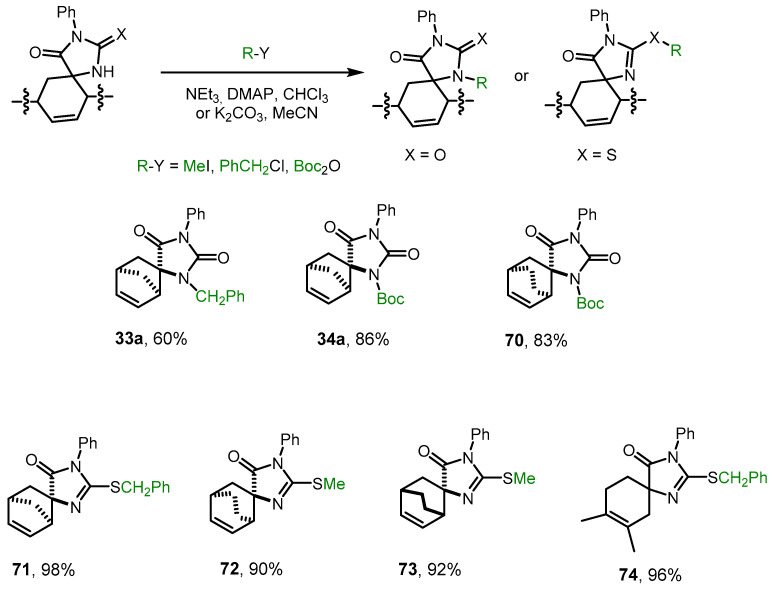
Alkylation and acylation of spirocyclic imidazolones.

We found that hydantoins **28a** and **44a** can be alkylated or acylated with PhCH_2_Cl or Boc_2_O in high yields by refluxing in chloroform or acetonitrile (Scheme 7). The spectral data of the resulting products **33a** and **34a** completely coincided with the spectral data of the main products of the Diels–Alder reaction of methylidenehydantoins **15** and **16** with cyclopentadiene (see Section 2.2).

Thiohydantoins **34a**, **45a**, **45b,** and **54** were alkylated with MeI and PhCH_2_Cl under milder conditions at room temperature in acetonitrile in the presence of K_2_CO_3_, exclusively at the sulfur atom. The formation of S–CH_2_Ph and S–Me bonds in corresponding imidazolones **72**-**75** was confirmed by ^13^C NMR spectroscopy data.

### 2.7. Desulfurization of Spiro-Thiohydantoins

Since methylidenehydantoin**1** reacted with dienes **25** and **26** with low conversion even in the presence of Lewis acids (Section 2.3 and Section 2.4), we proposed an alternative scheme for the synthesis of spirocyclic hydantoins from their thiohydantoin analogs. The procedure described in the literature for the hydrolysis of S-alkylated thiohydantoins under the action of HCl in refluxing ethanol [13,28] applied to compound **72** did not lead to the formation of the corresponding hydantoin **28a**. In the ^1^H NMR spectrum of the reaction mixture, there were no characteristic CH=CH signals of the norbornene skeleton protons, which probably indicates the ongoing processes of cationic polymerization under the reaction conditions.

**Scheme 8:**
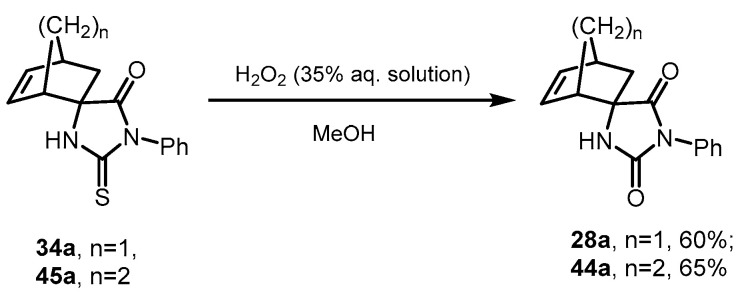
Desulfurization of spiro-thiohydantoins by H_2_O_2_.

The desired transformation of spirothiohydantoins **34a** and **45a** into spirohydantoins **28a** and **44a** was achieved under milder conditions by treating methanolic solutions of the starting compounds with 35% aqueous H_2_O_2_ at room temperature in good yields (Scheme 8).

**Scheme 9:**
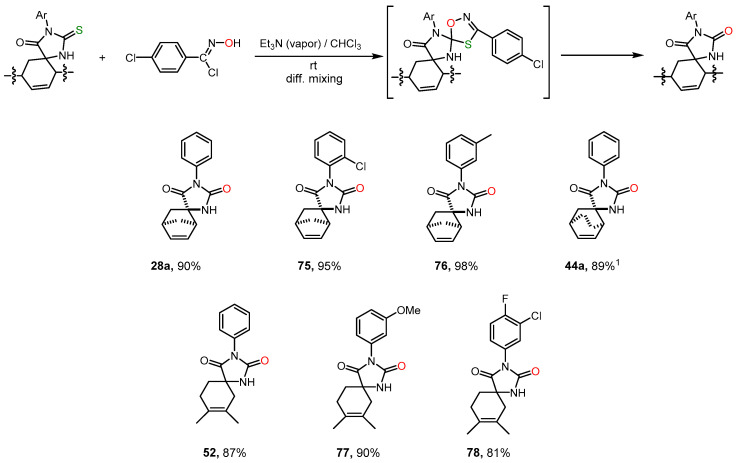
^1^ This product was isolated as an inseparable mixture with the addition products of nitrile oxide at the C=C bond. Desulfurization of spiro-thiohydantoins by the nitrile oxide action.

However, an even more effective way of thiohydantoins desulfurizing turned out to be their interaction with nitrile oxides [29] (Scheme 9). The reactive nitrile oxide was generated in situ from N-hydroxyimidoyl chloride under the action of a tertiary amine. As a result of subsequent N-hydroxyimidoyl chloride 1,3-dipolar cycloaddition at the C=S bond, an unstable oxatriazole was obtained, which then decomposed to form hydantoin, analogously to that described in [30]. For this reaction, we used a recently proposed convenient method of diffusion reagents mixing, which made it possible to suppress undesirable dimerization processes of the 1,3-dipole and introduce thiohydantoin and the dipole precursor into the reaction in a strictly equimolar ratio [31]. Under these conditions, hydantoins **28a**, **52**, **75**-**78** were formed in high yields without the side products of dipole addition to the C=C bond. During the synthesis of compound **44a**, a certain amount (no more than 10%) of the addition product of nitrile oxide to the C=C bond was formed.

### 2.8. Cytotoxicity against Human Cell Lines

Some of the obtained spiro-derivatives were tested for cytotoxicity using the standard MTT assay [32]. Cytotoxicity was evaluated using the cell lines of various etiologies: breast cancer MCF7, human lung carcinoma A549, non-cancer human embryonic kidney cell line HEK293T, and non-cancer lung fibroblast VA13 cell line. Lung cancers and breast cancers are among the most common causes of tumor lesions and related death in the world [33], and non-cancerous cells were applied for the specificity of action evaluation. The results are shown in Table 5; the dose-response dependency graphs are available in Supplementary Information.

Generally, the tested compounds show rather low or moderate cytotoxicity to all tested cell lines; their IC_50abs_ values (IC_50abs_ is the concentration resulting in a two-fold decrease in the number of cells in comparison with untreated cells) against A549 and MCF7 cell lines mostly ranging from 20 to 100 µM. Additionally, the tested compounds show little selectivity to all cell lines tested. Despite the nominal IC_50abs_ selectivity of the compounds **55** and **61** against A549 compared to VA13, their IC_50_ values (IC_50_ is the concentration resulting in half of the maximal cytotoxic effect) were quite similar (Table S1).

It may also be noticed that the introduction into the tested spiro-imidazolones of halogenated phenyl ring instead of non-halogenated one in most cases increased cytotoxicity against all tested cell lines. It can be seen by comparing the data obtained for compound **63**, which is the most cytotoxic of all the tested compounds and has a dihalogenated phenyl ring, with the data obtained for compounds **49a**, **55**, **58**, and **59**, which have monohalogenated phenyl ring, and the non-halogenated compounds **28**, **34**. The compounds with OMe- or Me-substitutient in the phenyl rings at N(3) imidazolone atom had no significant cytotoxicity on the most tested cell lines (Table 5, compounds **37**-**39**, **48**, **50**, **56**, **60**, **66**).

### 2.9. Cytotoxicity against E. coli

All compounds investigated for cytotoxicity effects were also analyzed for antibacterial activity. Tests were carried out on *E. coli* BW25113 DTC-pDualrep2 strain that has a normal cell wall and the *E. coli* BW25113 LPTD-pDualrep2strain that has damaged cell walls. Some of the tested compounds showed a noticeable antibacterial effect against *E. coli* BW25113 DTC-pDualrep2 but were almost inactive against *E. coli* BW25113 LPTD-pDualrep2. All the tested compounds were analyzed for the mechanism of antibacterial activity by means of the pDualrep2 [34] reporter system, which allows for the detection of translational inhibitors and SOS-response inducers, but none of these mechanisms is involved.

Then the minimal inhibitory concentration (MIC) was measured for *E. coli* BW25113 DTC and *E. coli* K-12 (WT) for the 12 most bacteriotoxic compounds from the plate test. MIC of compound **77** against *E. coli* BW25113 DTC is 125 µM, while the other compounds either show MIC ~500 µM, 1 mM, or are inactive in this test (Table 6).

All the tested compounds were completely inactive against *E. coli* K-12 at concentrations of 1 mM and below. It indicates that the studied compounds are toxic to *E. coli* but are effectively removed from the cell by efflux systems and, therefore, do not affect the wild type of *E. coli.*

## 3. Materials and Methods

All solvents were purified using standard procedures [35]. Starting compounds were purchased from commercial sources (Sigma–Aldrich, ABCR, AKSci, Burlington, VT, USA). Reactions were checked by TLC analysis using silica plates ^1^H, and ^13^C NMR spectra were recorded on BrukerAvance or Agilent 400-MR spectrometers (400 MHz for ^1^H, 100 MHz for ^13^C). Chemical shifts are reported in parts per million relative to TMS. 

Electrospray ionization high-resolution mass spectra were recorded on a TripleTOF 5600+ quadrupole time-of-flight mass spectrometer (ABSciex, Concord, Vaughan, ON, Canada) with DuoSpray ion source in positive ion mode. The capillary voltage was 5.5 kV; nebulizing and curtain gas pressure—15 and 25 psi, respectively; ion source temperature ambient; declustering potential 20 V; *m*/*z* range 100–1200. Elemental compositions of the detected ions were determined based on accurate masses and isotopic distributions using Formula Finder software (ABSciex, Concord, ON, Canada).

The X-Ray data for compound **51a** were collected via STOE diffractometer Pilatus100K detector, Cu Kα (1.54086Å) radiation, rotation method mode. STOE X-AREA software was used for cell refinement and data reduction. Data collection and image processing were performed with X-Area 1.67 (STOE & Cie GmbH, Darmstadt, Germany, 2013). Intensity data were scaled with LANA (part of X-Area) in order to minimize differences in intensities of symmetry-equivalent reflections (multi-scan method).

The structures were solved and refined with SHELX(1) program [36]. The non-hydrogen atoms were refined by using the anisotropic full matrix least-square procedure. Hydrogen atoms were placed in the calculated positions and allowed to ride on their parent atoms [C-H 0.93–0.98; Uiso 1.2 Ueq(parent atom)]. Hydrogen atoms at nitrogen atoms N2A and N2B (see Figure 2) were localized from Fourier syntheses and refined freely. Molecular geometry calculations were performed with the SHELX program, and the molecular graphics were prepared by using DIAMOND(2) version 4 software [37].

Some crystallographic data for **51a** are listed in Table S2; hydrogen bonds are given in Table S3 (see Supplementary part of this article); CCDC-2232689 contain the Supplementary crystallographic data. These data can be obtained free of charge from The Cambridge Crystallographic Data Centre via www.ccdc.cam.ac.uk/data_request/cif, 1 February 2023.

**Experimental procedures and characteristic data** for all synthesized compounds are given in the Supplementary Information.

**Cell cultures.** Human embryonic kidney HEK293T cell line was kindly provided by Dr. E. Knyazhanskaya; immortalized human fibroblasts cell line VA13 was kindly provided by Dr. M. Rubtsova; human breast cancer cell line MCF7 and human lung adenocarcinoma cell line A549 were kindly provided by Dr. S. Dmitriev. MCF7, VA13, A549, and HEK293T cell lines were maintained in DMEM/F-12 (Thermo Fisher Scientific, Waltham, Massachusetts, USA) culture medium containing 10% fetal bovine serum (Thermo Fisher Scientific, Waltham, Massachusetts, USA) and 50 µg/mL penicillin and 0.05 mg/mL streptomycin at 37°C (Thermo Fisher Scientific, Waltham, Massachusetts, USA) in 5% CO_2_. Cells were maintained at 37°C in a humidified incubator MCO-18AC (Sanyo, Japan) supplied with 5% CO_2_. Cell cultures were tested for the absence of mycoplasma.

**In Vitro Survival Assay (MTT Assay).** The cytotoxicity was tested using the MTT (3-(4,5-dimethylthiazol-2-yl)2,5-diphenyl tetrazolium bromide) assay [32] with some modifications. A total of 2500 cells per well for the MCF7, HEK293T cell lines, 3000 cells for the A549 cell line, or 4000 cells per well for the VA-13 cell line were plated out in 135 µL of DMEM-F12 media (Gibco, USA) in a 96-well plate and then incubated in the 5% CO_2_ incubator for first 20 h without treating. After this, 15 µL of media-DMSO solutions of tested substances were added to the cells (final DMSO concentrations in the media were 0,5% or less) and treated for65 h with 45 nM–100 µM (eight dilutions) of our substances (triplicate each) and doxorubicin as a control substance. Then the MTT reagent (Paneco LLC, Moscow, Russia) was added to cells up to the final concentration of 0.5 g/L (10 × stock solution in PBS was used), and cells were incubated for 2 h at 37 °C under an atmosphere of 5% CO_2_. Then the MTT solution was discarded, and 140 µL of DMSO (PharmaMed LLC, Russia) was added. The plates were swayed on a shaker (200 rpm) to dissolve the formazan. The absorbance was measured using a microplate reader (VICTOR ×5 Light Plate Reader, PerkinElmer, USA) at a wavelength of 555 nm (in order to measure formazan concentration). The results were used to construct dose-response graphs and to estimate IC_50abs_values (IC_50abs_ is the concentration resulting in a two-fold decrease in the number of cells in comparison with the untreated cells) and, in some cases, IC_50_ values (IC_50_ is the concentration resulting in half of the maximal cytotoxic effect) with GraphPadSoftware, Inc., San Diego, CA, USA.

***E. coli* cytotoxicity assay with a screening of Mechanism of Action.** A total of 50 µL of 20 mM solution of each compound in DMSO and DMSO as a control substance was added into wells punched in an agar plate containing a lawn of the *E. coli* BW25113 DTC-pDualrep2 or *E. coli* BW25113 LPTD-pDualrep2, which are hypersensitive to antibiotics. *E. coli* DTC [38] lacks the *tolC* gene, which encodes the outer membrane component of several multidrug transporters [39]. *E. coli* LPTD [40] has the 23-amino-acid deletion in the *lptD* gene, an inessential protein functioning in the final stages of the assembly of lipopolysaccharides into the outer membrane [41]. After overnight incubation at 37 °C, the plate was scanned with the ChemiDoc (Bio-Rad, Hercules, CA, USA) system with two channels, including “Cy3-blot” (553/574 nm, green pseudocolor) for RFP fluorescence and “Cy5-blot” (588/633 nm, red pseudocolor) for Katushka2S fluorescence. Cells without reporter construction could be detected in both channels. The induction of the expression of Katushka2S is triggered by translation inhibitors, while RFP is up-regulated by the induction of DNA damage and SOS response. In addition, 2 µL of levofloxacin (25 µg/mL) and erythromycin (5 mg/mL) were used as positive controls for DNA biosynthesis and ribosome inhibitors, respectively.

**Determination of Minimal Inhibitory Concentration (MIC).** MIC values were determined by monitoring growth in 96-well plates of E. coli cultures exposed to serial dilutions of compounds. Specifically, overnight *E. coli* BW25113 DTC and K12 cultures were diluted in 96-well plates to an OD590 of 0.01 in LB medium. The wells were then supplemented with the solution of each tested compound at concentrations of 1 mM, 500 µM, 250 µM, 125 µM, 62.5 µM, 31.25 µM, 15.63 µM, 7.81 µM, 3.91 µM, and 1.95 µM. Then the plates were incubated with shaking (200 r.p.m.) overnight at 37°C, and cell growth was assessed by scanning OD590 each well with a VictorX5 reader.

## 4. Conclusions

In the present study, a series of new spiro-derivatives containing hydantoin and thiohydantoin fragments was synthesized by [4+2]-cycloaddition of 1,3-dienes (cyclopentadiene, cyclohexadiene, 2,3-dimethylbutadiene, isoprene)to 5-methylidene-substituted imidazolones (hydantoins or thiohydantoins). It was shown that the studied cycloaddition reactions proceed regioselectively and stereoselectively. Reactions of methylideneimidazolones with cyclopentadiene proceed by co-heating the reactants; reactions with cyclohexadiene, 2,3-dimethylbutadiene, and isoprene require catalysis by Lewis acids. It was demonstrated that ZnI_2_ is an effective catalyst for the interaction of methylidenethiohydantoins with non-activated dienes. The possibility of alkylation and acylation of the obtained spiro-hydantoinsat the N(1)nitrogen atoms with PhCH_2_Cl or Boc_2_O and the alkylation of the spiro-thiohydantoinsat the S atoms with MeI or PhCH_2_Cl in high yields have been demonstrated. The preparativetransformation of spiro-thiohydantoins into corresponding spiro-hydantoinsin mild conditions by treating with 35% aqueous H_2_O_2_ or nitrile oxide has been carried out.

Some synthesized spiro-imidazolones in preliminary biological tests demonstrated moderate cytotoxic activity on MCF7, A549, HEK293T, and VA13 cell lines. They show only little (at concentrations more than 100 µM) antibacterial effects in the experiments with wild type of *E. coli*.

## Figures and Tables

**Table 1:** Diels–Alder reactions of methylideneimidazolone derivatives with cyclopentadiene.

Products	X	R_1_	R_2_	R_3_	*exo*:*endo*^1^	Yield, %
**28a + 28b**	O	Ph	H	H	91:9	81 + 9 ^2,3^ [24]
**29a + 29b**	O	Ph	Ac	H	77:23	73 (78:22) ^3,4^
**30a +30b**	O	Ph	COOEt	H	79:21	83 (83:17) ^3,4^
**31a +31b**	O	Ph	Ts	H	87:13	91 (89:11) ^3,4^
**32a + 32b**	O	Ph	Boc	H	85:15	73 (85:15) ^3,4^
**33a**	O	Ph	CH_2_Ph	H	100:0	10 ^3,5^
**34a + 34b**	S	Ph	H	H	91:9	79 + 8 ^2,3^ [24]
**35a + 35b**	S	2-FC_6_H_4_	H	H	91:9	71 + 7 ^2,3^
**36a + 36b**	S	2-ClC_6_H_4_	H	H	91:9	68 + 0 ^2,3^
**37a + 37b**	S	3-MeC_6_H_4_	H	H	91:9	80 + 8 ^2,3^
**38a + 38b**	S	3-MeOC_6_H_4_	H	H	91:9	81 + 9 ^2,3^
**39a + 39b**	S	4-MeOC_6_H_4_	H	H	91:9	78 + 8 ^2,3^
**40a**	O	Ph	H	Br	100:0	64 ^6,7^
**41a**	O	Ph	H	I	100:0	37 ^6,7^
**42a**	O	H	H	4-FC_6_H_4_	-	0^7^
**43a**	S	Ph	H	4-MeOC_6_H_4_	-	0^7^

^1^ Based on ^1^H NMP spectra of reaction mixture. ^2^ Isolated yields for major and minor isomers. ^3^ Conditions: cyclopentadiene (8 equivalents.), CHCl_3_, reflux, 6h. ^4^ Isolated yields for the isomers mixture; isomers ratio is indicated in the parentheses. ^5^ Yield by ^1^H NMP spectra of reaction mixture. ^6^ Isolated yields. ^7^ Conditions: cyclopentadiene (20 equivalents), o-xylene, reflux, 13 h.

**Table 2:** Diels–Alder reactions of 5-methylideimidazolones with cyclohexadiene.

Products	X	LA	Ar	Yield a ^1^, %	Yield b ^1^, %
**44a + 44b**	O	AlCl_3_	Ph	16	0
		ZnI_2_		0	0
**45a + 45b**	S	ZnI_2_	Ph	54	19
**46a + 46b**	S	ZnI_2_	2-FC_6_H_4_	44	15
**47a + 47b**	S	ZnI_2_	3-MeC_6_H_4_	60	25
**48a + 48b**	S	ZnI_2_	3-MeOC_6_H_4_	51	21
**49a + 49b**	S	ZnI_2_	4-ClC_6_H_4_	55	20
**50a + 50b**	S	ZnI_2_	4-MeOC_6_H_4_	50	19
**51a + 51b**	S	ZnI_2_	2,6-Me_2_C_6_H_3_	44	15

^1^ Isolated yields.

**Table 3:** Diels–Alder reactions of methylideneimidazolones with 2,3-dimethylbutadiene.

Product	X	LA	Ar	R	Yield ^1^, %
**52**	O	AlCl_3_	Ph	H	47
		ZnI_2_			0
**53**	S	AlCl_3_	Ph	H	45
		ZnI_2_			92
**54**	S	ZnI_2_	2-FC_6_H_4_	H	84
**55**	S	ZnI_2_	2-ClC_6_H_4_	H	71
**56**	S	ZnI_2_	3-MeC_6_H_4_	H	85
**57**	S	ZnI_2_	3-MeOC_6_H_4_	H	79
**58**	S	ZnI_2_	4-FC_6_H_4_	H	74
**69**	S	ZnI_2_	4-ClC_6_H_4_	H	82
**60**	S	ZnI_2_	4-MeOC_6_H_4_	H	88
**61**	S	ZnI_2_	2,6-Me_2_C_6_H_3_	H	68
**62**	S	ZnI_2_	3-Cl,4-FC_6_H_3_	H	81

^1^ isolated yields.

**Table 4:** Diels–Alder reactions of methylideneimidazolones with isoprene.

Product	X	LA	Ar	Yield a + b ^1^, %
**63a + 63b**	O	AlCl_3_	Ph	70
**64a + 64b**	S	ZnI_2_	Ph	82
**65a + 65b**	S	ZnI_2_	2-ClC_6_H_4_	61
**66a + 66b**	S	ZnI_2_	3-MeC_6_H_4_	64
**67a + 67b**	S	ZnI_2_	3-MeOC_6_H_4_	76
**68a + 68b**	S	ZnI_2_	4-ClC_6_H_4_	68
**69a + 69b**	S	ZnI_2_	4-MeOC_6_H_4_	71

^1^ isolated yields.

**Table 5:** Cytotoxicity effects of the compounds calculated as IC_50abs_ (IC_50abs_—concentration of compounds, which caused 50% death of cells) for 65-h-treatment of the cell lines of different etiology using the standard MTT assay.

			Cell line			
Compound	HEK293T	MCF7	VA13	A549	C_max_	Units
**28a**	>>100	>>100	>>100	>>100	100	µM
**28b**	>>100	>>100	>>100	>>100	100	µM
**34a**	83.0 ± 3.7	>>100	>>100	>>100	100	µM
**35b**	87.4 ± 9.9	>>100	>>100	>>100	100	µM
**37a**	>>100	>>100	>>100	>>100	100	µM
**37b**	115 ± 17 **	>>100	>>100	>>100	100	µM
**38a**	111 ± 9 **	>>100	>>100	>>100	100	µM
**38b**	112 ± 8 **	>>100	>>100	>>100	100	µM
**39b**	120 ± 13 **	>>100	>>100	>>100	100	µM
**40a**	56.4 ± 2.5	>>100	>>100	>>100	100	µM
**41a**	69.1 ± 4.8	>>100	>>100	>>100	100	µM
**45a**	21.3 ± 2.3	141 ± 28 **	>>100	85.2 ± 10.1	100	µM
**48a**	98.0 ± 9.7	>>100	>>100	>>100	100	µM
**48b**	108 ± 12 **	>>100	>>100	>>100	100	µM
**49a**	6.7 ± 0.7	37.8 ± 6.1	30.9 ± 3.4	16.9 ± 1.9	100	µM
**50a**	41.9 ± 3.8	>>100	>>100	>>100	100	µM
**50b**	109 ± 12 **	>>100	>>100	>>100	100	µM
**51a**	>>100	>>100	>>100	>>100	100	µM
**51b**	87.5 ± 7.2	104 ± 11 **	>>100	140 ± 21 **	100	µM
**54**	29.6 ± 3	122 ± 29 **	>>100	49.5 ± 5	100	µM
55 *	~5	~100	>>100	~10	100	µM
**56**	22.0 ± 8.0	102 ± 17 **	98.7 ± 17.6	>>100	100	µM
**57**	18.3 ± 1.7	50.6 ± 5.2	80.8 ± 13.7	35.4 ± 4.8	100	µM
**58**	13.7 ± 2.3	59.0 ± 13.3	45.0 ± 7.5	23.2 ± 3.5	100	µM
**59**	4.4 ± 0.6	76.6 ± 16	52.6 ± 7.8	26.2 ± 3.9	100	µM
**60**	10.2 ± 1.3	98.3 ± 19.8	75.3 ± 17	52.2 ± 5.7	100	µM
61 *	~4	~100	N/A	~10	100	µM
**62**	6.8 ± 0.3	11.1 ± 0.6	9.8 ± 0.4	7.9 ± 0.4	100	µM
**66a + 66b**	44.6 ± 4.9	146 ± 22 **	110 ± 13 **	144 ± 36 **	100	µM
**72**	60.1 ± 3.4	>>100	>>100	>>100	100	µM
**75**	99.0 ± 11.1	>>100	>>100	>>100	100	µM
**76**	48.6 ± 2.4	86.4 ± 7.3	132 ± 17 **	135 ± 14 **	100	µM

C_max_—the highest concentration studied in the test.>>100—compound shows little or no cytotoxicity. IC_50abs_ values could not be calculated. *—approximate IC_50abs_ values are given, see IC_50_ values in Table 6. **—IC_50abs_ value, calculated by data extrapolation, exceeds C_max_.N/A—not available.

**Table 6:** Antibacterial effects shown as MIC for *E. coli* BW25113 DTC and *E. coli* K-12. MIC values are given in µM.

Strain	28a	28b	35b	40a	41a	51a	55	60	62	72	75	76
*E. coli* DTC	>1000	>1000	1000	1000	1000	>1000	>1000	>1000	1000	500	500	125
*E. coli* K12	>1000	>1000	>1000	>1000	>1000	>1000	>1000	>1000	>1000	>1000	>1000	>1000

>1000—compound is inactive in this test.

## Data Availability

The data is available upon request from the authors.

## Supplementary Materials

The following supporting information can be downloaded at: https://www.mdpi.com/article/10.3390/ijms24055037/s1: the experimental details.
