# Supplementary material for: [4+2]-Cycloaddition to 5-Methylidene-Hydantoins and 5-Methylidene-2-Thiohydantoins in the Synthesis of Spiro-2-Chalcogenimidazolones
Source: Int J Mol Sci. 2023 Mar 6;24(5):5037. doi: 10.3390/ijms24055037 (PMC10002963; doi:10.3390/ijms24055037)
Supplement: Supplementary file 1 [file ijms-24-05037-s001.zip › ijms-2260039-supplementary.pdf]

[4+2]-Cycloaddition to 5-methylidene-hydantoins and 5-methylidene-2-thiohydantoins in synthesis of spiro-2-chalcogenimidazolones

Dmitry E. Shybanov[1], Maxim E. Kukushkin[1], Yuri K. Grishin[1], Yanislav S. Hrytseniuk[1], Vitaly A. Roznyatovsky[1], Viktor A. Tafeenko[1], Dmitry A. Skvortsov[1], Nikolai V. Zyk[1] and Elena K. Beloglazkina[1,*]

[1] Department of Chemistry, M. V. Lomonosov Moscow State University, 119991 Moscow, Russian Federation.

Supplementary Information

Table of Contents

General Procedure for the synthesis of methylidenehydantoins **12-15**……………….……..……………..3

Synthesis of methylidenehydantoin **16**……………………………………..………………………..……7

General procedure for the synthesis of 5-halogenomethylidenehydantoins **17, 18**………………………9

Synthesis of 5-halogenmethylidenethiohydantoin **21**…………………………………………..………11

General procedure of Diels-Alder reaction of methylideneimidazolones **1-6, 9, 12-16** with cyclopentadiene ………………………………...…………………….…………………………..….12

General procedure of Diels-Alder reaction of 5-halomethylidenehydantoins **17, 18** with cyclopentadiene
………………………………..…………………………………………………………...…….34

General procedure of Diels-Alder reaction of methylideneimidazolones **1-3, 5, 6, 8-10** with 1,3-cyclohexadiene ……………………………………………………………………….…….37

General procedure of Diels-Alder reaction of methylideneimidazolones **1-12** with 2,3-dimethylbuta-1,3-diene
…………………………………………………………………………………………..….57

General procedure of Diels-Alder reaction of methylideneimidazolones **1, 2, 4-6, 8, 9** with isoprene ………..69

General procedure for alkylation of imidazolones **28a, 34a, 45a, 45b, 53** ……………………………….79

General procedure of acylation of hydantoins **28a, 44a** ……………………..…………………….…….85

General procedure of desulfurization of thiohydantoins **34a, 45a** with hydrogen peroxide.…………….88

General procedure of desulfurization of thiohydantoins **34a, 36a, 37a, 53, 57, 62** with 1,3-dipolar cycloaddition of nitrile oxides using diffusion mixing technique ………………………………..…….89

Cytotoxicity study: concentration-cytotoxicity dependencies (by MTT test)….…….…………………..….94

Cytotoxicity study: Table 1S. Cytotoxicity IC_{50} values for the compounds **55** and **61** for HEK293T, MCF7, VA13 and A549 cell lines …………………………………………………………………….….96

X-Ray data ……………………………………………………………………………………..….97

References……………………………………………………………………………………..……..98

General Procedure for the synthesis of methylidenehydantoins 12-15.

To a solution of methylidenehydantoin **1** (0.27 mmol) and acylation agent (0.30 mmol) in chloroform (30 ml) triethylamine (30 mg, 0.30 mmol) and DMAP (3 mg, 0.02 mmol) were added and the mixture was stirred overnight. The resulting mixture was washed with 2% aqueous HCl (2 x 15 ml). The organic phase was dried over anhydrous Na_2SO_4 and filtered. The solvent was then removed in vacuo, and the residue was purified by column chromatography on silica gel.

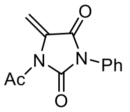

1-acetyl-5-methylene-3-phenylimidazolidine-2,4-dione (**12**) (isolated using chloroform as eluent). From methylideneimidazolone **1** (51 mg, 0.27 mmol), Ac_2O (31 mg, 0.30 mmol), triethylamine (30 mg, 0.30 mmol) and DMAP (3 mg, 0.02 mmol) compound **12** (59 mg, 95%) was obtained as a pale yellow solid.
The spectral data are consistent with the literature data [1].

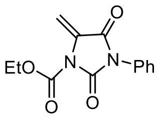

Ethyl 5-methylene-2,4-dioxo-3-phenylimidazolidine-1-carboxylate (**13**) (isolated using chloroform as eluent). From methylideneimidazolone **1** (51 mg, 0.27 mmol), ClCOOEt (33 mg, 0.30 mmol), triethylamine (30 mg, 0.30 mmol) and DMAP (3 mg, 0.02 mmol) compound **13** (62 mg, 88%) was obtained as a pale yellow oil.

^{1}H NMR (400 MHz, $CDCl_3$): δ 7.53-7.44 (m, 2H), 7.43-7.38 (m, 3H), 6.23 (d, J = 1.2 Hz, 1H), 6.03 (d, J = 1.2 Hz, 1H), 4.47 (q, J = 7.1 Hz, 2H), 1.42 (d, J = 7.1 Hz, 3H). **^{13}C NMR** (101 MHz, $CDCl_3$): δ 160.1, 149.8, 149.3, 131.6, 130.5, 129.3 (2C), 129.0, 126.4 (2C), 106.6, 64.3, 14.2. **HRMS** (ESI+) m/z calcd. for ($C_{13}H_{13}N_2O_4$, M+H): 261.0870, found: (M+H): 261.0867.

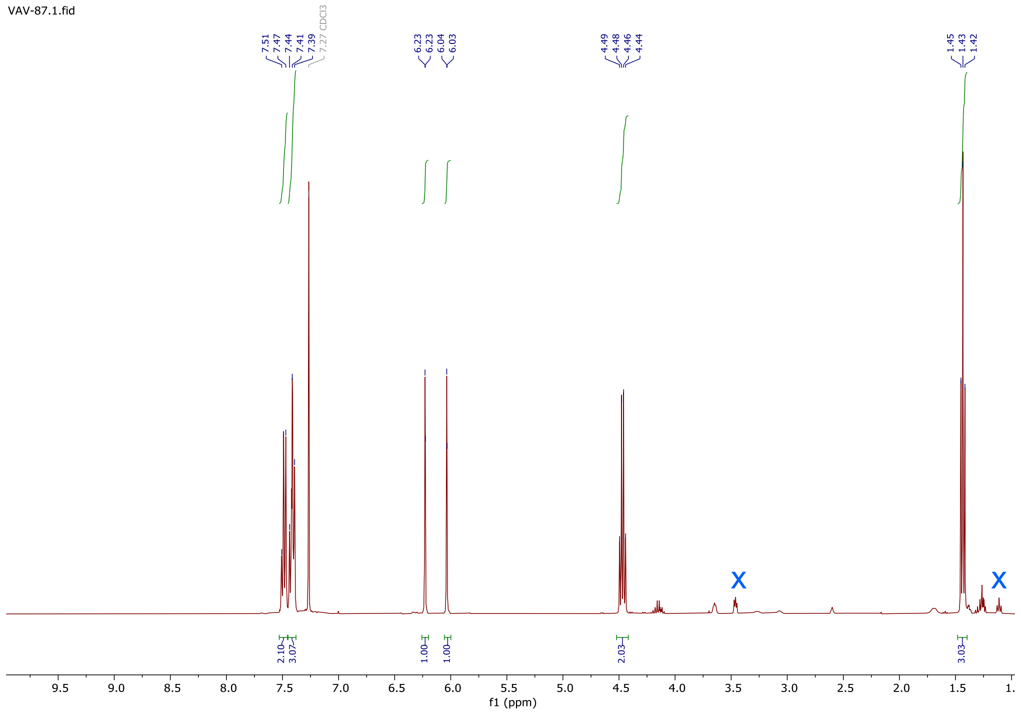

Figure S1. ^{1}H NMR spectra of compound **13** (*the sign "x" marks the residual signals of* Et_3N).

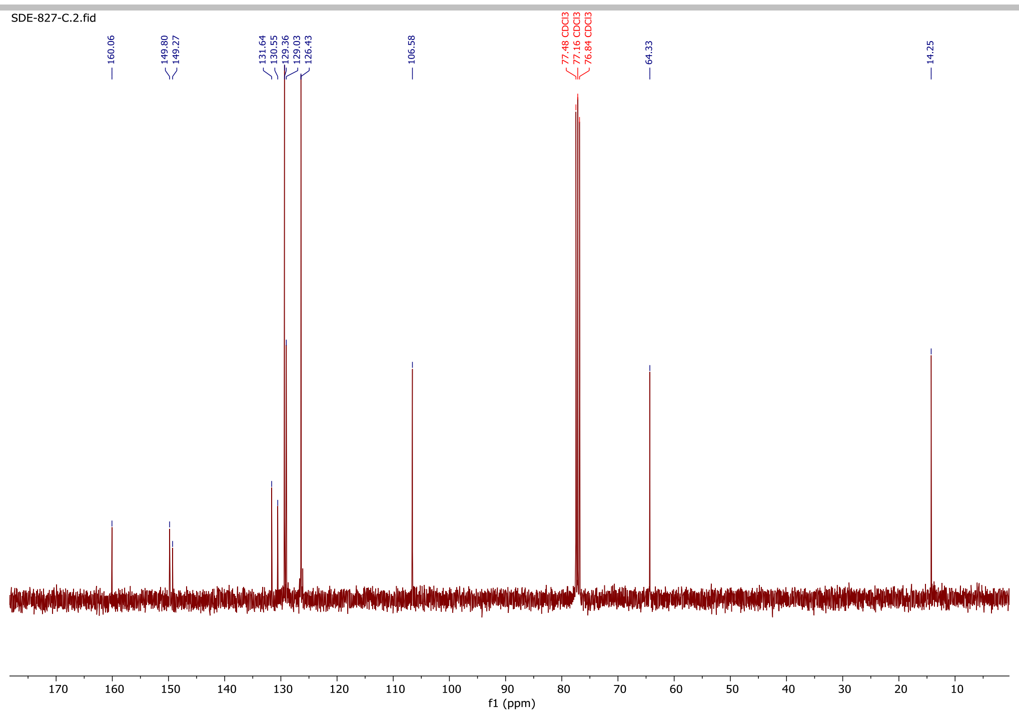

Figure S2. ^{13}C NMR spectra of compound **13.**

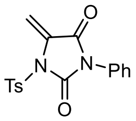

5-methylene-3-phenyl-1-tosylimidazolidine-2,4-dione (**14**) (isolated using chloroform as eluent). From methylideneimidazolone **1** (51 mg, 0.27 mmol), TsCl (57 mg, 0.30 mmol), triethylamine (30 mg, 0.30 mmol) and DMAP (3 mg, 0.02 mmol) compound **14** (74 mg, 80%) was obtained as a white crystalline solid.

^{1}**H NMR** (400 MHz, $CDCl_3$): δ 8.02-7.97 (m, 1H), 7.49-7.32 (m, 7H), 6.23 (d, J = 1.0 Hz, 1H), 5.96 (d, J = 1.0 Hz, 1H), 2.46 (s, 3H). ^{13}**C NMR** (101 MHz, $CDCl_3$): δ 159.7, 149.8, 146.7, 134.5, 131.6, 130.2 (2C), 129.3 (2C), 129.0 (2C), 128.5 (2C), 126.0 (2C), 104.7, 21.9. **HRMS** (ESI+) m/z calcd. for ($C_{17}H_{15}N_2O_4S$, M+H): 343.0747, found: (M+H): 343.0750.

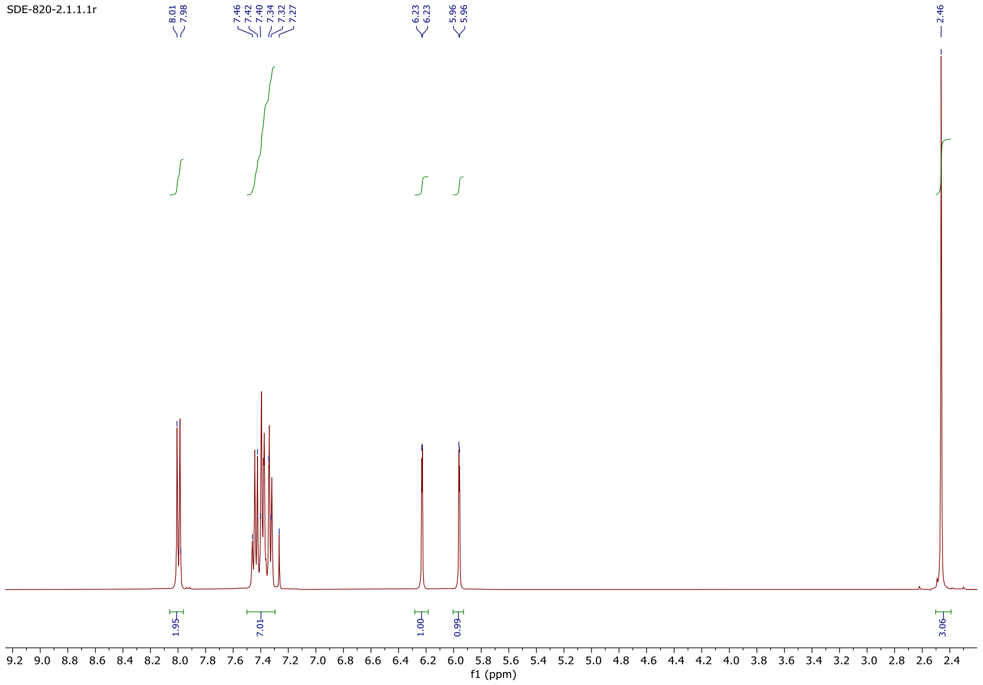

Figure S3. ¹H NMR spectra of compound **14.**

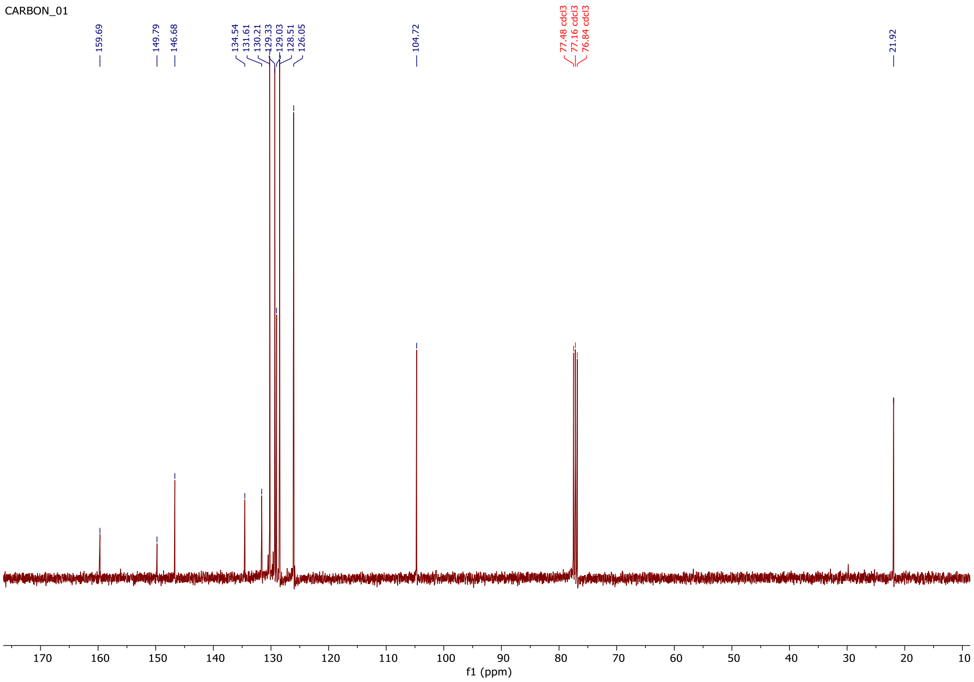

Figure S4. ¹³C NMR spectra of compound **14.**

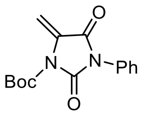

Tert-butyl 5-methylene-2,4-dioxo-3-phenylimidazolidine-1-carboxylate (**15**) (isolated using chloroform as eluent). From methylideneimidazolone **1** (51 mg, 0.27 mmol), Boc_2O (65 mg, 0.30 mmol), triethylamine (30 mg, 0.30 mmol) and DMAP (3 mg, 0.02 mmol) compound **15** (73 mg, 94%) was obtained as a pale yellow oil.

¹H NMR (400 MHz, $CDCl_3$): δ 7.49-7.45 (m, 3H), 7.41-7.38 (m, 2H), 6.14 (s, J = 1.0 Hz, 1H), 5.98 (d, J = 1.0 Hz, 1H), 1.61 (s, 9H). **¹³C NMR** (101 MHz, $CDCl_3$): δ 160.1, 149.4, 147.9, 131.8, 130.5, 129.2 (2C), 128.8, 126.4 (2C), 105.9, 85.6, 28.0 (3C). **HRMS** (ESI+) m/z calcd. for ($C_{15}H_{17}N_2O_4$, M+H): 289.1183, found: (M+H): 289.1183.

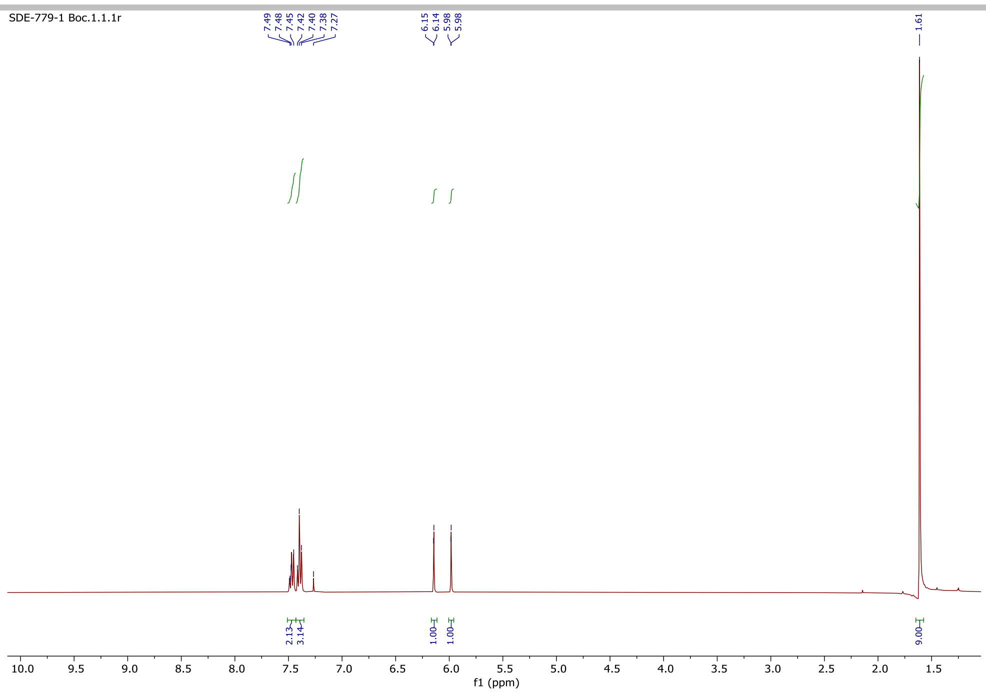

Figure S5. ^{1}H NMR spectra of compound **15.**

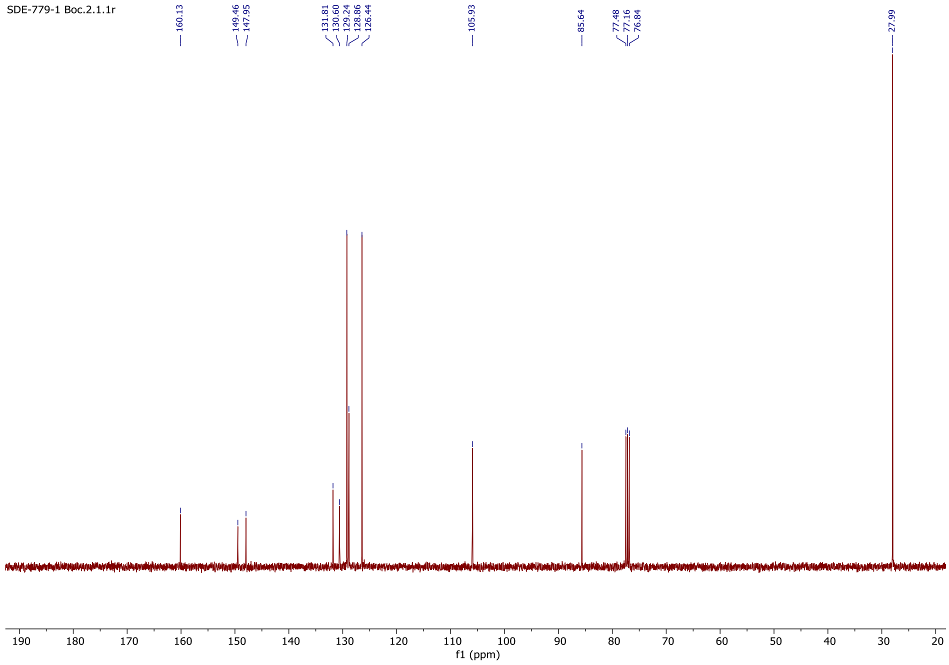

Figure S6. ^{13}C NMR spectra of compound **15.**

Synthesis of methylidenehydantoin 16.

To a solution of methylidenehydantoin **1** (0.27 mmol) and K_2CO_3 (0.54 mmol) in acetonitrile (30 ml) $PhCH_2Cl$ (0.27 mmol) was added and the mixture was stirred overnight. The solvent was then removed in vacuo, the residue was dissolved in chloroform (20 mL), the insoluble solid was filtered off, and the solution was concentrated under reduced pressure giving a target compound.

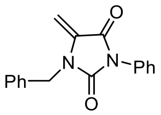

(1-benzyl-5-methylene-3-phenylimidazolidine-2,4-dione (**16**). From methylideneimidazolone **1** (51 mg, 0.27 mmol), K_2CO_3 (75 mg, 0.54 mmol) and $PhCH_2Cl$ (34 mg, 0.27 mmol) compound **16** (71 mg, 95%) was obtained as a colorless oil.

^{1}H NMR (400 MHz, $CDCl_3$): δ 7.52-7.49 (m, 4H), 7.37-7.31 (m, 6H), 5.49 (d, J = 2.4 Hz, 1H), 4.89 (s, 2H), 4.82 (d, J = 2.4 Hz, 1H). **^{13}C NMR** (101 MHz, $CDCl_3$): δ 161.4, 153.4, 135.2, 135.0, 131.5, 129.2 (2C), 129.0 (2C), 128.3, 128.2, 127.6 (2C), 126.0 (2C), 96.2, 44.5. **HRMS** (ESI+) m/z calcd. for ($C_{17}H_{15}N_2O_2$, M+H): 279.1128, found: (M+H): 279.1121.

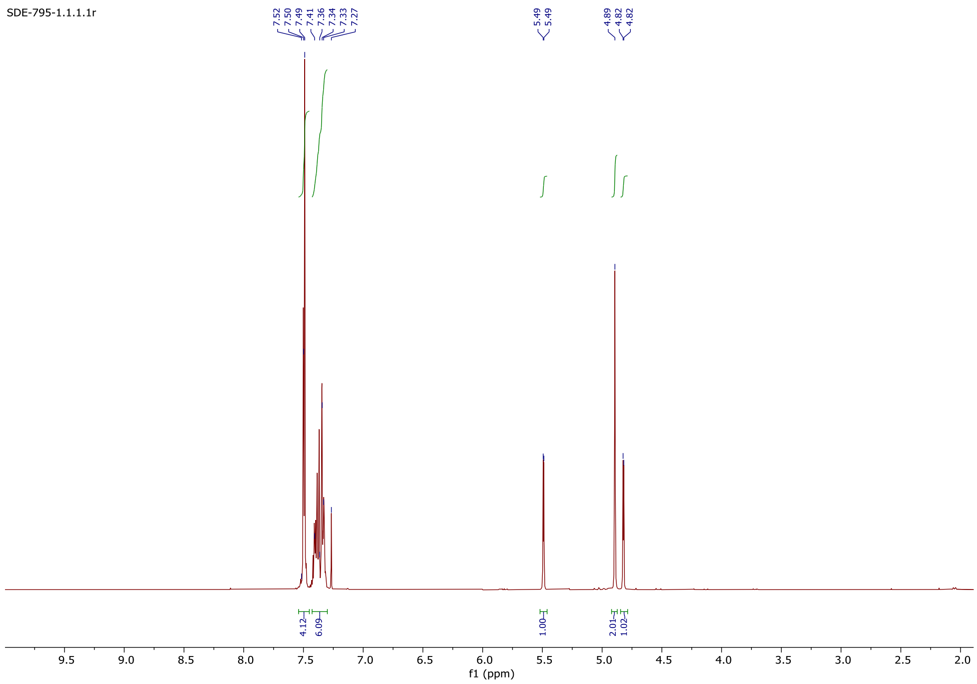

Figure S7. ^{1}H NMR spectra of compound **16.**

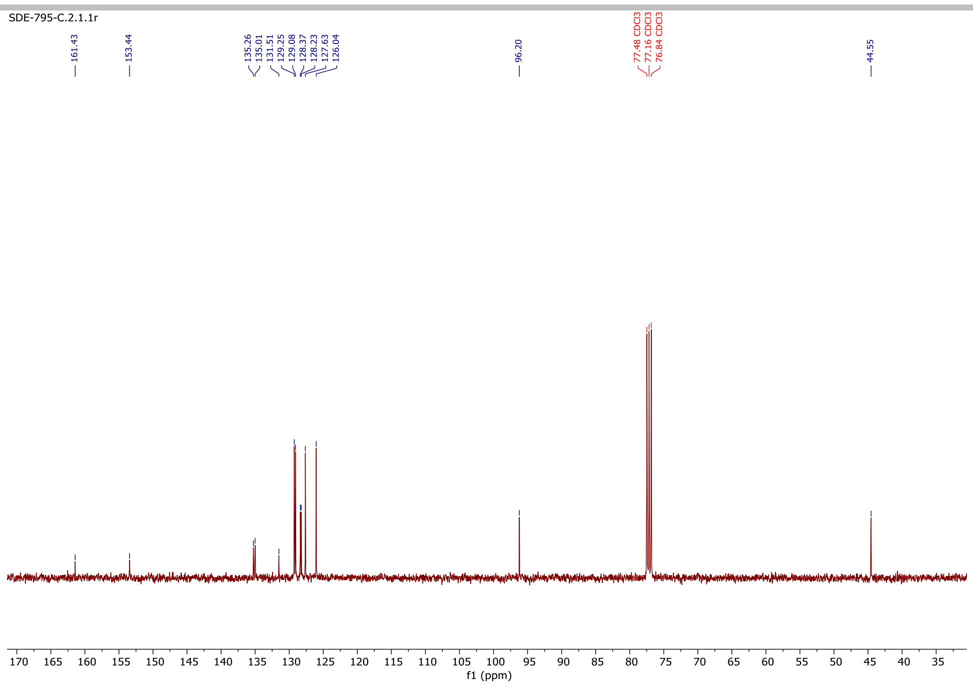

Figure S8. ^{13}C NMR spectra of compound **16.**

General procedure for the synthesis of 5-halogenomethylidenehydantoins 17, 18.

To a solution of methylidenehydantoin **1** (0.27 mmol) and Hal_2 (0.30 mmol) in chloroform (30 ml) triethylamine (30 mg, 0.30 mmol) was added and the mixture was stirred for 1 h. The solvent was then removed in vacuo, the residue was purified by column chromatography on silica gel.

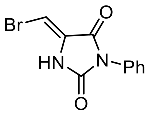

(Z)-5-(bromomethylene)-3-phenylimidazolidine-2,4-dione (**17**) (isolated using methanol/chloroform (1:200) as eluent). From methylideneimidazolone **1** (51 mg, 0.27 mmol), Br_2 (48 mg, 0.30 mmol) and triethylamine (30 mg, 0.30 mmol) compound **17** (71 mg, 98%) was obtained as a white crystalline solid.

^{1}H NMR (400 MHz, DMSO-d6): δ 11.16 (bs, 1H), 7.52–7.44 (m, 2H), 7.44–7.37 (m, 3H), 6.81 (s, 1H). **^{13}C NMR** (101 MHz, DMSO-d6): δ 160.1, 153.1, 133.6, 131.5, 128.8 (2C), 128.2, 127.0 (2C), 92.1. **HRMS** (ESI+) m/z calcd. for ($C_{10}H_8BrN_2O_2$, M+H): 266.9763, found: (M+H): 266.9760.

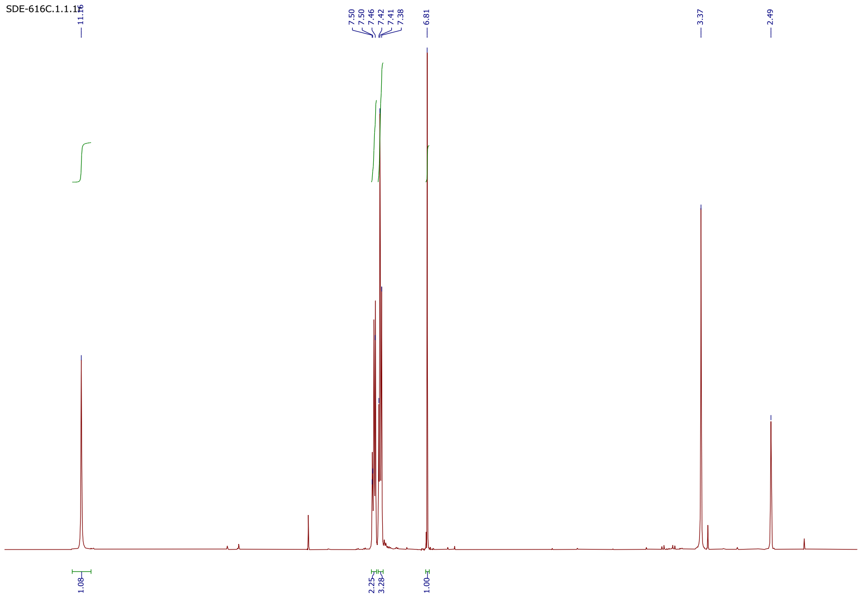

Figure S9. ^{1}H NMR spectra of compound **17.**

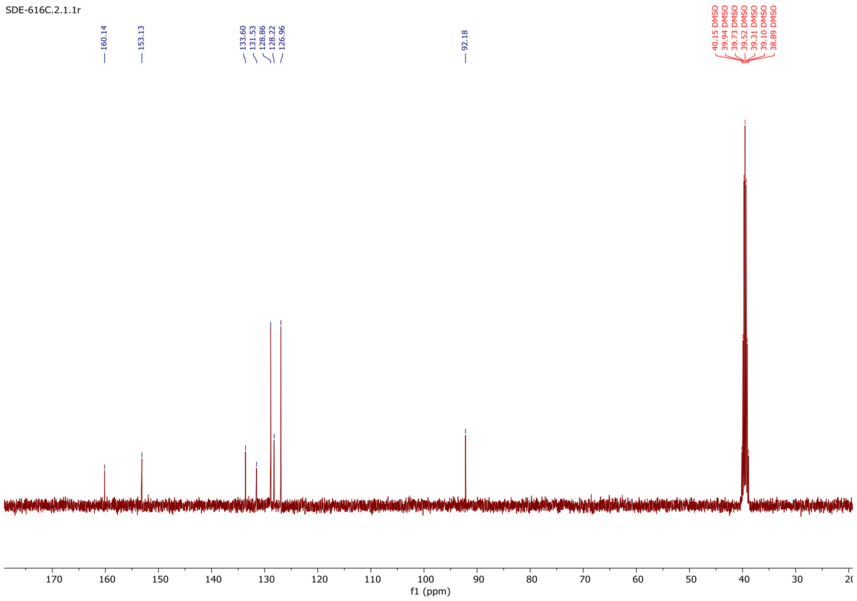

Figure S10. ^{13}C NMR spectra of compound **17.**

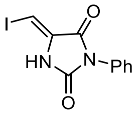

(Z)-5-(iodomethylene)-3-phenylimidazolidine-2,4-dione (**18**) (isolated using methanol/chloroform (1:200) as eluent). From methylideneimidazolone **1** (51 mg, 0.27 mmol), I_2 (76 mg, 0.30 mmol) and triethylamine (30 mg, 0.30 mmol) compound **18** (79 mg, 93%) was obtained as a pale yellow crystalline solid.

^{1}H NMR (400 MHz, DMSO-d6): δ 10.94 (bs, 1H), 7.52-7.45 (m, 2H), 7.45-7.37 (m, 3H), 6.77 (s, 1H). **^{13}C NMR** (101 MHz, DMSO-d6): δ 159.3, 153.2, 138.3, 131.7, 128.8 (2C), 128.1, 126.9 (2C), 64.3. **HRMS** (ESI+) m/z calcd. for ($C_{10}H_8IN_2O_2$, M+H): 314.9625, found: (M+H): 314.9628.

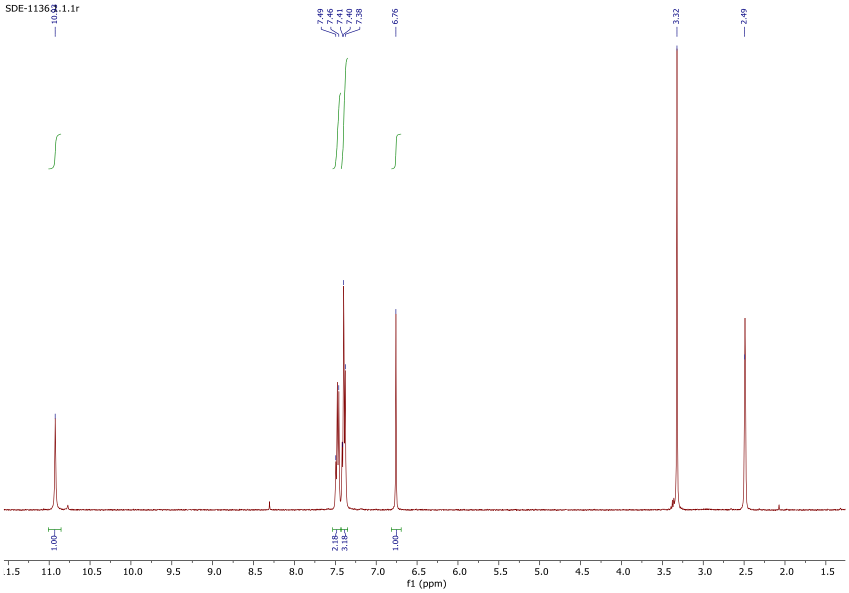

Figure S11. ^{1}H NMR spectra of compound **18.**

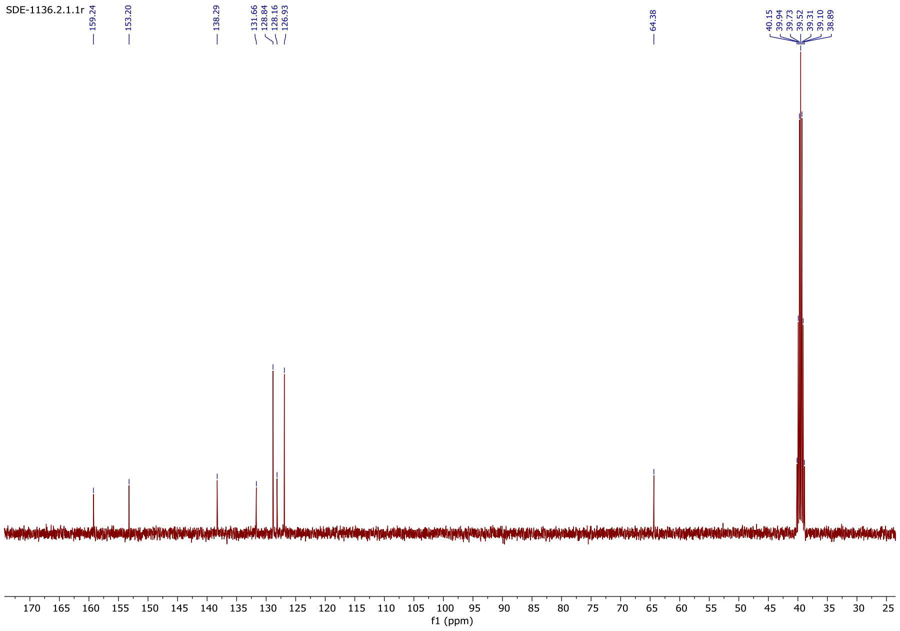

Figure S12. ^{13}C NMR spectra of compound **18.**

Synthesis of 5-halogenmethylidenethiohydantoin 21.

To a solution of methylidenethiohydantoin **2** (0.98 mmol) in chloroform (30 ml) at 0°C the solution of Br_2 (157 mg, 0.98 mmol) in chloroform (20 ml) was added dropwise and the mixture was stirred for 1 h. The solvent was then removed in vacuo, the residue was purified by column chromatography on silica gel.

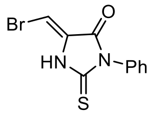

(Z)-5-(bromomethylene)-3-phenyl-2-thioxoimidazolidin-4-one (**21**) (isolated using chloroform as eluent). From methylideneimidazolone **2** (200 mg, 0.98 mmol) and Br_2 (157 mg, 0.98 mmol) compound **21** (25 mg, 9%) was obtained as a light yellow crystalline solid.

^{1}H NMR (400 MHz, DMSO-d6): δ 12.71 (bs, 1H), 7.51-7.42 (m, 3H), 7.36-7.32 (m, 3H), 6.95 (s, 1H). **^{13}C NMR** (101 MHz, DMSO-d6): δ 178.7, 160.8, 133.9, 133.0, 129.0 (3C), 128.9 (2C), 94.9. **HRMS** (ESI+) m/z calcd. for ($C_{10}H_8BrN_2OS$, M+H): 282.9535, found: (M+H): 282.9530.

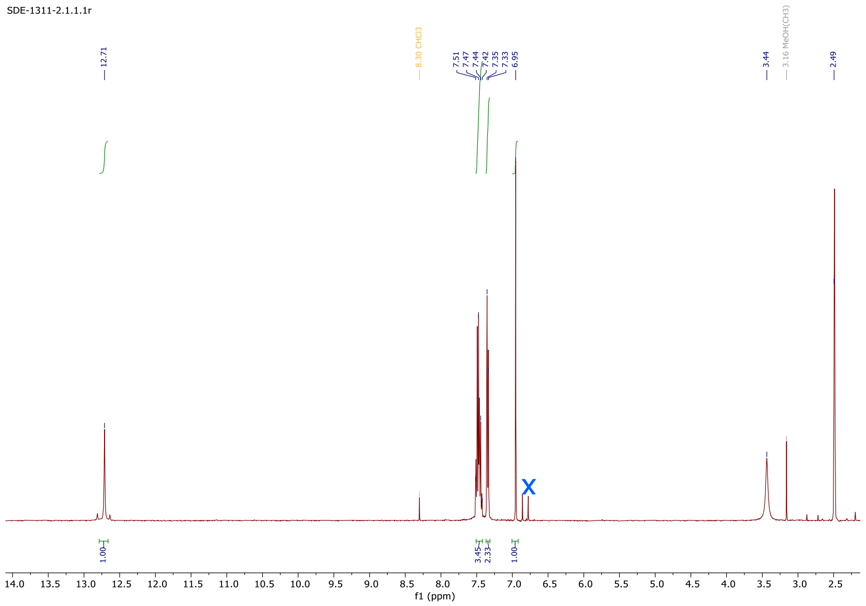

Figure S13. ^{1}H_NMR spectra of compound **21** (*the sign "x" marks the residual signals of CHBr group of (E)-isomer*).

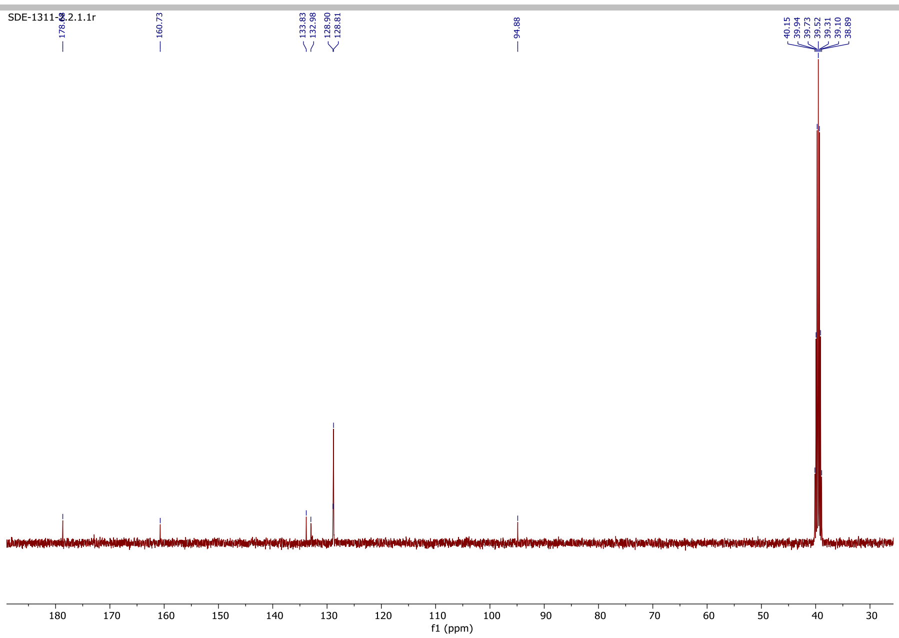

Figure S14. ^{13}C NMR spectra of compound **21.**

General procedure of Diels-Alder reaction of methylideneimidazolones 1-6, 9, 12-16 with cyclopentadiene.

To a solution of methylidenehydantoin (1 eq.) in chloroform (30 ml) cyclopentadiene (8 eq.) was added and the mixture was refluxed for 6 h. The reaction was cooled to room temperature and the mixture was evaporated under vacuum. The residue was purified by column chromatography on silica gel.

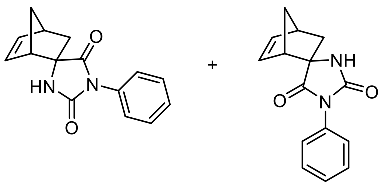

(1S,2S*,4S*)-1′-phenylspiro[bicyclo[2.2.1]heptane-2,4′-imidazolidin]-5-ene-2′,5′-dione* (**28a**) (isolated using methanol/chloroform (1:800) as eluent) and *(1S*,2R*,4S*)-1′-phenylspiro[bicyclo[2.2.1]heptane-2,4′-imidazolidin]-5-ene-2′,5′-dione* (**28b**) (isolated using methanol/chloroform (1:400) as eluent). From methylidenehydantoin **1** (184 mg, 0.98 mmol) and cyclopentadiene (201 mg, 3.04 mmol) compound **28a** (202 mg, 81%) and compound **28b** (22 mg, 9%) were obtained as a white or pale yellow crystalline solids.
The spectral data are consistent with the literature data [1].

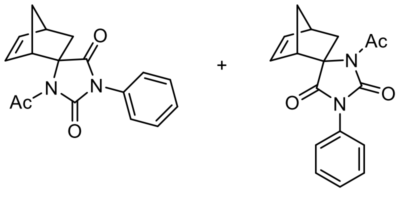

(1S,2S*,4S*)-3′-Acetyl-1′-phenylspiro[bicyclo[2.2.1]heptane-2,4′-imidazolidin]-5-ene-2′,5′-dione* (**29a**) and *(1S*,2R*,4S*)-3′-acetyl-1′-phenylspiro[bicyclo[2.2.1]heptane-2,4′-imidazolidin]-5-ene-2′,5′-dione* (**29b**) (isolated using chloroform as eluent). From methylidenehydantoin **12** (87 mg, 0.38 mmol) and cyclopentadiene (201 mg, 3.04 mmol) the mixture of compounds **29a** and **29b** in 78/22 ratio (82 mg, 73%) was obtained as a pale brown oil.

Major isomer 29a: ^{1}**H NMR** (400 MHz, $CDCl_3$): δ 7.52-7.34 (m, 5H), 6.52 (dd, J_1 = 3.1 Hz, J_2 = 5.4 Hz, 1H), 6.02 (dd, J_1 = 2.8 Hz, J_2 = 5.4 Hz, 1H), 3.22-3.11 (m, 2H), 2.92-2.86 (m, 1H), 2.52 (s, 3H), 2.34-2.27 (m, 2H), 1.40-1.35 (m, 1H). ^{13}**C NMR** (101 MHz, $CDCl_3$): δ 174.4, 170.8, 154.3, 144.0, 133.1, 131.3, 129.3 (2C), 128.9, 126.7 (2C), 73.0, 57.0, 46.9, 44.1, 33.1, 28.5.

Minor isomer 29b: ^{1}**H NMR** (400 MHz, $CDCl_3$): δ 7.52-7.34 (m, 5H), 6.53-6.48 (m, 1H), 6.05-6.01 (m, 1H), 3.22-3.11 (m, 2H), 3.01 (s, 1H), 2.76 (d, J = 8.5 Hz, 1H), 2.64 (s, 3H), 1.72-1.67 (m, 1H), 1.52 (d, J = 8.5 Hz, 1H). ^{13}**C NMR** (101 MHz, $CDCl_3$): δ 172.7, 171.2, 154.3, 141.9, 133.1, 131.2, 129.2 (2C), 128.8, 126.5 (2C), 73.2, 57.0, 46.9, 44.3, 32.5, 28.4.

HRMS (ESI+) m/z calcd. for ($C_{17}H_{17}N_2O_3$, M+H): 297,1239, found: (M+H): 297.1240.

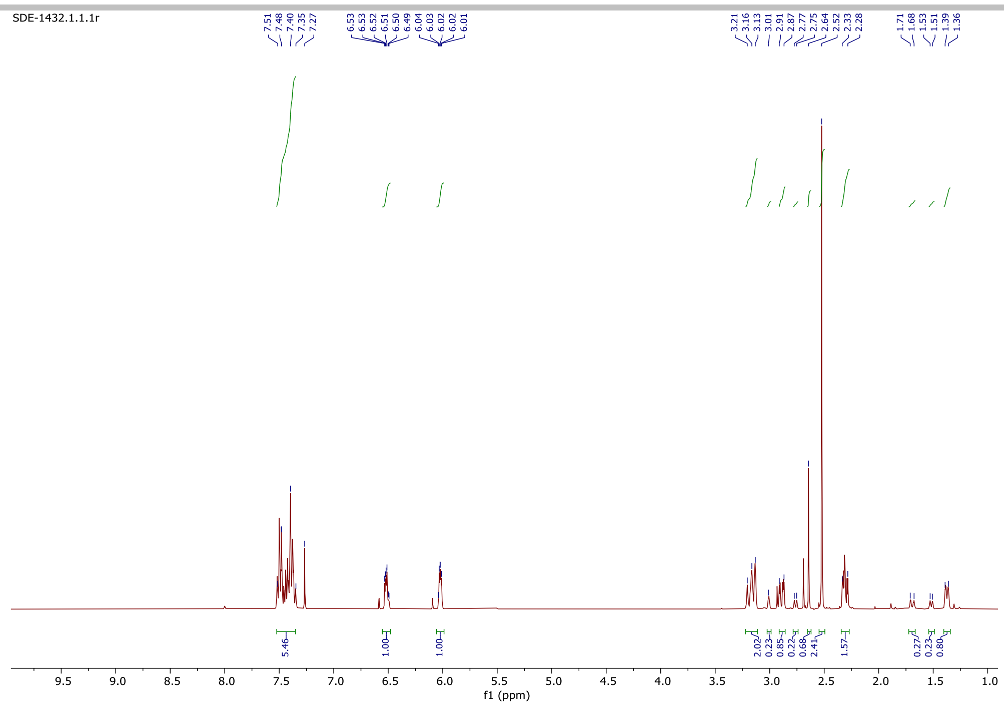

Figure S15. ^{1}H NMR spectra of the mixture of compounds **29a** and **29b.**

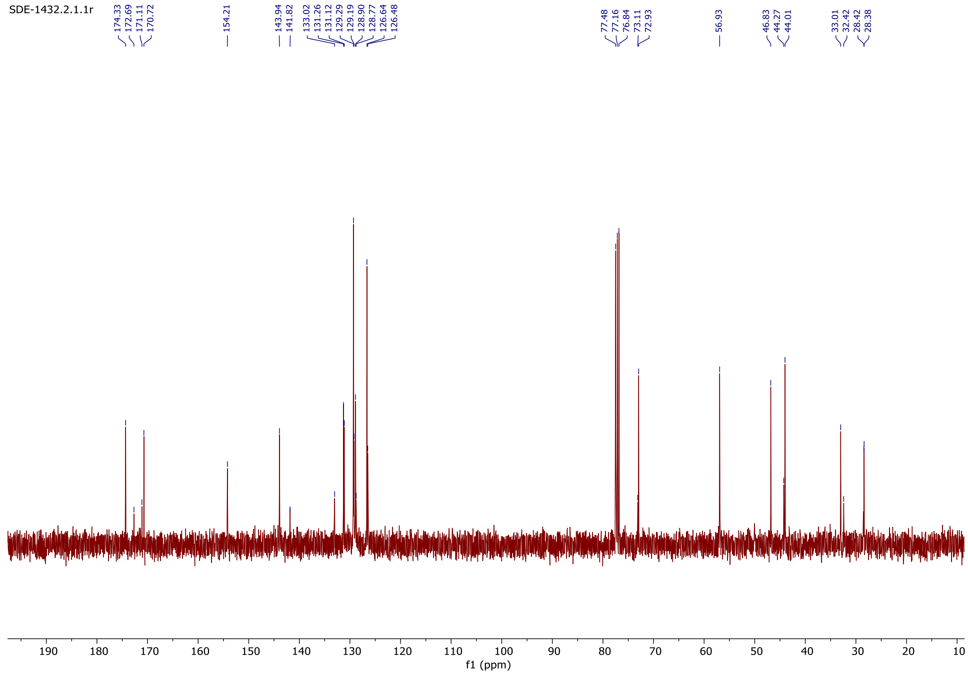

Figure S16. ^{13}C NMR spectra of the mixture of compounds **29a** and **29b.**

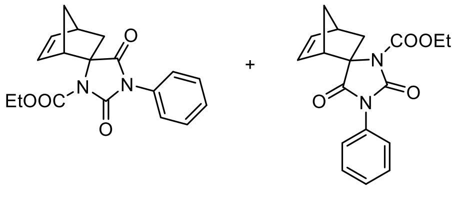

major isomer minor isomer

Ethyl (1S,2S*,4S*)-2′,5′-dioxo-1′-phenylspiro[bicyclo[2.2.1]heptane-2,4′-imidazolidin]-5-ene-3′-carboxylate* (**30a**) and *ethyl (1S*,2R*,4S*)-2′,5′-dioxo-1′-phenylspiro[bicyclo[2.2.1]heptane-2,4′-imidazolidin]-5-ene-3′-carboxylate* (**30b**) (isolated using chloroform as eluent). From methylidenehydantoin **13** (99 mg, 0.38 mmol) and cyclopentadiene (201 mg, 3.04 mmol) the mixture of compounds **30a** and **30b** in 83/17 ratio (103 mg, 83%) was obtained as a pale yellow oil.

Major isomer 30a: **^{1}H NMR** (400 MHz, $CDCl_3$): δ 7.47-7.35 (m, 5H), 6.49 (dd, J_1 = 3.1 Hz, J_2 = 5.6 Hz, 1H), 6.06 (dd, J_1 = 3.0 Hz, J_2 = 5.6 Hz, 1H), 4.38-4.18 (m, 2H), 3.16 (s, 1H), 3.13 (s, 1H), 2.66 (dd, J_1 = 3.5 Hz, J_2 = 12.4 Hz, 1H), 2.38-2.32 (m, 2H), 1.44-1.41 (m, 1H), 1.33 (t, J = 7.2 Hz, 3H). **^{13}C NMR** (101 MHz, $CDCl_3$): δ 174.0, 151.9, 151.1, 144.0, 131.2, 130.6, 129.1 (2C), 128.6, 126.5 (2C), 71.6, 63.6, 55.6, 47.3, 43.3, 35.0, 13.9.
Minor isomer 30b: **^{1}H NMR** (400 MHz, $CDCl_3$): δ 7.47-7.35 (m, 5H), 6.50-6.46 (m, 1H), 6.07-6.04 (m, 1H), 4.38-4.18 (m, 2H), 3.16 (s, 1H), 3.10-3.04 (m, 1H), 2.52 (d, J = 8.9 Hz, 1H), 2.38-2.32 (m, 1H), 1.78 (dd, J_1 = 2.4 Hz, J_2 = 12.4 Hz, 1H), 1.53 (d, J = 8.8 Hz, 1H), 1.38 (t, J = 7.1 Hz, 3H). **^{13}C NMR** (101 MHz, $CDCl_3$): δ 172.4, 151.8, 151.1, 141.4, 133.0, 130.6, 129.0 (2C), 128.5, 126.4 (2C), 72.2, 63.7, 56.0, 49.0, 43.7, 34.3, 14.1.
HRMS (ESI+) m/z calcd. for ($C_{18}H_{19}N_2O_4$, M+H): 327.1339, found: (M+H): 327.1340.

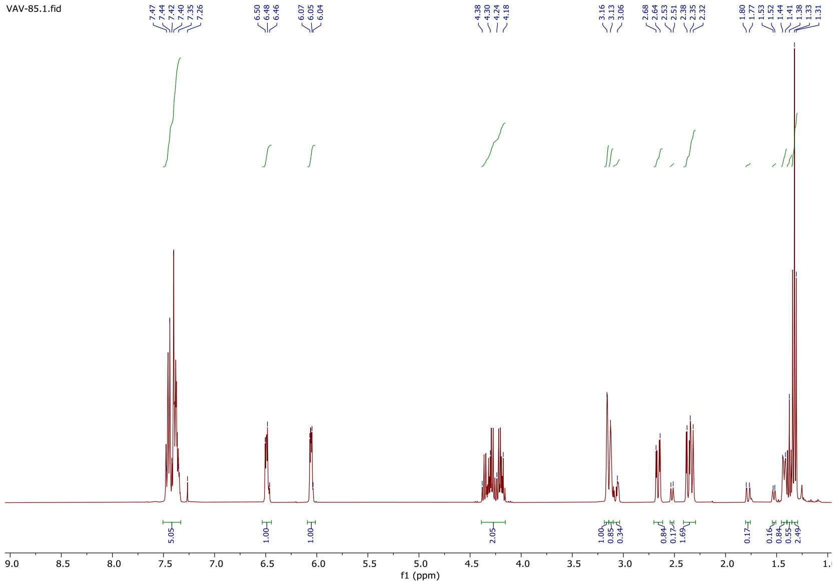

Figure S17. ^{1}H NMR spectra of the mixture of compounds **30a** and **30b.**

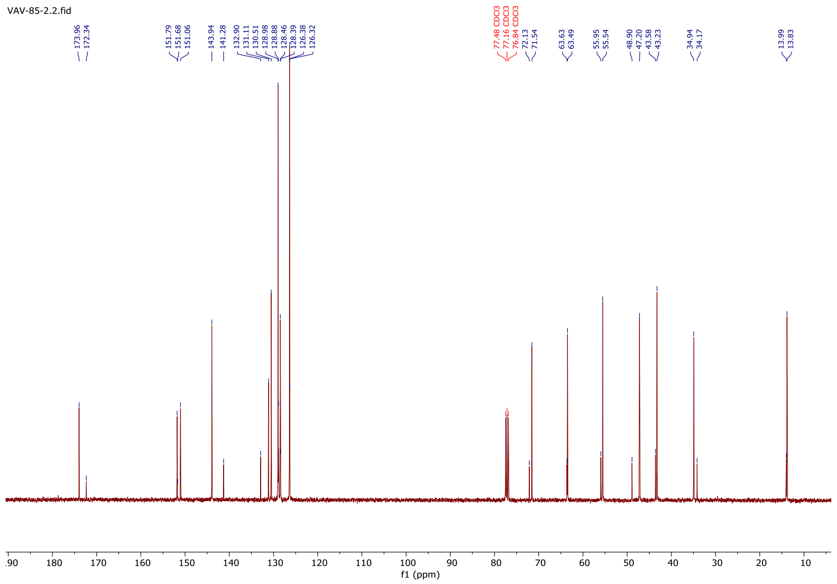

Figure S18. ^{13}C NMR spectra of the mixture of compounds **30a** and **30b.**

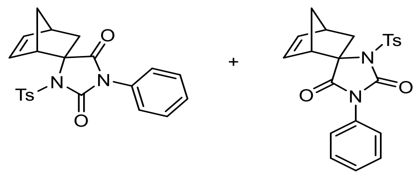

major isomer minor isomer

(1S,2S*,4S*)-1′-Phenyl-3′-tosylspiro[bicyclo[2.2.1]heptane-2,4′-imidazolidin]-5-ene-2′,5′-dione* (**31a**) and *(1S*,2R*,4S*)-1′-phenyl-3′-tosylspiro[bicyclo[2.2.1]heptane-2,4′-imidazolidin]-5-ene-2′,5′-dione* (**31b**) (isolated using chloroform as eluent). From methylidenehydantoin **14**

(130 mg, 0.38 mmol) and cyclopentadiene (201 mg, 3.04 mmol) the mixture of compounds **31a** and **31b** in 89/11 ratio (141 mg, 91%) was obtained as a pale yellow oil.

Major isomer 31a: ^{1}**H NMR** (400 MHz, $CDCl_3$): δ 7.90-7.86 (m, 2H), 7.45-7.26 (m, 7H), 6.72 (dd, J_1 = 3.0 Hz, J_2 = 5.4 Hz, 1H), 6.31 (dd, J_1 = 2.9 Hz, J_2 = 5.4 Hz, 1H), 3.21-3.16 (m, 2H), 2.70 (dd, J_1 = 3.2 Hz, J_2 = 12.4 Hz, 1H), 2.47-2.43 (m, 1H), 2.42 (s, 3H), 2.32 (d, J = 9.1 Hz, 1H), 1.49-1.45 (m, 1H). ^{13}**C NMR** (101 MHz, $CDCl_3$): δ 173.6, 152.6, 145.2, 144.3, 136.7, 132.3, 130.8, 129.5 (2C), 129.2 (2C), 128.9 (2C), 128.8, 126.3 (2C), 76.4, 56.6, 46.8, 43.6, 33.6, 21.8.

Minor isomer 31b: ^{1}**H NMR** (400 MHz, $CDCl_3$): δ 8.01-7.97 (m, 2H), 7.45-7.26 (m, 7H), 6.50 (dd, J_1 = 2.8 Hz, J_2 = 5.4 Hz, 1H), 5.99 (dd, J_1 = 2.9 Hz, J_2 = 5.3 Hz, 1H), 3.27 (s, 1H), 3.21-3.16 (m, 1H), 3.06-2.99 (m, 2H), 2.44 (s, 3H), 1.92 (dd, J_1 = 2.2 Hz, J_2 = 12.6 Hz, 1H), 1.71 (d, J = 9.2 Hz, 1H).

HRMS (ESI+) m/z calcd. for ($C_{22}H_{21}N_2O_4S$, M+H): 409.1217, found: (M+H): 409.1212.

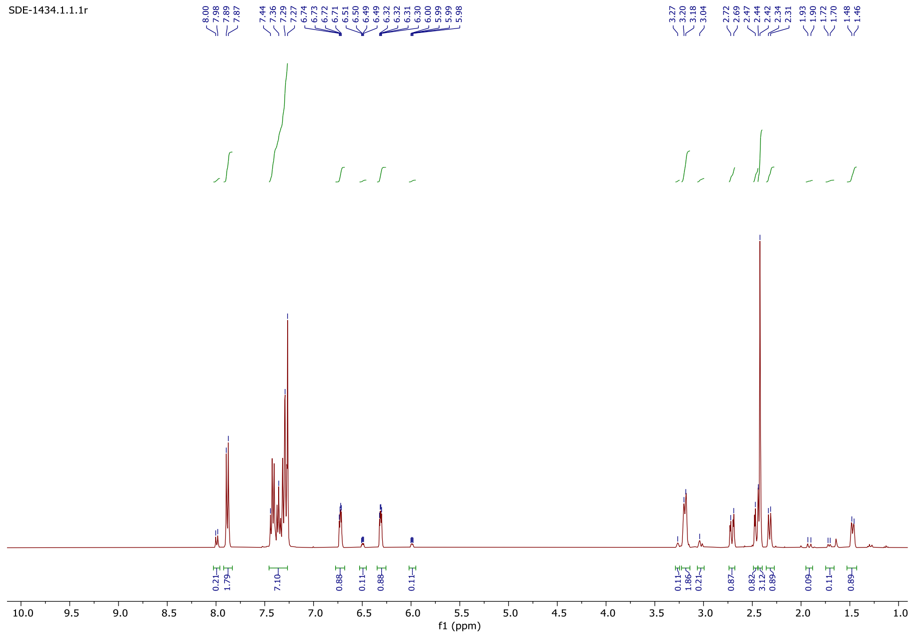

Figure S19. ^{1}H NMR spectra of the mixture of compounds **31a** and **31b.**

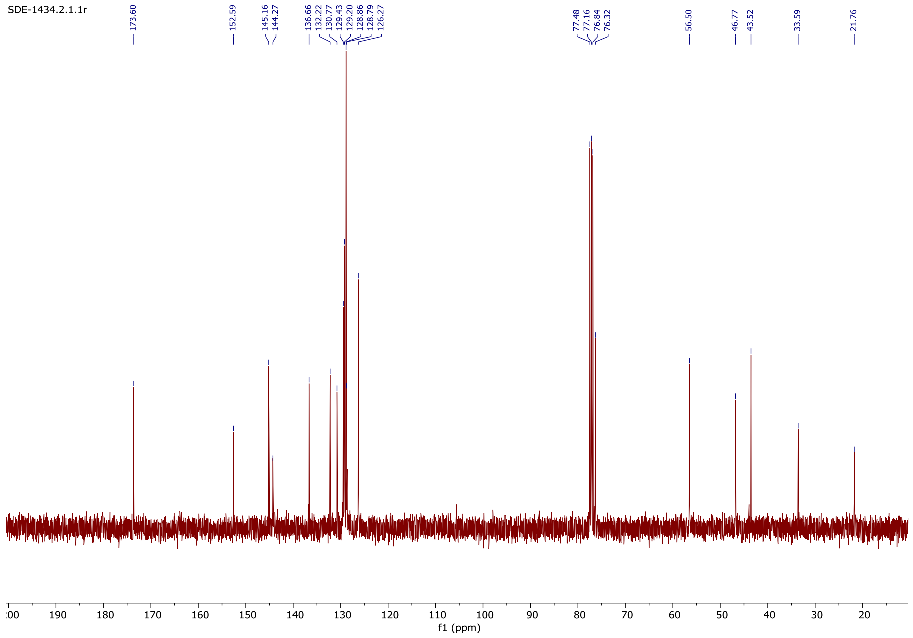

Figure S20. ^{13}C NMR spectra of the mixture of compounds **31a** and **31b.**

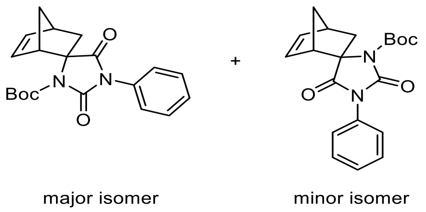

tert-Butyl (1S,2S*,4S*)-2′,5′-dioxo-1′-phenylspiro[bicyclo[2.2.1]heptane-2,4′-imidazolidin]-5-ene-3′-carboxylate* (**32a**) and *tert-butyl (1S*,2R*,4S*)-2′,5′-dioxo-1′-phenylspiro[bicyclo[2.2.1]heptane-2,4′-imidazolidin]-5-ene-3′-carboxylate* (**32b**) (isolated using chloroform as eluent). From methylidenehydantoin **15** (109 mg, 0.38 mmol) and cyclopentadiene (201 mg, 3.04 mmol) the mixture of compounds **32a** and **32b** in 85/15 ratio (98 mg, 73%) was obtained as a white crystalline solids.

Major isomer 32a: **^{1}H NMR** (400 MHz, $CDCl_3$): δ 7.48-7.36 (m, 5H), 6.50 (dd, J_1 = 3.1 Hz, J_2 = 5.4 Hz, 1H), 6.10 (dd, J_1 = 2.9 Hz, J_2 = 5.4 Hz, 1H), 3.18 (s, 1H), 3.13 (s, 1H), 2.71 (dd, J_1 = 3.4 Hz, J_2 = 12.3 Hz, 1H), 2.38-2.32 (m, 2H), 1.53 (s, 9H), 1.47-1.42 (m, 1H). **^{13}C NMR** (101 MHz, $CDCl_3$): δ 174.5, 152.2, 149.7, 144.3, 131.4, 130.9, 129.2 (2C), 128.6, 126.6 (2C), 84.4, 71.4, 55.6, 47.4, 43.4, 35.2, 27.8 (3C).

Minor isomer 32b: **^{1}H NMR** (400 MHz, $CDCl_3$): δ 7.48-7.36 (m, 5H), 6.51-6.47 (m, 1H), 6.11-6.06 (m, 1H), 3.20-3.06 (m, 3H), 2.45 (d, J = 8.8 Hz, 1H), 2.38-2.32 (m, 1H), 1.81-1.75 (m, 1H), 1.58 (s, 9H).

HRMS (ESI+) m/z calcd. for ($C_{20}H_{23}N_2O_4$, M+H): 355.1652, found: (M+H): 355.1652.

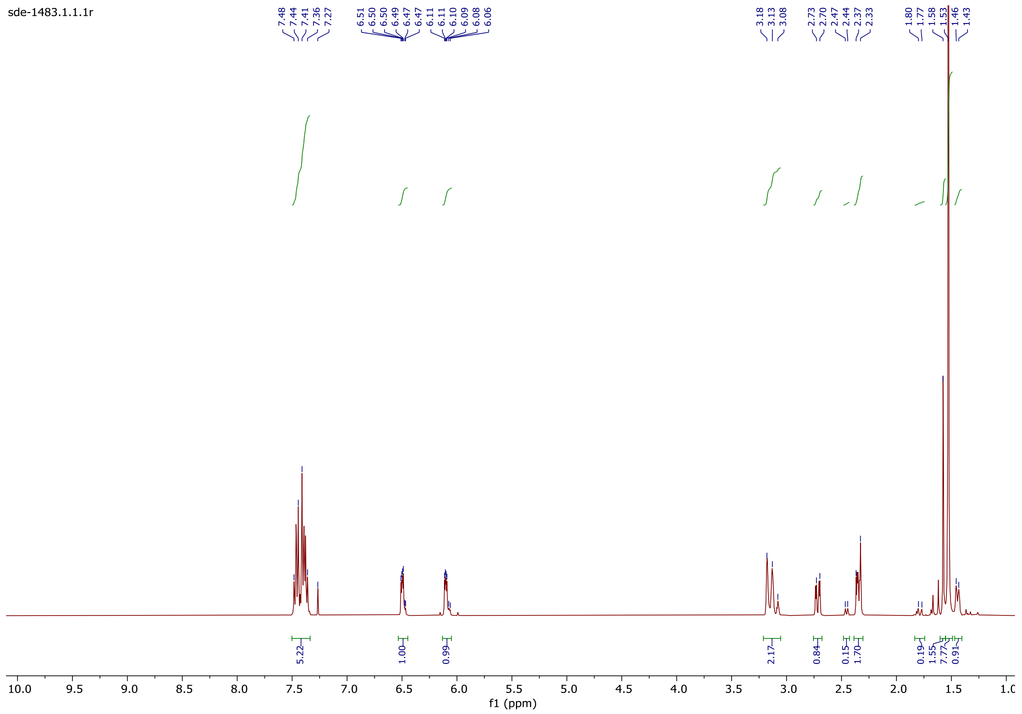

Figure S21. ^{1}H NMR spectra of the mixture of compounds **32a** and **32b.**

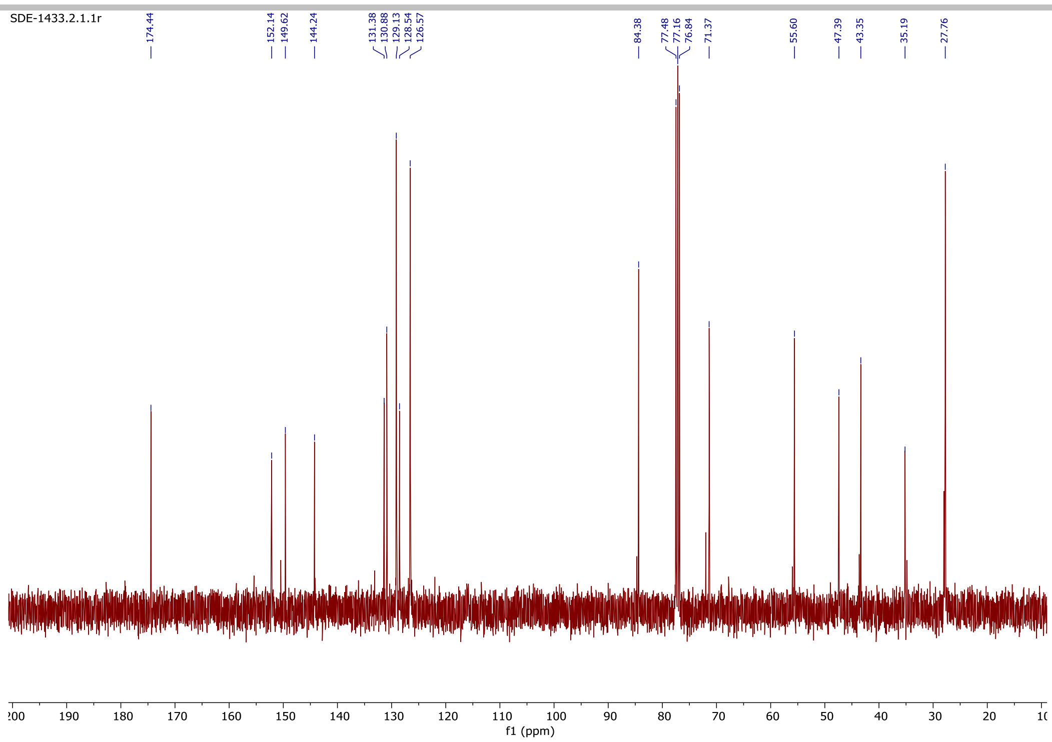

Figure S22. ^{13}C NMR spectra of the mixture of compounds **32a** and **32b.**

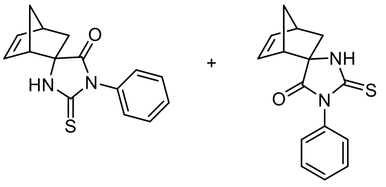

major isomer minor isomer

(1S,2S*,4S*)-1′-Phenyl-2′-thioxospiro[bicyclo[2.2.1]heptane-2,4′-imidazolidin]-5-en-5′-one* (**34a**) (isolated using chloroform as eluent) and *(1S*,2R*,4S*)-1′-phenyl-2′-thioxospiro[bicyclo[2.2.1]heptane-2,4′-imidazolidin]-5-en-5′-one* (**34b**) (isolated using methanol/chloroform (1:400) as eluent). From methylidenethiohydantoin **2** (200 mg, 0.98 mmol) and cyclopentadiene (201 mg, 3.04 mmol) compound **34a** (209 mg, 79%) and compound **34b** (22 mg, 8%) were obtained as a white or pale yellow crystalline solids.

The spectral data are consistent with the literature data [1].

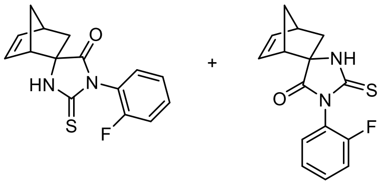

major isomer minor isomer

(1S,2S*,4S*)-1′-(2-Fluorophenyl)-2′-thioxospiro[bicyclo[2.2.1]heptane-2,4′-imidazolidin]-5-en-5′-one* (**35a**) (isolated using chloroform as eluent) and *(1S*,2R*,4S*)-1′-(2-fluorophenyl)-2′-thioxospiro[bicyclo[2.2.1]heptane-2,4′-imidazolidin]-5-en-5′-one* (**35b**) (isolated using methanol/chloroform (1:400) as eluent). From methylidenethiohydantoin **3** (218 mg, 0.98 mmol) and cyclopentadiene (201 mg, 3.04 mmol) compound **35a** (201 mg, 71%) and compound **35b** (20 mg, 7%) were obtained as a white or pale yellow crystalline solids.

Major isomer 35a: ^{1}**H NMR** (400 MHz, $CDCl_3$): δ 7.49-7.47 (m, 1H), 7.39-7.35 (m, 1H), 7.31-7.25 (m, 3H), 6.65-6.62 (m, 1H), 6.33-6.27 (m, 1H), 3.23-3.19 (m, 1H), 3.12 (s, 1H), 2.51-2.45 (s, 1H), 2.28-2.26 (m, 1H), 1.61-1.59 (m, 1H), 1.54-1.48 (m, 1H). ^{13}**C NMR** (101 MHz, $CDCl_3$): (two sets of signals for C(Ar)-N – rotamers) δ 180.9 (s, C+C′), 175.9 (s), 175.8 (s), 157.6 (d, J = 244.7 Hz, C+C′), 142.5 (s), 142.4 (s), 132.9 (d, J = 18.1 Hz, C+C′), 131.1 (d, J = 7.7 Hz), 131.0 (d, J = 7.9 Hz), 130.5 (s), 130.4 (s), 124.2 (d, J = 4.0 Hz), 124.1 (d, J = 3.9 Hz), 120.4 (d, J = 12.9 Hz, C+C′), 116.3 (d, J = 19.5 Hz), 116.2 (d, J = 19.5 Hz), 69.6 (s), 69.5 (s), 52.6 (s), 52.2 (s), 46.9 (s), 46.8 (s), 42.2 (s), 42.1 (s), 40.6 (s), 40.2 (s). **HRMS** (ESI+) m/z calcd. for ($C_{15}H_{14}FN_2OS$, M+H): 289.0805, found: (M+H): 289.0801.

Minor isomer 35b: **^{1}H NMR** (400 MHz, $CDCl_3$): 8.68 (bs, 1H), 7.53-7.42 (m, 1H), 7.42-7.33 (m, 1H), 7.33-7.19 (m, 2H), 6.45 (dd, J_1 = 3.0 Hz, J_2 = 5.7 Hz, 1H), 6.17 (dd, J_1 = 3.2 Hz, J_2 = 5.8 Hz, 1H), 3.17-3.12 (m, 1H), 3.12-3.07 (m, 1H), 2.11-2.04 (m, 1H), 1.93 (td, J_1 = 2.7 Hz, J_2 = 12.2 Hz, 1H), 1.86–1.69 (m, 2H). **^{13}C NMR** (101 MHz, $CDCl_3$): (two sets of signals for C(Ar)-N – rotamers) δ 181.2 (s), 181.1 (s), 174.6 (s), 174.5 (s), 158.0 (d, J = 253.4 Hz), 157.9 (d, J = 251.2 Hz), 140.6 (s), 140.5 (s), 131.6 (s), 131.5 (s), 131.4 (d, J = 7.9 Hz), 131.3 (d, J = 7.9 Hz), 130.9 (s), 130.8 (s), 124.5 (d, J = 3.6 Hz), 124.4 (d, J = 3.6 Hz), 120.9 (d, J = 13.0 Hz), 120.8 (d, J = 13.0 Hz), 116.7 (d, J = 19.4 Hz), 116.6 (d, J = 19.4 Hz), 69.6 (s), 69.5 (s), 54.2 (s), 53.7 (s), 49.5 (s), 49.4 (s), 43.0 (s), 42.9 (s), 41.5 (s), 40.9 (s). **^{19}F NMR** (376 MHz, $CDCl_3$): δ -119.05 (ddd, J_1 = 4.9 Hz, J_2 = 7.2 Hz, J_3 = 9.7 Hz), -119.56 (ddd, J_1 = 5.1 Hz, J_2 = 7.2 Hz, J_3 = 9.07 Hz). **HRMS** (ESI+) m/z calcd. for ($C_{15}H_{14}FN_2OS$, M+H): 289.0805, found: (M+H): 289.0808.

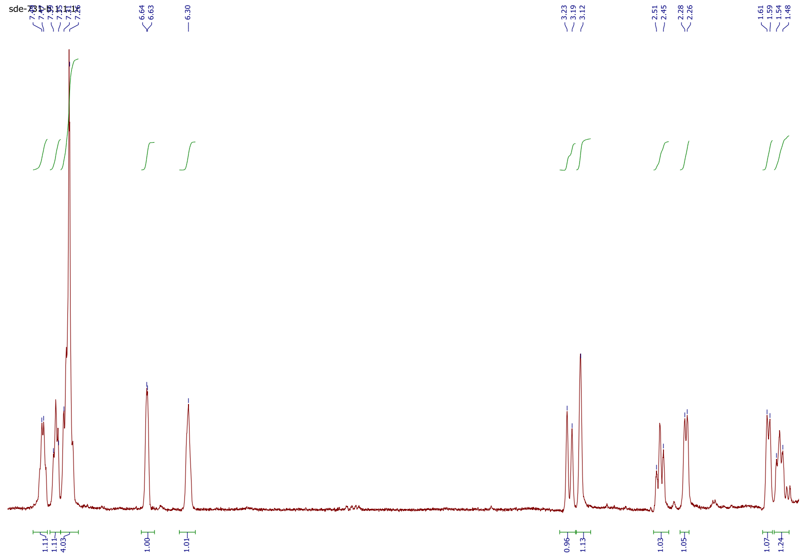

Figure S23. ^{1}H NMR spectra of compound **35a.**

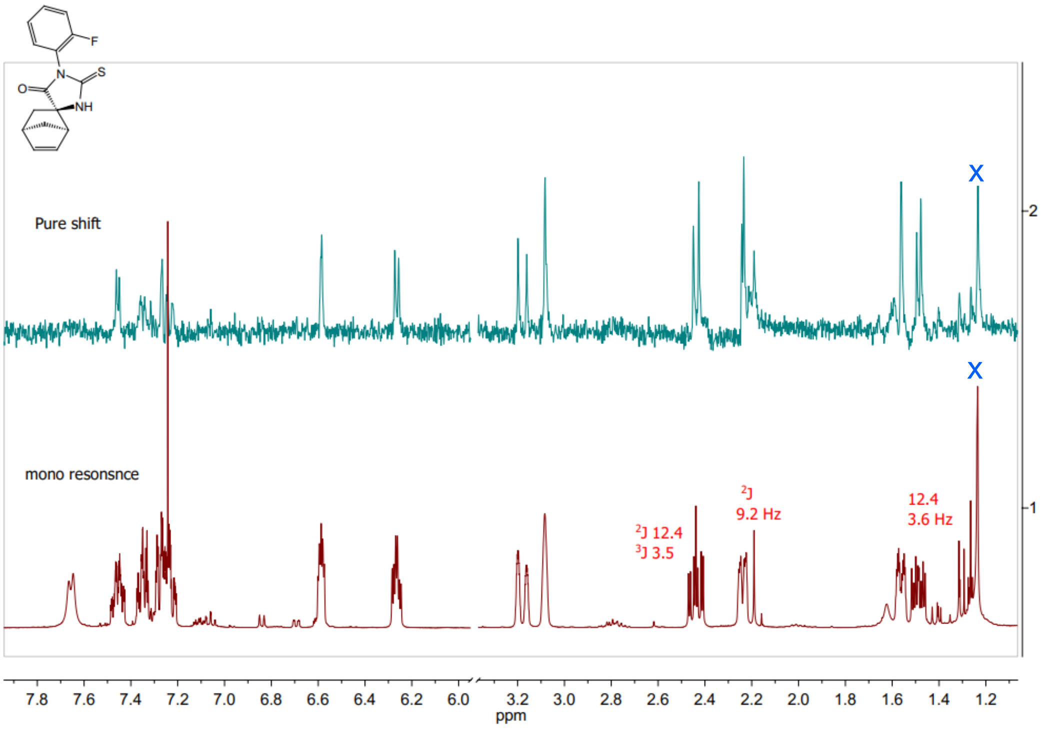

Figure S24. ^{1}H NMR spectra (pureshift) of compound **35a** (*the sign "x" marks the residual signals of tar*).

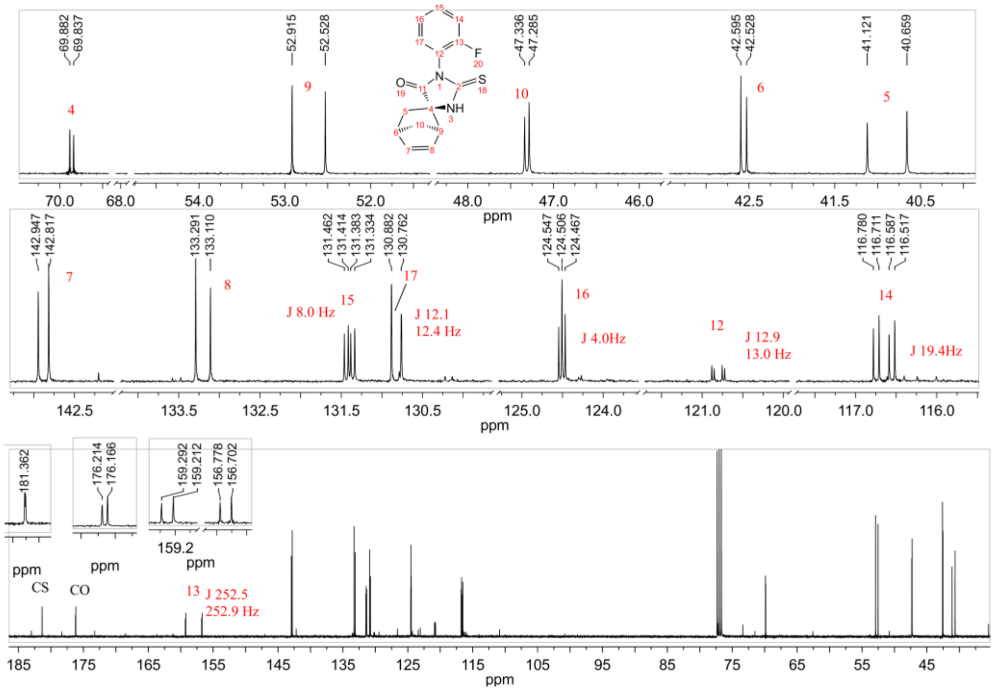

Figure S25. ^{13}C NMR spectra of compound **35a.**

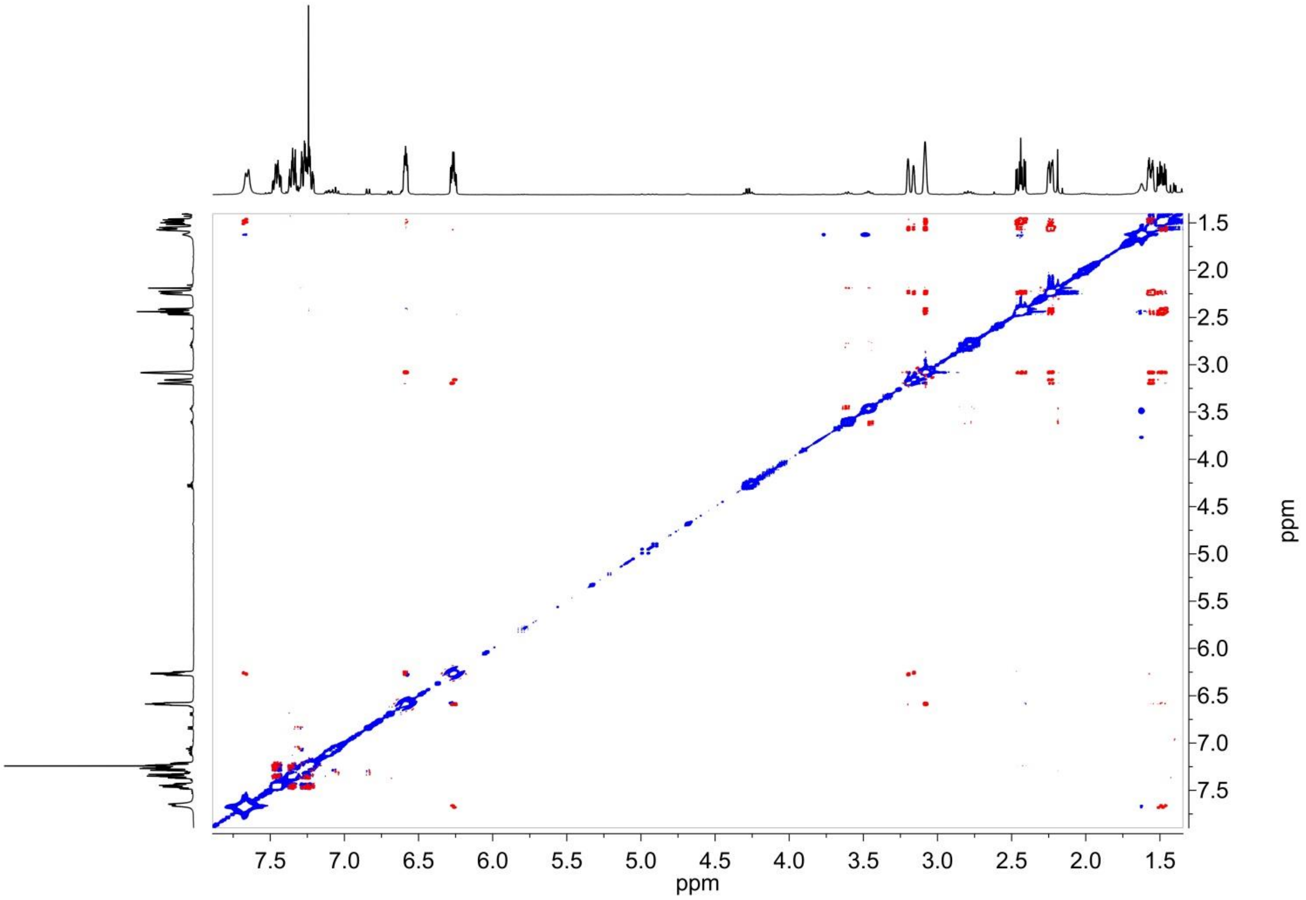

Figure S26. NOESY ^1H-^1H NMR spectra of compound **35a.**

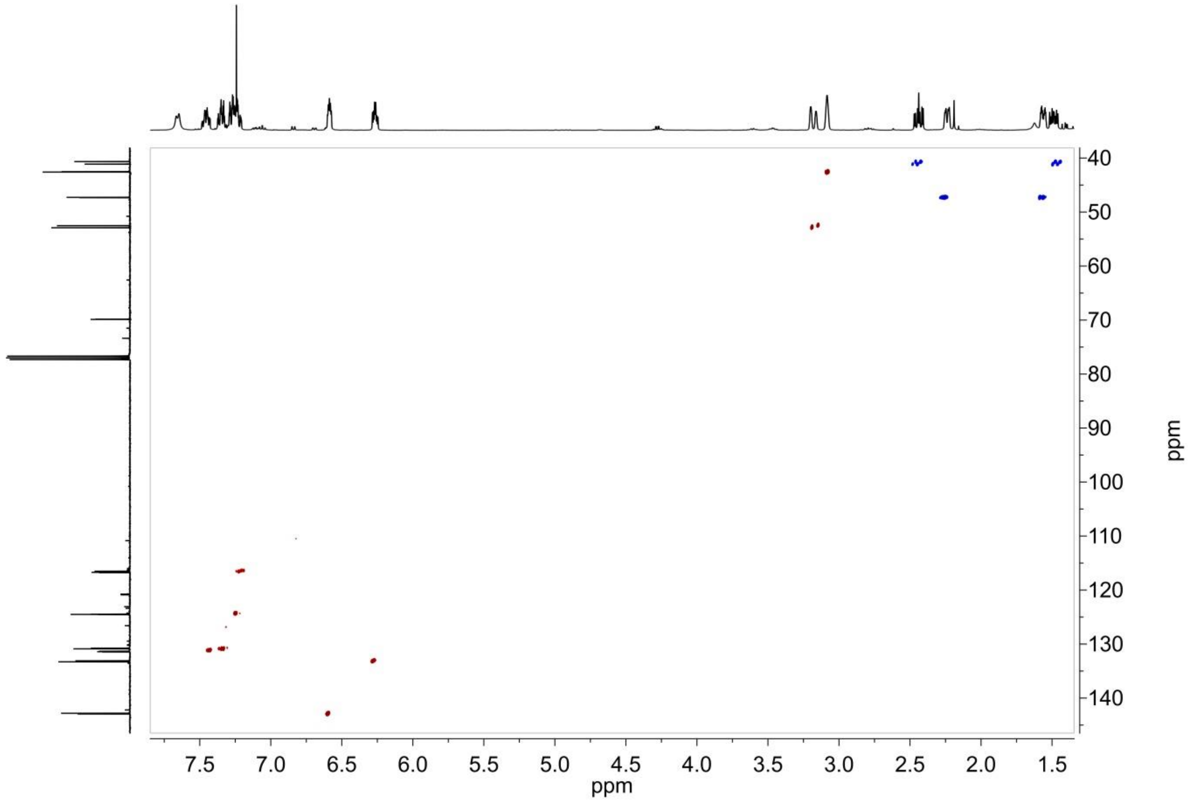

Figure S27. HSQC ^{1}H-^{13}C NMR spectra of compound **35a.**

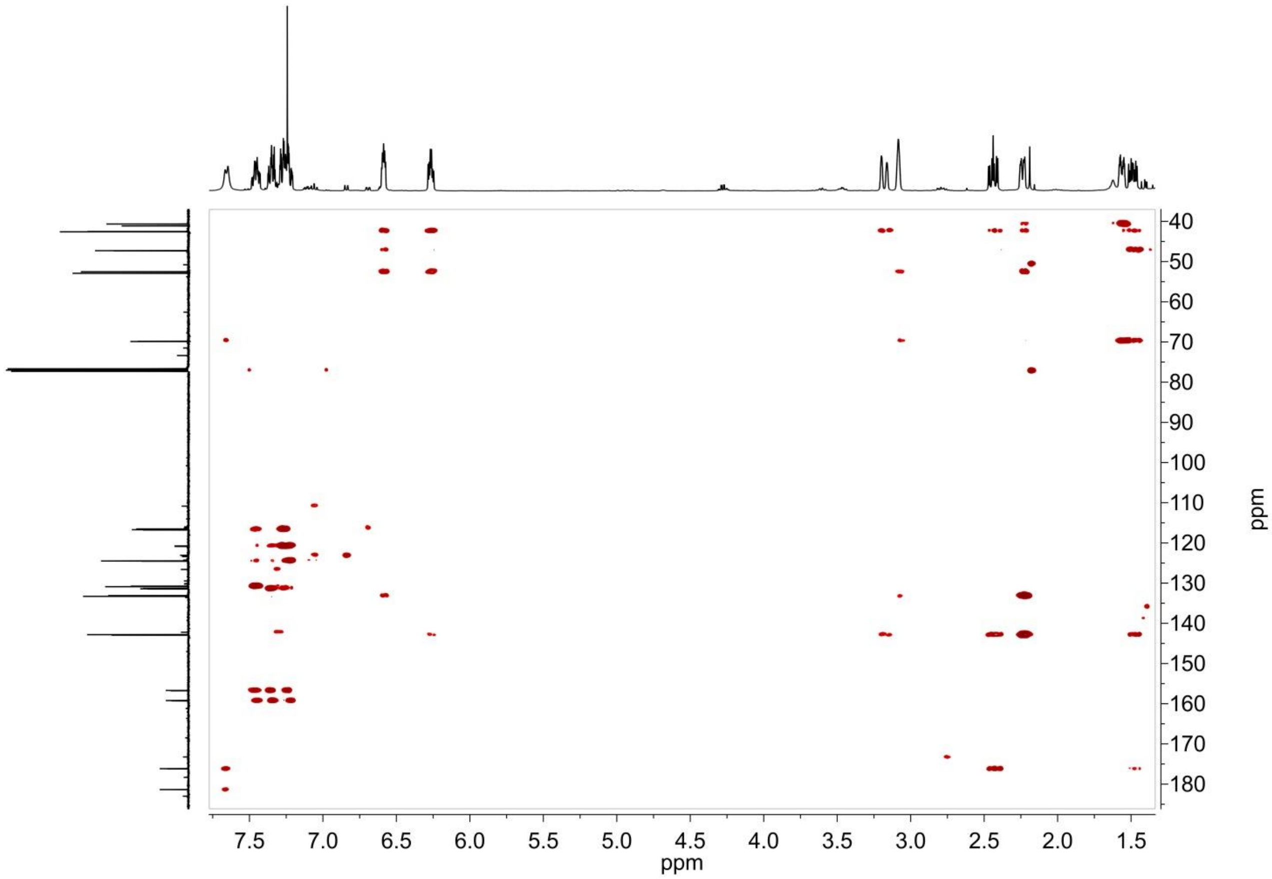

Figure S28. HMBC ^{1}H-^{13}C NMR spectra of of compound **35a.**

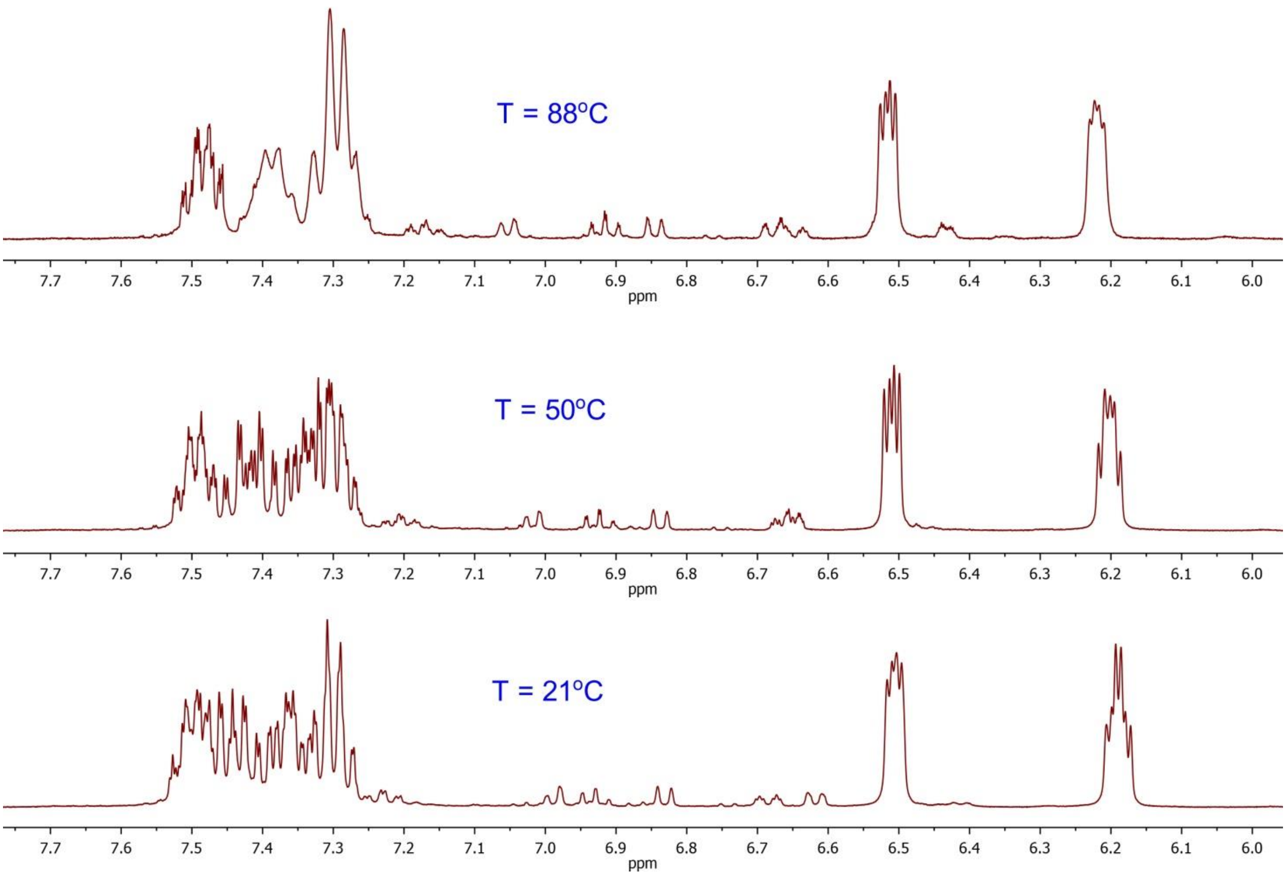

Figure S29. ^{1}H NMR spectra of compound **35a** at various temperatures (DMSO-d6).

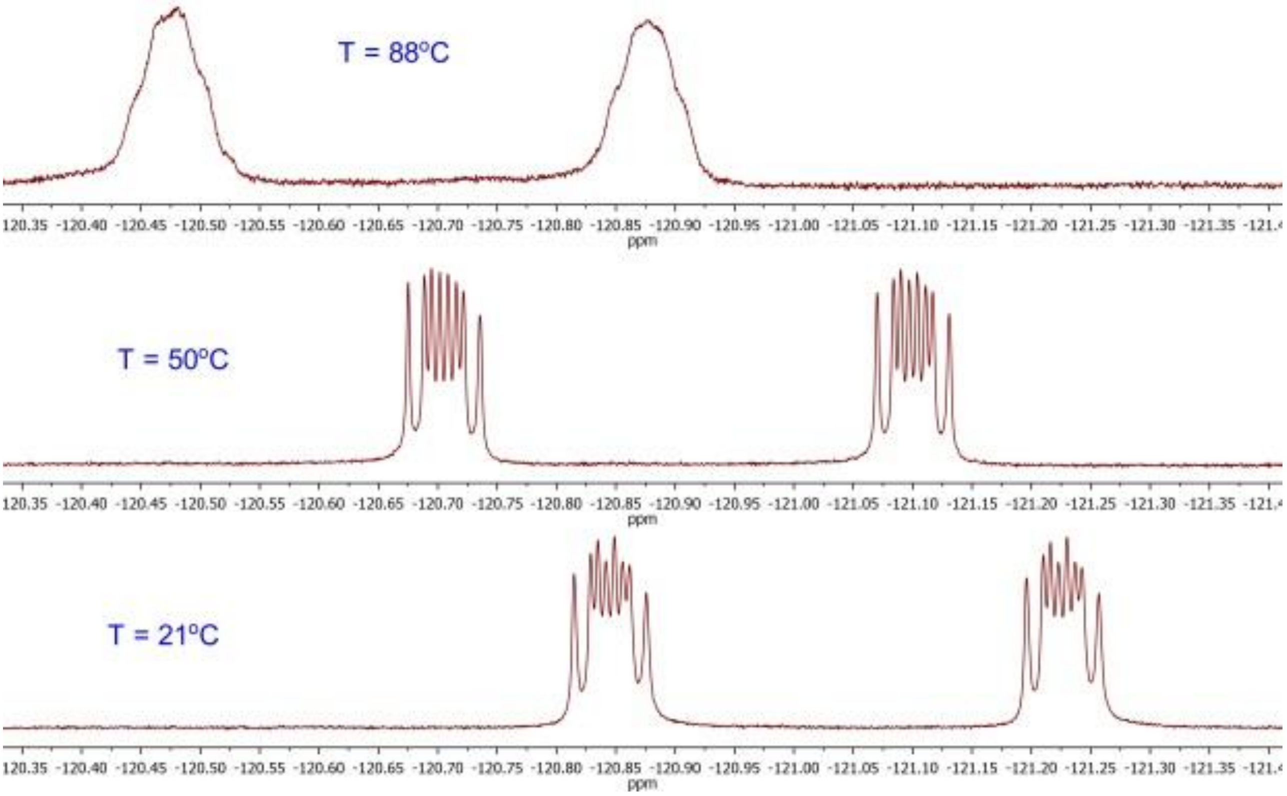

Figure S30. ^{19}F NMR spectra of compound **35a** at various temperatures (DMSO-d6).

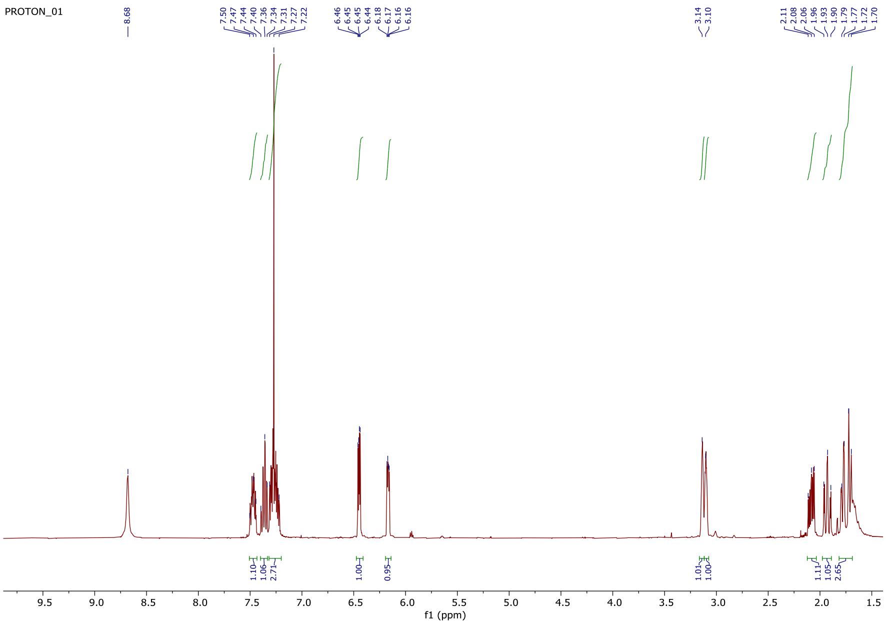

Figure S31. ^{1}H NMR spectra of compound **35b.**

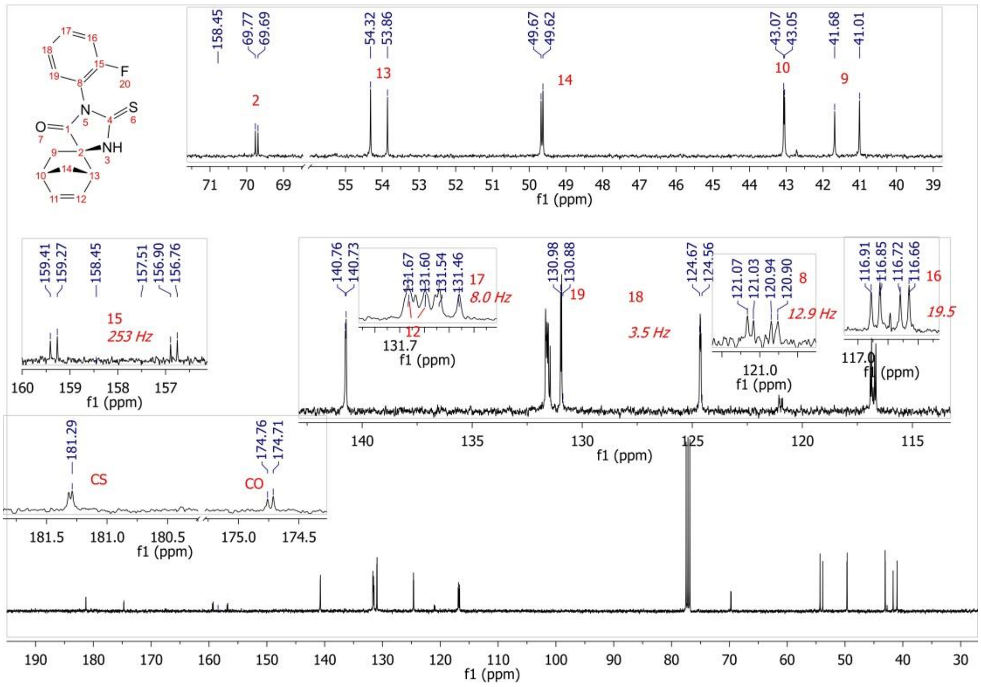

Figure S32. ^{13}C NMR spectra of compound **35b.**

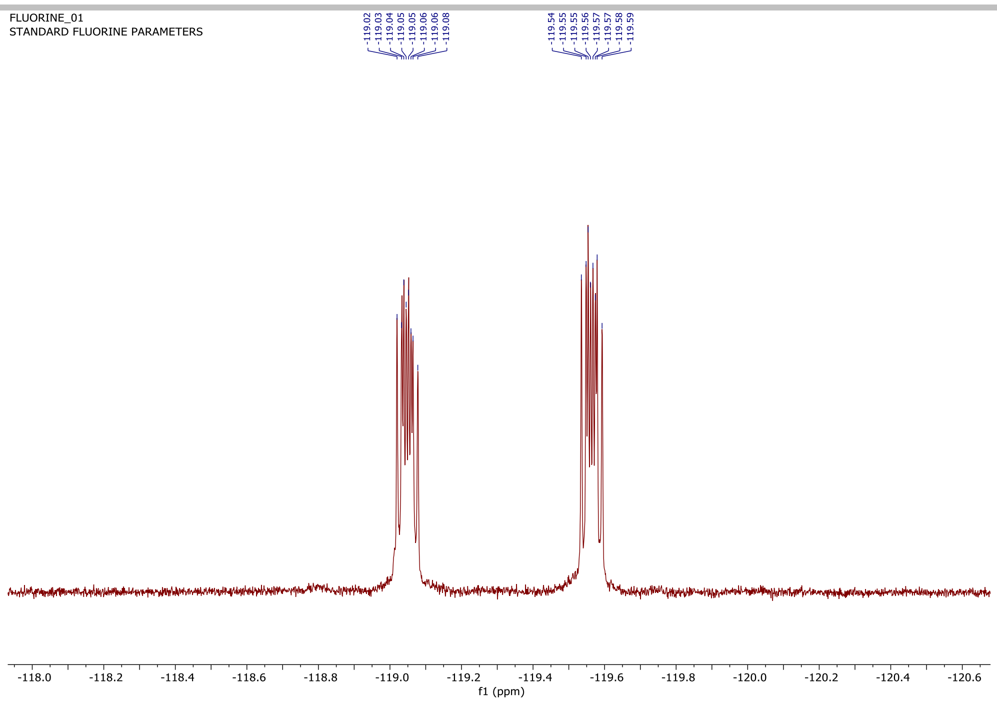

Figure S33. ^{19}F NMR spectra of compound **35b.**

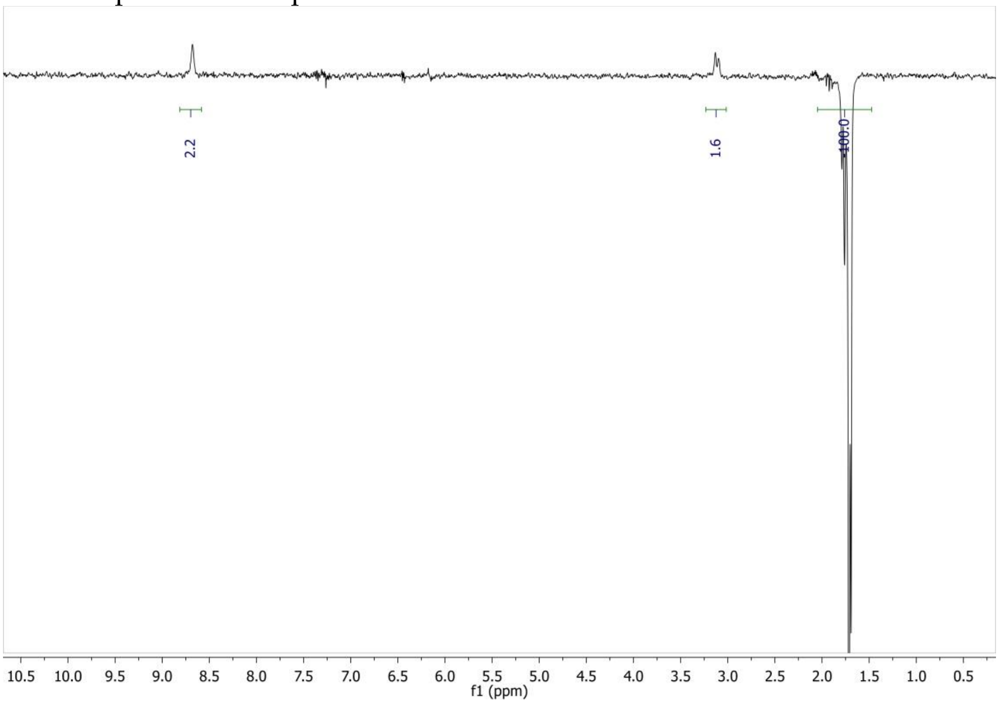

Figure S34. ^{1}H NOESY1D NMR spectra of compound **35b.**

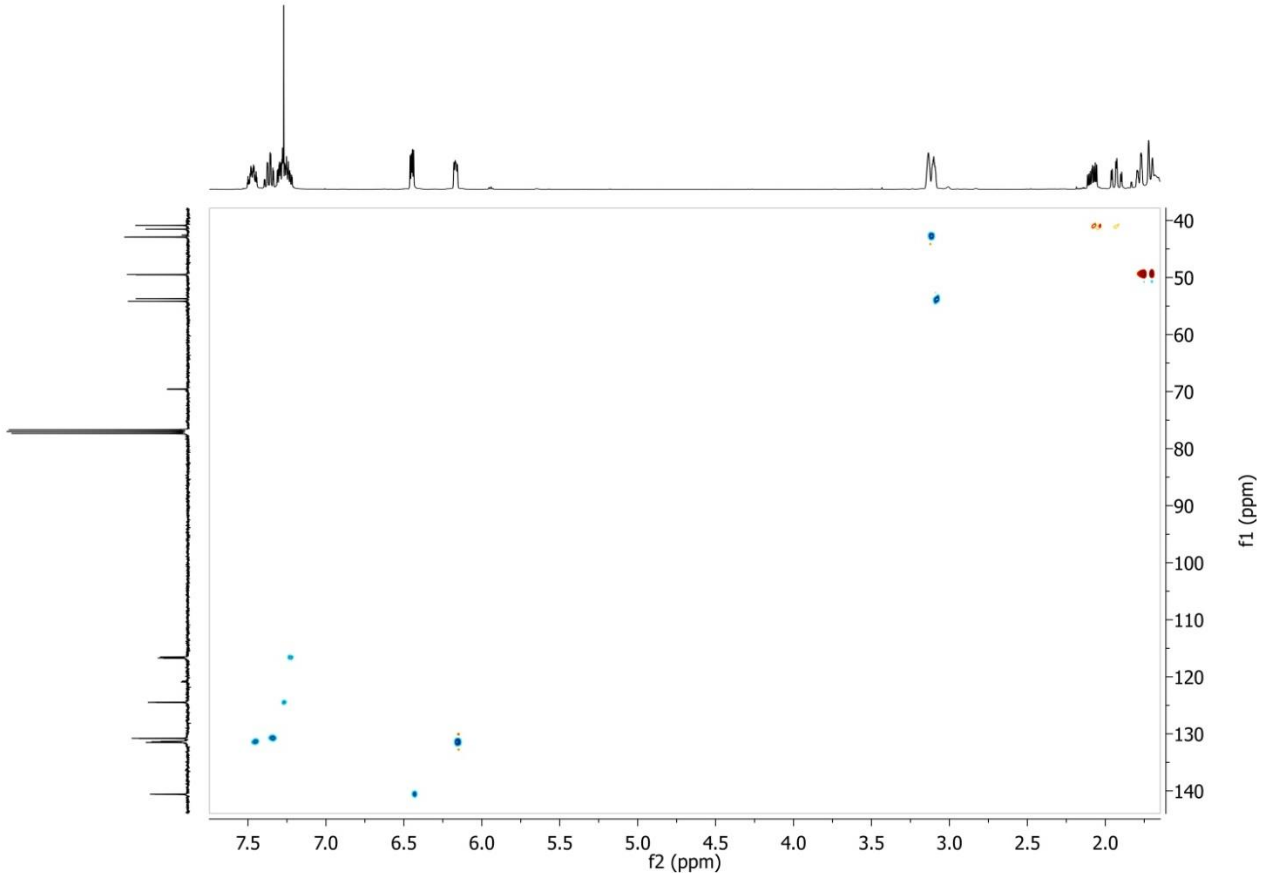

Figure S35. HSQC ^1H-^{13}C NMR spectra of compound **35b.**

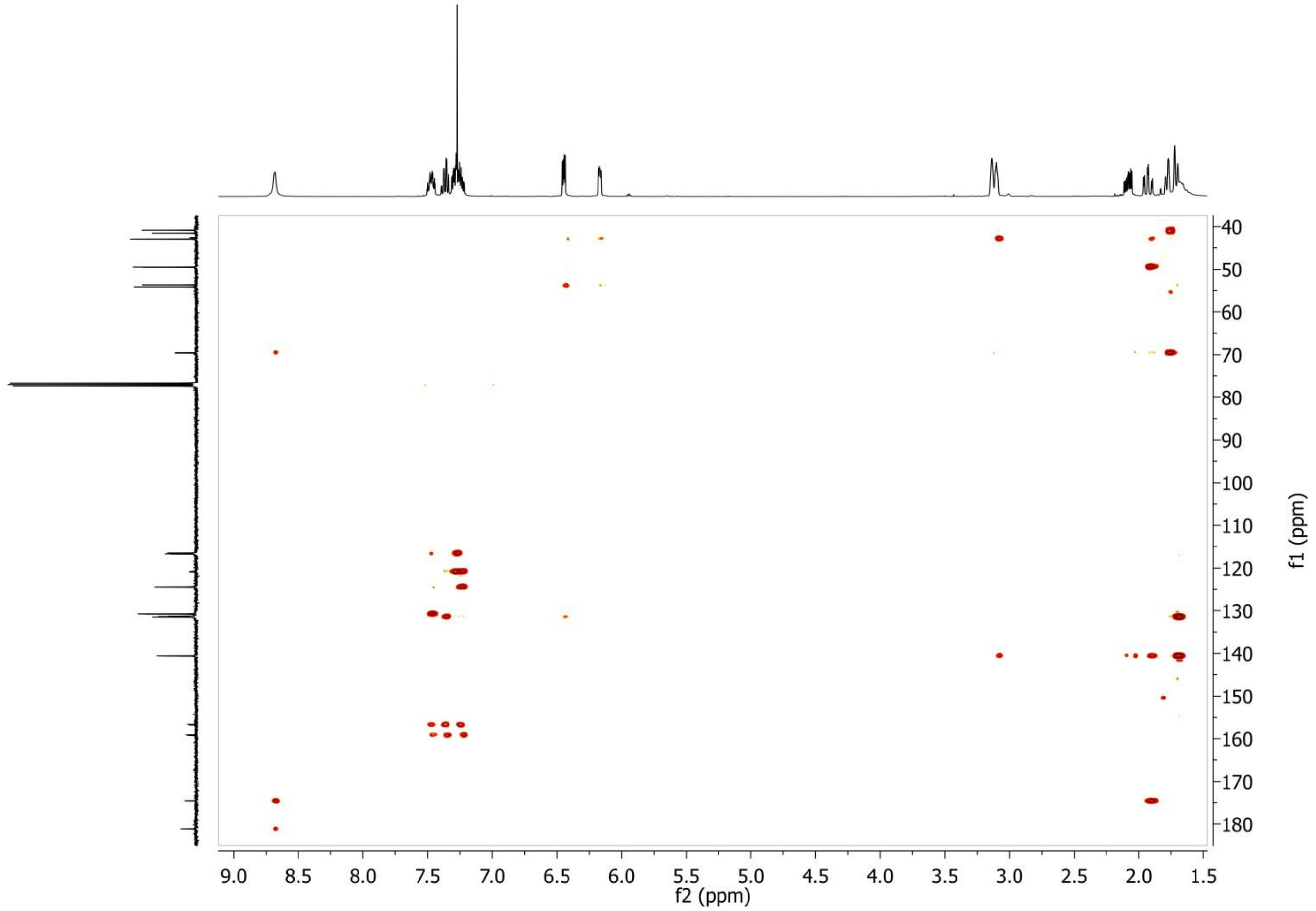

Figure S36. HMBC ^{1}H-^{13}C NMR spectra of of compound **35b.**

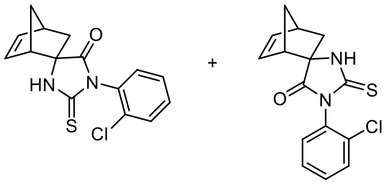

major isomer minor isomer

(1S,2S*,4S*)-1′-(2-Chlorophenyl)-2′-thioxospiro[bicyclo[2.2.1]heptane-2,4′-imidazolidin]-5-en-5′-one* (**36a**) (isolated using chloroform as eluent) and *(1S*,2R*,4S*)-1′-(2-chlorophenyl)-2′-thioxospiro[bicyclo[2.2.1]heptane-2,4′-imidazolidin]-5-en-5′-one* (**36b**) (isolated using methanol/chloroform (1:400) as eluent). From methylidenethiohydantoin **4** (234 mg, 0.98 mmol) and cyclopentadiene (201 mg, 3.04 mmol) compound **36a** (203 mg, 68%) was obtained as a white crystalline solid. A minor product can be detected in the NMR spectra of the reaction mixture, but it was not possible to isolate it preparatively.

Major isomer 36a: 7.79 (bs, 1H), 7.59-7.56 (m, 1H), 7.48-7.35 (m, 3H), 6.61-6.59 (m, 1H), 6.31-6.27 (m, 1H), 3.25-3.20 (m, 1H), 3.10 (s, 1H), 2.50-2.43 (s, 1H), 2.29-2.25 (m, 1H), 1.60-1.49 (m, 2H). **^{13}C NMR** (101 MHz, $CDCl_3$): (two sets of signals of C(Ar)-N – rotamers) δ 181.3 (C+C′), 176.4, 176.2, 143.0, 142.9, 133.5, 133.5, 133.4, 133.4, 131.4 (C+C′), 131.3 (C+C′), 131.2 (C+C′), 130.5, 130.4, 127.8 (C+C′), 70.2, 70.1, 53.2, 52.4, 47.5, 47.4, 42.7, 42.6, 41.4, 40.5. **HRMS** (ESI+) m/z calcd. for ($C_{15}H_{14}ClN_2OS$, M+H): 305.0510, found: (M+H): 305.0506.

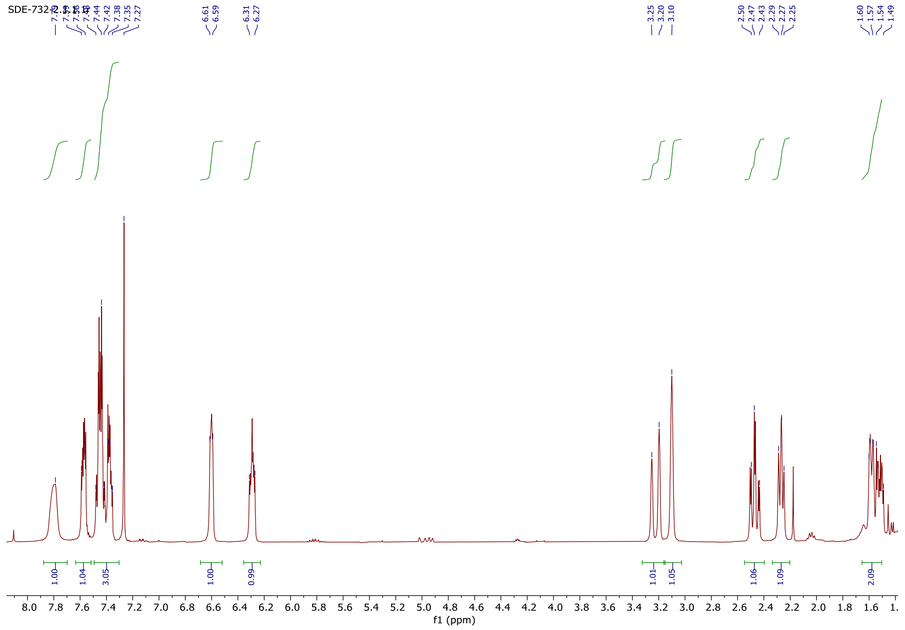

Figure S37. ^{1}H NMR spectra of compound **36a.**

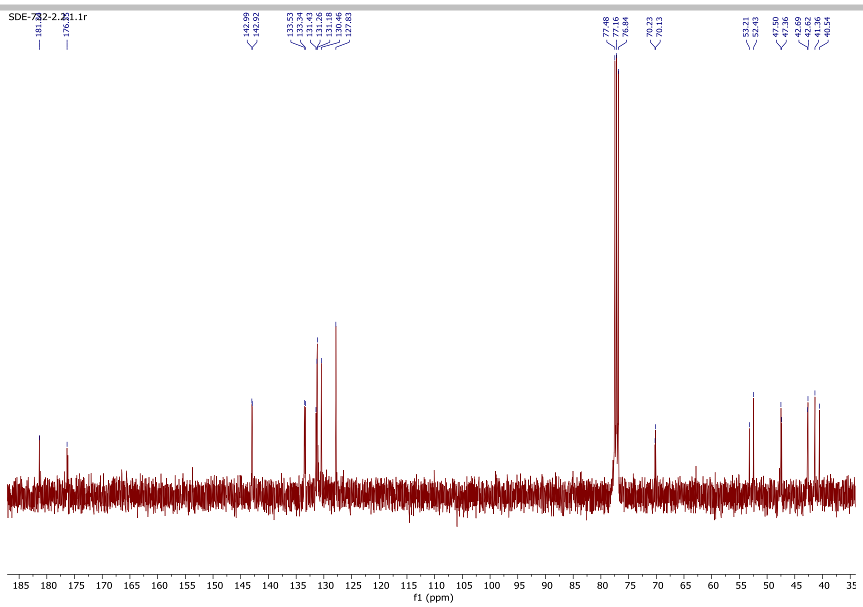

Figure S38. ^{13}C NMR spectra of compound **36a.**

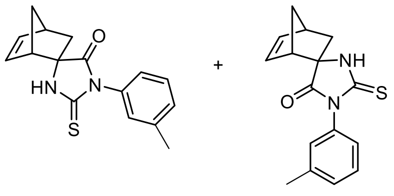

major isomer minor isomer

(1S,2S*,4S*)-2′-Thioxo-1′-(m-tolyl)spiro[bicyclo[2.2.1]heptane-2,4′-imidazolidin]-5-en-5′-one* (**37a**) (isolated using chloroform as eluent) and *(1S*,2R*,4S*)-2′-thioxo-1′-(m-tolyl)spiro[bicyclo[2.2.1]heptane-2,4′-imidazolidin]-5-en-5′-one* (**37b**) (isolated using methanol/chloroform (1:400) as eluent). From methylidenethiohydantoin **5** (214 mg, 0.98 mmol) and cyclopentadiene (201 mg, 3.04 mmol) compound **37a** (223 mg, 80%) and compound **37b** (22 mg, 8%) were obtained as a white or pale yellow crystalline solids.

Major isomer 37a: ^{1}**H NMR** (400 MHz, $CDCl_3$): δ 7.75 (bs, 1H), 7.42-7.38 (m, 1H), 7.28-7.26 (m, 1H), 7.16-7.14 (m, 2H), 6.61 (dd, J_1 = 3.1 Hz, J_2 = 5.6 Hz, 1H), 6.28 (dd, J_1 = 2.9 Hz, J_2 = 5.6 Hz, 1H), 3.17 (s, 1H), 3.10 (s, 1H), 2.46-2.42 (m, 1H), 2.42 (s, 3H), 2.27 (d, J = 9.2 Hz, 1H), 1.60-1.56 (m, 1H), 1.50 (dd, J_1 = 3.6 Hz, J_2 = 12.4 Hz, 1H). ^{13}**C NMR** (101 MHz, $CDCl_3$): δ 181.6, 176.6, 142.4, 138.8, 133.0, 132.4, 129.7, 128.5, 128.4, 125.0, 69.2, 52.4, 46.9, 42.2, 40.5, 21.0. **HRMS** (ESI+) m/z calcd. for ($C_{16}H_{17}N_2OS$, M+H): 285.1056, found: (M+H): 285.1057.

Minor isomer 37b: ^{1}**H NMR** (400 MHz, $CDCl_3$): δ 8.84 (bs, 1H), 7.42-7.38 (m, 1H), 7.28-7.26 (m, 1H), 7.15-7.09 (m, 2H), 6.43 (dd, J_1 = 3.0 Hz, J_2 = 5.6 Hz, 1H), 6.16 (dd, J_1 = 3.1 Hz, J_2 = 5.7 Hz, 1H), 3.11 (s, 1H), 3.06 (s, 1H), 2.41 (s, 3H), 2.07 (dd, J_1 = 3.6 Hz, J_2 = 12.6 Hz, 1H), 1.90 (dd, J_1 = 2.5 Hz, J_2 = 12.6 Hz, 1H), 1.80-1.69 (m, 2H). ^{13}**C NMR** (101 MHz, $CDCl_3$): δ 181.4, 175.0, 140.1, 138.7, 132.4, 131.1, 129.6, 128.5, 128.4, 125.0, 68.9, 53.5, 49.1, 42.5, 40.9, 20.9. **HRMS** (ESI+) m/z calcd. for ($C_{16}H_{17}N_2OS$, M+H): 285.1056, found: (M+H): 285.1055.

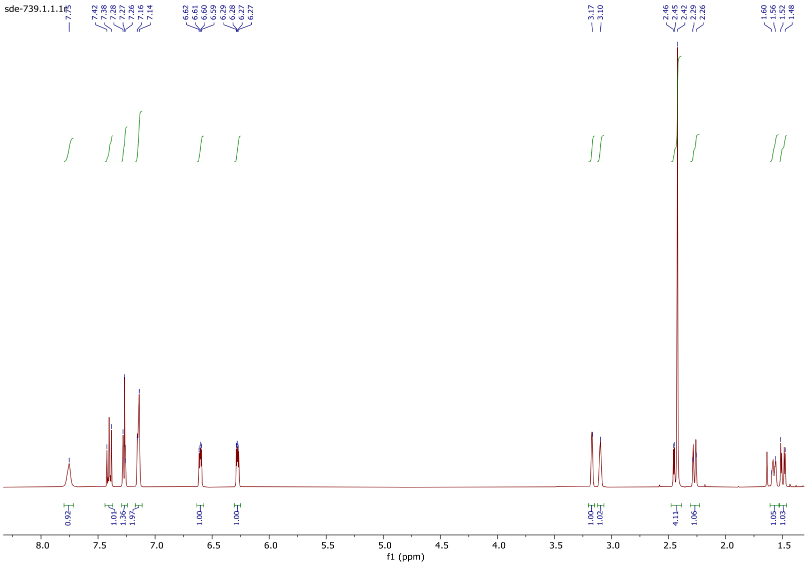

Figure S39. ^{1}H NMR spectra of compound **37a.**

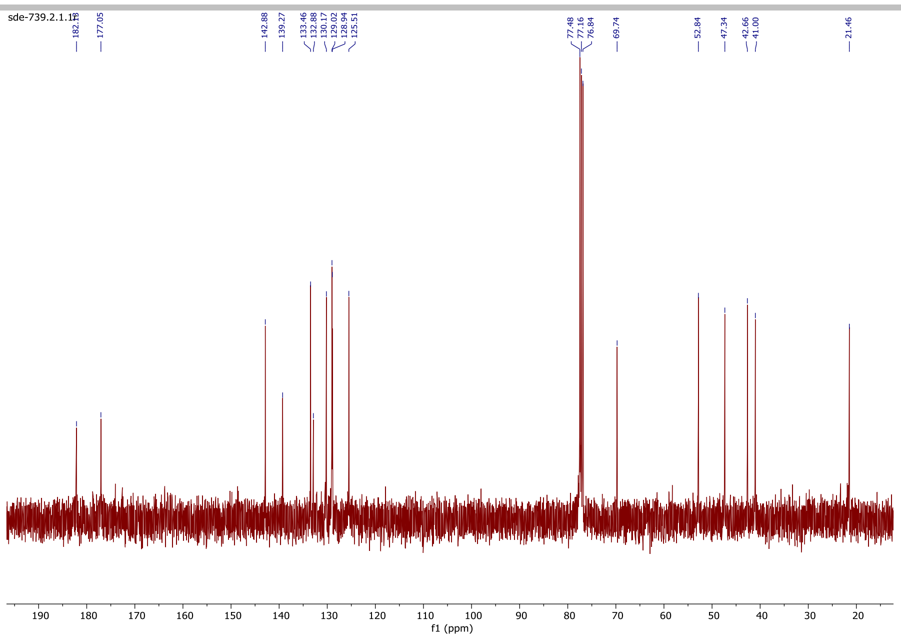

Figure S40. ^{13}C NMR spectra of compound **37a.**

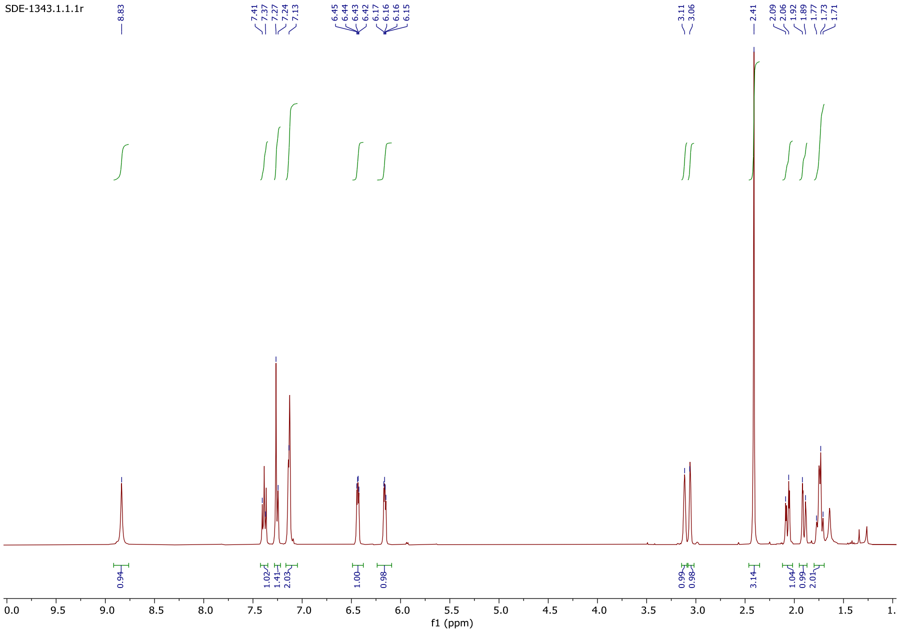

Figure S41. ^{1}H NMR spectra of compound **37b.**

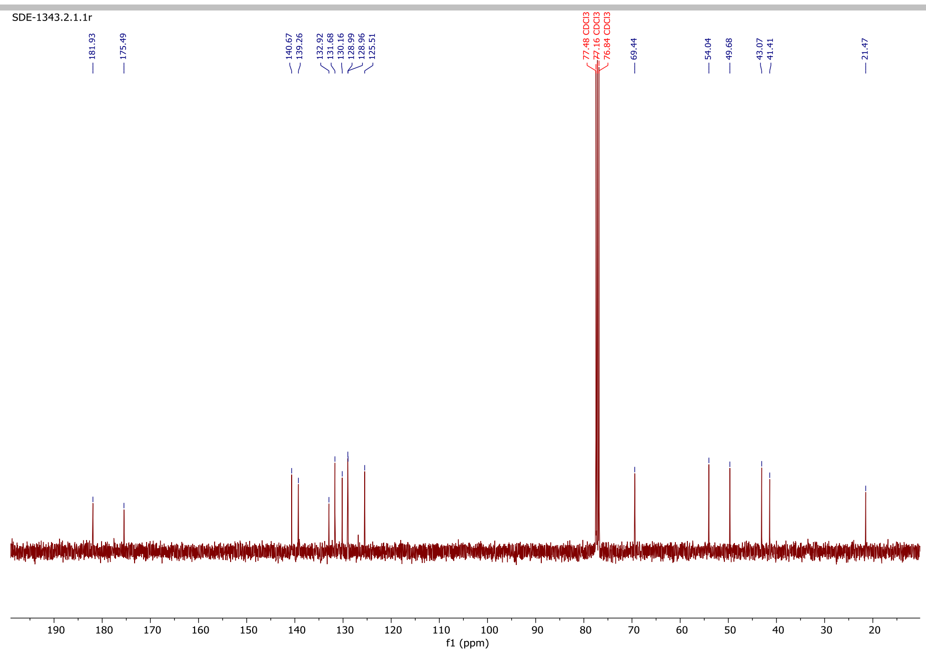

Figure S42. ^{13}C NMR spectra of compound **37b.**

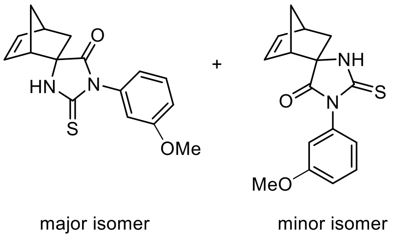

(1S,2S*,4S*)-1′-(3-Methoxyphenyl)-2′-thioxospiro[bicyclo[2.2.1]heptane-2,4′-imidazolidin]-5-en-5′-one* (**38a**) (isolated using chloroform as eluent) and *(1S*,2R*,4S*)-1′-(3-methoxyphenyl)-2′-thioxospiro[bicyclo[2.2.1]heptane-2,4′-imidazolidin]-5-en-5′-one* (**38b**) (isolated using methanol/chloroform (1:400) as eluent). From methylidenethiohydantoin **6** (229 mg, 0.98 mmol) and cyclopentadiene (201 mg, 3.04 mmol) compound **38a** (216 mg, 74%) and compound **38b** (17 mg, 6%) were obtained as a white or pale yellow crystalline solids.

Major isomer 38a: ^{1}**H NMR** (400 MHz, $CDCl_3$): δ 7.93 (bs, 1H), 7.44-7.39 (m, 1H), 7.02-6.99 (m, 1H), 6.95-6.92 (m, 1H), 6.88 (t, J = 2.2 Hz, 1H), 6.59 (dd, J_1 = 3.1 Hz, J_2 = 5.7 Hz, 1H), 6.26 (dd, J_1 = 3.0 Hz, J_2 = 5.7 Hz, 1H), 3.84 (s, 3H), 3.20 – 3.14 (m, 1H), 3.09 (s, 1H), 2.43 (dd, J_1 = 3.5 Hz, J_2 = 12.3 Hz, 1H), 2.26 (d, J = 9.2 Hz, 1H), 1.59-1.55 (m, 1H), 1.49 (dd, J_1 = 3.5 Hz, J_2 = 12.4 Hz, 1H). ^{13}**C NMR** (101 MHz, $CDCl_3$): δ 181.4, 176.4, 159.6, 142.3, 133.5, 133.0, 129.4, 120.2, 114.7, 113.7, 69.3, 55.1, 52.4, 46.8, 42.2, 40.5. **HRMS** (ESI+) m/z calcd. for ($C_{16}H_{17}N_2O_2S$, M+H): 301.1005, found: (M+H): 301.1011.

Minor isomer 38b: ^{1}**H NMR** (400 MHz, $CDCl_3$): δ 9.08 (bs, 1H), 7.42-7.37 (m, 1H), 7.02-6.93 (m, 2H), 6.89-6.84 (m, 1H), 6.43 (dd, J_1 = 3.1 Hz, J_2 = 5.7 Hz, 1H), 6.16 (dd, J_1 = 3.1 Hz, J_2 = 5.7 Hz, 1H), 3.83 (s, 3H), 3.10 (s, 1H), 3.06 (s, 1H), 2.07 (dd, J_1 = 3.6 Hz, J_2 = 12.6 Hz, 1H), 1.89 (d, J = 12.6 Hz, 1H), 1.74 (s, 2H). ^{13}**C NMR** (101 MHz, $CDCl_3$): δ 181.1, 174.9, 159.6, 140.2, 133.5, 131.1, 129.3, 120.1, 114.7, 113.7, 69.0, 55.1, 53.6, 49.2, 42.6, 40.8. **HRMS** (ESI+) m/z calcd. for ($C_{16}H_{17}N_2O_2S$, M+H): 301.1005, found: (M+H): 301.1008.

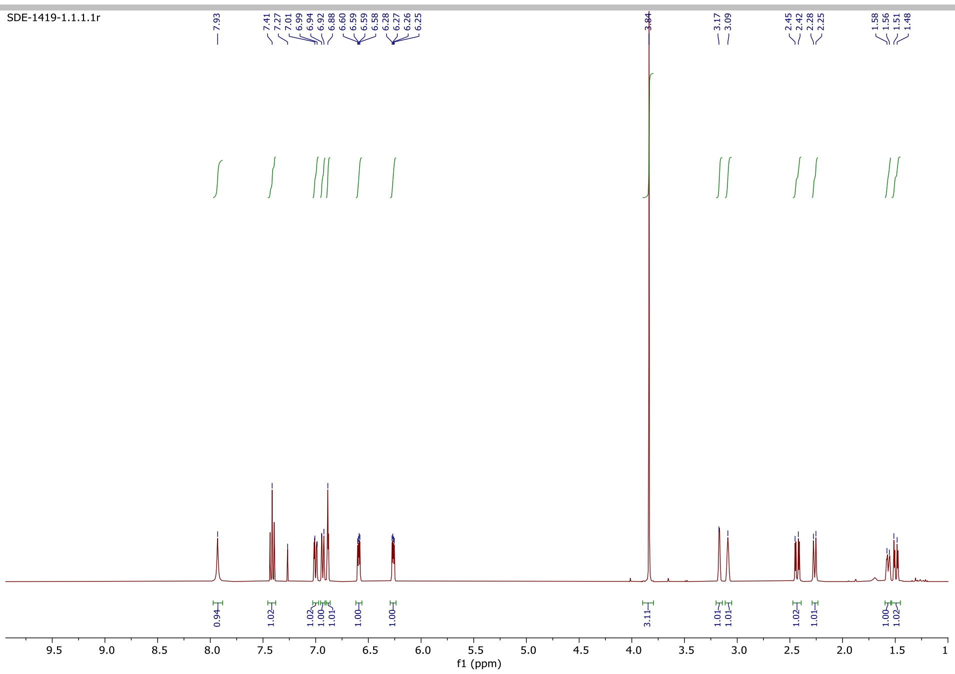

Figure S43. ^1H NMR spectra of compound **38a.**

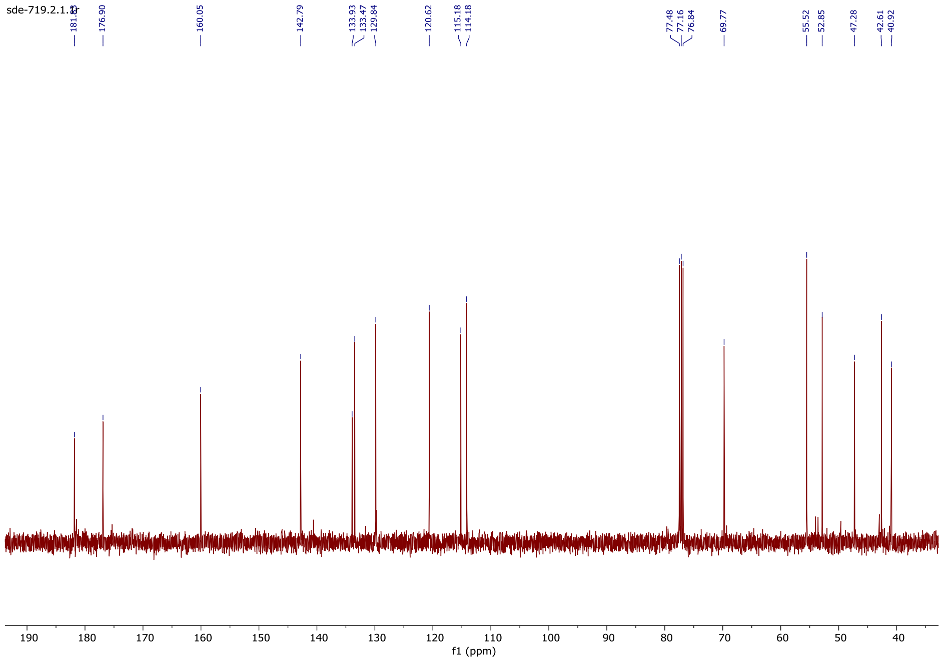

Figure S44. ^{13}C NMR spectra of compound **38a.**

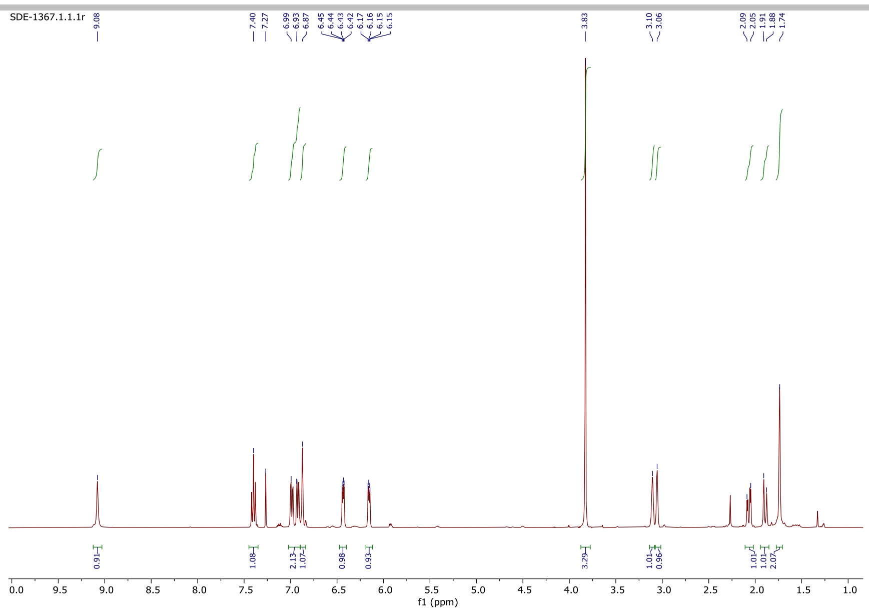

Figure S45. ^{1}H NMR spectra of compound **38b.**

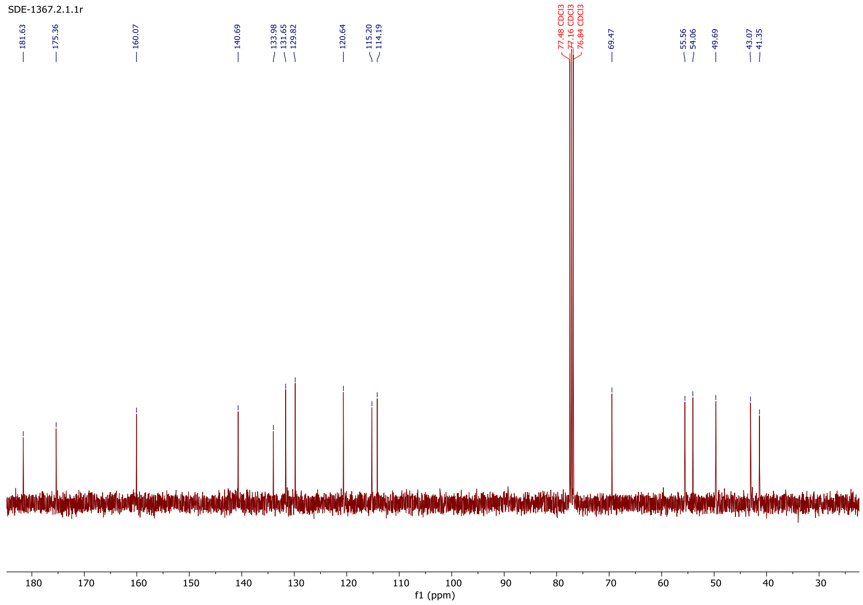

Figure S46. ^{13}C NMR spectra of compound **38b.**

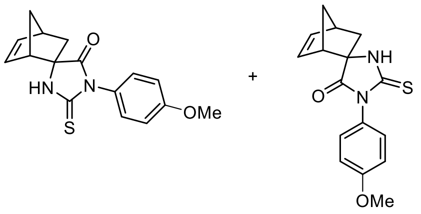

major isomer

minor isomer

(1S,2S*,4S*)-1′-(4-Methoxyphenyl)-2′-thioxospiro[bicyclo[2.2.1]heptane-2,4′-imidazolidin]-5-en-5′-one* (**39a**) (isolated using chloroform as eluent) and *(1S*,2R*,4S*)-1′-(4-methoxyphenyl)-2′-thioxospiro[bicyclo[2.2.1]heptane-2,4′-imidazolidin]-5-en-5′-one* (**39b**) (isolated using methanol/chloroform (1:400) as eluent). From methylidenethiohydantoin **9** (229 mg, 0.98 mmol) and cyclopentadiene (201 mg, 3.04 mmol) compound **39a** (229 mg, 78%) and compound **39b** (23 mg, 8%) were obtained as a white or pale yellow crystalline solids.

Major isomer 39a: ^{1}H NMR (400 MHz, $CDCl_3$): δ 7.36 (bs, 1H), 7.27-7.25 (m, 2H), 7.02-7.00 (m, 2H), 6.62 (dd, J_1 = 3.1 Hz, J_2 = 5.6 Hz, 1H), 6.28 (dd, J_1 = 3.0 Hz, J_2 = 5.6 Hz, 1H), 3.85 (s, 3H), 3.17 (s, 1H), 3.11 (s, 1H), 2.45 (dd, J_1 = 3.5 Hz, J_2 = 12.4 Hz, 1H), 2.28 (d, J = 8.7 Hz, 1H), 1.59-1.57 (m, 1H), 1.49 (dd, J_1 = 3.4 Hz, J_2 = 12.4 Hz, 1H). **^{13}C NMR** (101 MHz, $CDCl_3$): δ 182.0, 176.7, 159.5, 142.4, 132.9, 129.0 (2C), 125.1, 114.0

(2C), 69.1, 55.1, 52.3, 46.9, 42.2, 40.6. **HRMS** (ESI+) m/z calcd. for ($C_{16}H_{17}N_2O_2S$, M+H): 301.1005, found: (M+H): 301.1006.

Minor isomer 39b: ^{1}**H NMR** (400 MHz, $CDCl_3$): δ ^{1}H NMR (400 MHz, $CDCl_3$) δ 8.98 (bs, 1H), 7.29-7.18 (m, 2H), 7.06-6.96 (m, 2H), 6.43 (dd, J_1 = 3.1 Hz, J_2 = 5.6 Hz, 1H), 6.15 (dd, J_1 = 3.1 Hz, J_2 = 5.7 Hz, 1H), 3.84 (s, 3H), 3.10 (s, 1H), 3.05 (s, 1H), 2.06 (dd, J_1 = 3.6 Hz, J_2 = 12.6 Hz, 1H), 1.93-1.80 (m, 1H), 1.75 (s, 2H). ^{13}**C NMR** (101 MHz, $CDCl_3$): δ 181.6, 175.1, 159.4, 140.2, 131.2, 129.0 (2C), 125.1, 114.0 (2C), 68.9, 55.1, 53.5, 49.2, 42.6, 40.8. **HRMS** (ESI+) m/z calcd. for ($C_{16}H_{17}N_2O_2S$, M+H): 301.1005, found: (M+H): 301.1007.

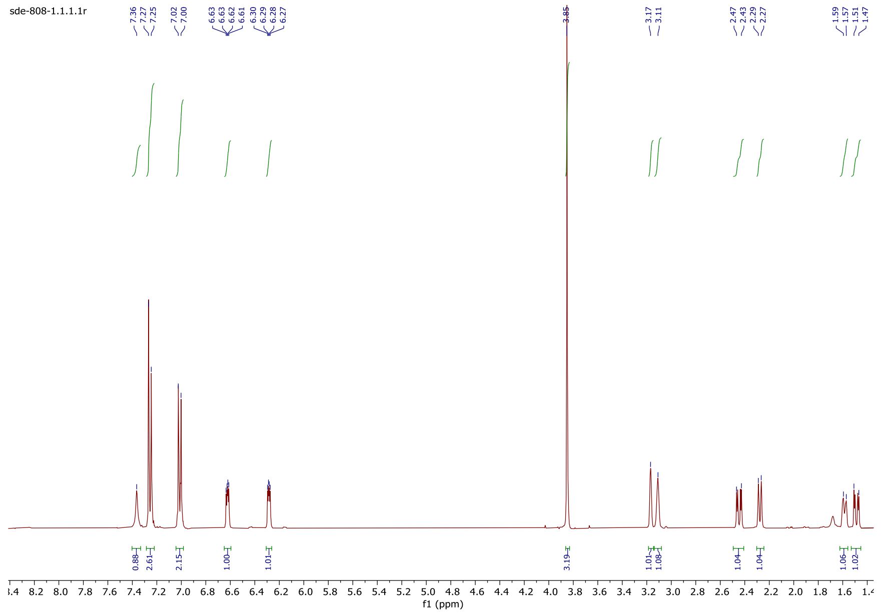

Figure S47. ^{1}H NMR spectra of compound **39a.**

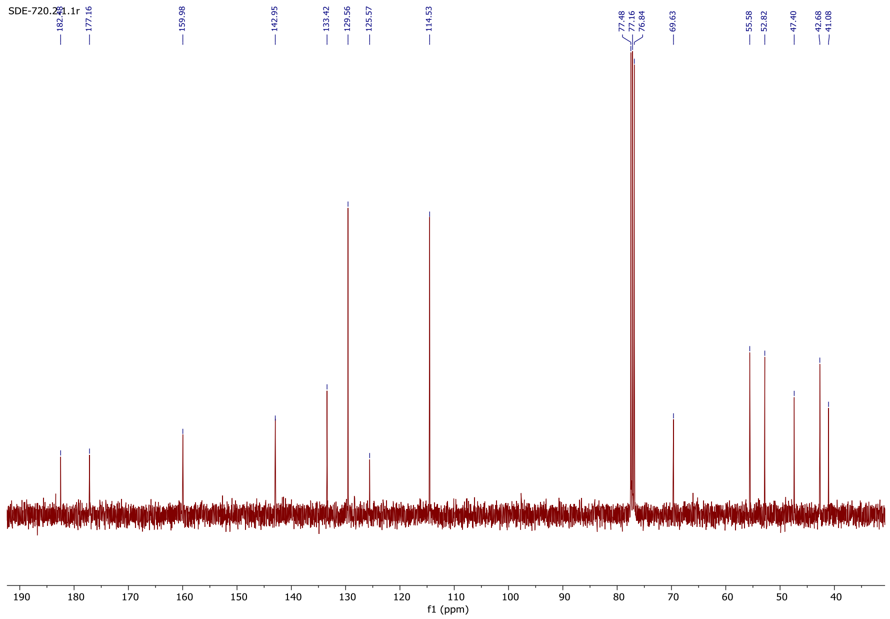

Figure S48. ^{13}C NMR spectra of compound **39a.**

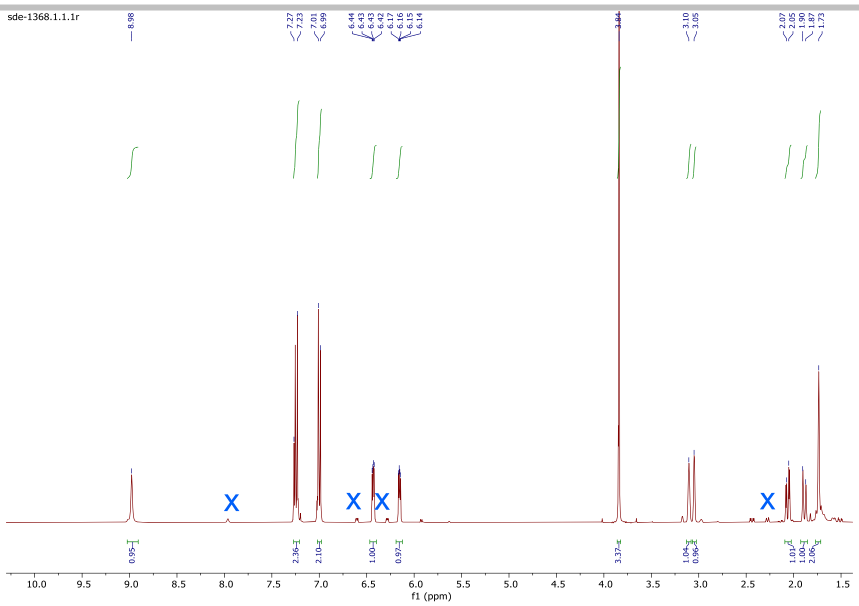

Figure S49. ^{1}H NMR spectra of compound **39b** (*the sign "x" marks the residual signals of isomer **39a***).

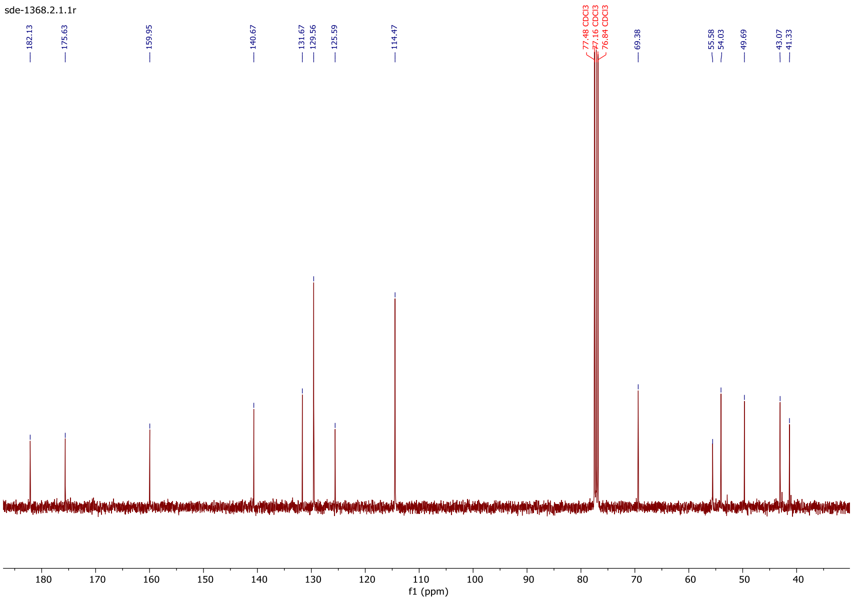

Figure S50. ^{13}C NMR spectra of compound **39b.**

General procedure of Diels-Alder reaction of 5-halomethylidenehydantoins 17, 18 with cyclopentadiene.

To a solution of 5-halomethylidenehydantoin (0.37 mmol) in o-xylene (30 ml) cyclopentadiene (488 mg, 7.40 mmol) was added and the mixture was refluxed for 13 h. The reaction was then cooled to room temperature and the mixture was evaporated under vacuum. The residue was purified by column chromatography on silica gel.

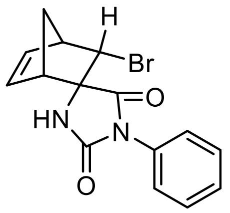

(1S,2S*,3R*,4R*)-3-bromo-1′-phenylspiro[bicyclo[2.2.1]heptane-2,4′-imidazolidin]-5-ene-2′,5′-dione* (**40a**) (isolated using chloroform as eluent). From 5-bromomethylidenehydantoin **17** (99 mg, 0.37 mmol) and cyclopentadiene (488 mg, 7.40 mmol) compound **40a** (79 mg, 64%) was obtained as a white crystalline solid.

^{1}H NMR (400 MHz, $CDCl_3$): δ 7.53-7.34 (m, 5H), 6.52 (dd, J_1 = 3.0 Hz, J_2 = 5.8 Hz, 1H), 6.41 (dd, J_1 = 3.1 Hz, J_2 = 5.8 Hz, 1H), 5.75 (bs, 1H), 4.75 (d, J = 3.4 Hz, 1H), 3.40-3.34 (m, 1H), 3.21-3.19 (m, 1H), 2.43 (d, J = 9.8 Hz, 1H), 1.86-1.79 (m, 1H). **^{13}C NMR** (101 MHz, $CDCl_3$): δ 174.0, 154.6, 140.8, 135.2, 131.3, 129.1 (2C), 128.3, 126.1 (2C), 66.1, 58.1, 51.8, 49.5, 44.6. **HRMS** (ESI+) m/z calcd. for ($C_{15}H_{14}BrN_2O_2$, M+H): 333.0233, found: (M+H): 333.0237.

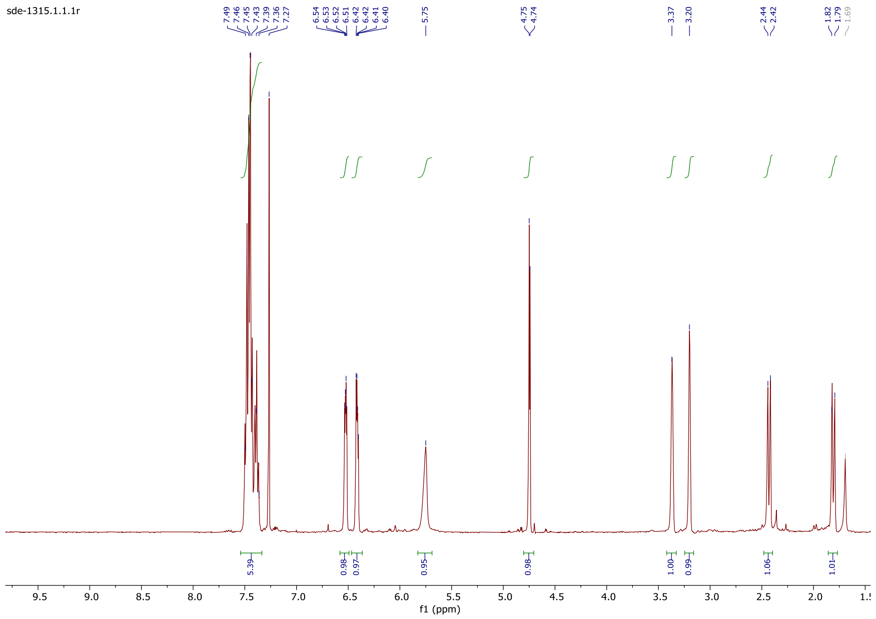

Figure S51. ^{1}H NMR spectra of compound **40a.**

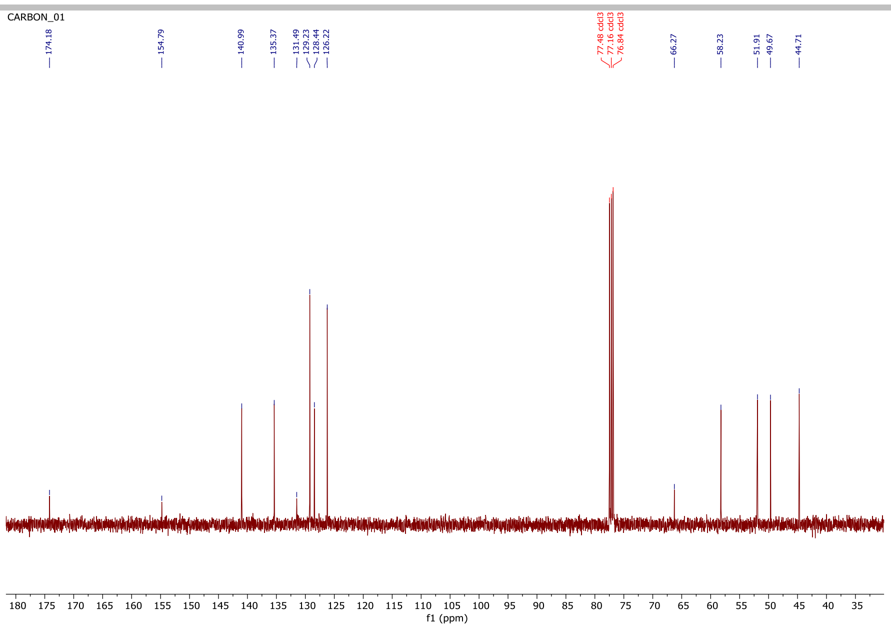

Figure S52. ^{13}C NMR spectra of compound **40a.**

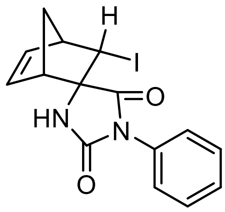

(1S,2S*,3R*,4R*)-3-Iodo-1′-phenylspiro[bicyclo[2.2.1]heptane-2,4′-imidazolidin]-5-ene-2′,5′-dione* (**41a**) (isolated using chloroform as eluent). From 5-iodomethylidenehydantoin **18** (116 mg, 0.37 mmol) and cyclopentadiene (488 mg, 7.40 mmol) compound **41a** (52 mg, 37%) was obtained as a white crystalline solid.

^{1}H NMR (400 MHz, $CDCl_3$): δ 7.53-7.40 (m, 4H), 7.44-7.34 (m, 1H), 6.47 (dd, J_1 = 3.0 Hz, J_2 = 5.8 Hz, 1H), 6.39 (dd, J_1 = 3.0 Hz, J_2 = 5.8 Hz, 1H), 5.84 (s, 1H), 4.73 (d, J = 3.2 Hz, 1H), 3.38 (s, 1H), 3.16 (s, 1H), 2.50 (d, J = 9.8 Hz, 1H), 1.87 (dt, J_1 = 1.9 Hz, J_2 = 9.8 Hz, 1H). **^{13}C NMR** (101 MHz, $CDCl_3$): δ 173.2, 154.2, 142.5, 134.7, 131.0, 128.7 (2C), 127.9, 125.7 (2C), 65.0, 50.2, 50.2, 45.4, 36.8. **HRMS** (ESI+) m/z calcd. for ($C_{15}H_{14}IN_2O_2$, M+H): 381.0095, found: (M+H): 381.0095.

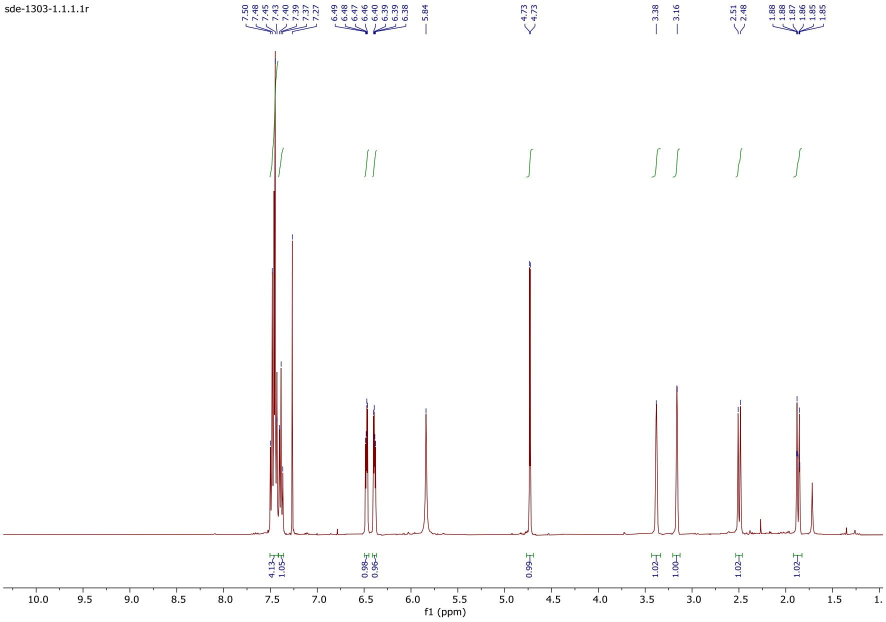

Figure S53. ^{1}H NMR spectra of compound **41a.**

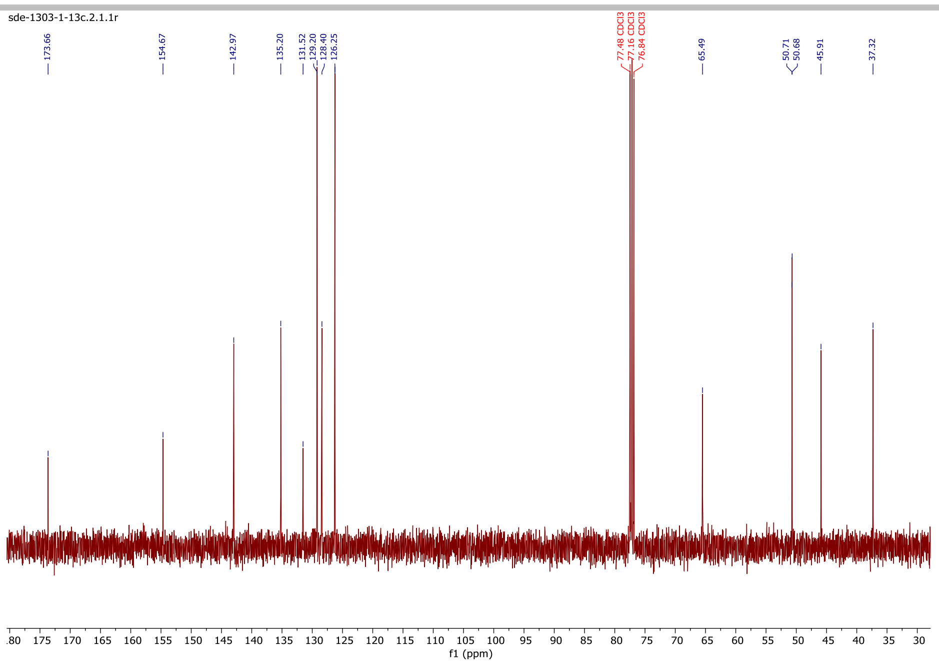

Figure S54. ^{13}C NMR spectra of compound **41a.**

General procedure of Diels-Alder reaction of methylideneimidazolones 1-3, 5, 6, 8-10 with 1,3-cyclohexadiene.

To a solution of methylideneimidazolone (0.49 mmol) and $AlCl_3$ or ZnI_2 (0.49 mmol) in boiling chloroform (30 ml) 1,3-cyclohexadiene (196 mg, 2.45 mmol) was added and the mixture was refluxed for 6 h. The reaction was then cooled to room temperature, the mixture was washed with water (15 ml) and filtered. The organic phase was dried over anhydrous Na_2SO_4, filtered and evaporated under vacuum. The residue was purified by column chromatography on silica gel.

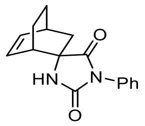

(1S,2S*,4S*)-1′-Phenylspiro[bicyclo[2.2.2]octane-2,4′-imidazolidin]-5-ene-2′,5′-dione* (**44a**) (isolated using methanol/chloroform (1:600) as eluent). From methylidenehydantoin **1** (92 mg, 0.49 mmol), $AlCl_3$ (65 mg, 0.49 mmol) and 1,3-cyclohexadiene (196 mg, 2.45 mmol) compound **44a** (21 mg, 16%) was obtained as a white crystalline solid.

^{1}H NMR (400 MHz, $CDCl_3$): δ 7.53-7.32 (m, 5H), 6.58-6.54 (m, 1H), 6.37-6.33 (m, 1H), 5.52 (bs, 1H), 2.83-2.79 (m, 2H), 2.42-2.30 (m, 1H), 2.26 (dd, J_1 = 2.0 Hz, J_2 = 13.6 Hz, 1H), 1.74-1.68 (m, 1H), 1.60 (dt, J_1 = 2.4 Hz, J_2 = 13.6 Hz, 1H), 1.35-1.20 (m, 2H), 1.16-1.12 (m, 2H). **^{13}C NMR** (101 MHz, $CDCl_3$): δ 175.1, 154.8, 137.8, 131.7, 131.2, 129.0 (2C), 128.0, 126.1 (2C), 63.7, 39.7, 39.3, 30.2, 23.8, 18.5. **HRMS** (ESI+) m/z calcd. for ($C_{16}H_{17}N_2O_2$, M+H): 269.1285, found: (M+H): 269.1273.

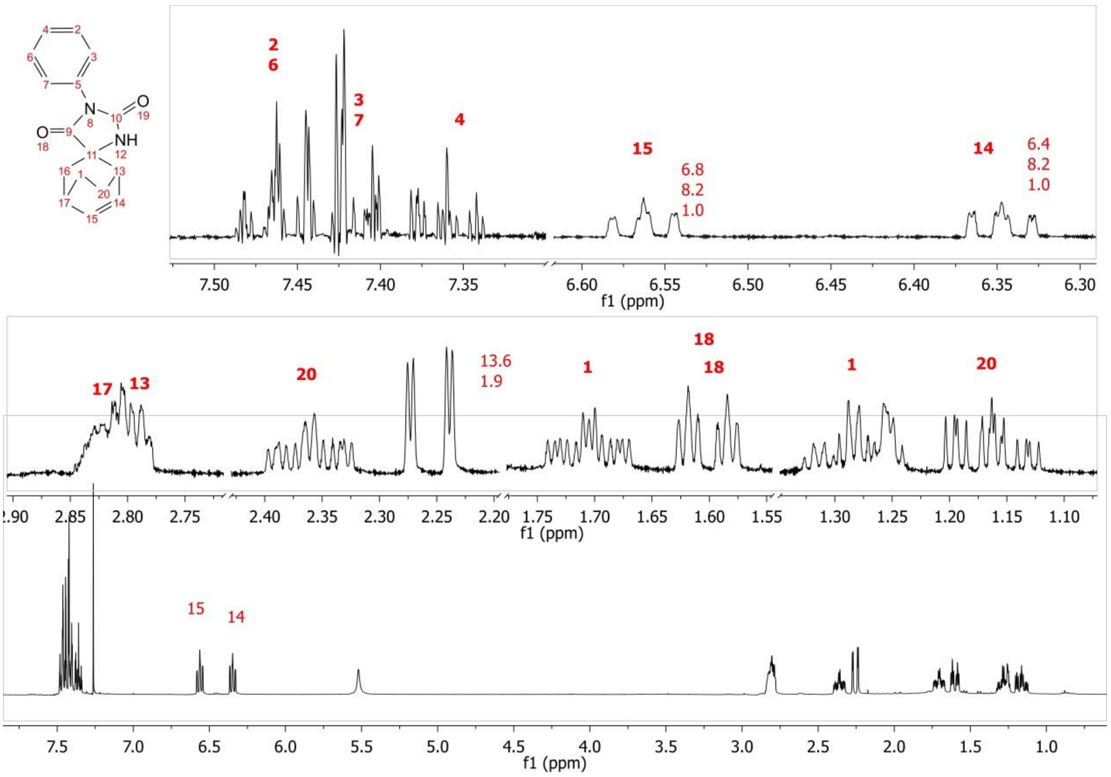

Figure S55. ^{1}H NMR spectra of compound **44a.**

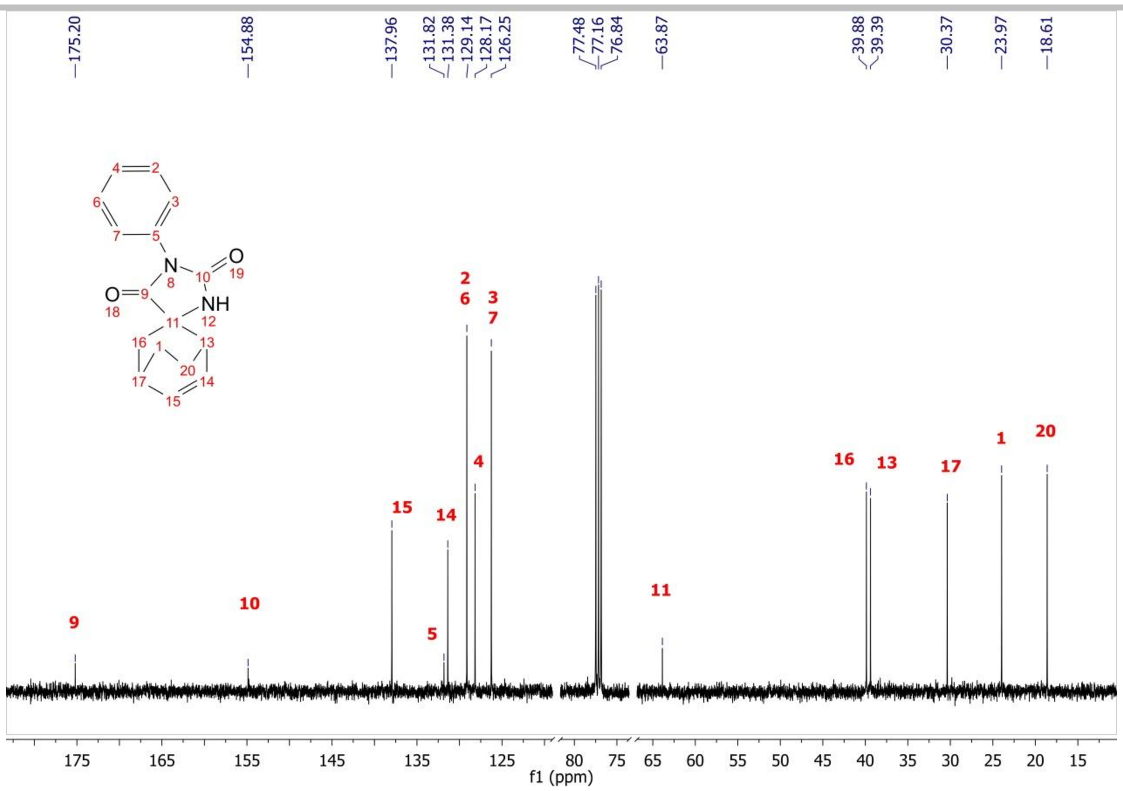

Figure S56. ^{13}C NMR spectra of compound **44a.**

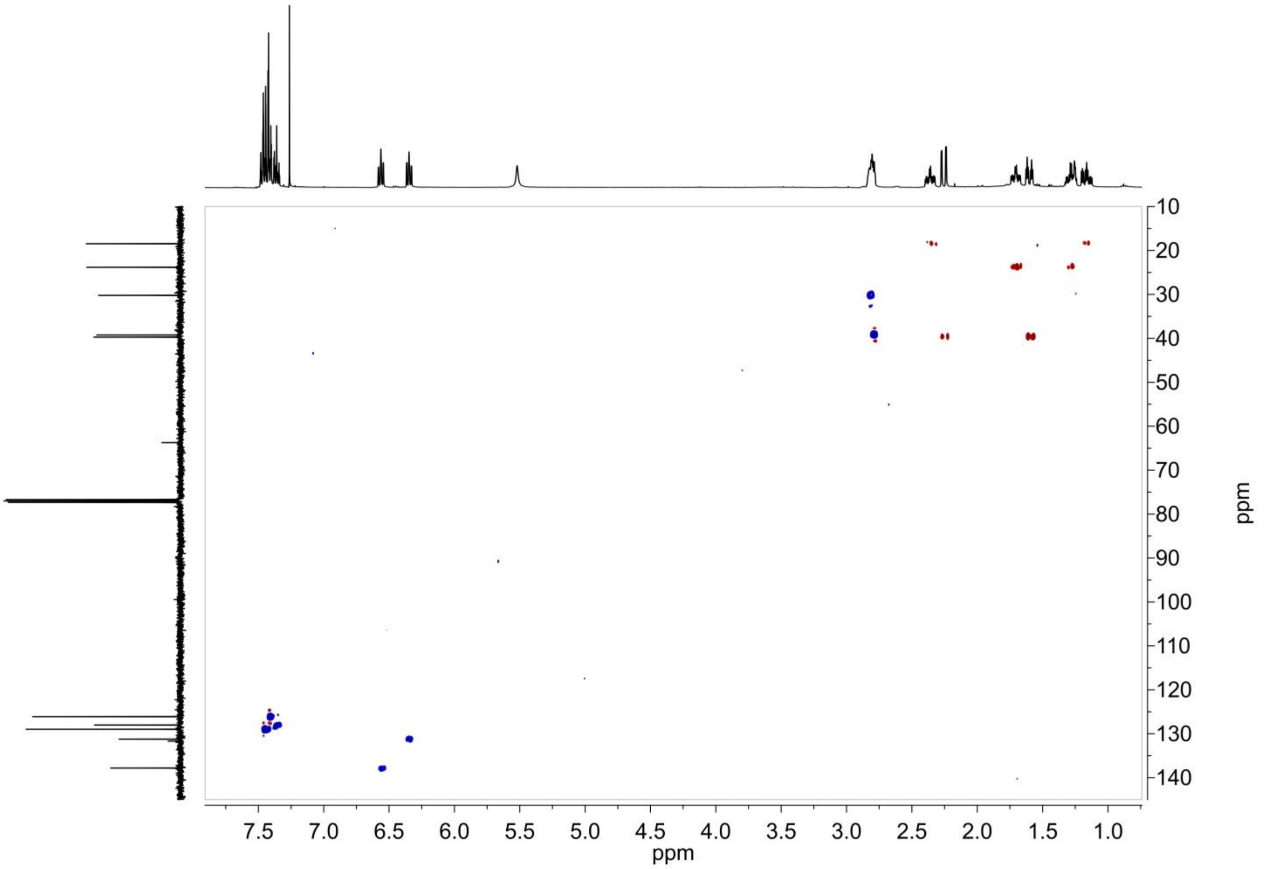

Figure S57. HSQC ^{1}H-^{13}C NMR spectra of of compound **44a**.

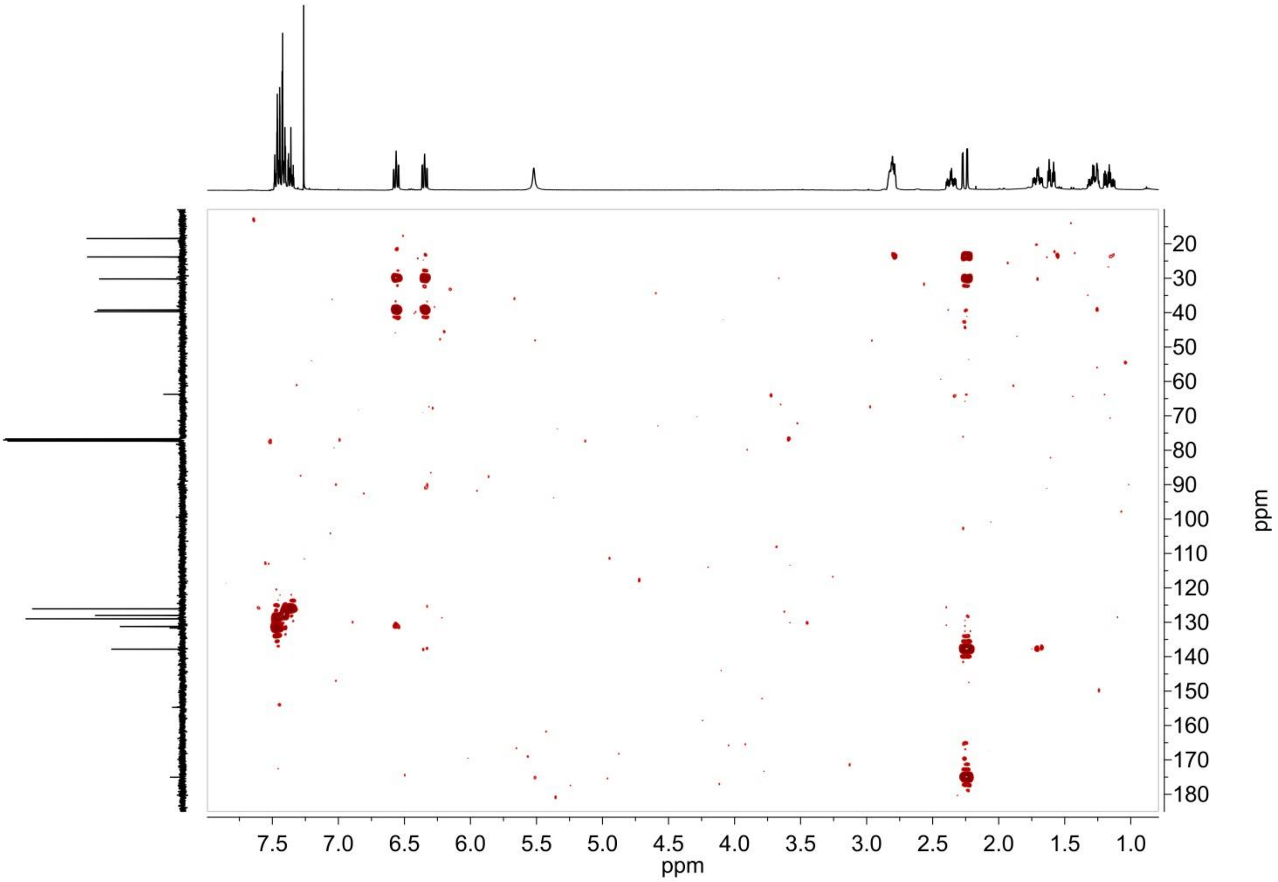

Figure S58. HMBC ^{1}H-^{13}C NMR spectra of of compound **44a**.

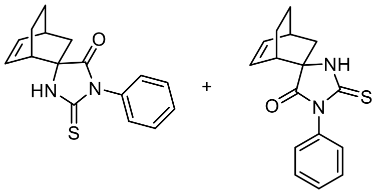

major isomer minor isomer

(1R,2R*,4R*)-1′-Phenyl-2′-thioxospiro[bicyclo[2.2.2]oct[5]ene-2,4′-imidazolidin]-5′-one* (**45a**) (isolated using chloroform as eluent) and *(1S*,2R*,4S*)-1′-phenyl-2′-thioxospiro[bicyclo[2.2.2]oct[5]ene-2,4′-imidazolidin]-5′-one* (**45b**) (isolated using methanol/chloroform (1:600) as eluent). From methylidenethiohydantoin **2** (100 mg, 0.49 mmol), ZnI_2 (156 mg, 0.49 mmol) and 1,3-cyclohexadiene (196 mg, 2.45 mmol) compound **45a** (75 mg, 54%) and compound **45b** (27 mg, 19%) were obtained as a white or pale yellow crystalline solids.

Major isomer 45a: ^{1}**H NMR** (400 MHz, $CDCl_3$): δ 7.60 (bs, 1H), 7.53-7.43 (m, 3H), 7.34-7.31 (m, 2H), 6.62-6.58 (m, 1H), 6.40-6.36 (m, 1H), 2.87-2.86 (m, 2H), 2.38-2.32 (m, 1H), 2.21 (dd, J_1 = 1.8 Hz, J_2 = 13.7 Hz, 1H), 1.73-1.65 (m, 2H), 1.34-1.29 (m, 1H), 1.20-1.12 (m, 1H). ^{13}**C NMR** (101 MHz, $CDCl_3$): δ 180.9, 175.2, 137.4, 132.5, 130.8, 128.7, 128.6 (2C), 127.9 (2C), 66.2, 38.6, 38.1, 29.6, 23.3, 17.4. **HRMS** (ESI+) m/z calcd. for ($C_{16}H_{17}N_2OS$, M+H): 285.1056, found: (M+H): 285.1057.

Minor isomer 45b: ^{1}**H NMR** (400 MHz, $CDCl_3$): δ 8.69 (bs, 1H), 7.52-7.43 (m, 3H), 7.33-7.31 (m, 2H), 6.48-6.44 (m, 1H), 6.34-6.30 (m, 1H), 2.83-2.82 (m, 1H), 2.77-2.76 (m, 1H), 2.07-2.03 (m, 1H), 1.99-1.95 (m, 1H), 1.78 (dd, J_1 = 2.7 Hz, J_2 = 13.5 Hz, 1H), 1.48-1.32 (m, 3H). ^{13}**C NMR** (101 MHz, $CDCl_3$): δ 181.2, 175.5, 134.8, 132.4, 130.4, 128.7, 128.6 (2C), 127.9 (2C), 65.6, 38.4, 37.8, 29.2, 22.4, 21.0. **HRMS** (ESI+) m/z calcd. for ($C_{16}H_{17}N_2OS$, M+H): 285.1056, found: (M+H): 285.1055.

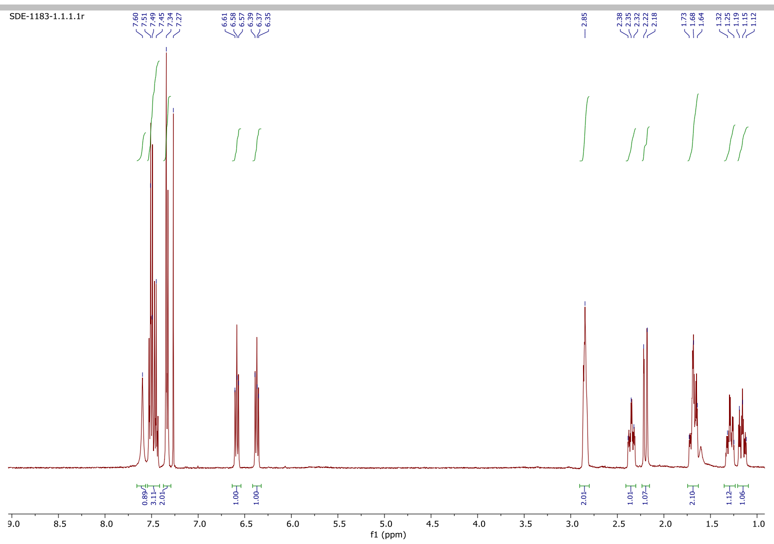

Figure S59. ^{1}H NMR spectra of compound **45a.**

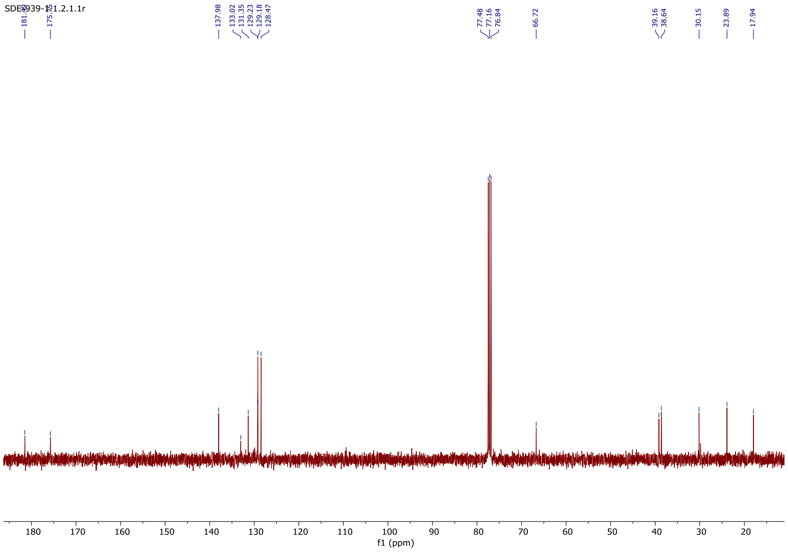

Figure S60. ^{13}C NMR spectra of compound **45a.**

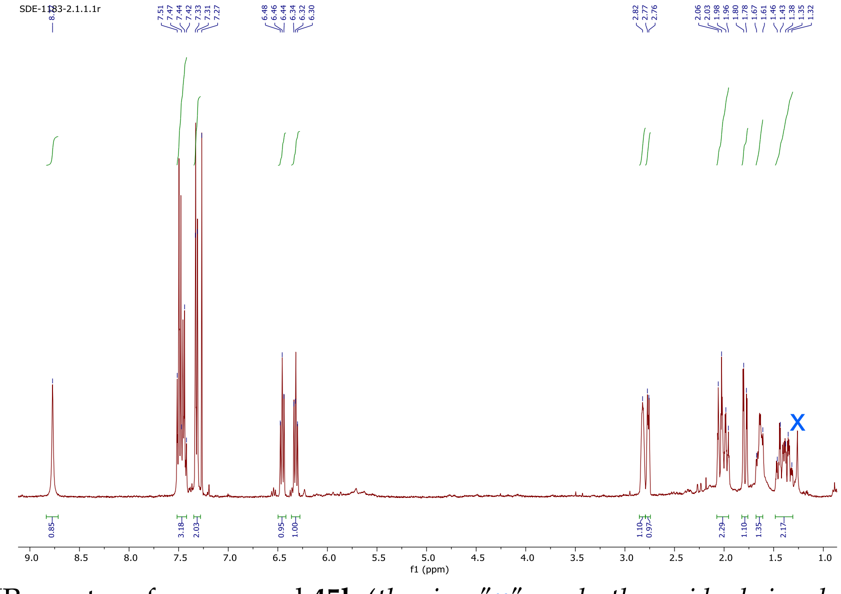

Figure S61. ^{1}H NMR spectra of compound **45b** (*the sign "x" marks the residual signals of tar*).

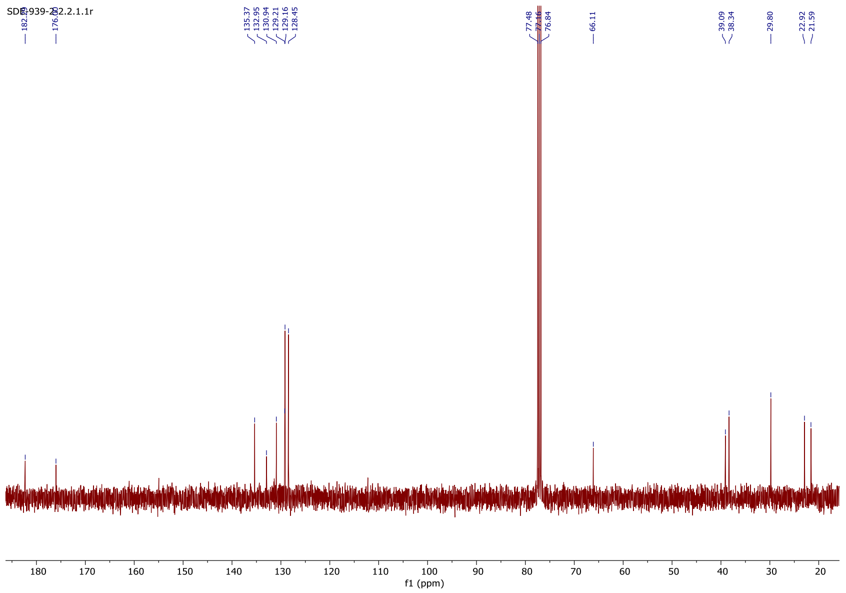

Figure S62. ^{13}C NMR spectra of compound **45b.**

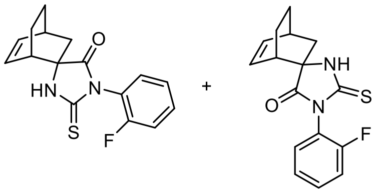

major isomer minor isomer

(1R,2R*,4R*)-1′-(2-Fluorophenyl)-2′-thioxospiro[bicyclo[2.2.2]oct[5]ene-2,4′-imidazolidin]-5′-one* (**46a**) (isolated using chloroform as eluent) and *(1S*,2R*,4S*)-1′-(2-fluorophenyl)-2′-thioxospiro[bicyclo[2.2.2]oct[5]ene-2,4′-imidazolidin]-5′-one* (**46b**) (isolated using methanol/chloroform (1:600) as eluent). From methylidenethiohydantoin **3** (109 mg, 0.49 mmol), ZnI_2 (156 mg, 0.49 mmol) and 1,3-cyclohexadiene (196 mg, 2.45 mmol) compound **46a** (65 mg, 44%) and compound **46b** (22 mg, 15%) were obtained as a white or pale yellow crystalline solids.

Major isomer 46a: ^{1}**H NMR** (400 MHz, DMSO-d6): δ 10.60 (bs, $1H_3$), 10.59 (bs, $1H_{3'}$), 7.53-7.26 (m, $1H_c$+$1H_{c'}$+$1H_d$+$1H_{d'}$+$1H_e$+$1H_{e'}$+$1H_f$+$1H_{f'}$), 7.34-7.31 (m, 3H), 6.47-6.44 (m, $1H_7$+$1H_{7'}$), 6.34-6.29 (m, $1H_8$+$1H_{8'}$), 2.91-2.90 (m, $1H_{9'}$), 2.78-2.77 (m, $1H_6$+$1H_{6'}$), 2.67-2.66 (m, $1H_9$), 2.16-2.08 (m, $1H_{10}$+$1H_{10'}$), 2.02 (dd, J_1 = 2.0 Hz, J_2 = 13.5 Hz, $1H_5$), 1.95 (dd, J_1 = 2.0 Hz, J_2 = 13.5 Hz, $1H_{5'}$), 1.55 (d, J = 13.5 Hz, $1H_5$+$1H_{5'}$), 1.55-1.46 (m, $1H_{11}$+$1H_{11'}$), 1.22-1.18 (m, $1H_{11}$+$1H_{11'}$), 1.02-0.98 (m, $1H_{10}$+$1H_{10'}$). ^{13}**C NMR** (101 MHz, DMSO-d6): (two sets of signals for C(Ar)-N – rotamers) δ 179.7 (s, $C_{2'}$), 179.6 (s, C_2), 175.7 (s, $C_{12'}$), 175.6 (s, C_{12}), 157.8 (d, J = 250.0 Hz, C_b), 157.5 (d, J = 250.0 Hz, $C_{b'}$), 136.0 (s, C_7+$C_{7'}$), 131.9 (s, C_8), 131.7 (d, J = 12.0 Hz, C_f+$C_{f'}$), 131.6 (s, $C_{8'}$), 131.5 (d, J = 8.1 Hz, $C_{d'}$), 131.3 (d, J = 8.1 Hz, C_d), 124.8 (d, J = 3.7 Hz, $C_{e'}$), 124.7 (d, J = 3.7 Hz, C_e), 116.1 (d, J = 19.3 Hz, C_c+$C_{c'}$), 66.4 (s, C_4), 66.3 (s, $C_{4'}$), 38.5 (s, C_9), 38.3 (s, $C_{5'}$), 37.8 (s, C_5), 37.5 (s, $C_{9'}$), 29.4 (s, $C_{6'}$), 29.3 (s, C_6), 23.2 (s, $C_{11'}$), 23.1(s, C_{11}), 18.1 (s, $C_{10'}$), 18.0 (s, C_{10}). ^{19}**F NMR** (376 MHz, DMSO-d6): δ -120.01 (ddd, J_1 = 5.4 Hz, J_2 = 7.6 Hz, J_3 = 10.1 Hz), -121.56 (ddd, J_1 = 5.4 Hz, J_2 = 7.6 Hz, J_3 = 10.2 Hz). **HRMS** (ESI+) m/z calcd. for ($C_{16}H_{16}FN_2OS$, M+H): 303.0962, found: (M+H): 303.0957.

Minor isomer 46b: ^{1}**H NMR** (400 MHz, DMSO-d6): δ 11.00 (bs, 1H), 7.52-7.26 (m, 4H), 6.37-6.33 (m, 1H), 6.20-6.16 (m, 1H), 2.80-2.55 (m, 2H), 2.03-1.97 (m, 1H), 1.91-1.64 (m, 3H), 1.23-1.21 (m, 1H), 1.13-1.10 (m, 1H). ^{13}**C NMR** (101 MHz, DMSO-d6): (two sets of signals for C(Ar)-N – rotamers) δ 180.7 (s), 180.6 (s), 176.1 (s), 176.0 (s), 158.2 (d, J = 249.8 Hz), 158.0 (d, J = 249.8 Hz), 135.3 (s), 135.2 (s), 132.2 (d, J = 15.1 Hz, C+C′), 131.9 (d, J = 7.9 Hz), 131.8 (d, J = 7.9 Hz), 131.7 (s), 131.5 (s), 125.2 (d, J = 3.6 Hz), 125.1 (d, J = 3.6 Hz), 121.5 (d, J = 12.6 Hz, C+C′), 116.6 (d, J = 19.4 Hz), 116.5 (d, J = 19.4 Hz), 66.4 (s), 66.3 (s), 38.9 (s),

38.3 (s), 37.9 (s), 37.8 (s), 29.6 (s), 29.5 (s), 22.7 (s, C+C′), 21.3 (s), 21.2 (s). **HRMS** (ESI+) m/z calcd. for ($C_{16}H_{16}FN_2OS$, M+H): 303.0962, found: (M+H): 303.0963.

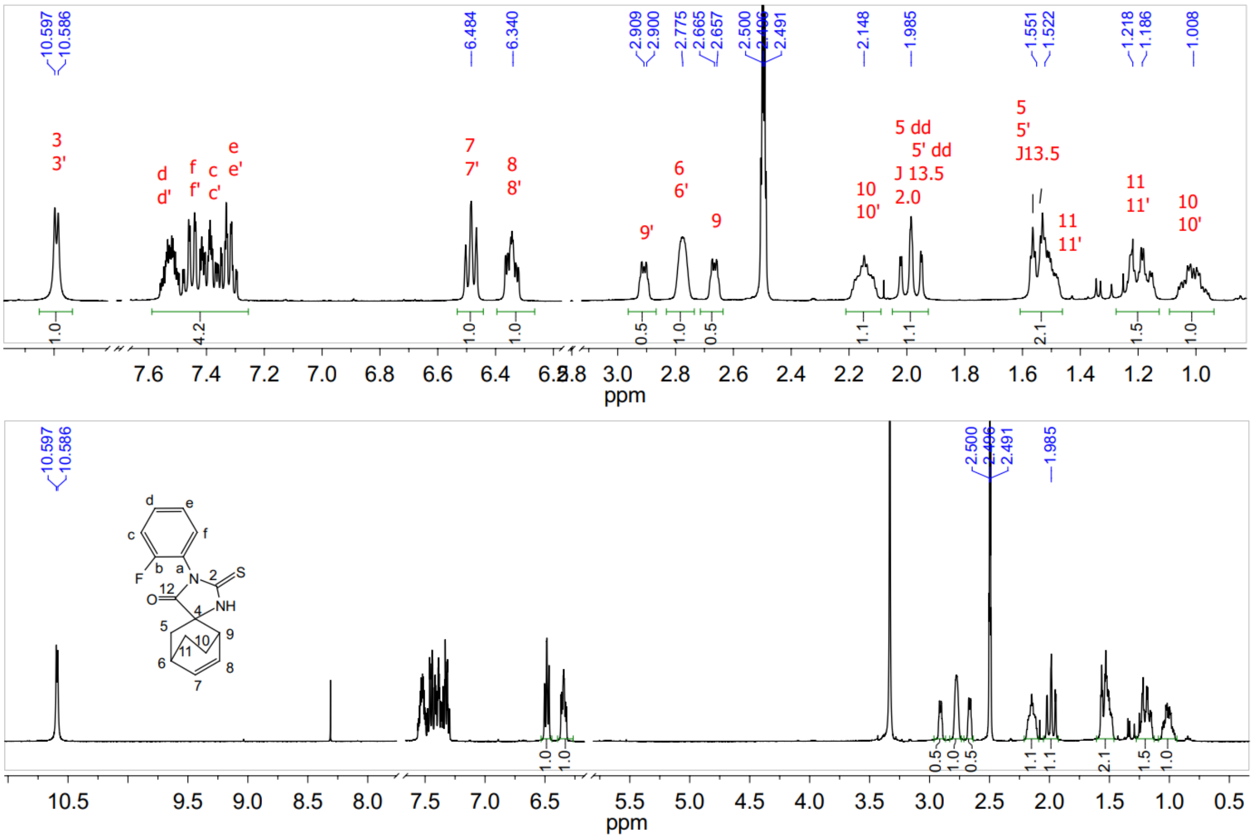

Figure S63. ^{1}H NMR spectra of compound **46a.**

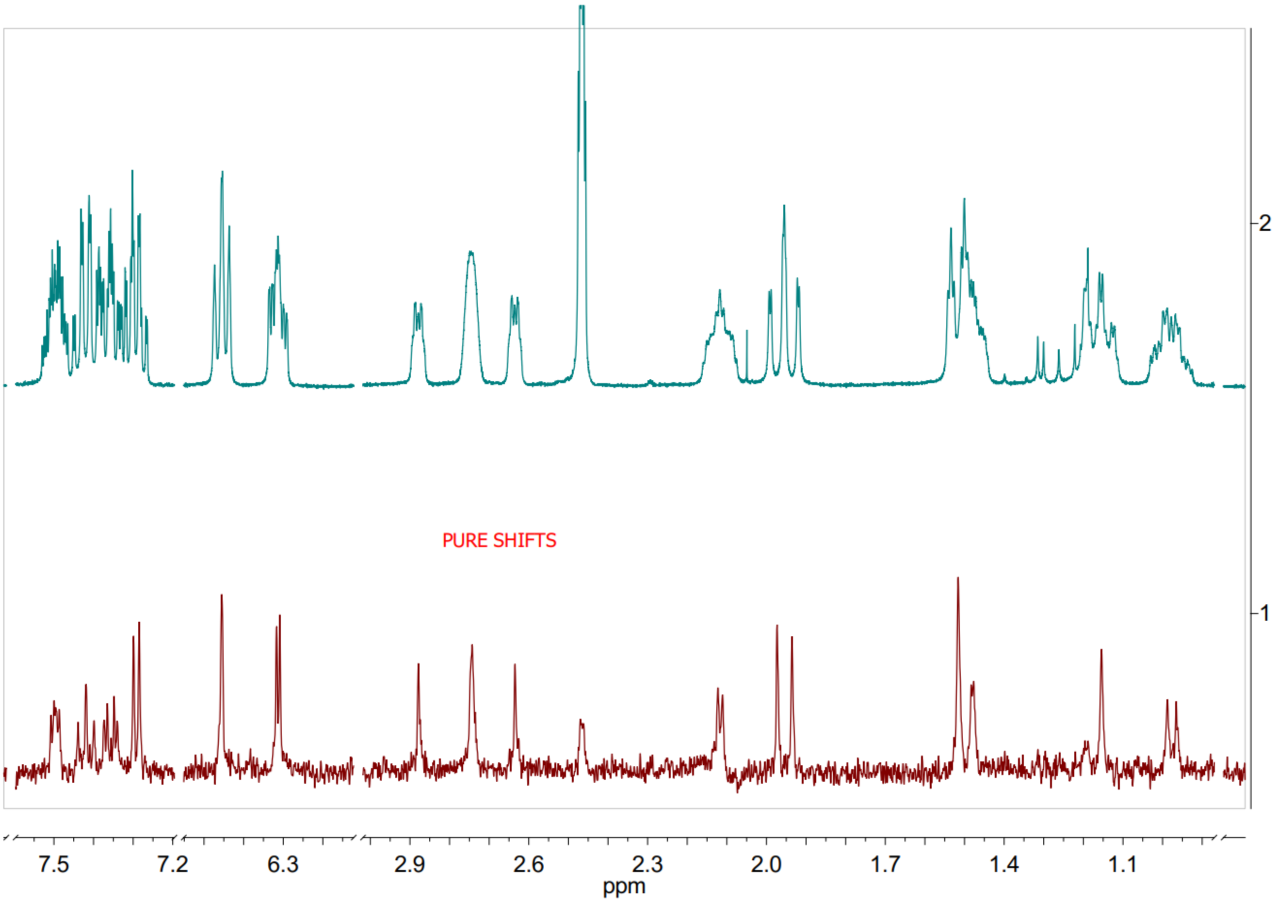

Figure S64. ^{1}H NMR spectra (pureshift) of compound **46a.**

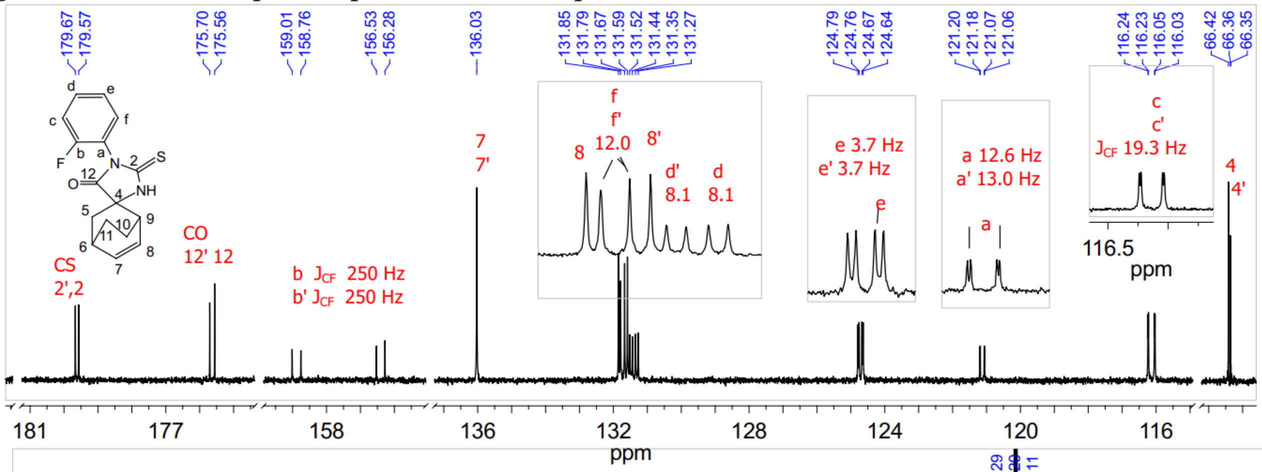

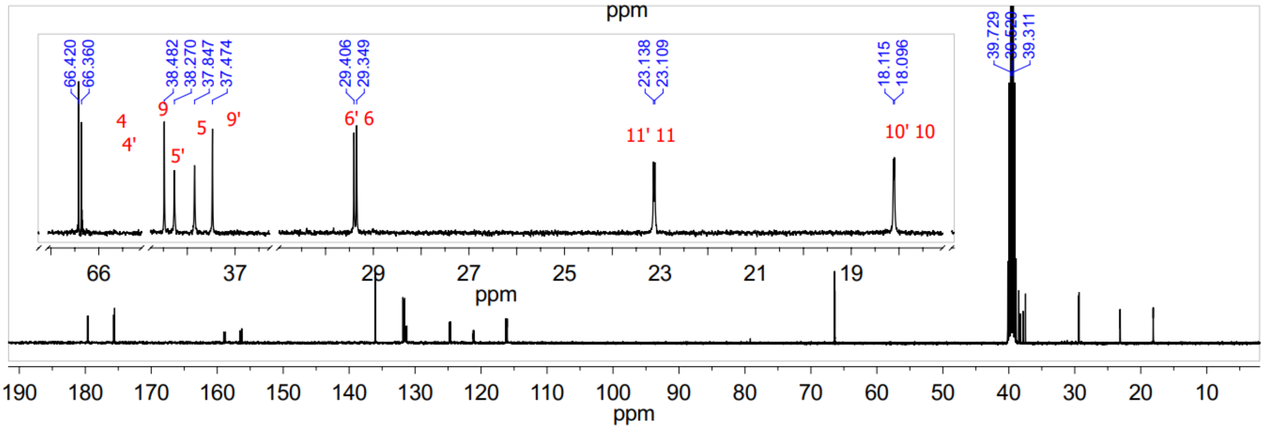

Figure S65. ^{13}C NMR spectra of compound **46a.**

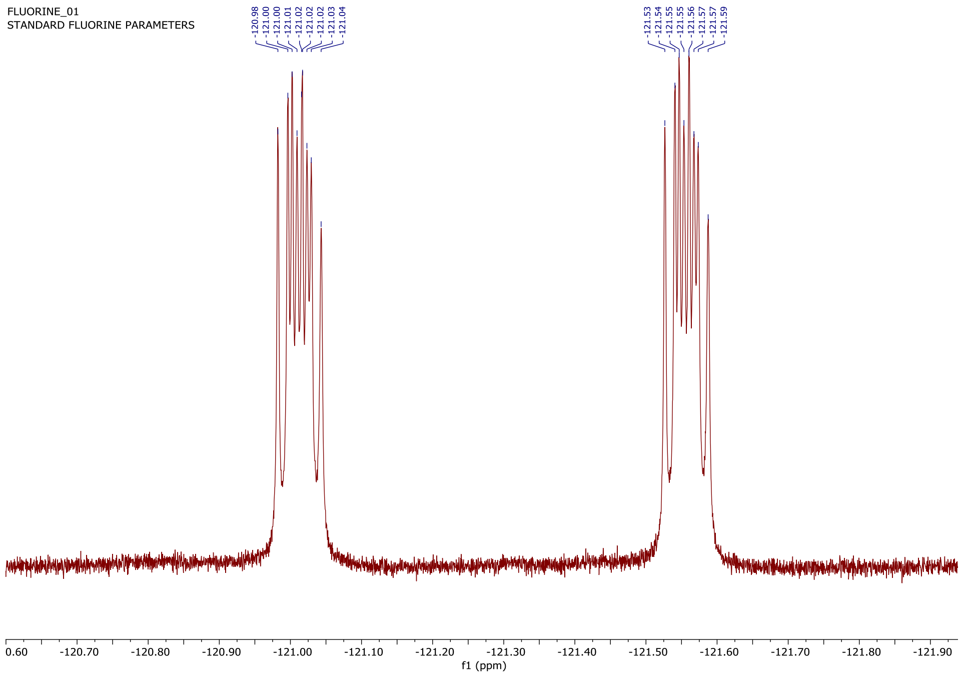

Figure S66. ^{19}F NMR spectra of compound **46a.**

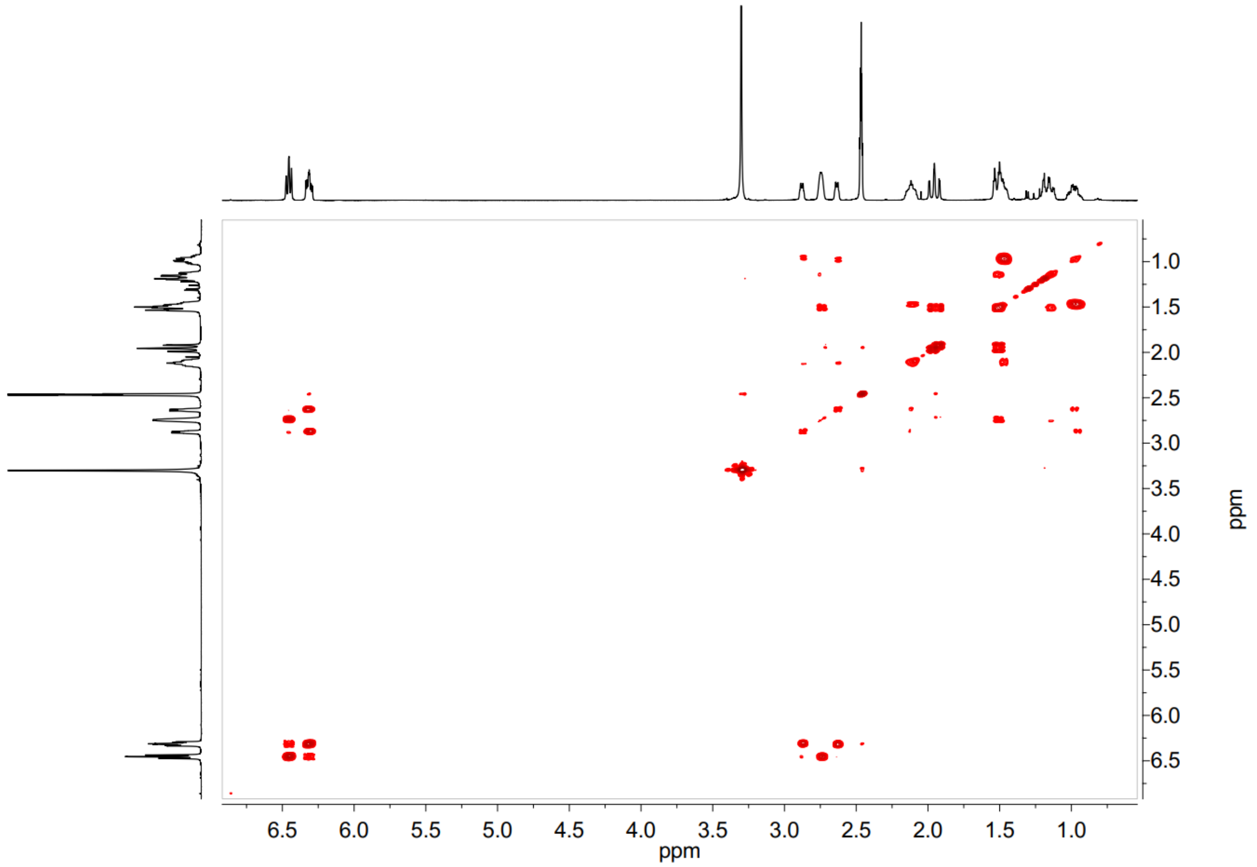

Figure S67. COSY ^{1}H-^{1}H NMR spectra of compound **46a.**

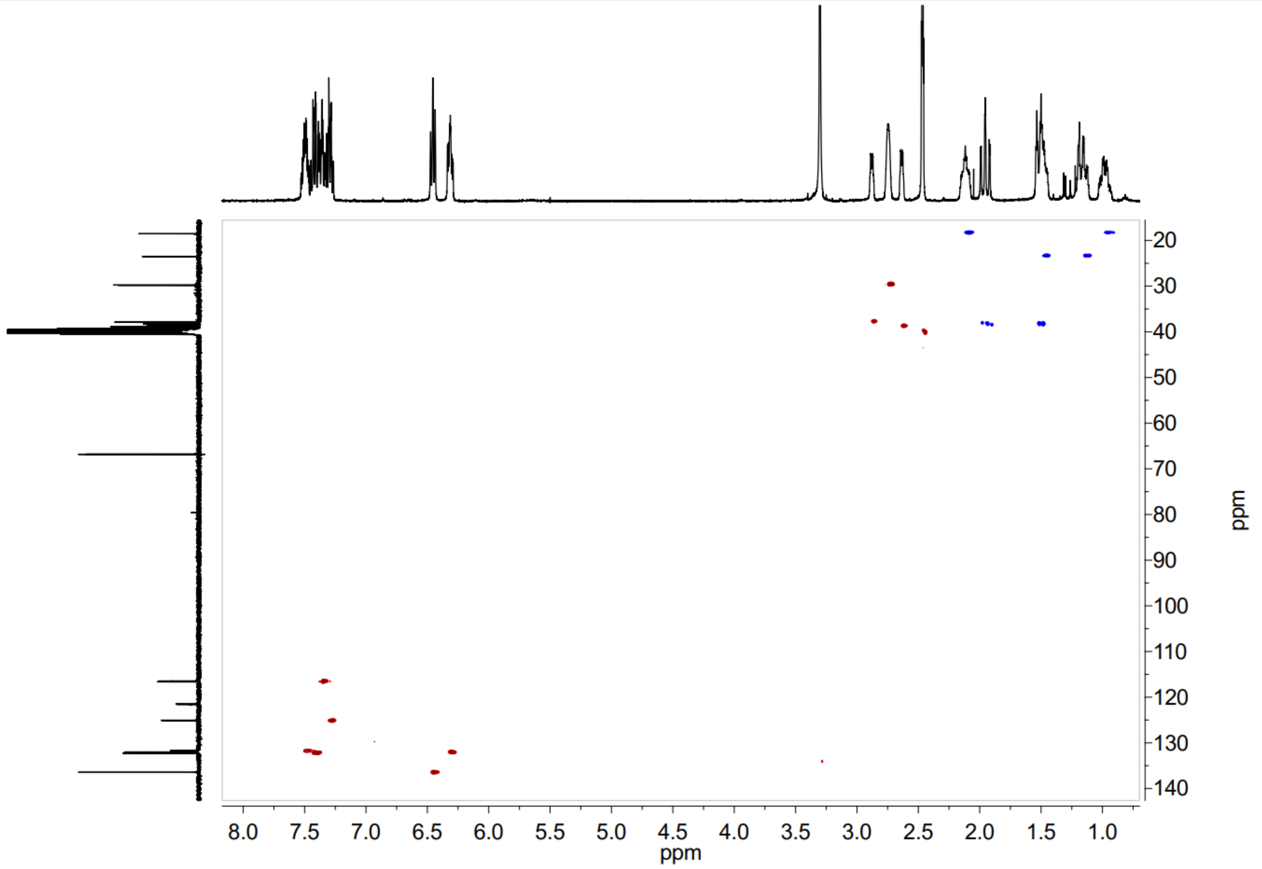

Figure S68. HSQC ^{1}H-^{13}C NMR spectra of compound **46a.**

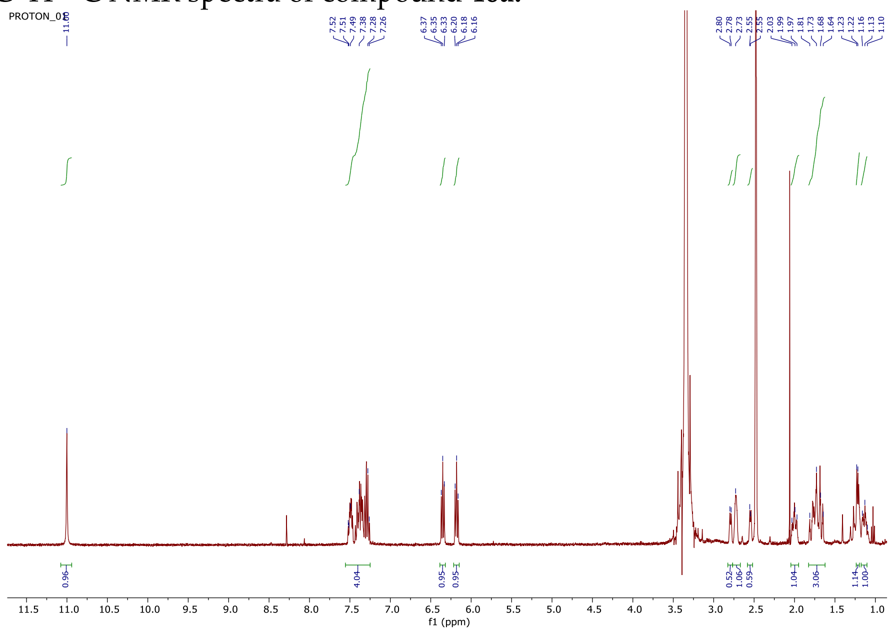

Figure S69. ^{1}H NMR spectra of compound **46b.**

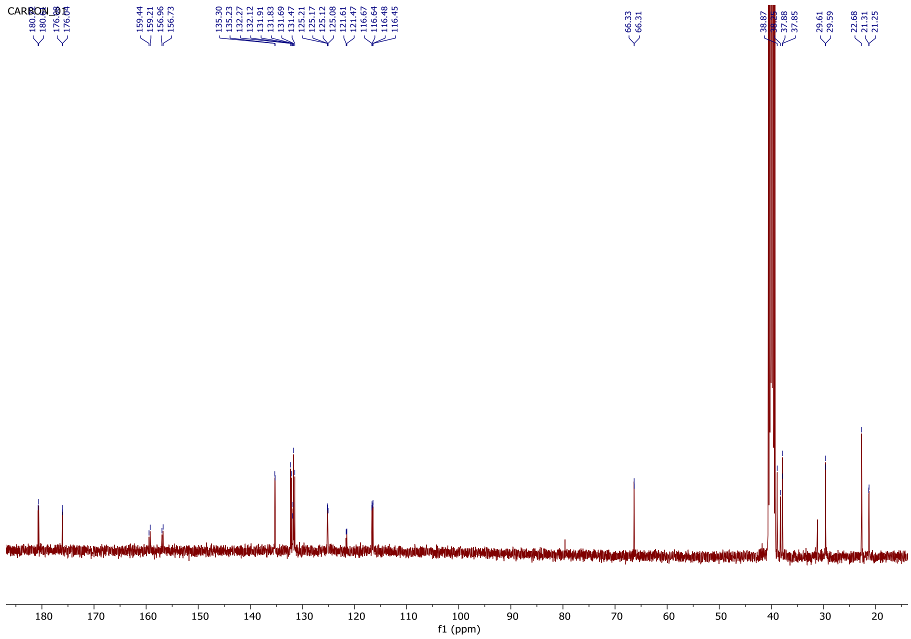

Figure S70. ^{13}C NMR spectra of compound **46b.**

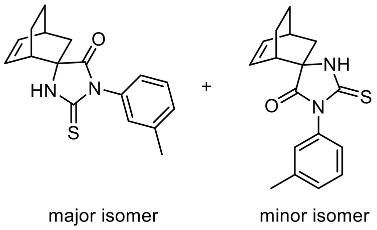

(1R,2R*,4R*)-2′-Thioxo-1′-(m-tolyl)spiro[bicyclo[2.2.2]oct[5]ene-2,4′-imidazolidin]-5′-one* (**47a**) (isolated using chloroform as eluent) and *(1S*,2R*,4S*)-2′-thioxo-1′-(m-tolyl)spiro[bicyclo[2.2.2]oct[5]ene-2,4′-imidazolidin]-5′-one* (**47b**) (isolated using methanol/chloroform (1:600) as eluent). From methylidenethiohydantoin **5** (107 mg, 0.49 mmol), ZnI_2 (156 mg, 0.49 mmol) and 1,3-cyclohexadiene (196 mg, 2.45 mmol) compound **47a** (88 mg, 60%) and compound **47b** (37 mg, 25%) were obtained as a white or pale yellow crystalline solids.

Major isomer 47a: ^{1}**H NMR** (400 MHz, DMSO-d6): δ 10.39 (bs, 1H), 7.35-7.31 (m, 1H), 7.23-7.21 (m, 1H), 7.09-7.05 (m, 2H), 6.48-6.44 (m, 1H), 6.33-6.30 (m, 1H), 2.82-2.75 (m, 2H), 2.32 (s, 3H), 2.18-2.13 (m, 1H), 1.96 (d, J = 13.7 Hz, 1H), 1.52-1.49 (m, 2H), 1.28-1.24 (m, 1H), 1.00-0.95 (m, 1H). ^{13}**C NMR** (101 MHz, DMSO-d6): 180.8, 176.5, 138.6, 136.4, 133.9, 132.2, 129.8, 129.7, 128.9, 126.5, 66.4, 38.7, 38.2, 29.9, 23.6, 21.2, 18.5. **HRMS** (ESI+) m/z calcd. for ($C_{17}H_{19}N_2OS$, M+H): 299.1213, found: (M+H): 299.1212.

Minor isomer 47b: ^{1}**H NMR** (400 MHz, DMSO-d6): δ 10.84 (bs, 1H), 7.34-7.31 (m, 1H), 7.22-7.20 (m, 1H), 7.07-7.03 (m, 2H), 6.36-6.32 (m, 1H), 6.18-6.16 (m, 1H), 2.72-2.69 (m, 2H), 2.31 (s, 3H), 2.03-1.98 (m, 1H), 1.80-1.71 (m, 2H), 1.66-1.63 (m, 1H), 1.28-1.24 (m, 1H), 1.15-1.08 (m, 1H). ^{13}**C NMR** (101 MHz, DMSO-d6): 181.4, 176.5, 138.5, 135.1, 133.9, 131.8, 129.8, 129.6, 128.9, 126.5, 65.9, 38.2, 29.7, 22.7, 21.3, 21.2. **HRMS** (ESI+) m/z calcd. for ($C_{17}H_{19}N_2OS$, M+H): 299.1213, found: (M+H): 299.1213.

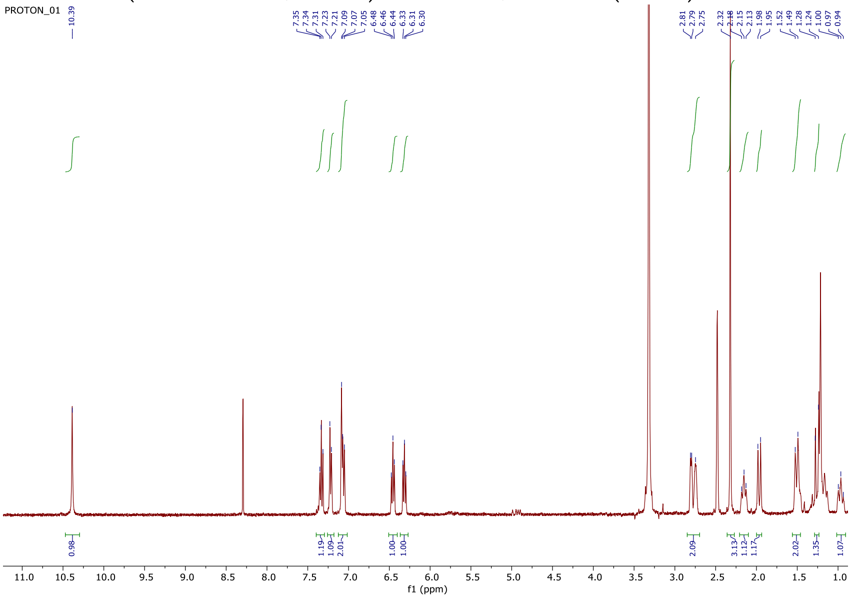

Figure S71. ^{1}H NMR spectra of compound **47a.**

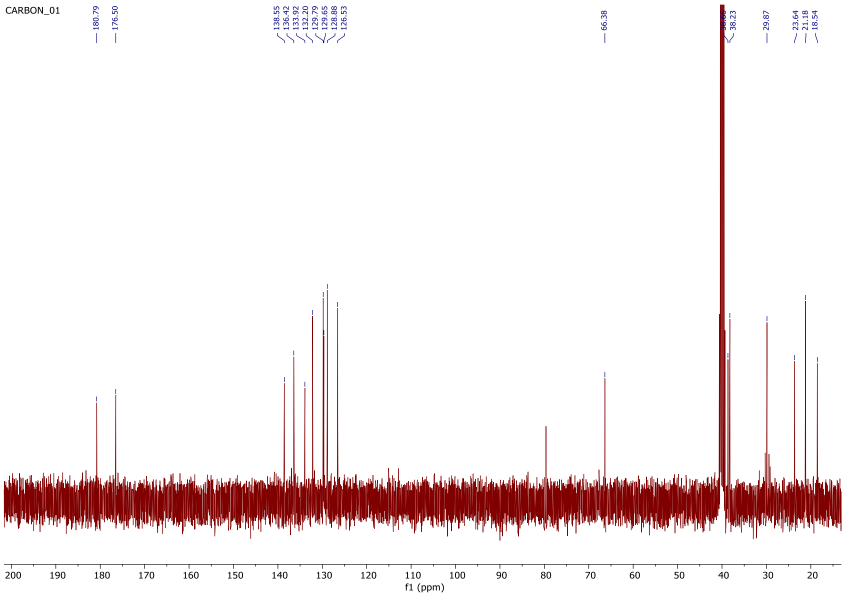

Figure S72. ^{13}C NMR spectra of compound **47a.**

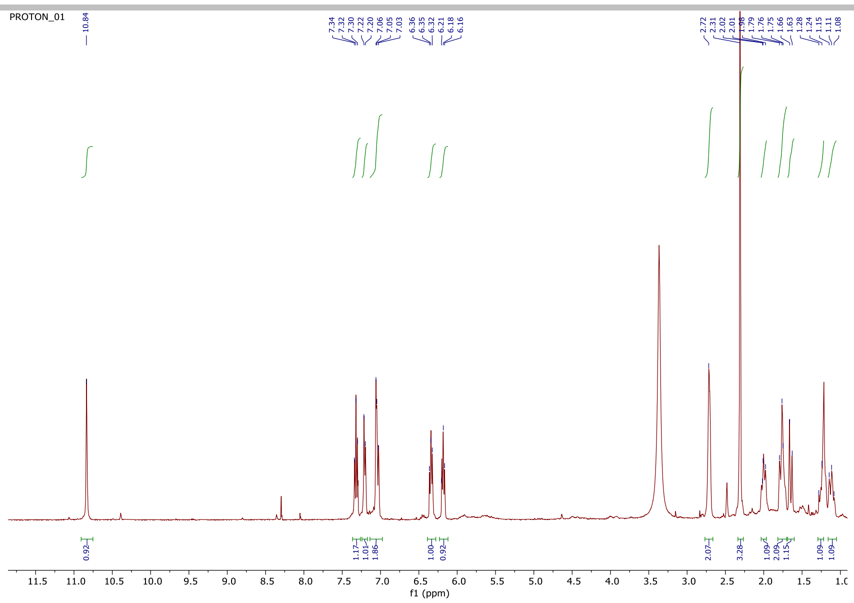

Figure S73. ^{1}H NMR spectra of compound **47b.**

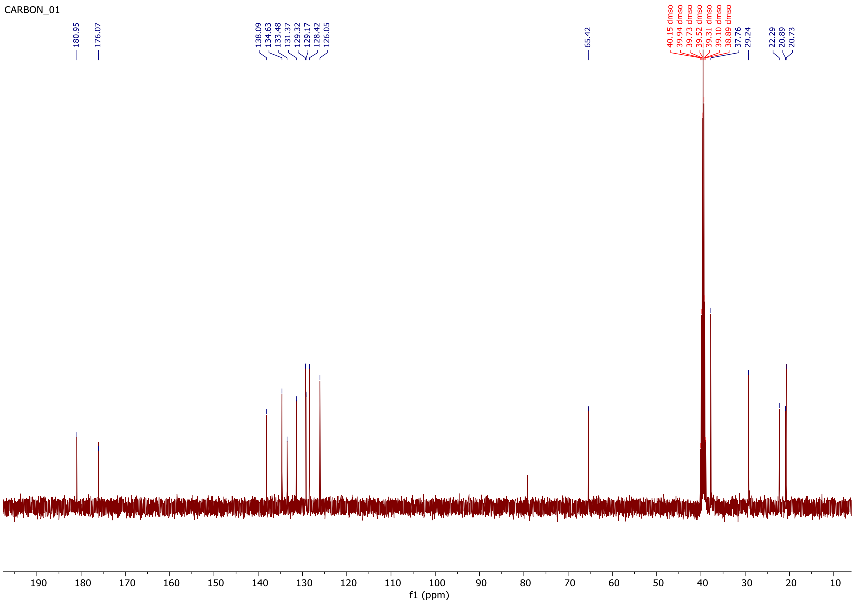

Figure S74. ^{13}C NMR spectra of compound **47b.**

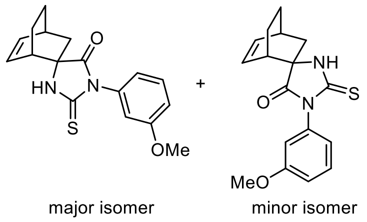

(1R,2R*,4R*)-1′-(3-Methoxyphenyl)-2′-thioxospiro[bicyclo[2.2.2]oct[5]ene-2,4′-imidazolidin]-5′-one* (**48a**) (isolated using chloroform as eluent) and *(1S*,2R*,4S*)-1′-(3-methoxyphenyl)-2′-thioxospiro[bicyclo[2.2.2]oct[5]ene-2,4′-imidazolidin]-5′-one* (**48b**) (isolated using methanol/chloroform (1:600) as eluent). From methylidenethiohydantoin **6** (115 mg, 0.49 mmol), ZnI_2 (156 mg, 0.49 mmol) and 1,3-cyclohexadiene (196 mg, 2.45 mmol) compound **48a** (78 mg, 51%) and compound **48b** (32 mg, 21%) were obtained as a white or pale yellow crystalline solids.

Major isomer 48a: ^{1}H NMR (400 MHz, $CDCl_3$): δ 7.67 (bs, 1H), 7.42-7.38 (m, 1H), 6.99 (dd, J_1 = 2.4 Hz, J_2 = 8.4 Hz, 1H), 6.92-6.90 (m, 1H) 6.86 (s, 1H), 6.60-6.56 (m, 1H), 6.38-6.35 (m, 1H), 3.83 (s, 3H), 2.84 (s, 2H), 2.37-2.32 (m, 1H), 2.19 (d, J = 12.5 Hz, 1H), 1.71-1.64 (m, 2H), 1.34-1.29 (m, 1H), 1.19-1.12 (m, 1H). **^{13}C NMR** (101 MHz, $CDCl_3$): 180.8, 175.2, 159.8, 137.4, 133.4, 130.8, 129.3, 120.1, 114.7, 113.7, 66.2, 55.0,

38.6, 38.1, 29.6, 23.3, 17.4. **HRMS** (ESI+) m/z calcd. for ($C_{17}H_{19}N_2O_2S$, M+H): 315.1162, found: (M+H): 315.1162.

Minor isomer 48b: ^{1}**H NMR** (400 MHz, $CDCl_3$): δ 8.93 (bs, 1H), 7.41-7.37 (m, 1H), 6.99-6.96 (m, 1H), 6.91-6.85 (m, 1H),6.86-6.83 (m, 1H), 6.47-6.43 (m, 1H), 6.33-6.30 (m, 1H), 3.83 (s, 3H), 2.82-2.76 (m, 2H), 2.05-1.96 (m, 2H), 1.80-1.76 (m, 1H), 1.68-1.62 (m, 1H), 1.43-1.26 (m, 2H). ^{13}**C NMR** (101 MHz, $CDCl_3$): 181.6, 175.4, 159.5, 134.8, 133.4, 130.4, 129.3, 120.1, 114.7, 113.7, 65.7, 55.0, 38.4, 37.8, 29.2, 22.4, 21.1. **HRMS** (ESI+) m/z calcd. for ($C_{17}H_{19}N_2O_2S$, M+H): 315.1162, found: (M+H): 315.1162.

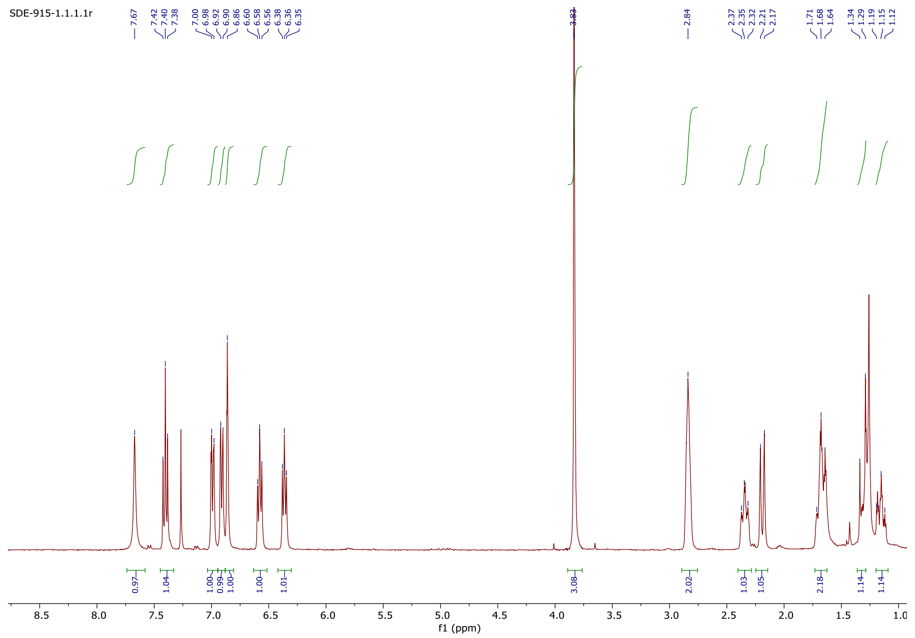

Figure S75. ^{1}H NMR spectra of compound **48a.**

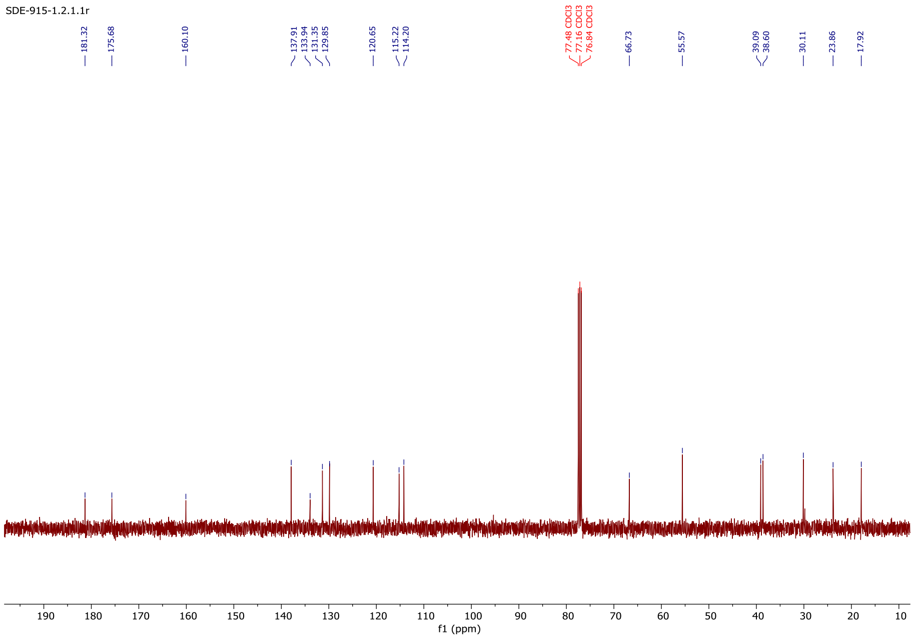

Figure S76. ^{13}C NMR spectra of compound **48a.**

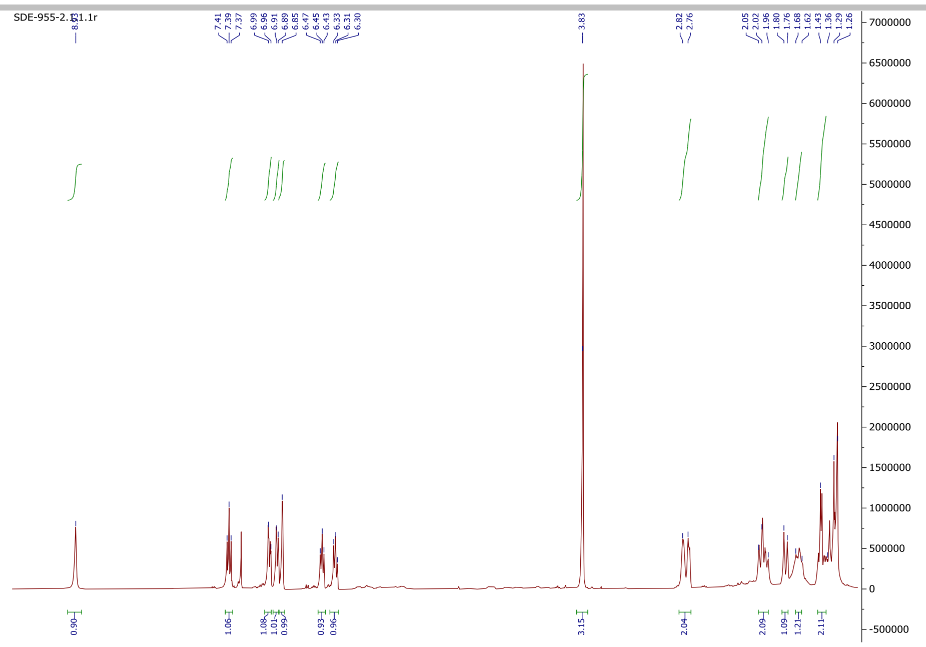

Figure S77. ^{1}H NMR spectra of compound **48b.**

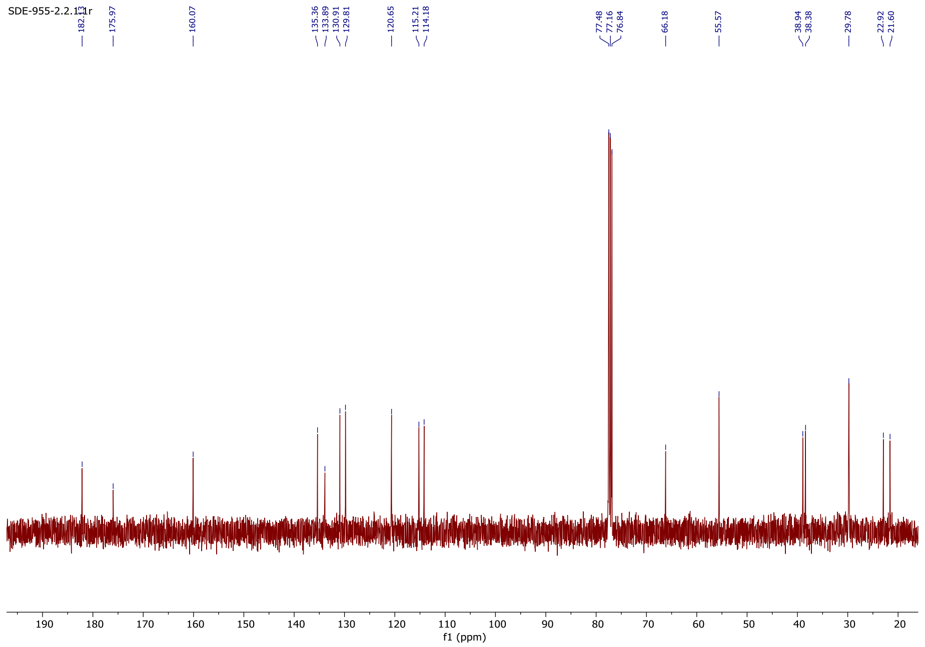

Figure S78. ^{13}C NMR spectra of compound **48b.**

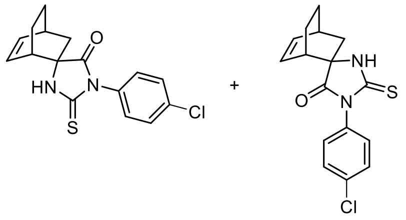

major isomer minor isomer

(1R,2R*,4R*)-1′-(4-Chlorophenyl)-2′-thioxospiro[bicyclo[2.2.2]oct[5]ene-2,4′-imidazolidin]-5′-one* (**49a**) (isolated using chloroform as eluent) and *(1S*,2R*,4S*)-1′-(4-chlorophenyl)-2′-thioxospiro[bicyclo[2.2.2]oct[5]ene-2,4′-imidazolidin]-5′-one* (**49b**) (isolated using methanol/chloroform (1:600) as eluent). From methylidenethiohydantoin **8** (117 mg, 0.49 mmol), ZnI_2 (156 mg, 0.49 mmol) and 1,3-cyclohexadiene (196 mg, 2.45 mmol) compound **49a** (86 mg, 55%) and compound **49b** (31 mg, 20%) were obtained as a white or pale yellow crystalline solids.

Major isomer 49a: ^{1}**H NMR** (400 MHz, DMSO-d6): δ 10.46 (bs, 1H), 7.54-7.51 (m, 2H), 7.36-7.34 (m, 2H), 6.47-6.43 (m, 1H), 6.32-6.29 (m, 1H), 2.86-2.84 (m, 1H), 2.76-2.74 (m, 1H), 2.16-2.13 (m, 1H), 1.98 (d, J = 11.9 Hz, 1H), 1.52-1.48 (m, 2H), 1.19-1.13 (m, 1H), 1.00-0.94 (m, 1H). ^{13}**C NMR** (101 MHz, DMSO-d6):

180.3, 176.3, 136.5, 133.6, 132.9, 132.1, 131.4 (2C), 129.1 (2C), 66.5, 38.7, 38.1, 29.9, 23.6, 18.5. **HRMS** (ESI+) m/z calcd. for ($C_{16}H_{16}ClN_2OS$, M+H): 319.0666, found: (M+H): 319.0664.

Minor isomer 49b: ^{1}**H NMR** (400 MHz, DMSO-d6): δ 10.90 (bs, 1H), 7.52-7.50 (m, 2H), 7.33-7.30 (m, 2H), 6.36-6.32 (m, 1H), 6.19-6.15 (m, 1H), 2.75-2.70 (m, 2H), 2.02-1.96 (m, 1H), 1.80-1.73 (m, 2H), 1.66-1.63 (m, 1H), 1.27-1.23 (m, 1H), 1.14-1.08 (m, 1H). ^{13}**C NMR** (101 MHz, DMSO-d6): 180.9, 176.3, 135.2, 133.4, 132.9, 131.8, 131.3 (2C), 129.1 (2C), 66.0, 38.2, 38.1, 29.7, 22.7, 21.3. **HRMS** (ESI+) m/z calcd. for ($C_{16}H_{16}ClN_2OS$, M+H): 319.0666, found: (M+H): 319.0663.

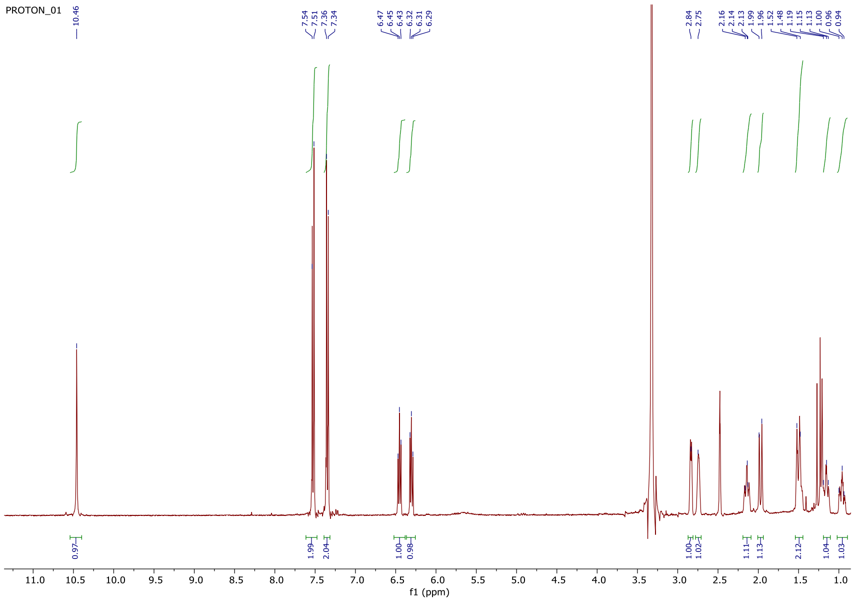

Figure S79. ^{1}H NMR spectra of compound **49a.**

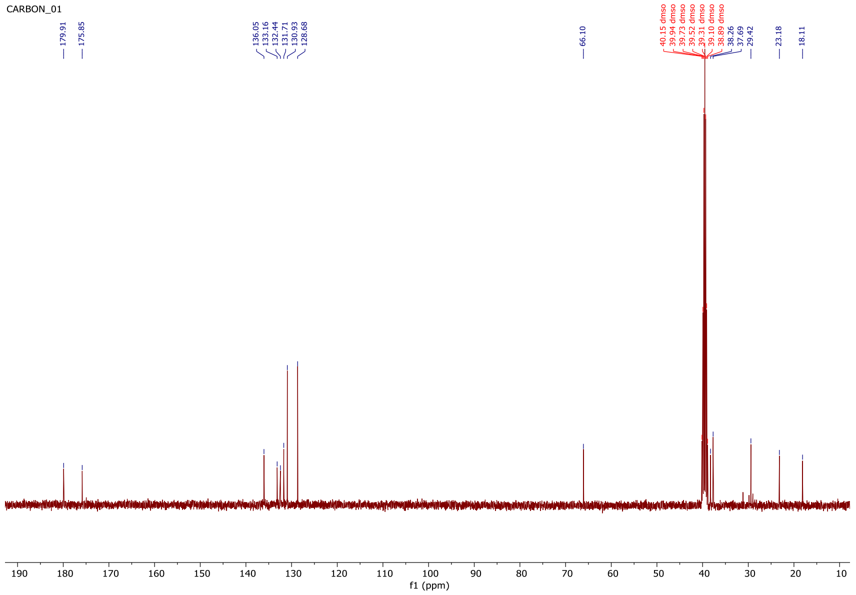

Figure S80. ^{13}C NMR spectra of compound **49a.**

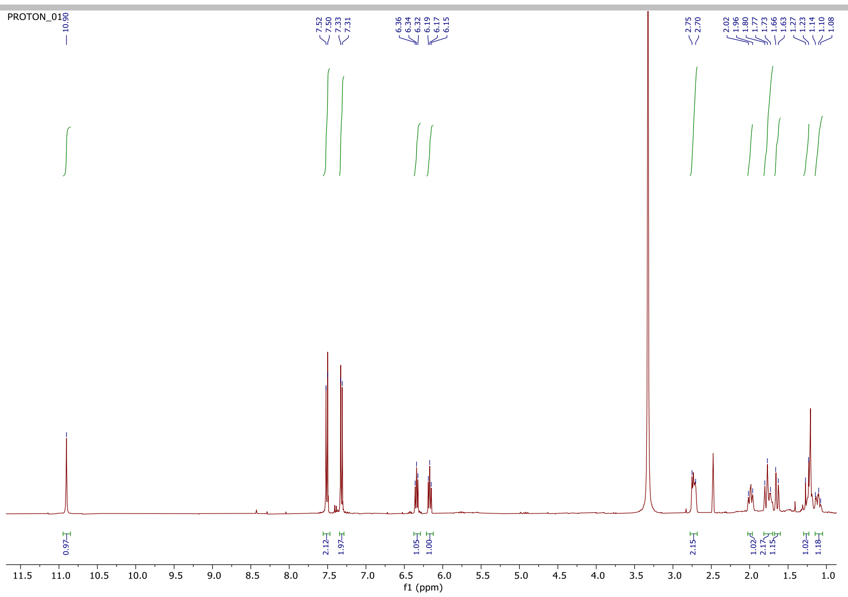

Figure S81. ^{1}H NMR spectra of compound **49b.**

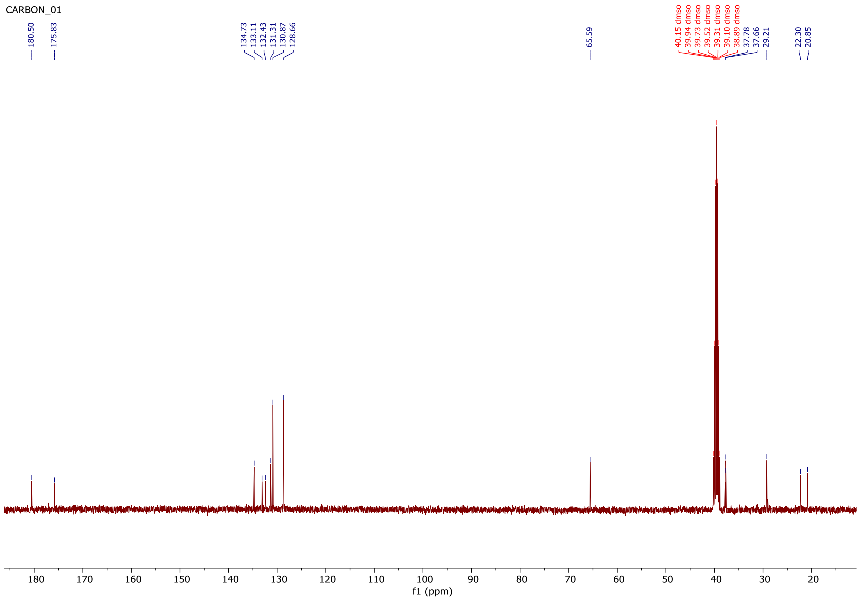

Figure S82. ^{13}C NMR spectra of compound **49b.**

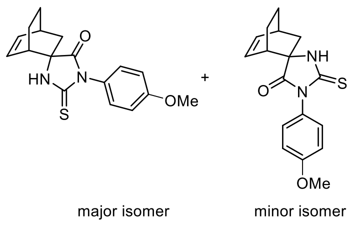

(1R,2R*,4R*)-1′-(4-Methoxyphenyl)-2′-thioxospiro[bicyclo[2.2.2]oct[5]ene-2,4′-imidazolidin]-5′-one* (**50a**) (isolated using chloroform as eluent) and *(1S*,2R*,4S*)-1′-(4-methoxyphenyl)-2′-thioxospiro[bicyclo[2.2.2]oct[5]ene-2,4′-imidazolidin]-5′-one* (**50b**) (isolated using methanol/chloroform (1:600) as eluent). From methylidenethiohydantoin **9** (115 mg, 0.49 mmol), ZnI_2 (156 mg, 0.49 mmol) and 1,3-cyclohexadiene (196 mg, 2.45 mmol) compound **50a** (77 mg, 50%) and compound **50b** (29 mg, 19%) were obtained as a white or pale yellow crystalline solids.

Major isomer 50a: **^{1}H NMR** (400 MHz, $CDCl_3$): δ 7.67 (bs, 1H), 7.24-7.22 (m, 2H), 7.01-6.99 (m, 2H), 6.60-6.56 (m, 1H), 6.38-6.35 (m, 1H), 3.84 (s, 3H), 2.84-2.82 (m, 2H), 2.38-2.31 (m, 1H), 2.18 (d, J = 13.7 Hz, 1H), 1.71-1.63 (m, 2H), 1.37-1.34 (m, 1H), 1.19-1.12 (m, 1H). **^{13}C NMR** (101 MHz, $CDCl_3$): 181.3, 175.4, 159.4, 137.4, 130.8, 129.0 (2C), 125.0, 113.0 (2C), 66.1, 55.1, 38.6, 38.1, 29.6, 23.3, 17.4. **HRMS** (ESI+) m/z calcd. for ($C_{17}H_{19}N_2O_2S$, M+H): 315.1162, found: (M+H): 315.1159.

Minor isomer 50b: [1]**H NMR** (400 MHz, $CDCl_3$): δ 8.57 (bs, 1H), 7.23-7.21 (m, 2H), 7.00-6.98 (m, 2H), 6.47-6.43 (m, 1H), 6.34-6.30 (m, 1H), 3.84 (s, 3H), 2.82-2.76 (m, 1H), 2.75-2.74 (m, 1H), 2.05-1.94 (m, 2H), 1.79-1.75 (m, 1H), 1.66-1.63 (m, 1H), 1.42-1.34 (m, 2H). [13]**C NMR** (101 MHz, $CDCl_3$): 182.1, 175.8, 159.4, 134.8, 130.4, 129.0 (2C), 125.0, 113.9 (2C), 65.5, 38.5, 37.8, 29.3, 22.3, 21.0. **HRMS** (ESI+) m/z calcd. for ($C_{17}H_{19}N_2O_2S$, M+H): 315.1162, found: (M+H): 315.1159.

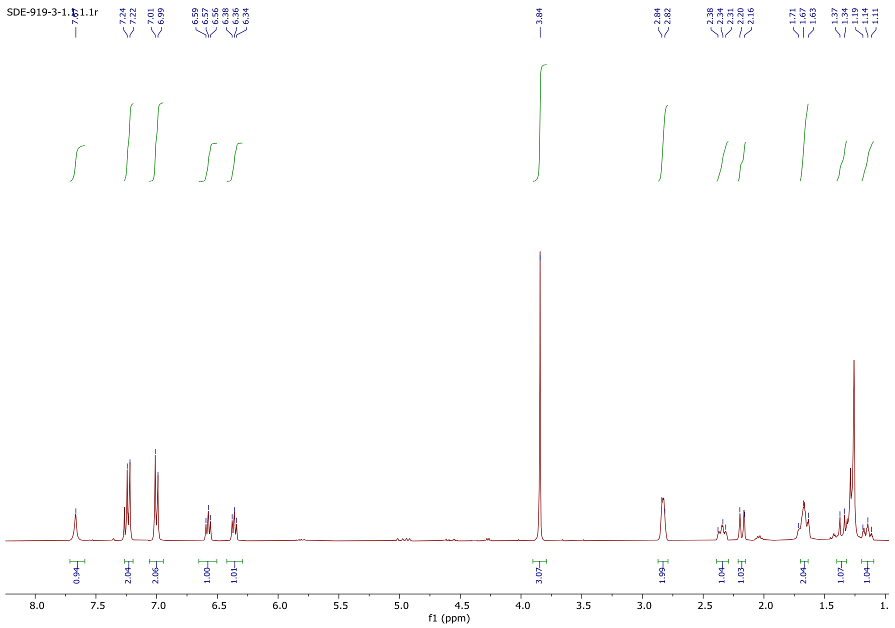

Figure S83. ^{1}H NMR spectra of compound **50a.**

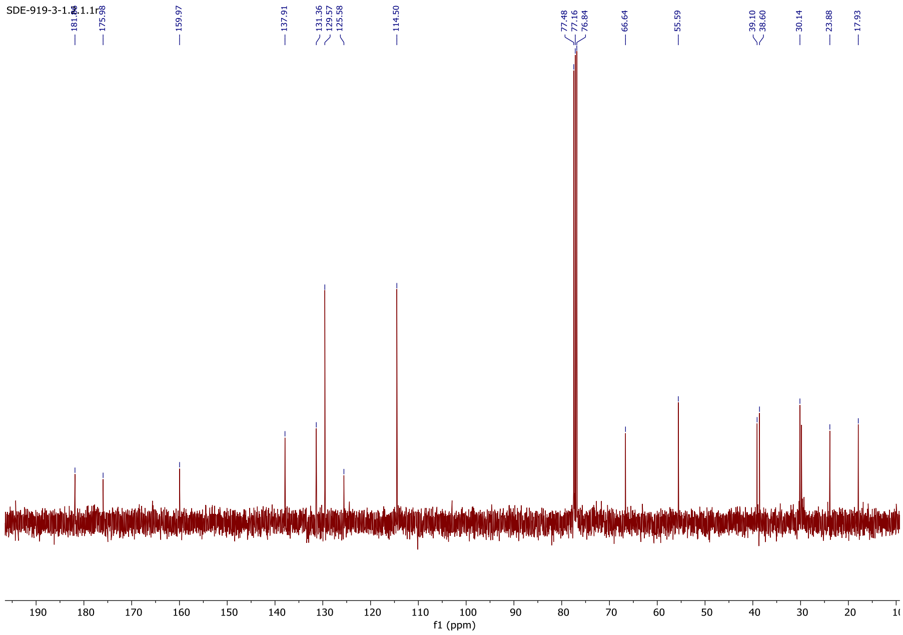

Figure S84. ^{13}C NMR spectra of compound **50a.**

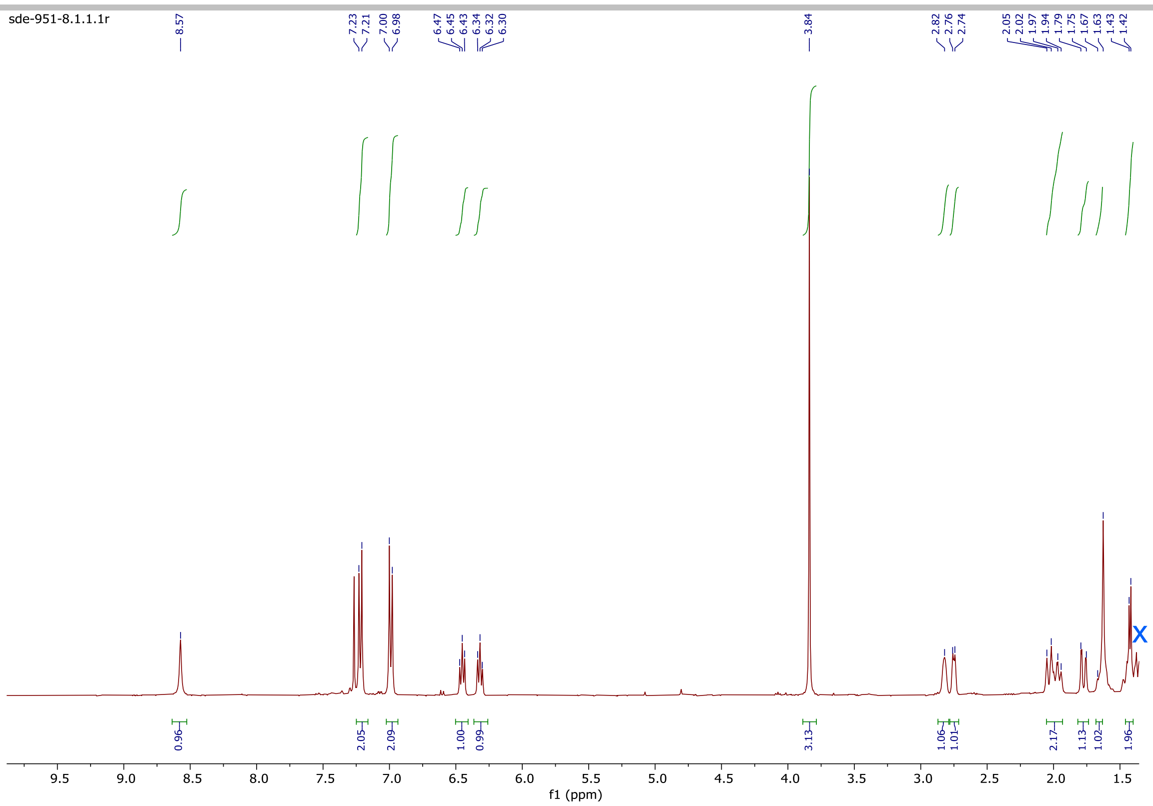

Figure S85. ^{1}H NMR spectra of compound **50b** (*the sign "x" marks the residual signals of tar*).

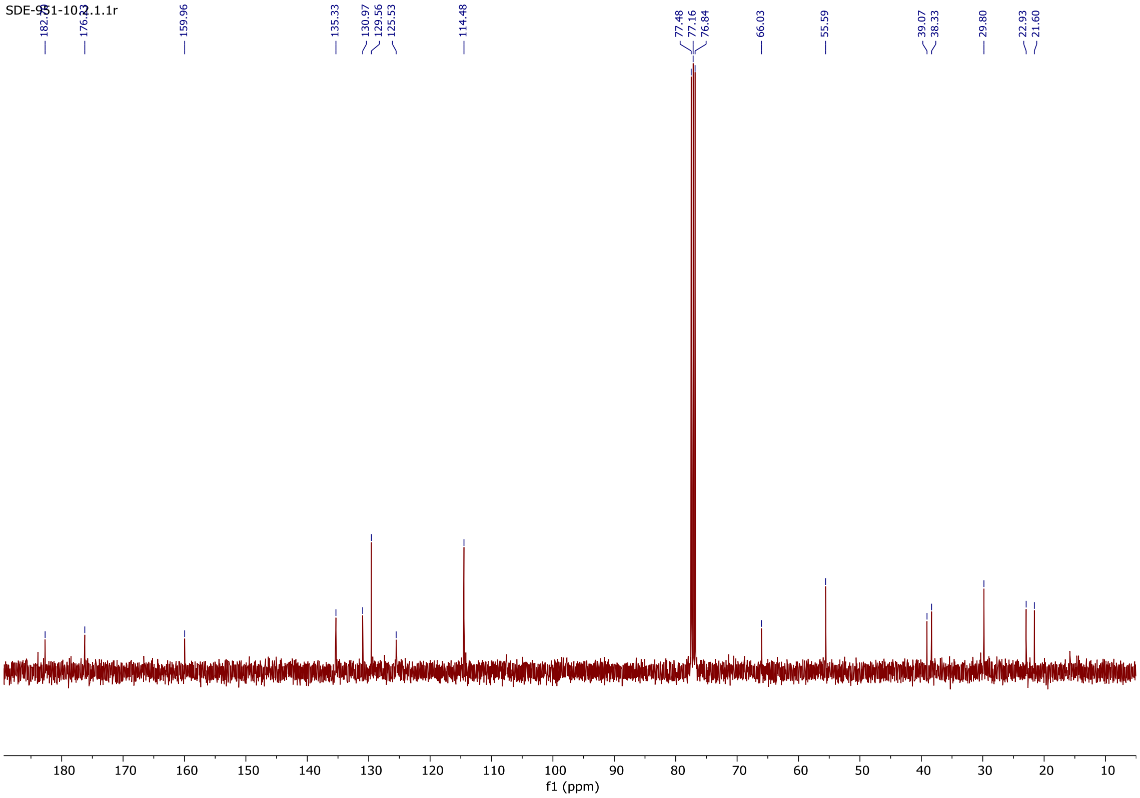

Figure S86. ^{13}C NMR spectra of compound **50b.**

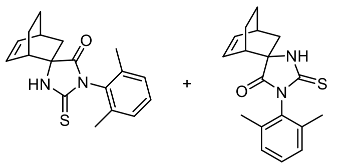

major isomer minor isomer

(1R,2R*,4R*)-1′-(2,6-Dimethylphenyl)-2′-thioxospiro[bicyclo[2.2.2]oct[5]ene-2,4′-imidazolidin]-5′-one* (**51a**) (isolated using chloroform as eluent) and *(1S*,2R*,4S*)-1′-(2,6-dimethylphenyl)-2′-thioxospiro[bicyclo[2.2.2]oct[5]ene-2,4′-*

imidazolidin]-5′-one (**51b**) (isolated using methanol/chloroform (1:600) as eluent). From methylidenethiohydantoin **10** (114 mg, 0.49 mmol), ZnI_2 (156 mg, 0.49 mmol) and 1,3-cyclohexadiene (196 mg, 2.45 mmol) compound **51a** (67 mg, 44%) and compound **51b** (22 mg, 15%) were obtained as a white or pale yellow crystalline solids.

Major isomer 51a: **^{1}H NMR** (400 MHz, $CDCl_3$): δ ^{1}H NMR (400 MHz, $CDCl_3$) δ 8.38 (bs, 1H), 7.30-7.25 (m, 1H), 7.19-7.16 (m, 2H), 6.57-6.51 (m, 1H), 6.40-6.34 (m, 1H), 2.84-2.79 (m, 2H), 2.39-2.34 (m, 1H), 2.21 (s, 3H), 2.20-2.15 (m, 1H), 2.12 (s, 3H), 1.72-1.65 (m, 2H), 1.31-1.25 (m, 1H), 1.18-1.12 (m, 1H). **^{13}C NMR** (101 MHz, $CDCl_3$): δ 180.2, 175.1, 137.2, 136.4, 136.1, 131.0, 130.7, 129.3, 128.1, 128.0, 66.5, 38.4, 38.3, 29.6, 23.3, 17.5, 17.3. **HRMS** (ESI+) m/z calcd. for ($C_{18}H_{21}N_2OS$, M+H): 313.1369, found: (M+H): 313.1370.

Minor isomer 51b: **^{1}H NMR** (400 MHz, $CDCl_3$): δ 9.38 (bs, 1H), 7.28-7.25 (m, 1H), 7.18-7.15 (m, 2H), 6.47-6.43 (m, 1H), 6.35-6.32 (m, 1H), 2.84-2.82 (m, 1H), 2.76-2.74 (m, 1H), 2.21 (s, 3H), 2.10 (s, 3H), 2.08-2.00 (m, 2H), 1.82-1.78 (m, 1H), 1.68-1.61 (m, 1H), 1.43-1.27 (m, 2H). **^{13}C NMR** (101 MHz, $CDCl_3$): δ 181.1, 175.3, 136.5, 136.1, 134.8, 130.5, 130.4, 129.2, 128.0, 66.1, 38.2, 38.1, 29.2, 22.5, 21.2, 17.5, 17.3. **HRMS** (ESI+) m/z calcd. for ($C_{18}H_{21}N_2OS$, M+H): 313.1369, found: (M+H): 313.1367.

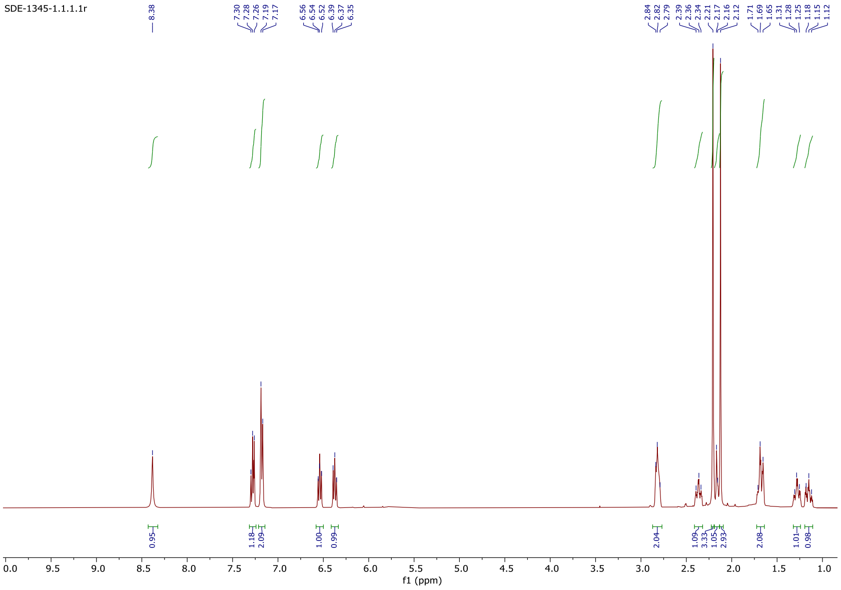

Figure S87. ^{1}H NMR spectra of compound **51a.**

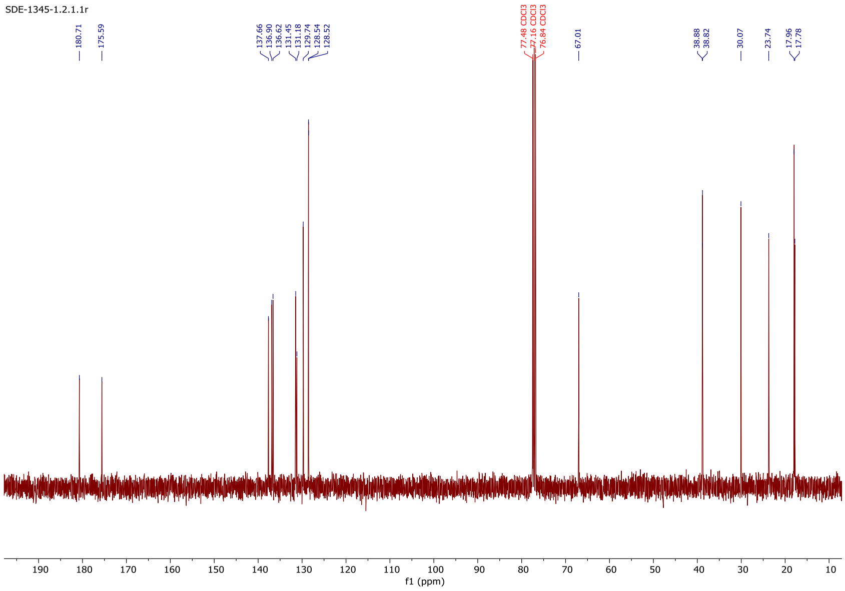

Figure S88. ^{13}C NMR spectra of compound **51a.**

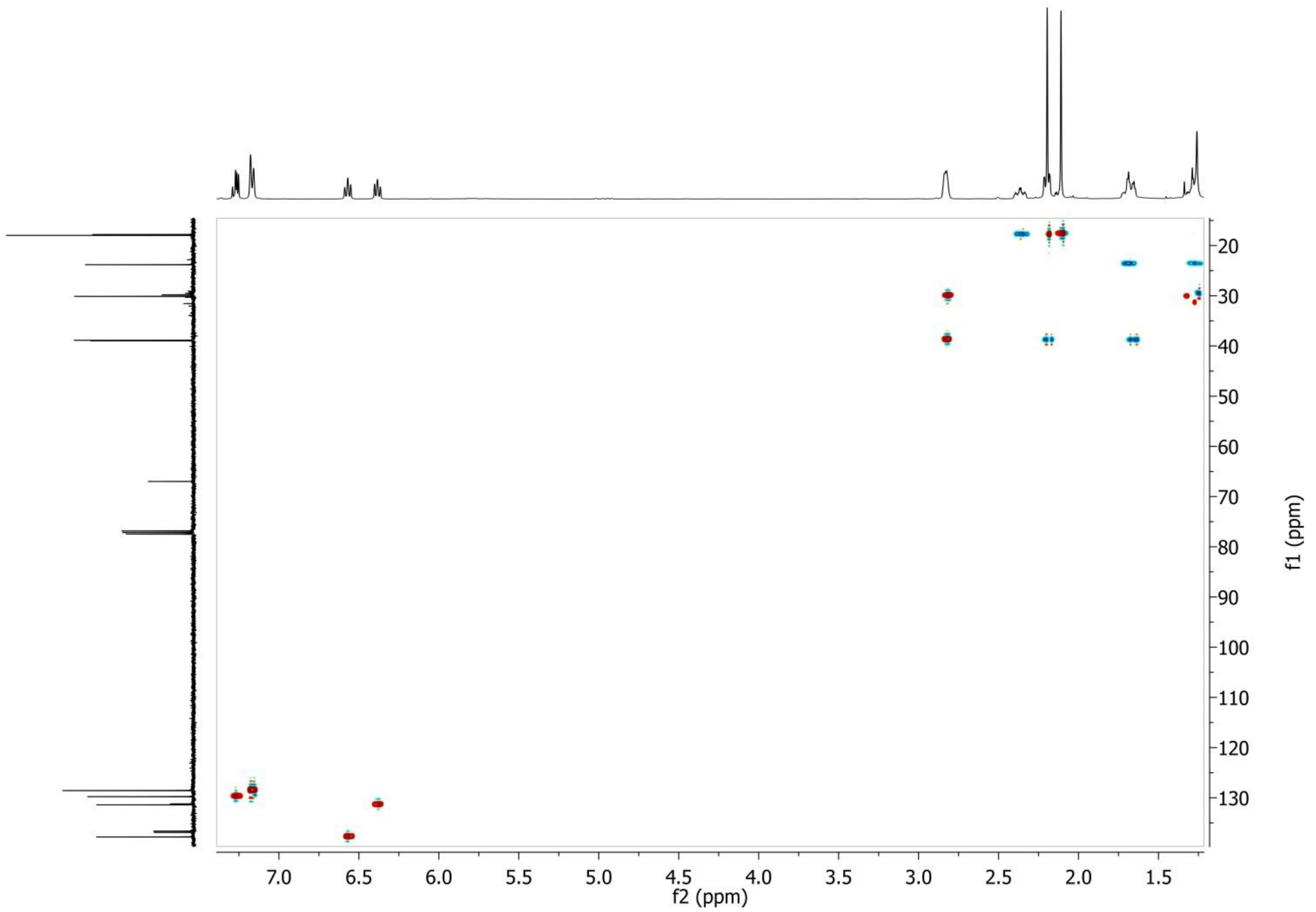

Figure S89. HSQC ^{1}H-^{13}C NMR spectra of of compound **51a.**

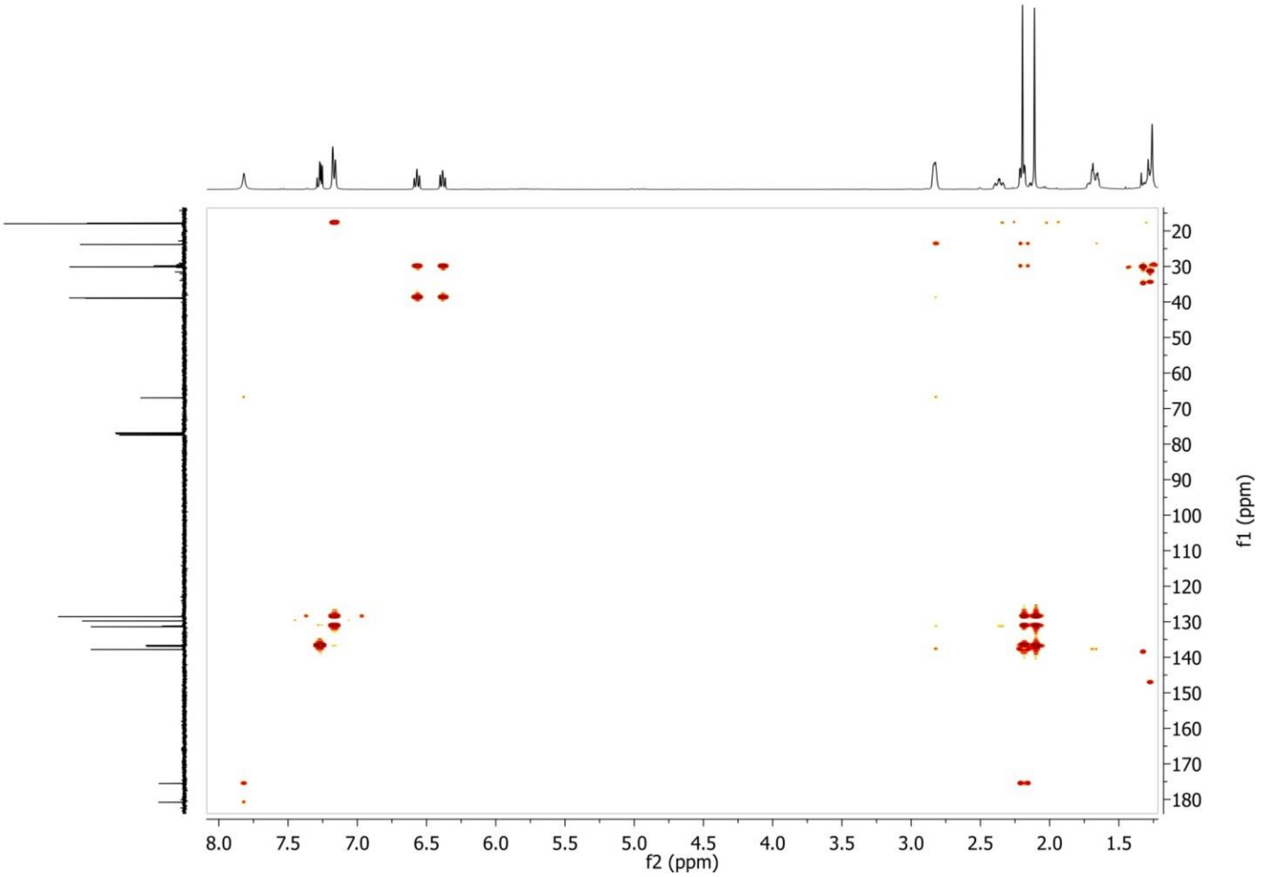

Figure S90. HMBC ^{1}H-^{13}C NMR spectra of of compound **51a.**

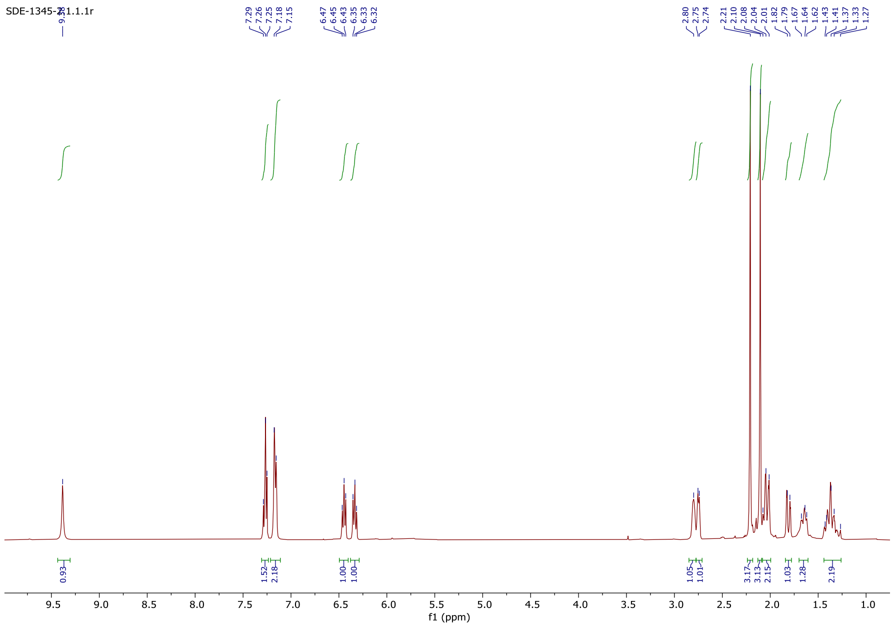

Figure S91. ^{1}H NMR spectra of compound **51b.**

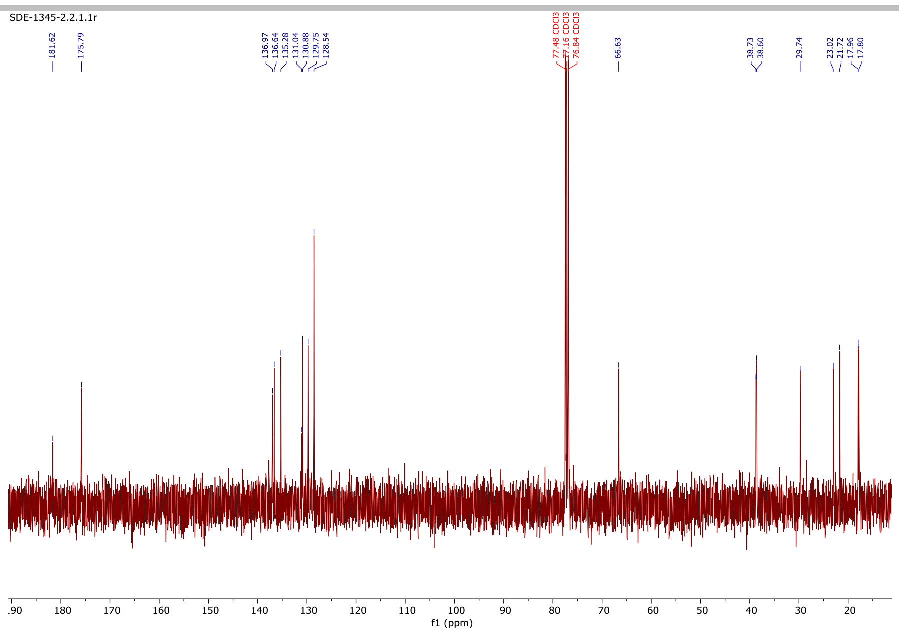

Figure S92. ^{13}C NMR spectra of compound **51b.**

General procedure of Diels-Alder reaction of methylideneimidazolones 1-12 with 2,3-dimethylbuta-1,3-diene.

To a solution of methylideneimidazolone (0.27 mmol) and $AlCl_3$ or ZnI_2 (0.27 mmol) in boiling chloroform (15 ml) 2,3-dimethylbuta-1,3-diene (108 mg, 1.35 mmol) was added 2,3-dimethylbuta-1,3-diene (108 mg, 1.35 mmol) and the mixture was refluxed for 6 h. The reaction was then cooled to room temperature and the mixture was washed with water (15 ml) and filtered. The organic phase was dried over anhydrous Na_2SO_4, filtered and evaporated under vacuum. The residue was purified by column chromatography on silica gel.

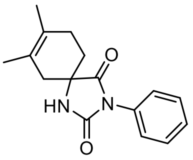

7,8-Dimethyl-3-phenyl-1,3-diazaspiro[4.5]dec-7-ene-2,4-dione (**52**) (isolated using methanol/chloroform (1:200) as eluent). From methylideneimidazolone **1** (51 mg, 0.27 mmol), $AlCl_3$ (36 mg, 0.27 mmol) and 2,3-dimethylbuta-1,3-diene (108 mg, 1.35 mmol) compound **52** (34 mg, 47%) was obtained as a pale yellow crystalline solid.

^{1}H NMR (400 MHz, $CDCl_3$): δ 7.48-7.42 (m, 4H), 7.38-7.34 (m, 1H), 6.46 (bs, 1H), 2.75 (d, J = 17.1 Hz, 1H), 2.20-2.18 (m, 2H), 2.12-2.06 (m, 1H), 2.03-1.98 (m, 1H), 1.83-1.80 (m, 1H), 1.66 (s, 6H). **^{13}C NMR** (101 MHz, $CDCl_3$): δ 175.3, 155.2, 131.3, 128.5 (2C), 127.6, 125.6 (2C), 124.9, 121.5, 60.3, 39.2, 29.4, 27.3, 18.7, 18.3. **HRMS** (ESI+) m/z calcd. for ($C_{16}H_{19}N_2O_2$, M+H): 271.1441, found: (M+H): 271.1439.

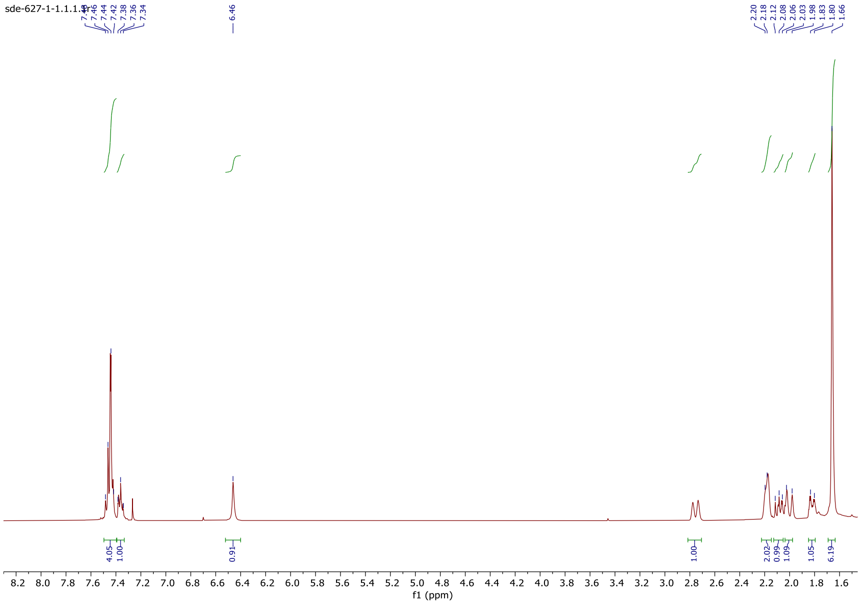

Figure S93. ^{1}H NMR spectra of compound **52.**

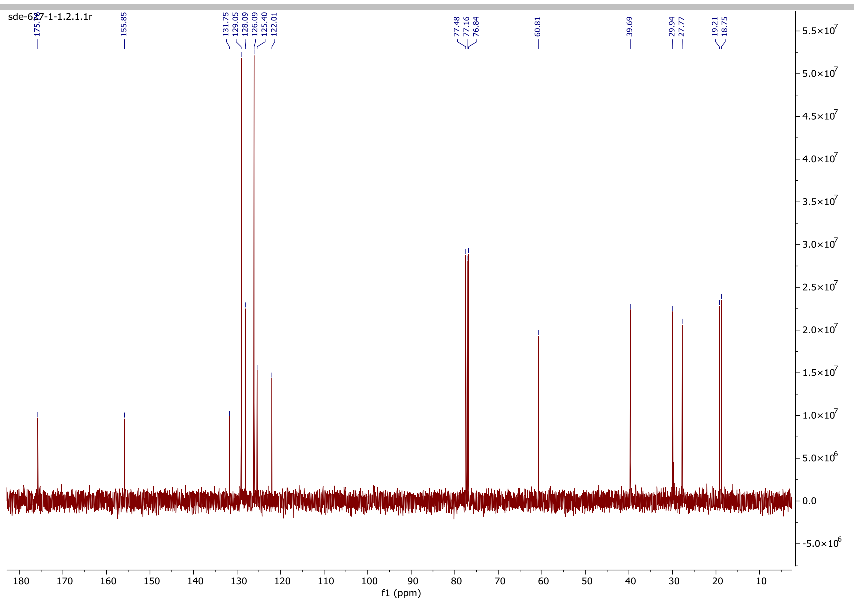

Figure S94. ^{13}C NMR spectra of compound **52.**

1

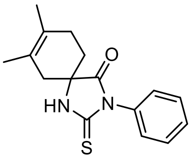

7,8-Dimethyl-3-phenyl-2-thioxo-1,3-diazaspiro[4.5]dec-7-en-4-one (**53**) (isolated using chloroform as eluent). From methylideneimidazolone **2** (55 mg, 0.27 mmol), ZnI_2 (86 mg, 0.27 mmol) and 2,3-dimethylbuta-1,3-diene (108 mg, 1.35 mmol) compound **53** (71 mg, 92%) was obtained as a white crystalline solid.

^{1}H NMR (400 MHz, $CDCl_3$): δ 7.75 (bs, 1H), 7.53-7.43 (m, 3H), 7.34-7.32 (m, 2H), 2.74 (d, J = 17.1 Hz, 1H), 2.24-2.22 (m, 2H), 2.11-2.05 (m, 2H), 1.93-1.89 (m, 1H), 1.70 (s, 6H). **^{13}C NMR** (101 MHz, $CDCl_3$): δ 182.1, 175.7, 132.3, 128.8 (2C), 128.7, 127.9 (2C), 125.1, 121.4, 63.3, 38.4, 29.1, 27.3, 18.7, 18.4. **HRMS** (ESI+) m/z calcd. for ($C_{16}H_{19}N_2OS$, M+H): 287.1213, found: (M+H): 287.1213.

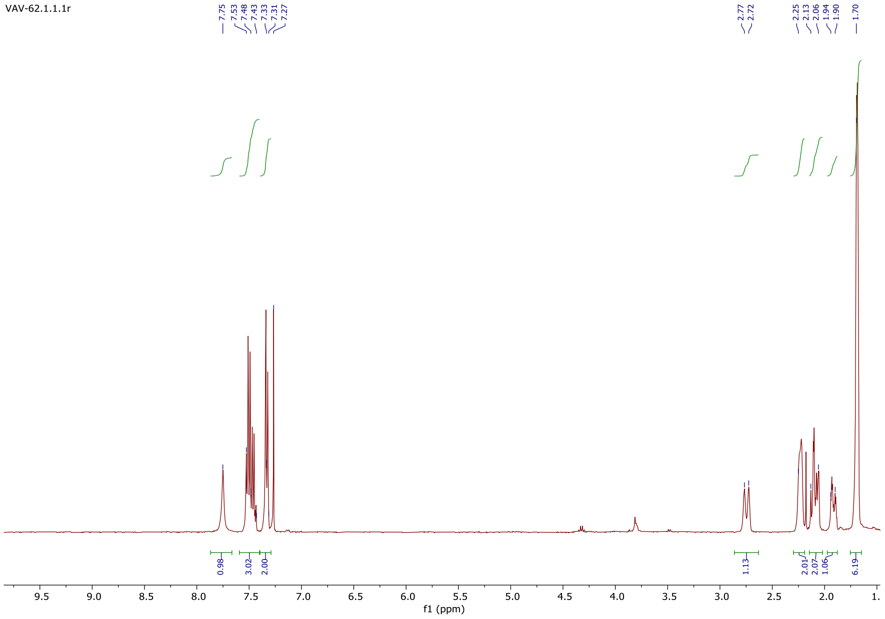

Figure S95. ^{1}H NMR spectra of compound **53.**

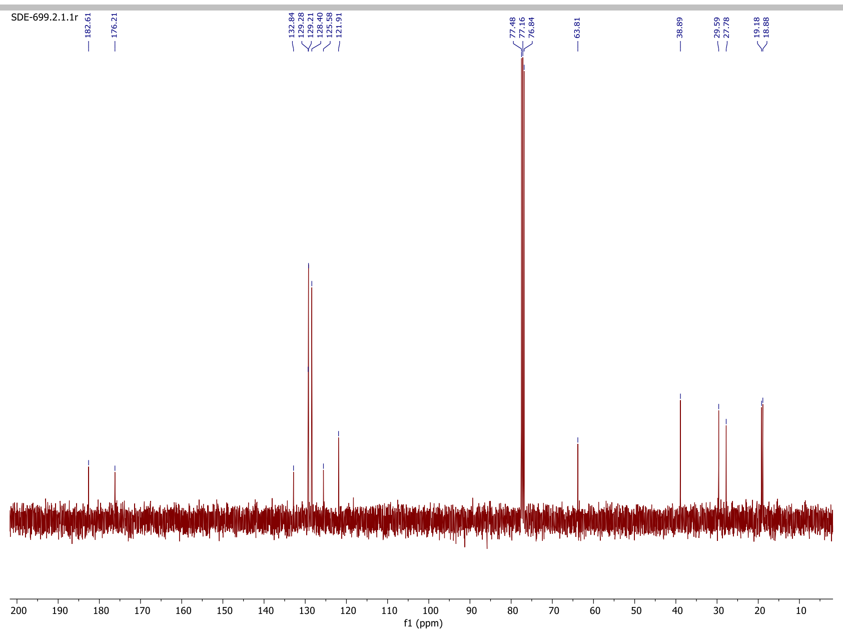

Figure S96. ^{13}C NMR spectra of compound **53.**

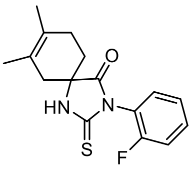

3-(2-Fuorophenyl)-7,8-dimethyl-2-thioxo-1,3-diazaspiro[4.5]dec-7-en-4-one (**54**) (isolated using chloroform as eluent). From methylideneimidazolone **3** (60 mg, 0.27 mmol), ZnI_2 (86 mg, 0.27 mmol) and 2,3-dimethylbuta-1,3-diene (108 mg, 1.35 mmol) compound **54** (69 mg, 84%) was obtained as a light yellow crystalline solid.

^{1}H NMR (400 MHz, $CDCl_3$): δ 7.97-7.96 (m, 1H), 7.49-7.46 (m, 1H), 7.36-7.22 (m, 3H), 2.74 (d, J = 17.1 Hz, 1H), 2.21 (s, 2H), 2.13-2.07 (m, 2H), 1.96-1.91 (m, 1H), 1.68 (s, 6H). **^{13}C NMR** (101 MHz, DMSO-d6): (two sets of signals for C(Ar)-N-rotamers) δ 181.2 (s), 181.1 (s), 175.6 (s, C+C′), 131.5 (d, J = 7.9 Hz, C+C′), 131.0 (s), 130.9 (s), 125.6 (s), 125.5 (s), 124.7 (s, C+C′), 121.9 (d, J = 14.6 Hz, C+C′), 120.8 (s, C+C′), 117.1 (d, J = 19.4 Hz, C+C′), 64.2 (s), 64.1 (s), 39.0 (s), 38.5 (s), 29.8 (s), 29.3 (s), 27.7 (s), 27.6 (s), 19.1 (s, C+C′), 18.8 (s, C+C′). **HRMS** (ESI+) m/z calcd. for ($C_{16}H_{16}FN_2OS$, M+H): 305.1118, found: (M+H): 305.1120.

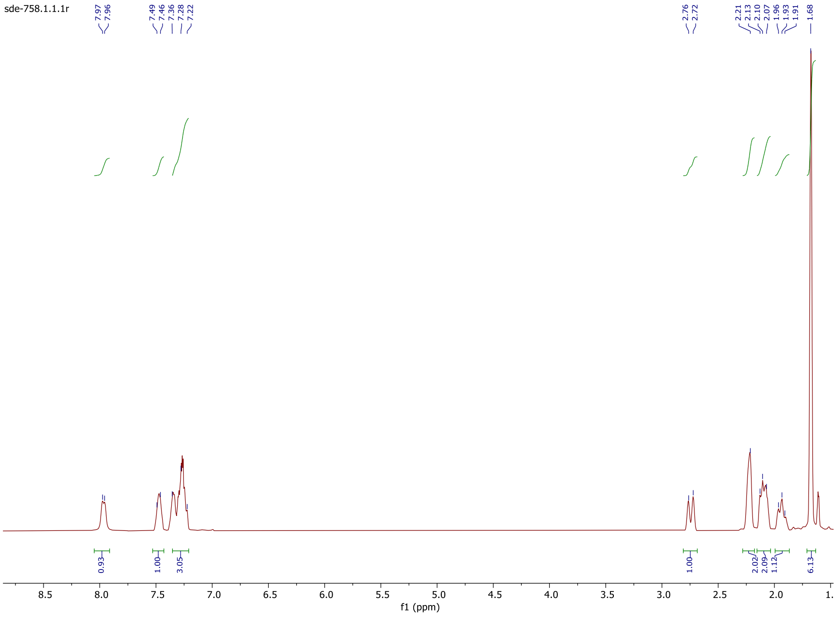

Figure S97. ^{1}H NMR spectra of compound **54.**

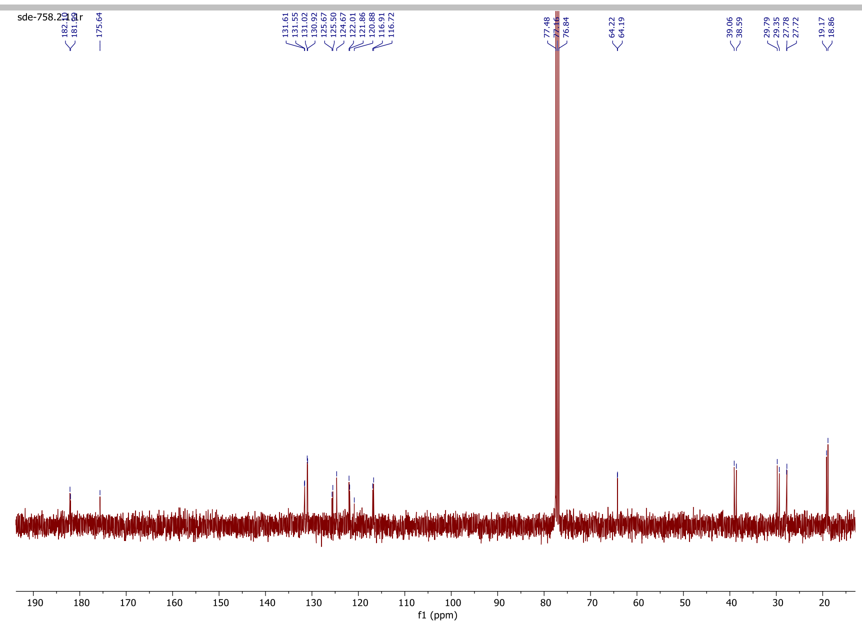

Figure S98. ^{13}C NMR spectra of compound **54.**

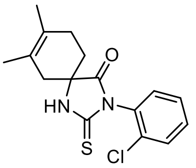

3-(2-Chlorophenyl)-7,8-dimethyl-2-thioxo-1,3-diazaspiro[4.5]dec-7-en-4-one (**55**) (isolated using chloroform as eluent). From methylideneimidazolone **4** (64 mg, 0.27 mmol), ZnI_2 (86 mg, 0.27 mmol) and 2,3-dimethylbuta-1,3-diene (108 mg, 1.35 mmol) compound **55** (61 mg, 71%) was obtained as a light yellow crystalline solid.

^{1}H NMR (400 MHz, $CDCl_3$): δ 7.84-7.82 (m, 1H), 7.57-7.56 (m, 1H), 7.45-7.41 (m, 2H), 7.37-7.33 (m, 1H), 2.75 (d, J = 17.1 Hz, 1H), 2.22 (s, 2H), 2.14-2.09 (m, 2H), 2.01-1.91 (m, 1H), 1.68 (s, 6H). **^{13}C NMR** (101 MHz, $CDCl_3$): (two sets of signals for C(Ar)-N-rotamers) δ 182.2, 181.9, 175.4 (C+C′), 133.5, 133.4, 131.2, 131.1, 131.1 (C+C′), 130.8, 130.8, 130.5 (C+C′), 127.7, 127.7, 125.5, 125.4, 121.9, 121.7, 64.2 (C+C′), 39.1, 38.4, 29.9, 29.3, 27.6, 27.5, 19.1, 19.0, 18.9, 18.8. **HRMS** (ESI+) m/z calcd. for ($C_{16}H_{18}ClN_2OS$, M+H): 321.0823, found: (M+H): 321.0827.

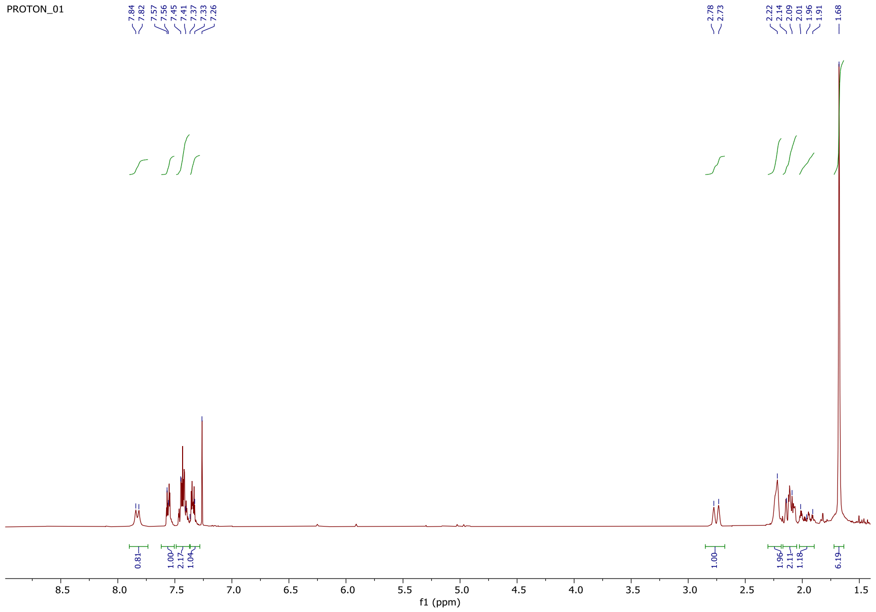

Figure S99. ^{1}H NMR spectra of compound **55.**

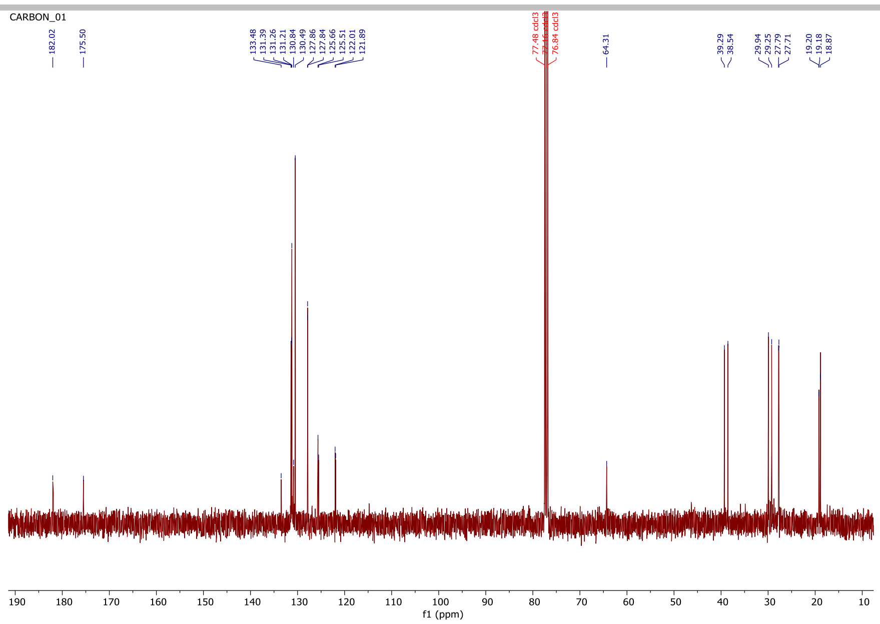

Figure S100. ^{13}C NMR spectra of compound **55.**

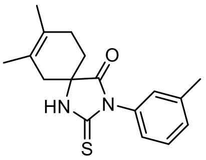

7,8-Dimethyl-2-thioxo-3-(m-tolyl)-1,3-diazaspiro[4.5]dec-7-en-4-one (**56**) (isolated using chloroform as eluent). From methylideneimidazolone **5** (59 mg, 0.27 mmol), ZnI_2 (86 mg, 0.27 mmol) and 2,3-dimethylbuta-1,3-diene (108 mg, 1.35 mmol) compound **56** (69 mg, 85%) was obtained as a white crystalline solid.

^{1}H NMR (400 MHz, $CDCl_3$): δ 8.37 (bs, 1H), 7.41-7.36 (m, 1H), 7.27-7.25 (m, 1H), 7.13 (s, 2H), 2.72 (d, J = 16.8 Hz, 1H), 2.41 (s, 3H), 2.21 (s, 2H), 2.11-2.04 (m, 2H), 1.92-1.86 (m, 1H), 1.66 (s, 6H). **^{13}C NMR** (101 MHz, $CDCl_3$): δ 182.2, 175.8, 138.7, 132.2, 129.6, 128.5, 128.4, 125.1, 125.0, 121.2, 63.4, 38.4, 29.1, 27.3, 20.9, 18.6, 18.4. **HRMS** (ESI+) m/z calcd. for ($C_{17}H_{21}N_2OS$, M+H): 301.1369, found: (M+H): 301.1380.

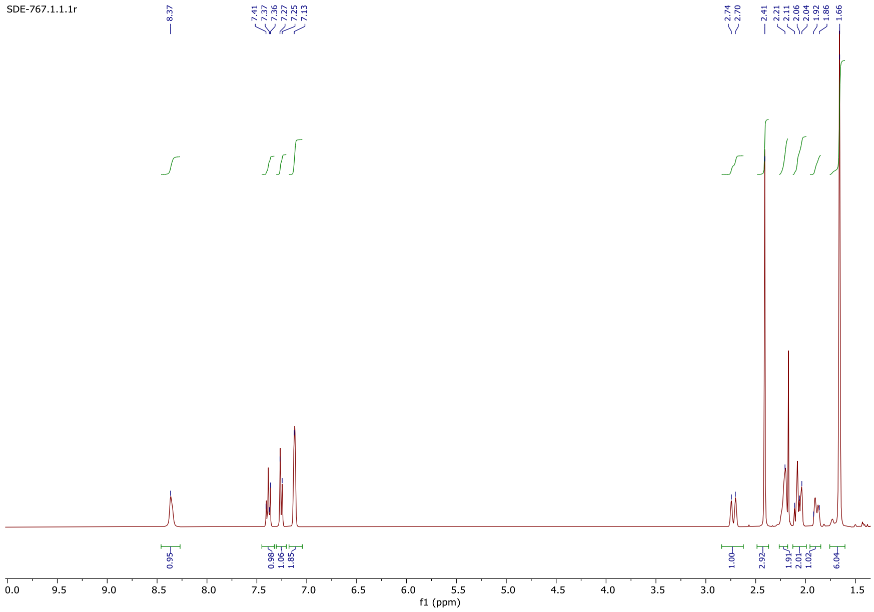

Figure S101. ^{1}H NMR spectra of compound **56.**

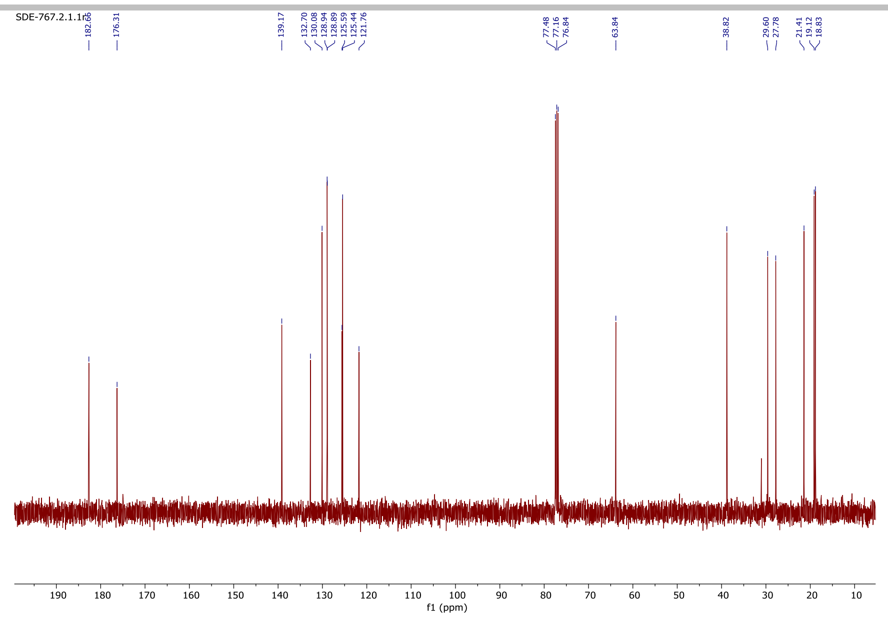

Figure S102. ^{13}C NMR spectra of compound **56.**

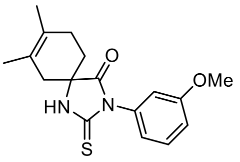

3-(3-Methoxyphenyl)-7,8-dimethyl-2-thioxo-1,3-diazaspiro[4.5]dec-7-en-4-one (**57**) (isolated using chloroform as eluent). From methylideneimidazolone **6** (63 mg, 0.27 mmol), ZnI_2 (86 mg, 0.27 mmol) and 2,3-dimethylbuta-1,3-diene (108 mg, 1.35 mmol) compound **57** (67 mg, 79%) was obtained as a white crystalline solid. **^{1}H NMR** (400 MHz, $CDCl_3$): δ 7.85 (bs, 1H), 7.40 (t, J = 8.1 Hz, 1H), 7.01-6.98 (m, 1H), 6.92-6.90 (m, 1H), 6.88-6.86 (m, 1H), 3.83 (s, 3H), 2.74 (d, J = 17.0 Hz, 1H), 2.21 (s, 2H), 2.10-2.07 (m, 2H), 1.92-1.90 (m, 1H), 1.68 (s, 6H). **^{13}C NMR** (101 MHz, $CDCl_3$): δ 182.0, 175.6, 159.6, 133.3, 129.4, 125.1, 121.4, 120.1, 114.8, 113.6, 63.3, 55.0, 38.4, 29.1, 27.3, 18.7, 18.4. **HRMS** (ESI+) m/z calcd. for ($C_{17}H_{21}N_2O_2S$, M+H): 317.1318, found: (M+H): 317.1317.

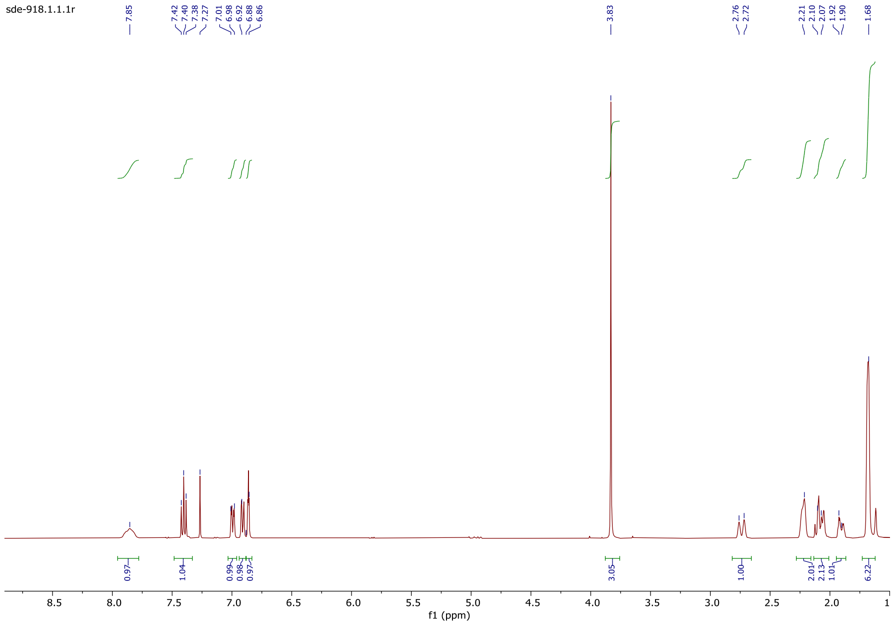

Figure S103. ^{1}H NMR spectra of compound **57.**

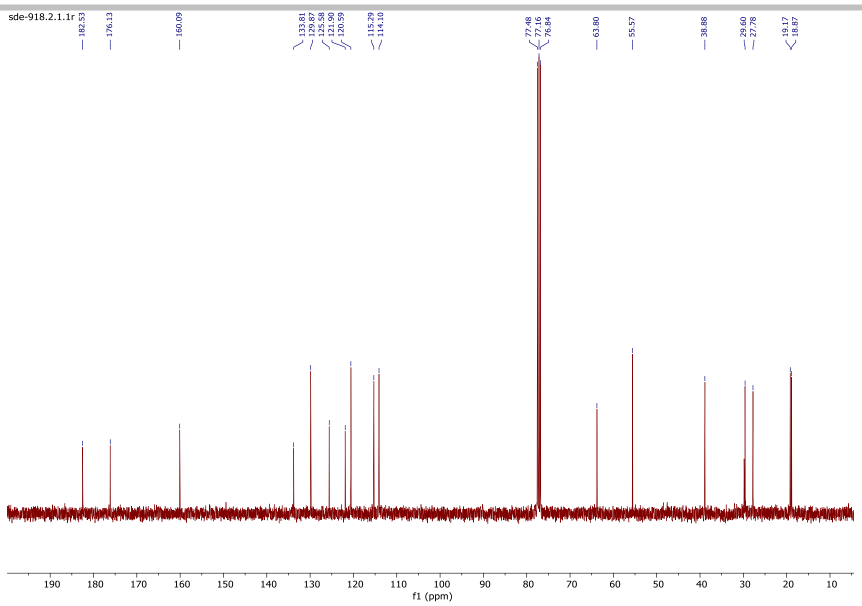

Figure S104. ^{13}C NMR spectra of compound **57.**

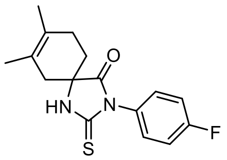

3-(4-Fluorophenyl)-7,8-dimethyl-2-thioxo-1,3-diazaspiro[4.5]dec-7-en-4-one (**58**) (isolated using chloroform as eluent). From methylideneimidazolone **7** (60 mg, 0.27 mmol), ZnI_2 (86 mg, 0.27 mmol) and 2,3-dimethylbuta-1,3-diene (108 mg, 1.35 mmol) compound **58** (61 mg, 74%) was obtained as a light yellow crystalline solid.

^{1}H NMR (400 MHz, $CDCl_3$): δ 7.99 (bs, 1H), 7.33-7.28 (m, 2H), 7.20-7.16 (m, 2H), 2.73 (d, J = 17.2 Hz, 1H), 2.24-2.21 (m, 2H), 2.12-2.04 (m, 2H), 1.92-1.87 (m, 1H), 1.68 (s, 6H). **^{13}C NMR** (101 MHz, $CDCl_3$): δ 181.9 (s), 175.6 (s), 162.2 (d, J = 249.2 Hz), 129.8 (d, J = 8.8 Hz, 2C), 128.2 (d, J = 3.1 Hz), 125.1 (s), 121.3 (s), 115.7 (d, J = 23.0 Hz, 2C), 63.3 (s), 38.4 (s), 29.1 (s), 27.2 (s), 18.6 (s), 18.3 (s). **HRMS** (ESI+) m/z calcd. for ($C_{16}H_{18}FN_2OS$, M+H): 305.1118, found: (M+H): 305.1125.

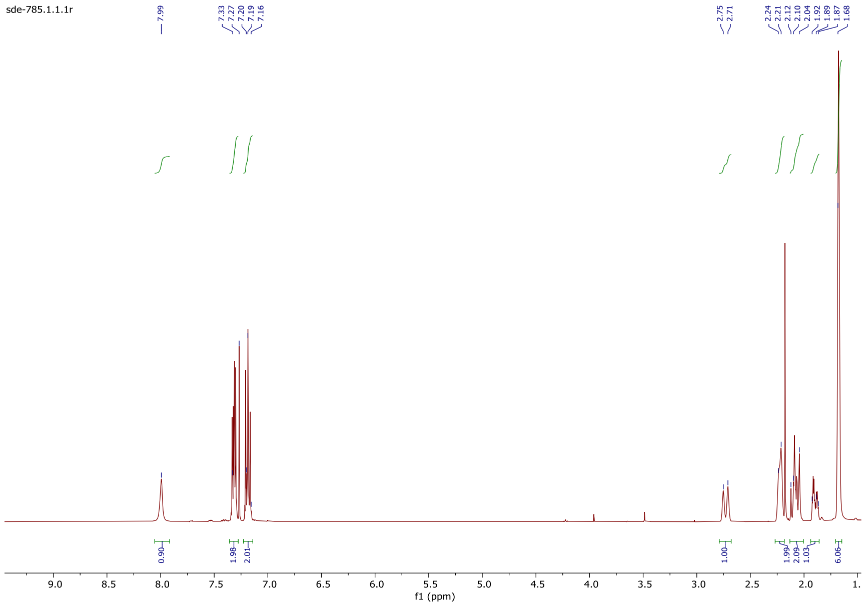

Figure S105. ^{1}H NMR spectra of compound **58.**

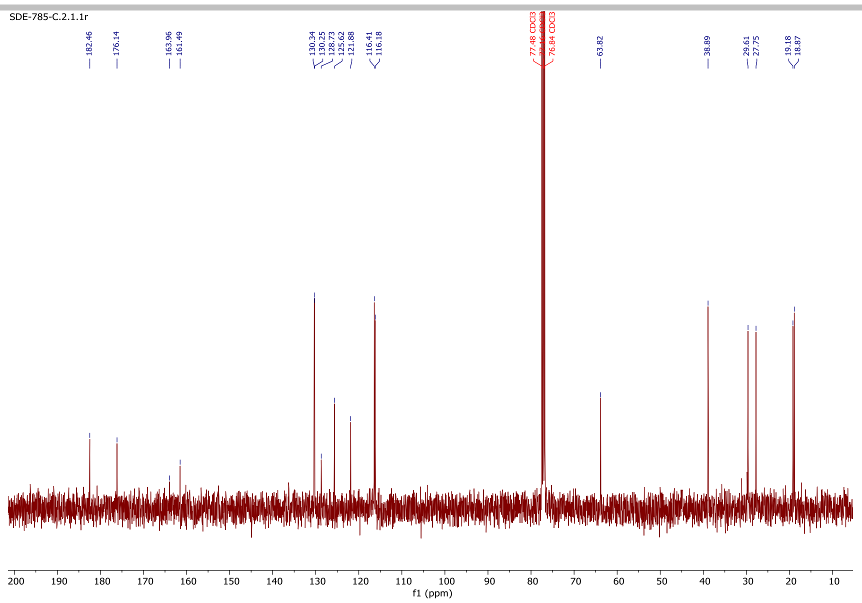

Figure S106. ^{13}C NMR spectra of compound **58.**

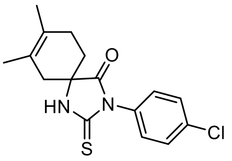

3-(4-Chlorophenyl)-7,8-dimethyl-2-thioxo-1,3-diazaspiro[4.5]dec-7-en-4-one (**59**) (isolated using chloroform as eluent). From methylideneimidazolone **8** (64 mg, 0.27 mmol), ZnI_2 (86 mg, 0.27 mmol) and 2,3-dimethylbuta-1,3-diene (108 mg, 1.35 mmol) compound **59** (71 mg, 82%) was obtained as a light yellow crystalline solid.
^{1}H NMR (400 MHz, $CDCl_3$): δ 7.87 (bs, 1H), 7.48-7.46 (m, 2H), 7.30-7.27 (m, 2H), 2.73 (d, J = 17.0 Hz, 1H), 2.24-2.22 (m, 2H), 2.12-2.04 (m, 2H), 1.91-1.88 (m, 1H), 1.69 (s, 3H), 1.68 (s, 3H). **^{13}C NMR** (101 MHz, $CDCl_3$): δ 181.6, 175.4, 134.7, 130.7, 129.2 (2C), 128.9 (2C), 125.1, 121.4, 63.3, 38.4, 29.1, 27.2, 18.6, 18.3. **HRMS** (ESI+) m/z calcd. for ($C_{16}H_{18}ClN_2OS$, M+H): 321.0823, found: (M+H): 321.0828.

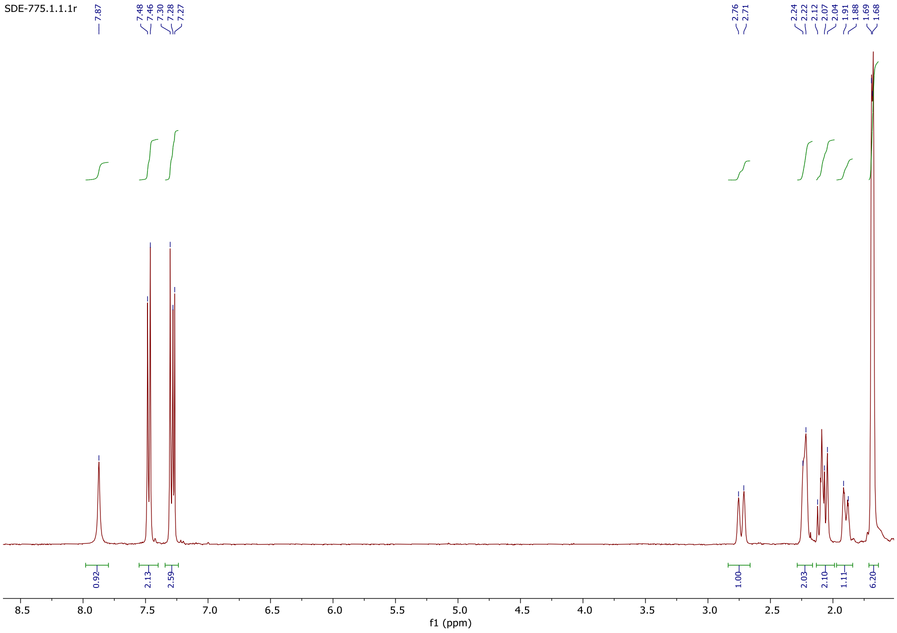

Figure S107. ^{1}H NMR spectra of compound **59**.

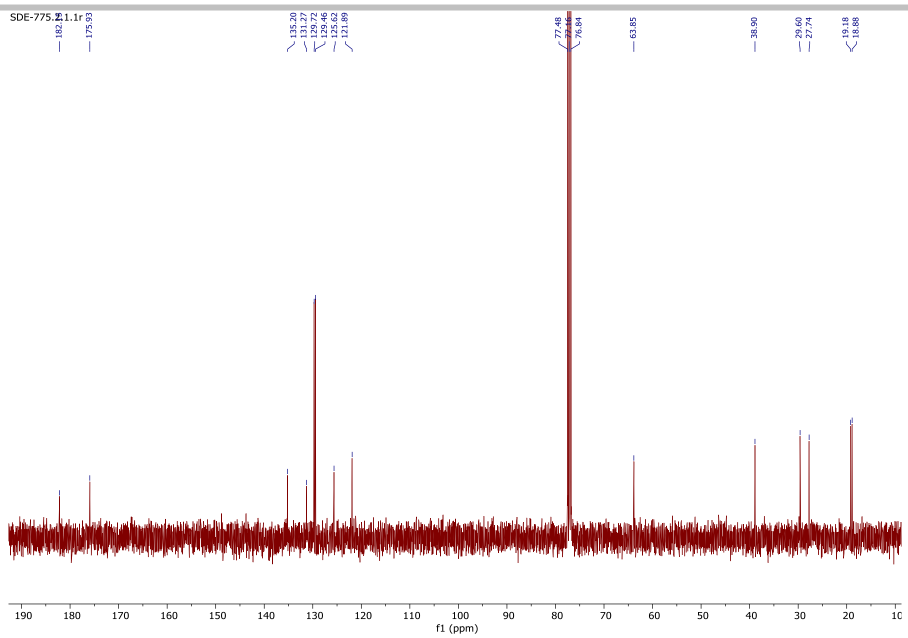

Figure S108. ^{13}C NMR spectra of compound **59**.

3-(4-Methoxyphenyl)-7,8-dimethyl-2-thioxo-1,3-diazaspiro[4.5]dec-7-en-4-one (**60**) (isolated using chloroform as eluent). From methylideneimidazolone **9** (63 mg, 0.27 mmol), ZnI_2 (86 mg, 0.27 mmol) and 2,3-dimethylbuta-1,3-diene (108 mg, 1.35 mmol) compound **60** (75 mg, 88%) was obtained as a light yellow crystalline solid.

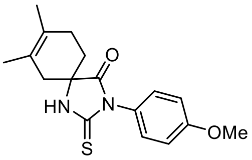

^{1}H NMR (400 MHz, $CDCl_3$): δ 7.73 (bs, 1H), 7.27-7.22 (m, 2H), 7.01-6.99 (m, 2H), 3.85 (s, 3H), 2.73 (d, J = 16.9 Hz, 1H), 2.23 (s, 2H), 2.12-2.07 (m, 2H), 1.91-1.87 (m, 1H), 1.69 (s, 3H), 1.67 (s, 3H). **^{13}C NMR** (101 MHz, $CDCl_3$): δ 182.5, 175.9, 159.5, 129.0 (2C), 125.0, 124.9, 121.5, 114.0 (2C), 63.1, 55.1, 38.4, 29.0, 27.2, 18.6, 18.3. **HRMS** (ESI+) m/z calcd. for ($C_{17}H_{21}N_2O_2S$, M+H): 317.1318, found: (M+H): 317.1325.

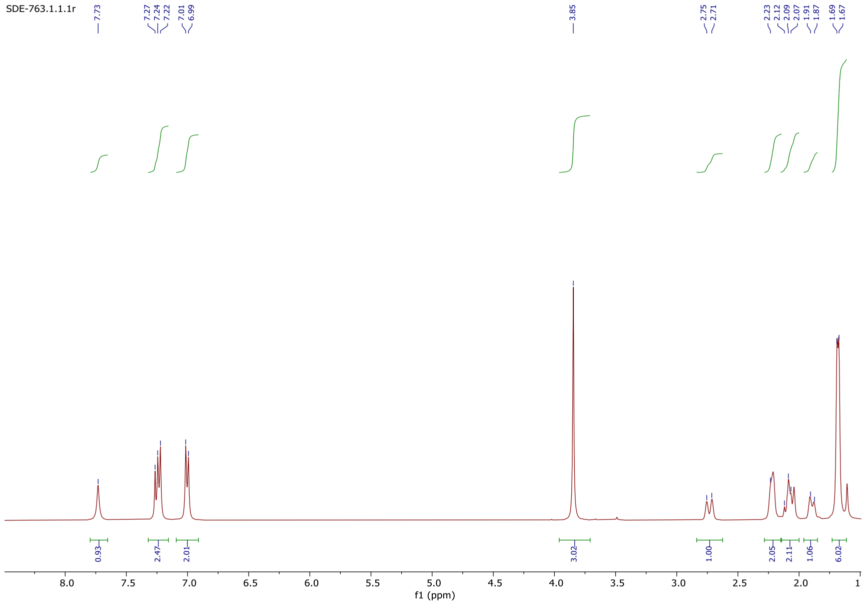

Figure S109. ^{1}H NMR spectra of compound **60**.

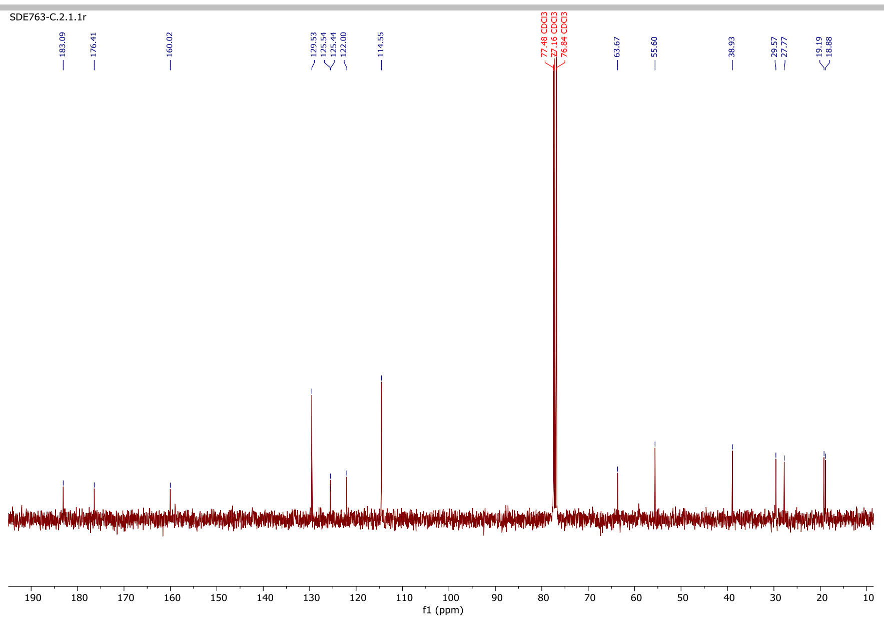

Figure S110. ^{13}C NMR spectra of compound **60**.

3-(2,6-Dimethylphenyl)-7,8-dimethyl-2-thioxo-1,3-diazaspiro[4.5]dec-7-en-4-one (**61**) (isolated using chloroform as eluent). From methylideneimidazolone **10** (63 mg, 0.27 mmol), ZnI_2 (86 mg, 0.27 mmol) and 2,3-dimethylbuta-1,3-diene (108 mg, 1.35 mmol) compound **61** (58 mg, 68%) was obtained as a yellow light crystalline solid.

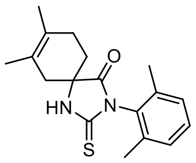

^{1}**H NMR** (400 MHz, $CDCl_3$): δ 8.07 (bs, 1H), 7.30-7.26 (m, 1H), 7.18-7.16 (m, 2H), 2.77 (d, J = 16.6 Hz, 1H), 2.22 (s, 2H), 2.17 (s, 6H), 2.13-2.03 (m, 2H), 1.91-1.88 (m, 1H), 1.68 (s, 6H). ^{13}**C NMR** (101 MHz, $CDCl_3$): δ 181.9, 176.1, 136.8, 136.7, 131.0, 129.8 (2C), 128.6, 125.6, 121.8, 64.1, 39.1, 29.7, 27.7, 19.2, 18.9, 17.9, 17.8. **HRMS** (ESI+) m/z calcd. for ($C_{18}H_{23}N_2OS$, M+H): 315.1526, found: (M+H): 315.1530.

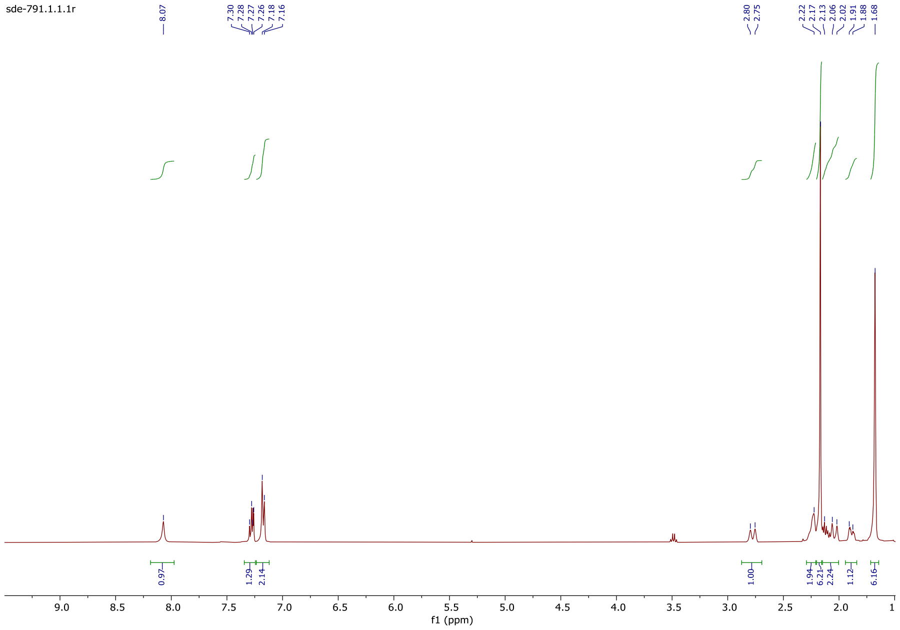

Figure S111. ^{1}H NMR spectra of compound **61**.

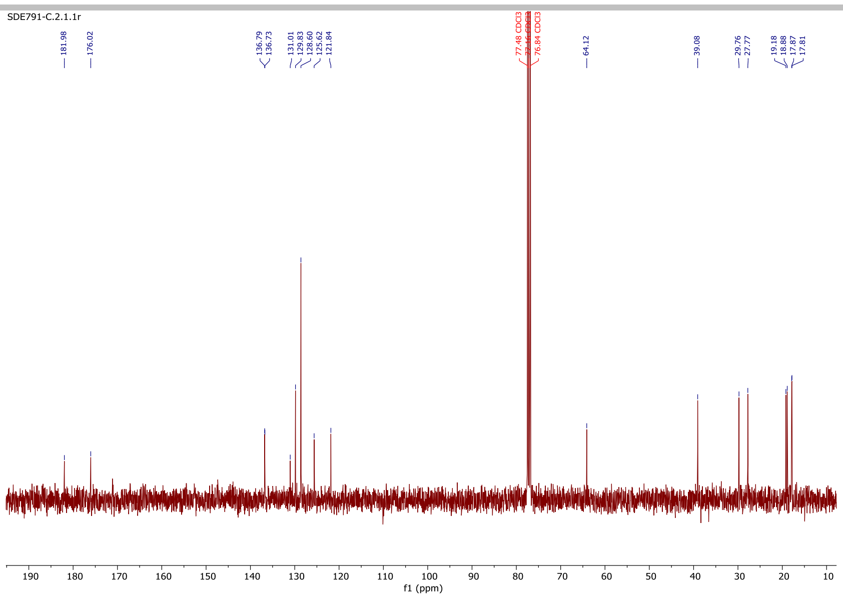

Figure S112. ^{13}C NMR spectra of compound **61**.

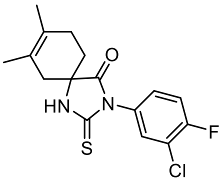

3-(3-Chloro-4-fluorophenyl)-7,8-dimethyl-2-thioxo-1,3-diazaspiro[4.5]dec-7-en-4-one (**62**) (isolated using chloroform as eluent). From methylideneimidazolone **11** (69 mg, 0.27 mmol), ZnI_2 (86 mg, 0.27 mmol) and 2,3-dimethylbuta-1,3-diene (108 mg, 1.35 mmol) compound **62** (74 mg, 81%) was obtained as a light yellow crystalline solid. **^{1}H NMR** (400 MHz, $CDCl_3$): δ 8.10 (bs, 1H), 7.45-7.43 (m, 1H), 7.27-7.25 (m, 2H), 2.73 (d, J = 17.1 Hz, 1H), 2.24 (s, 2H), 2.12-2.04 (m, 2H), 1.91-1.88 (m, 1H), 1.68 (s, 6H). **^{13}C NMR** (101 MHz, $CDCl_3$): δ 181.3 (s), 175.3 (s), 157.8 (d, J = 251.4 Hz), 130.4 (s, 2C), 128.6 (s), 128.0 (d, J = 7.9 Hz), 125.1 (s), 121.6 (s), 116.5 (d, J = 22.9 Hz), 63.4 (s), 38.3 (s), 29.1 (s), 27.2 (s), 18.6 (s), 18.3 (s). **HRMS** (ESI+) m/z calcd. for ($C_{16}H_{17}ClFN_2OS$, M+H): 339.0729, found: (M+H): 339.0736.

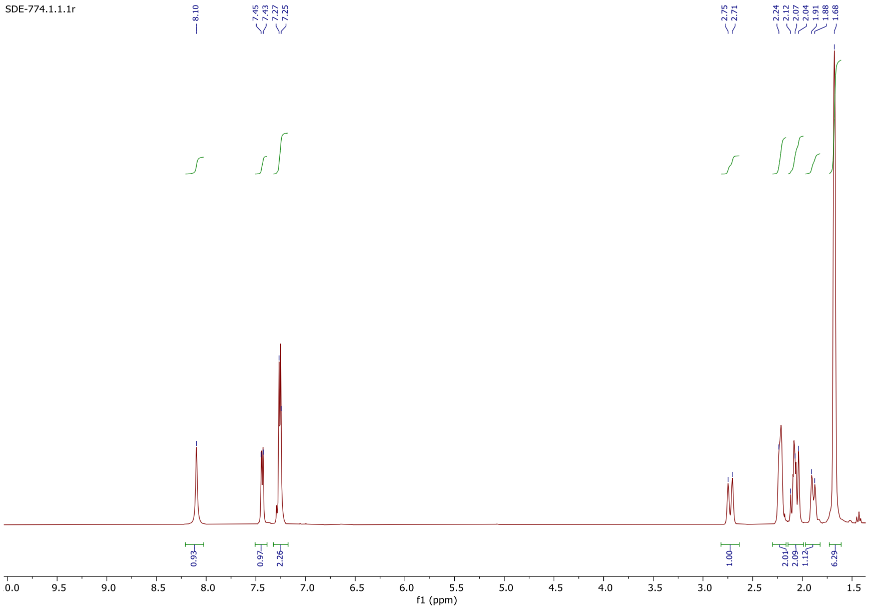

Figure S113. ^1H NMR spectra of compound **62**.

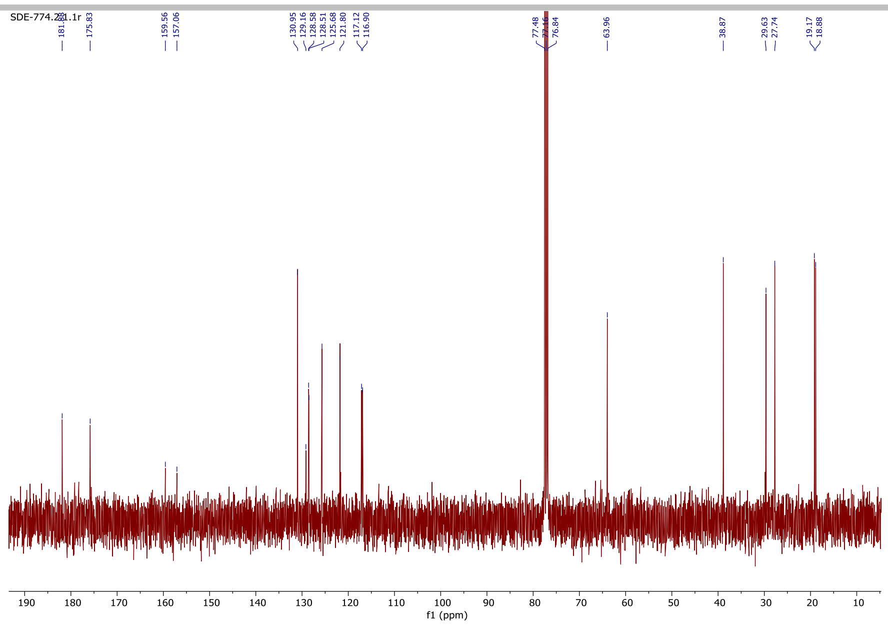

Figure S114. ^{13}C NMR spectra of compound **62**.

General procedure of Diels-Alder reaction of methylideneimidazolones 1, 2, 4-6, 8, 9 with isoprene.

To a solution of methylideneimidazolone (0.27 mmol) and $AlCl_3$ or ZnI_2 (0.27 mmol) in boiling chloroform (15 ml) isoprene (92 mg, 1.35 mmol) was added and the mixture was refluxed for 6 h. The reaction was cooled to room temperature and the mixture was washed with water (15 ml) and filtered. The organic phase was dried over anhydrous Na_2SO_4, filtered and evaporated under vacuum. The residue was purified by column chromatography on silica gel.

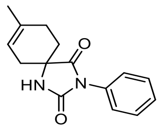

8-Methyl-3-phenyl-1,3-diazaspiro[4.5]dec-7-ene-2,4-dione (**63a**) (isolated using methanol/chloroform (1:400) as eluent). From methylidenehydantoin **1** (51 mg, 0.27 mmol), $AlCl_3$ (36 mg, 0.27 mmol) and isoprene (92 mg, 1.35 mmol) compound **63a** (48 mg, 70%) was obtained as a white crystalline solid.

^{1}H NMR (400 MHz, $CDCl_3$): δ 7.47-7.44 (m, 4H), 7.38-7.33 (m, 1H), 7.26 (bs, 1H), 5.43-5.37 (m, 1H), 2.75-2.69 (m, 1H), 2.18-2.06 (m, 4H), 1.88-1.82 (m, 1H), 1.68 (s, 3H). **^{13}C NMR** (101 MHz, $CDCl_3$): δ 176.0, 156.1, 134.1, 131.8, 128.9 (2C), 128.0, 126.0 (2C), 116.8, 116.7, 59.8, 34.0, 29.7, 26.3, 23.2. **HRMS** (ESI+) m/z calcd. for ($C_{15}H_{17}N_2O_2$, M+H): 257.1290, found: (M+H): 257.1283.

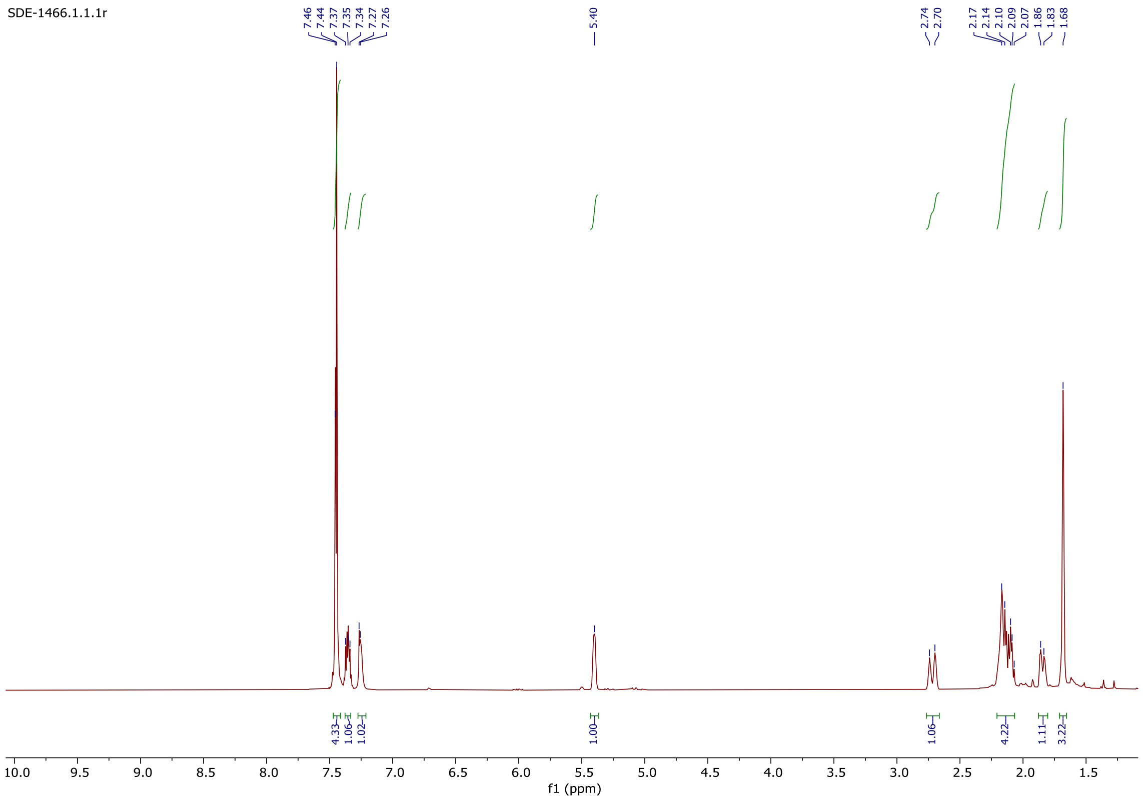

Figure S115. ^{1}H NMR spectra compound **63a**.

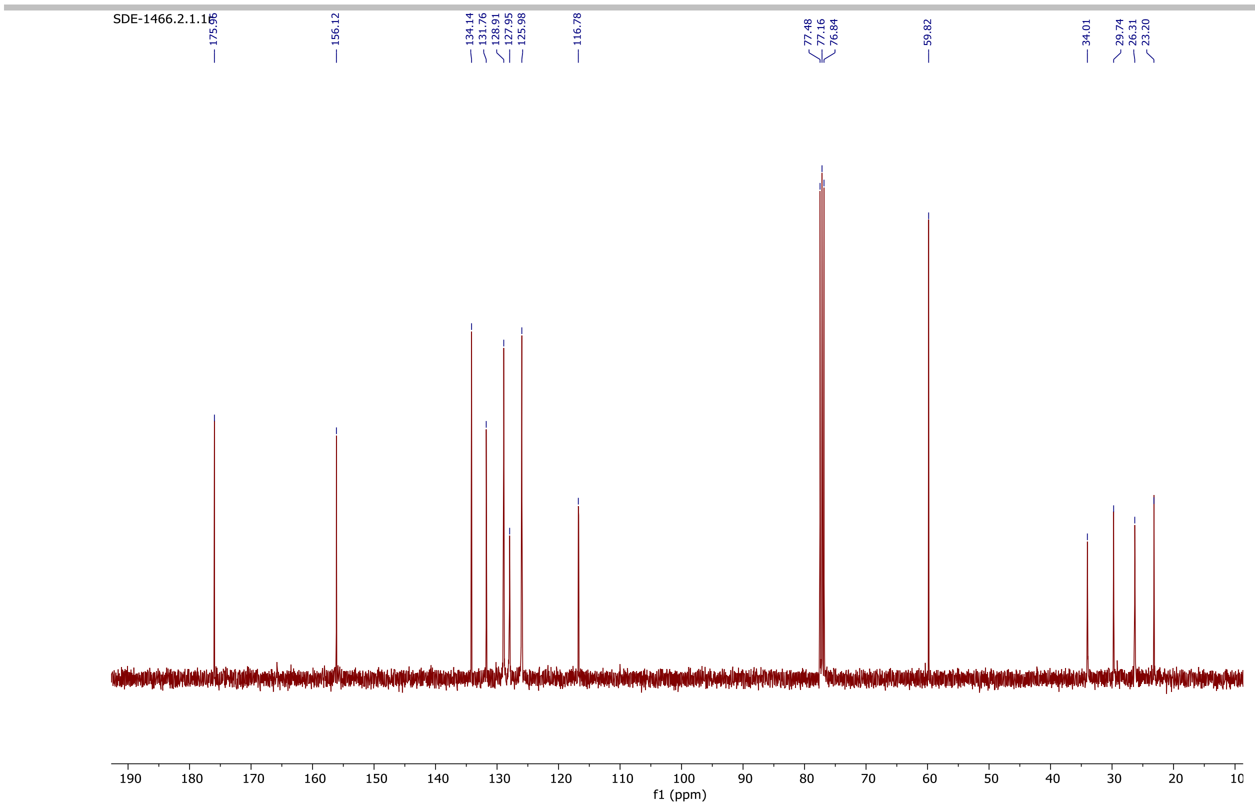

Figure S116. ^{13}C NMR spectra of compound **63a**.

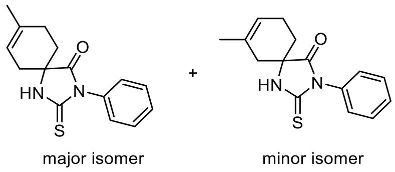

8-Methyl-3-phenyl-2-thioxo-1,3-diazaspiro[4.5]dec-7-en-4-one (**64a**) and *7-methyl-3-phenyl-2-thioxo-1,3-diazaspiro[4.5]dec-7-en-4-one* (**64b**) (isolated using chloroform as eluent). From methylidenethiohydantoin **2** (55 mg, 0.27 mmol), ZnI_2 (86 mg, 0.27 mmol) and isoprene (92 mg, 1.35 mmol) the mixture of compounds **64a** and **64b** in 87/13 ratio (60 mg, 82%) was obtained as a white crystalline solid.

Major isomer 64a: ^{1}H NMR (400 MHz, $CDCl_3$): δ 8.26 (bs, 1H), 7.53-7.43 (m, 3H), 7.34-7.32 (m, 2H), 5.45-5.43 (m, 1H), 2.75-2.70 (m, 1H), 2.25-2.05 (m, 4H), 1.97-1.92 (m, 1H), 1.73 (s, 3H). **^{13}C NMR** (101 MHz, $CDCl_3$): δ 182.1, 175.9, 133.8, 132.3, 128.8, 128.7 (2C), 127.9 (2C), 116.2, 62.3, 32.8, 28.9, 25.8, 22.9.

Minor isomer 64b: ^{1}H NMR (400 MHz, $CDCl_3$): δ 8.19 (bs, 1H), 7.53-7.43 (m, 3H), 7.34-7.32 (m, 2H), 5.55 (s, 1H), 2.75-2.70 (m, 1H), 2.25-2.05 (m, 4H), 1.97-1.92 (m, 1H), 1.73 (s, 3H).

HRMS (ESI+) m/z calcd. for ($C_{15}H_{17}N_2OS$, M+H): 273.1056, found: (M+H): 273.1054.

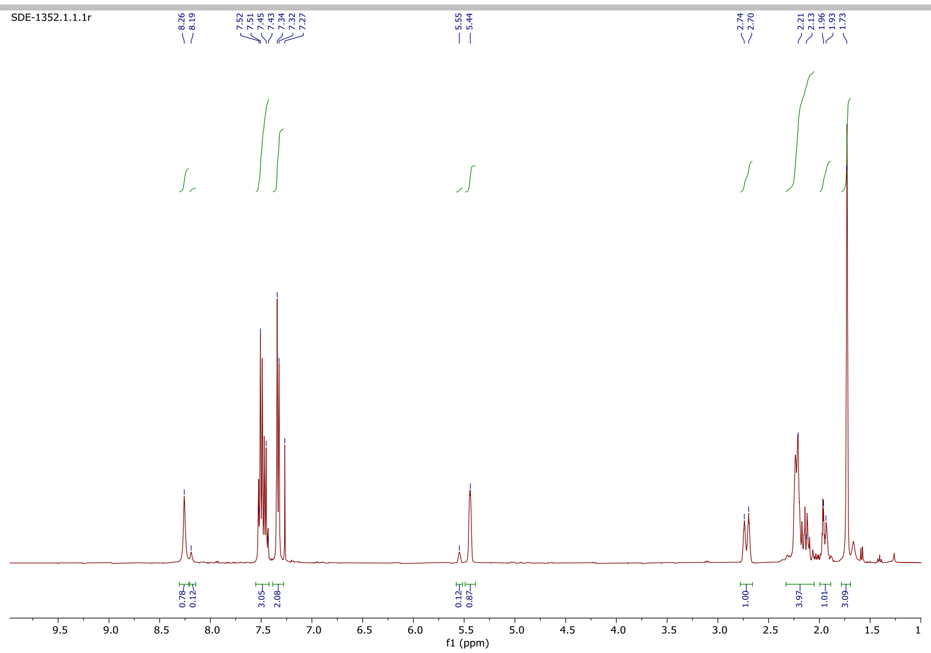

Figure S117. ^{1}H NMR spectra of the mixture of compounds **64a** and **64b**.

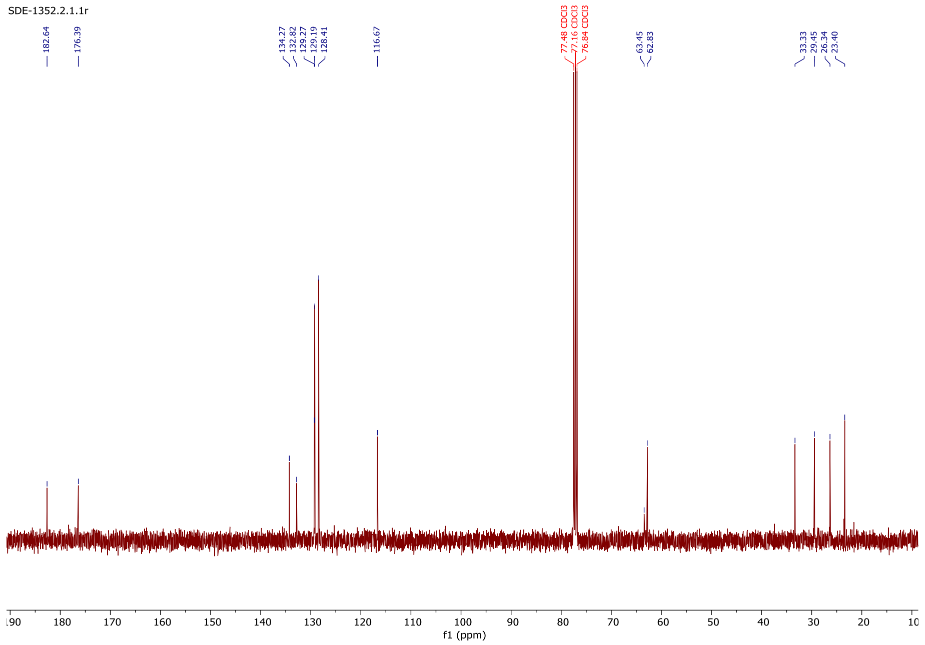

Figure S118. ^{13}C NMR spectra of the mixture of compounds **64a** and **64b**.

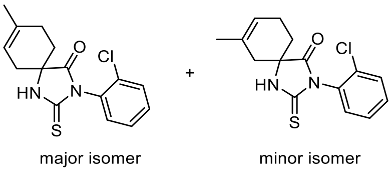

3-(2-Chlorophenyl)-8-methyl-2-thioxo-1,3-diazaspiro[4.5]dec-7-en-4-one (**65a**) and *3-(2-chlorophenyl)-7-methyl-2-thioxo-1,3-diazaspiro[4.5]dec-7-en-4-one* (**65b**) (isolated using chloroform as eluent). From methylidenethiohydantoin **4** (64 mg, 0.27 mmol), ZnI_2 (86 mg, 0.27 mmol) and isoprene (92 mg, 1.35 mmol) the mixture of compounds **65a** and **65b** in 94/6 ratio (51 mg, 61%) was obtained as a white crystalline solid.

Major isomer 65a: ^{1}**H NMR** (400 MHz, $CDCl_3$): δ 7.92 (bs, 1H), 7.57-7.55 (m, 1H), 7.47-7.40 (m, 2H), 7.36-7.35 (m, 1H), 5.47-5.45 (m, 1H), 2.77-2.72 (m, 1H), 2.32-2.12 (m, 4H), 2.07-1.96 (m, 1H), 1.74 (s, 3H). ^{13}**C NMR** (101 MHz, $CDCl_3$): (two sets of signals for C(Ar)-N-rotamers) δ 181.9, 181.8, 175.5, 175.4,

134.3, 134.2, 133.5, 133.4, 131.4, 131.3, 131.0 (C+C′), 130.3 (C+C′), 127.7 (C+C′), 116.8, 116.8, 116.7, 116.7, 63.2, 63.1, 33.7, 32.8, 30.2, 30.1, 26.2, 26.1, 23.2, 23.2.

Minor isomer 65b: ^{1}**H NMR** (400 MHz, $CDCl_3$): δ 7.92 (bs, 1H), 7.57-7.55 (m, 1H), 7.47-7.40 (m, 2H), 7.36-7.35 (m, 1H), 5.56 (s, 1H), 2.77-2.72 (m, 1H), 2.32-2.12 (m, 4H), 2.07-1.96 (m, 1H), 1.74 (s, 3H).

HRMS (ESI+) m/z calcd. for ($C_{15}H_{16}ClN_2OS$, M+H): 307.0666, found: (M+H): 307.0665.

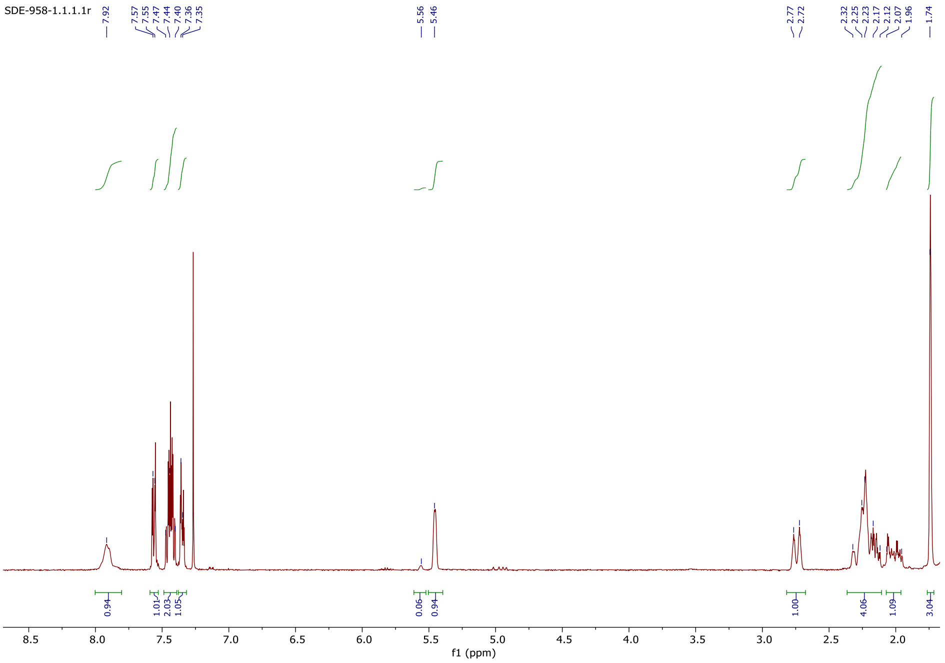

Figure S119. ^{1}H NMR spectra of the mixture of compounds **65a** and **65b**.

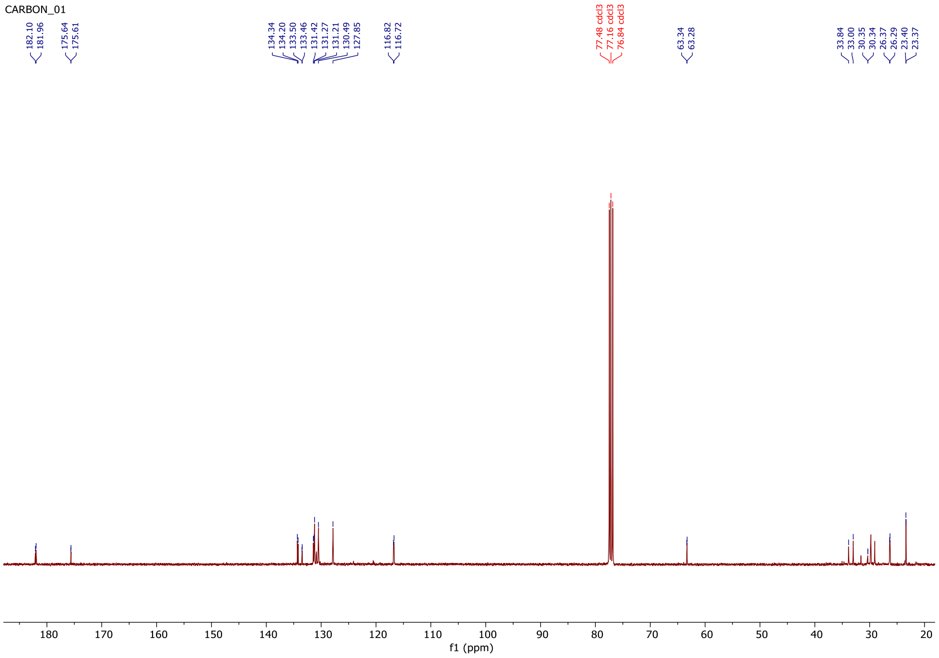

Figure S120. ^{13}C NMR spectra of the mixture of compounds **65a** and **65b**.

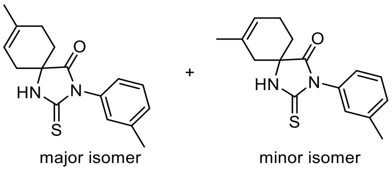

8-Methyl-2-thioxo-3-(m-tolyl)-1,3-diazaspiro[4.5]dec-7-en-4-one (**66a**) and *7-methyl-2-thioxo-3-(m-tolyl)-1,3-diazaspiro[4.5]dec-7-en-4-one* (**66b**) (isolated using chloroform as eluent). From methylidenethiohydantoin **5** (59 mg, 0.27 mmol), ZnI_2 (86 mg, 0.27 mmol) and isoprene (92 mg,

1.35 mmol) the mixture of compounds **66a** and **66b** in 89/11 ratio (49 mg, 64%) was obtained as a white crystalline solid.

Major isomer 66a: **^{1}H NMR** (400 MHz, $CDCl_3$): δ 7.78 (bs, 1H), 7.39-7.37 (m, 1H), 7.27-7.25 (m, 1H), 7.12-7.11 (m, 2H), 5.46-5.44 (m, 1H), 2.74-2.70 (m, 1H), 2.41 (s, 3H), 2.24-2.10 (m, 4H), 1.96-1.91 (m, 1H), 1.75 (s, 3H). **^{13}C NMR** (101 MHz, $CDCl_3$): δ 182.7, 176.3, 139.2, 134.1, 132.6, 130.1, 128.9, 128.8, 125.3, 116.6, 62.6, 33.2, 29.3, 26.2, 23.2, 21.3.

Minor isomer 66b: **^{1}H NMR** (400 MHz, $CDCl_3$): δ 7.72 (bs, 1H), 7.39-7.37 (m, 1H), 7.27-7.25 (m, 1H), 7.12-7.11 (m, 2H), 5.56 (s, 1H), 2.74-2.70 (m, 1H), 2.41 (s, 3H), 2.24-2.10 (m, 4H), 1.96-1.91 (m, 1H), 1.75 (s, 3H).

HRMS (ESI+) m/z calcd. for ($C_{16}H_{19}N_2OS$, M+H): 287.1213, found: (M+H): 287.1212.

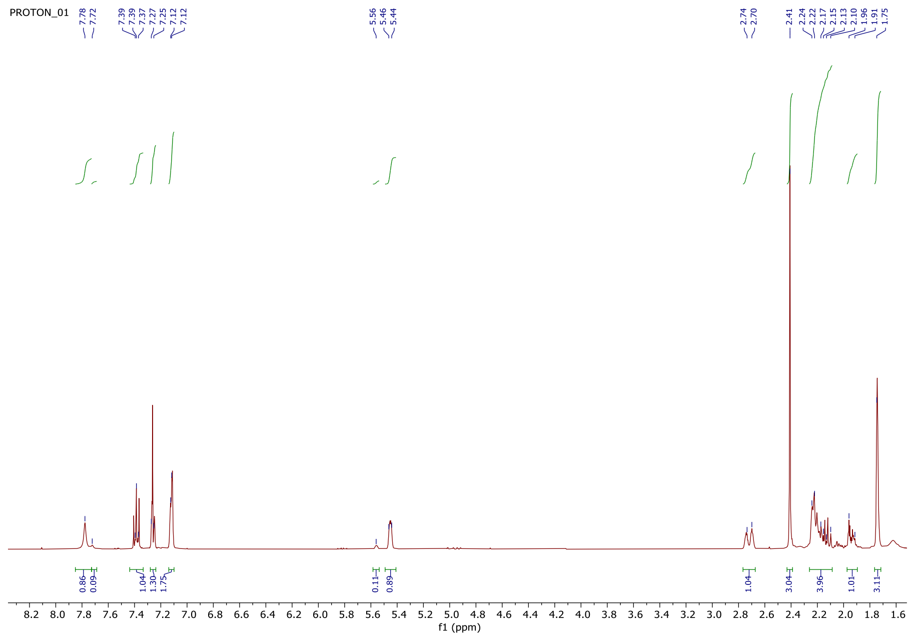

Figure S121. ^{1}H NMR spectra of the mixture of compounds **66a** and **66b**.

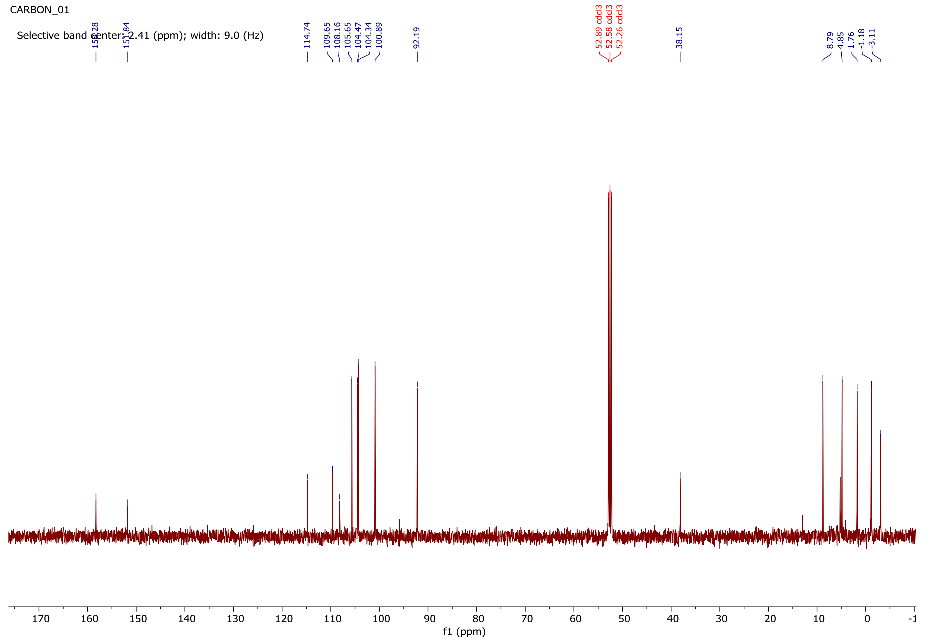

Figure S122. ^{13}C NMR spectra of the mixture of compounds **66a** and **66b**.

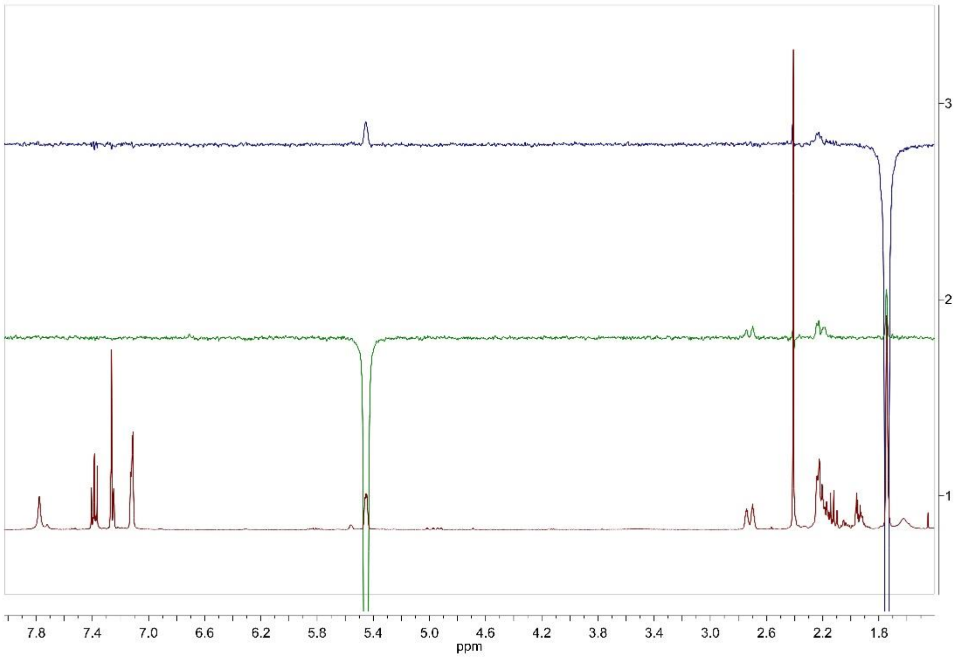

Figure S123. ^{1}H NOESY1D NMR spectra of the mixture of compounds **66a** and **66b**.

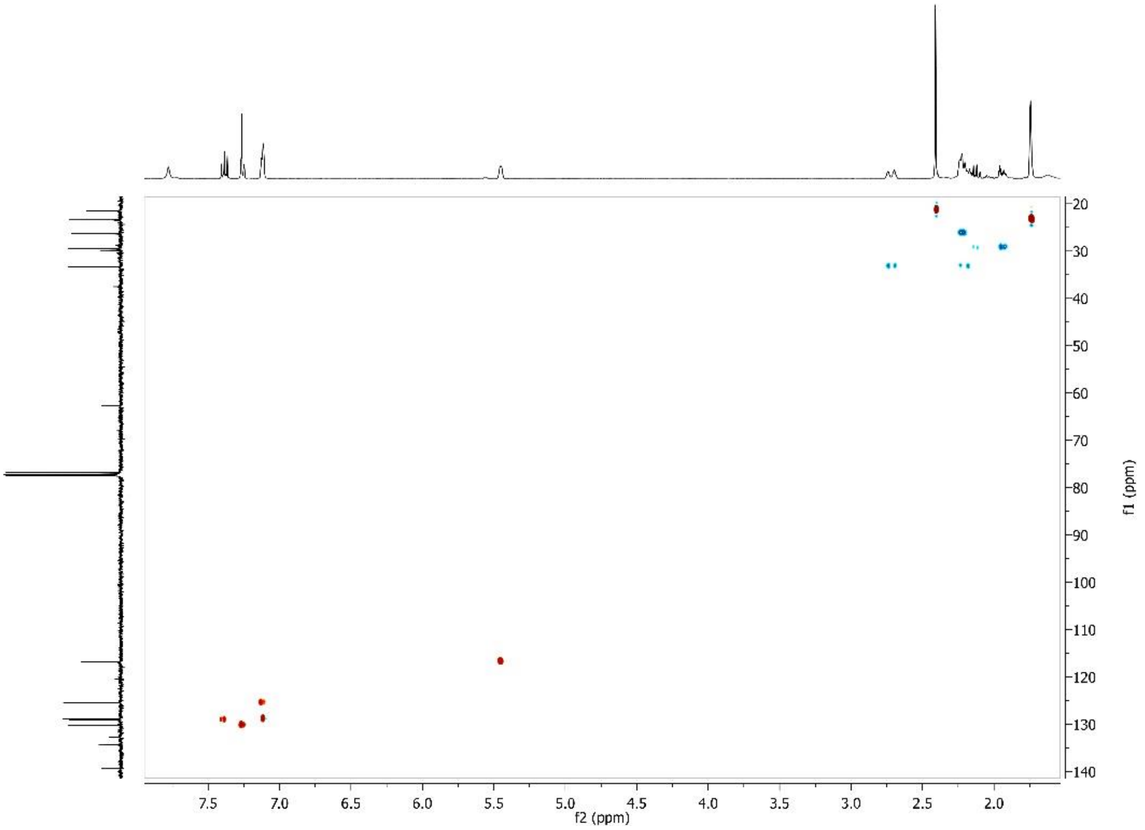

Figure S124. ^{1}H-^{13}C HCQS NMR spectra of the mixture of compounds **66a** and **66b**.

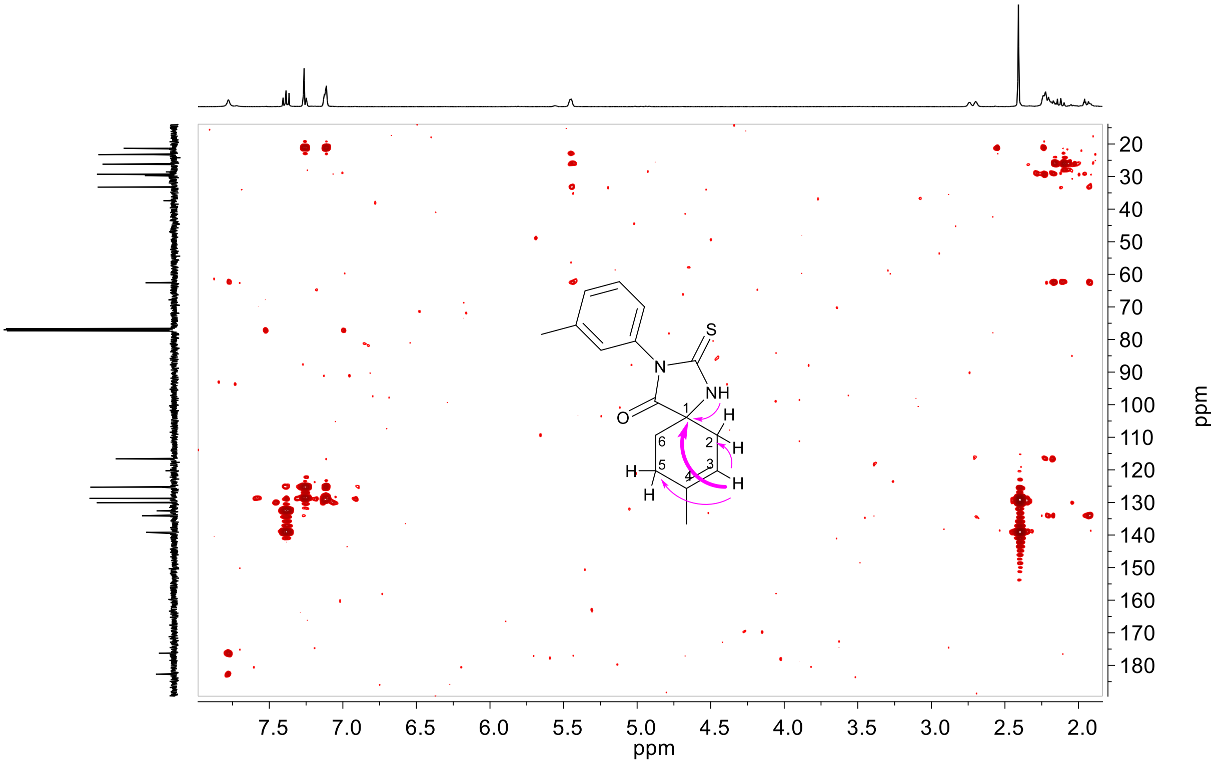

Figure S125. ^1H-^{13}C HMBC NMR spectra of the mixture of compounds **66a** and **66b**.

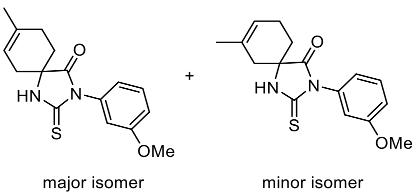

3-(3-methoxyphenyl)-8-methyl-2-thioxo-1,3-diazaspiro[4.5]dec-7-en-4-one (**67a**) and *3-(3-methoxyphenyl)-7-methyl-2-thioxo-1,3-diazaspiro[4.5]dec-7-en-4-one* (**67b**) (isolated using chloroform as eluent). From methylidenethiohydantoin **6** (63 mg, 0.27 mmol), ZnI_2 (86 mg, 0.27 mmol) and isoprene (92 mg, 1.35 mmol) the mixture of compounds **67a** and **67b** in 90/10 ratio (62 mg, 76%) was obtained as a white crystalline solid.

Major isomer 67a: ^{1}**H NMR** (400 MHz, $CDCl_3$): δ 7.77 (bs, 1H), 7.43-7.37 (m, 1H), 7.00 (ddd, J_1 = 0.8 Hz, J_2 = 2.5 Hz, J_3 = 8.4 Hz, 1H), 6.91 (ddd, J_1 = 0.8 Hz, J_2 = 2.5 Hz, J_3 = 8.4 Hz, 1H), 6.87-6.85 (m, 1H), 5.46-5.45 (m, 1H), 3.83 (s, 3H), 2.75-2.70 (m, 1H), 2.24-2.02 (m, 4H), 1.97-1.95 (m, 1H), 1.75 (s, 3H). ^{13}**C NMR** (101 MHz, $CDCl_3$): δ 182.4, 176.2, 160.0, 134.2, 133.7, 129.7, 120.5, 116.6, 115.1, 114.1, 62.8, 55.5, 33.2, 29.4, 26.3, 23.3.

Minor isomer 67b: ^{1}**H NMR** (400 MHz, $CDCl_3$): δ 7.73 (bs, 1H), 7.43-7.37 (m, 1H), 7.01-6.99 (m, 1H), 6.93-6.89 (m, 1H), 6.87-6.85 (m, 1H), 5.57-5.55 (m, 1H), 3.83 (s, 3H), 2.75-2.70 (m, 1H), 2.24-2.02 (m, 4H), 1.97-1.95 (m, 1H), 1.75 (s, 3H).

HRMS (ESI+) m/z calcd. for ($C_{16}H_{19}N_2O_2S$, M+H): 303.1162, found: (M+H): 303.1165.

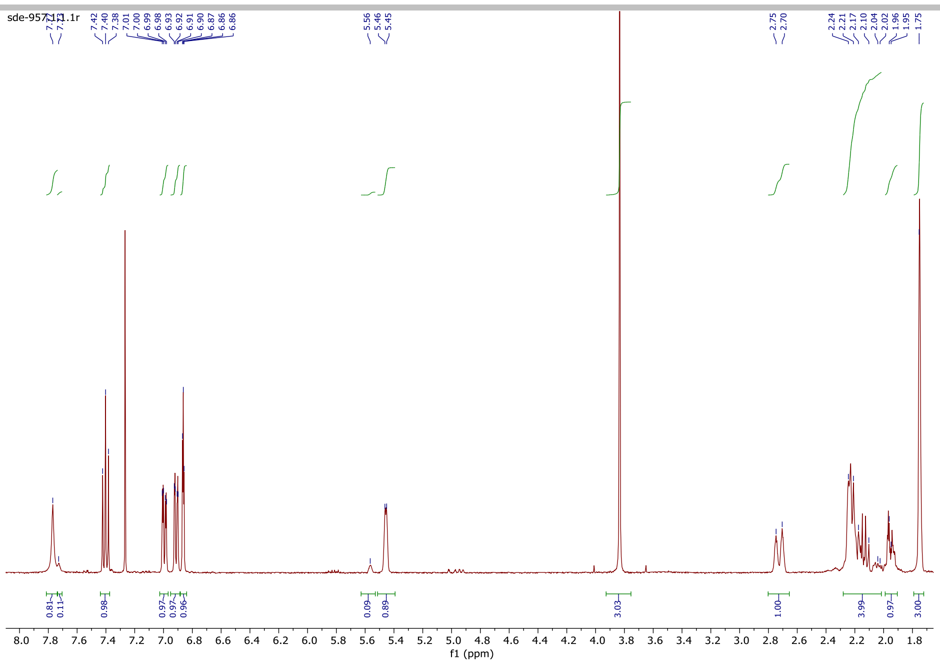

Figure S126. ^{1}H NMR spectra of the mixture of compounds **67a** and **67b**.

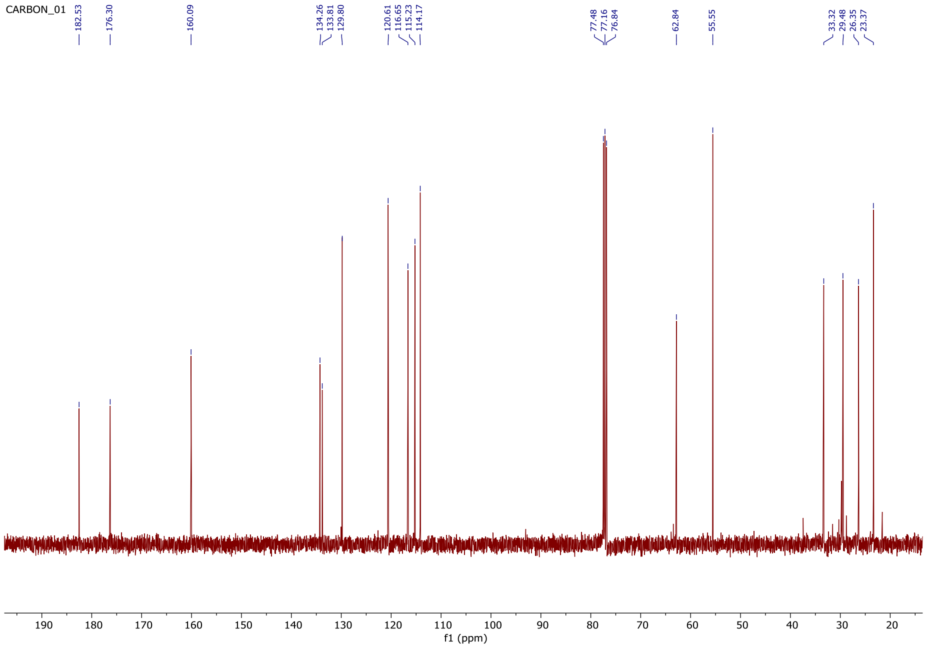

Figure S127. ^{13}C NMR spectra of the mixture of compounds **67a** and **67b**.

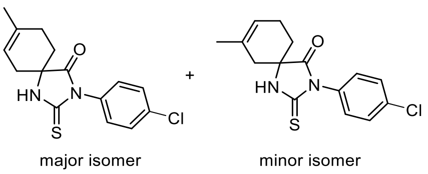

3-(4-chlorophenyl)-8-methyl-2-thioxo-1,3-diazaspiro[4.5]dec-7-en-4-one (**68a**) and *3-(4-chlorophenyl)-7-methyl-2-thioxo-1,3-diazaspiro[4.5]dec-7-en-4-one* (**68b**) (isolated using chloroform as eluent). From methylidenethiohydantoin **8** (64 mg, 0.27 mmol), ZnI_2 (86 mg, 0.27 mmol) and isoprene (92 mg, 1.35 mmol) the mixture of compounds **68a** and **68b** in 87/13 ratio (57 mg, 68%) was obtained as a white crystalline solid.

Major isomer 68a: ^{1}**H NMR** (400 MHz, $CDCl_3$): δ 7.70 (bs, 1H), 7.48-7.45 (m, 2H), 7.31-7.30 (m, 1H), 7.29-7.28 (m, 1H), 5.46-5.45 (m, 1H), 2.74-2.70 (m, 1H), 2.24-2.06 (m, 4H), 1.97-1.92 (m, 1H), 1.76 (s, 3H) ^{13}**C NMR** (101 MHz, $CDCl_3$): δ 182.1, 175.9, 135.1, 134.1, 131.2, 129.6 (2C), 129.3 (2C), 116.5, 62.7, 33.2, 29.3, 26.2, 23.2.

Minor isomer 68b: [1]**H NMR** (400 MHz, $CDCl_3$): δ 7.65 (bs, 1H), 7.48-7.45 (m, 2H), 7.31-7.30 (m, 1H), 7.29-7.28 (m, 1H), 5.57-5.56 (m, 1H), 2.74-2.70 (m, 1H), 2.24-2.06 (m, 4H), 1.97-1.92 (m, 1H), 1.76 (s, 3H). **HRMS** (ESI+) m/z calcd. for ($C_{15}H_{16}ClN_2OS$, M+H): 307.0666, found: (M+H): 307.0663.

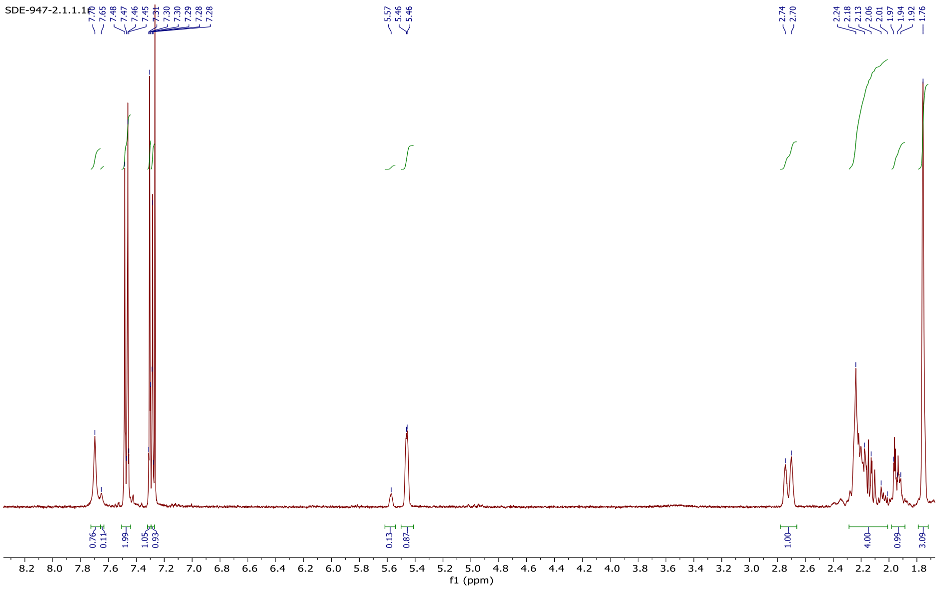

Figure S128. ^{1}H NMR spectra of the mixture of compounds **68a** and **68b**.

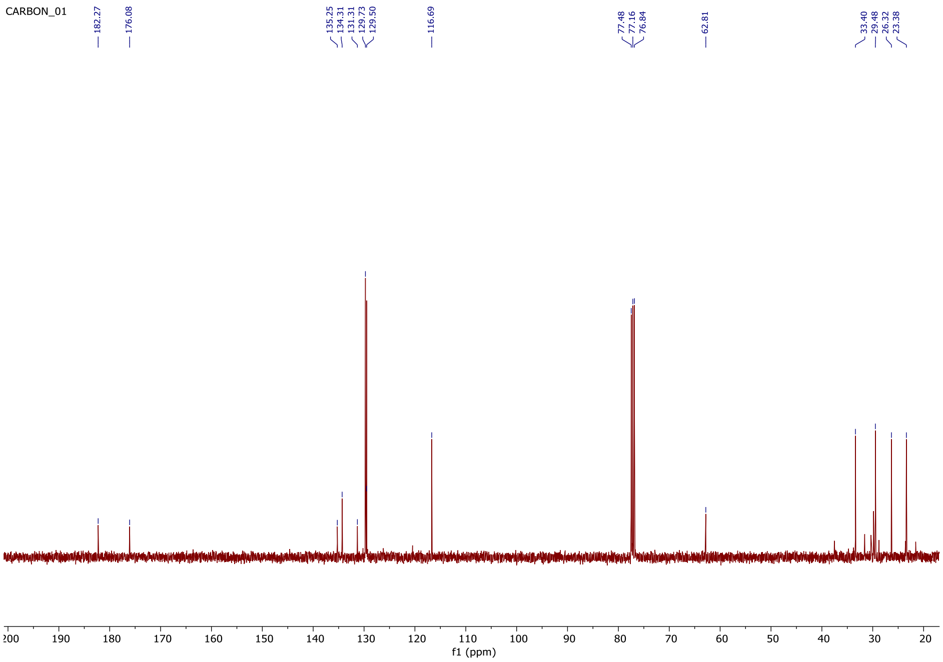

Figure S129. ^{13}C NMR spectra of the mixture of compounds **68a** and **68b**.

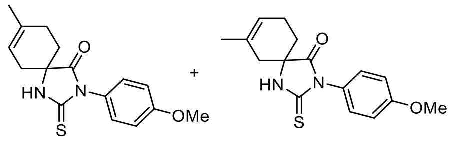

major isomer minor isomer

3-(4-methoxyphenyl)-8-methyl-2-thioxo-1,3-diazaspiro[4.5]dec-7-en-4-one (**69a**) and *3-(4-methoxyphenyl)-7-methyl-2-thioxo-1,3-diazaspiro[4.5]dec-7-en-4-one* (**69b**) (isolated using chloroform as eluent). From methylidenethiohydantoin **9** (63 mg, 0.27 mmol), ZnI_2 (86 mg, 0.27 mmol) and isoprene (92 mg, 1.35 mmol) the mixture of compounds **69a** and **69b** in 90/10 ratio (58 mg, 71%) was obtained as a white crystalline solid.

Major isomer 69a: [1]**H NMR** (400 MHz, $CDCl_3$): δ 7.66 (bs, 1H), 7.25-7.22 (m, 2H), 7.01-6.99 (m, 2H), 5.46-5.45 (m, 1H), 3.85 (s, 3H), 2.74-2.70 (m, 1H), 2.23-2.10 (m, 4H), 1.96-1.91 (m, 1H), 1.75 (s, 3H). [13]**C NMR** (101 MHz, $CDCl_3$): δ 183.0, 176.4, 159.9, 134.1, 129.4 (2C), 125.3, 116.7, 114.4 (2C), 62.5, 55.6, 33.2, 29.3, 26.2, 23.2.

Minor isomer 69b: [1]**H NMR** (400 MHz, $CDCl_3$): δ 7.66 (bs, 1H), 7.25-7.22 (m, 2H), 7.01-6.99 (m, 2H), 5.57-5.56 (m, 1H), 3.85 (s, 3H), 2.74-2.70 (m, 1H), 2.23-2.10 (m, 4H), 1.96-1.91 (m, 1H), 1.75 (s, 3H).

HRMS (ESI+) m/z calcd. for ($C_{16}H_{19}N_2O_2S$, M+H): 303.1162, found: (M+H): 303.1158.

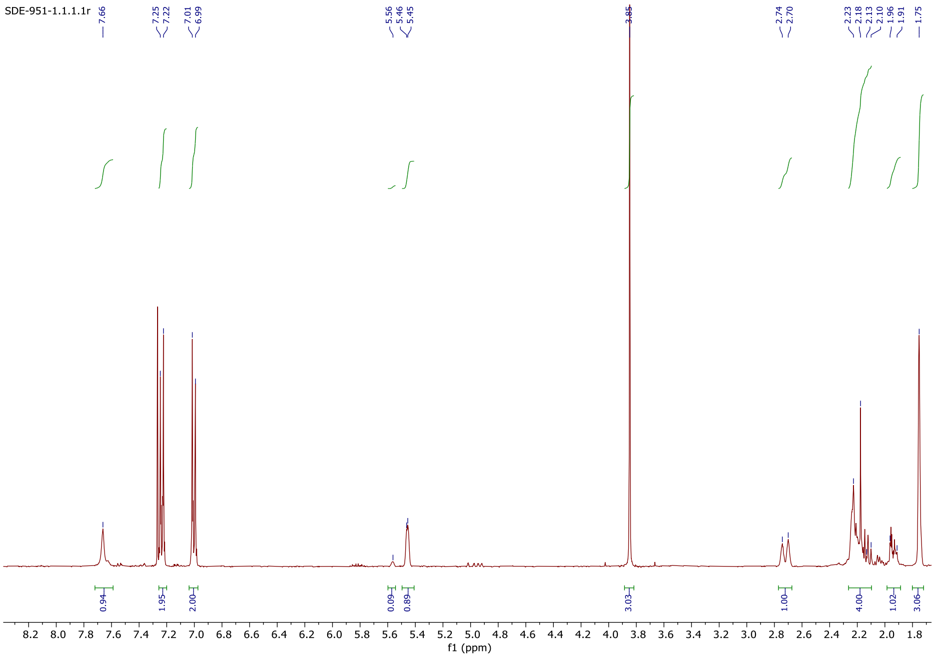

Figure S130. ^{1}H NMR spectra of the mixture of compounds **69a** and **69b**.

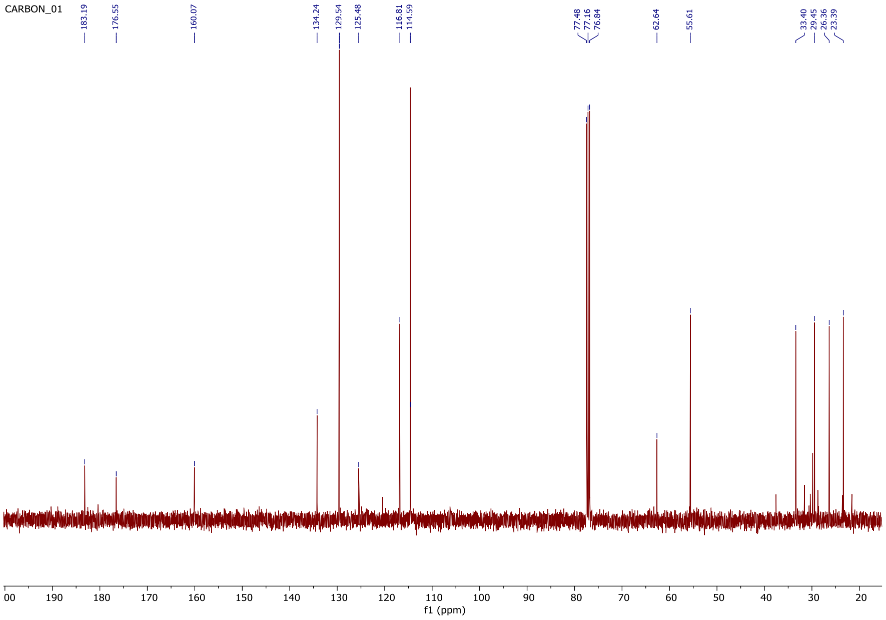

Figure S131. ^{13}C NMR spectra of the mixture of compounds **69a** and **69b**.

General procedure for alkylation of imidazolones 28a, 34a, 45a, 45b, 53.

To a solution of imidazolone (0.27 mmol) and K_2CO_3 (1.5-3 eq.) in acetonitrile (30 ml) alkylation agent (MeI or $PhCH_2Cl$, 1-3 eq.) was added and the mixture was refluxed for 8 h (for hydantoin derivatives) or stirred overnight (for thiohydantoin derivatives). The solvent was removed in vacuo, the residue was dissolved in chloroform (20 mL) and then the insoluble solid was filtered off. After the solvent was removed in vacuo, the residue was purified by column chromatography on silica gel.

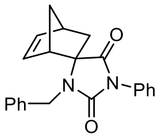

(1S,2S*,4S*)-3'-Benzyl-1'-phenylspiro[bicyclo[2.2.1]heptane-2,4'-imidazolidin]-5-ene-2',5'-dione* (**33a**) (isolated using chloroform as eluent). From methylideneimidazolone **28a** (69 mg, 0.27 mmol), K_2CO_3 (112 mg, 0.81 mmol) and $PhCH_2Cl$ (103 mg, 0.81 mmol) compound **33a** (56 mg, 60%) was obtained as a light yellow oil.

^{1}H NMR (400 MHz, $CDCl_3$): δ 7.55-7.17 (m, 10H), 6.51-6.47 (m, 1H), 5.86-5.79 (m, 1H), 4.67 (d, J = 16.3 Hz, 1H), 4.43 (d, J = 16.3 Hz, 1H), 3.11 (s, 1H), 3.00 (s, 1H), 2.61 (d, J = 9.0 Hz, 1H), 2.30 (dd, J_1 = 3.7 Hz, J_2 = 12.8 Hz, 1H), 1.62-1.42 (m, 2H). **^{13}C NMR** (101 MHz, $CDCl_3$): δ 175.8, 155.5, 140.3, 137.2, 133.6, 131.7, 128.5 (2C), 128.3 (2C), 127.5, 126.7, 126.2 (2C), 125.7 (2C), 69.1, 53.7, 47.8, 44.7, 41.6, 35.0. **HRMS** (ESI+) m/z calcd. for ($C_{22}H_{21}N_2O_2$, M+H): 345.1598, found: (M+H): 345.1600.

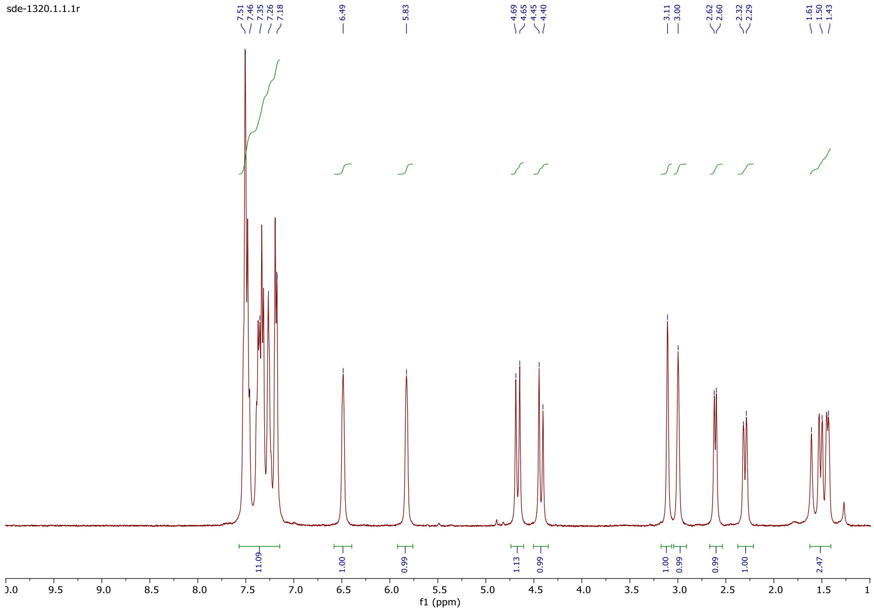

Figure S132. ^{1}H NMR spectra of compound **33a.**

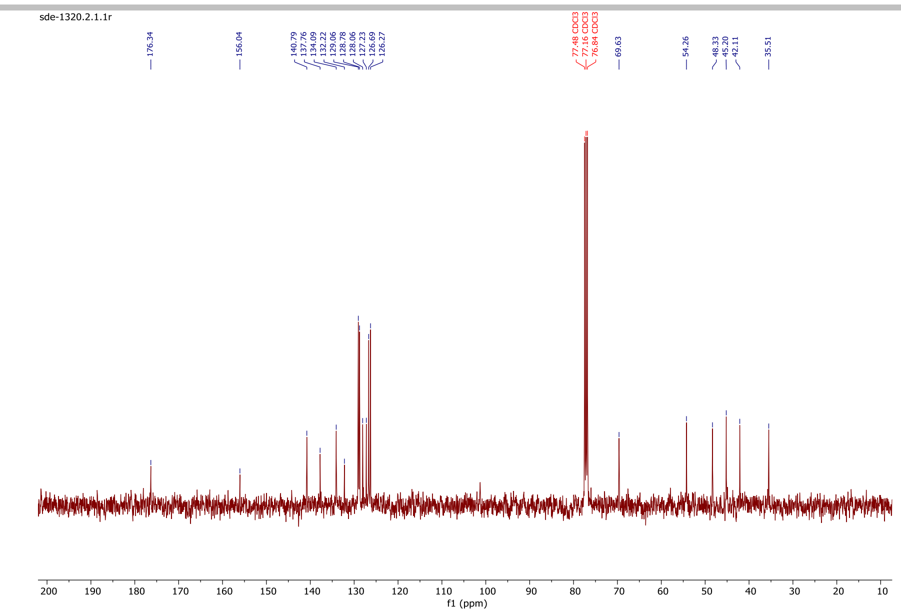

Figure S133. ^{13}C NMR spectra of compound **33a.**

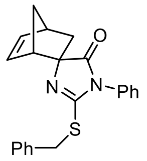

(1S,2S*,4S*)-2′-(Benzylthio)-1′-phenylspiro[bicyclo[2.2.1]heptane-2,4′-imidazol]-5-en-5′(1′H)-one* (**71**) (isolated using chloroform as eluent). From thiohydantoin **34a** (73 mg, 0.27 mmol), K_2CO_3 (57 mg, 0.41 mmol) and $PhCH_2Cl$ (34 mg, 0.27 mmol) compound **71** (95 mg, 98%) was obtained as a light yellow amorphous solid.

^{1}H NMR (400 MHz, $CDCl_3$): δ 7.49-7.21 (m, 10H), 6.54 (dd, J_1 = 3.1 Hz, J_2 = 5.6 Hz, 1H), 6.27 (dd, J_1 = 3.0 Hz, J_2 = 5.6 Hz, 1H), 4.36 (d, J = 13.0 Hz, 1H), 4.24 (d, J = 13.0 Hz, 1H), 3.11 (s, 1H), 2.97 (s, 1H), 2.37 (d, J = 8.5 Hz, 1H), 2.23 (dd, J_1 = 3.5 Hz, J_2 = 11.5 Hz, 1H), 1.57-1.45 (m, 2H). **^{13}C NMR** (101 MHz, $CDCl_3$): δ 184.0, 158.3, 138.4, 136.1, 135.0, 132.3, 129.0 (2C), 128.9 (2C), 128.4, 128.1 (2C), 127.2, 126.9 (2C), 77.3, 53.3, 46.1, 42.4, 39.1, 34.2. **HRMS** (ESI+) m/z calcd. for ($C_{22}H_{21}N_2OS$, M+H): 361.1369, found: (M+H): 361.1371.

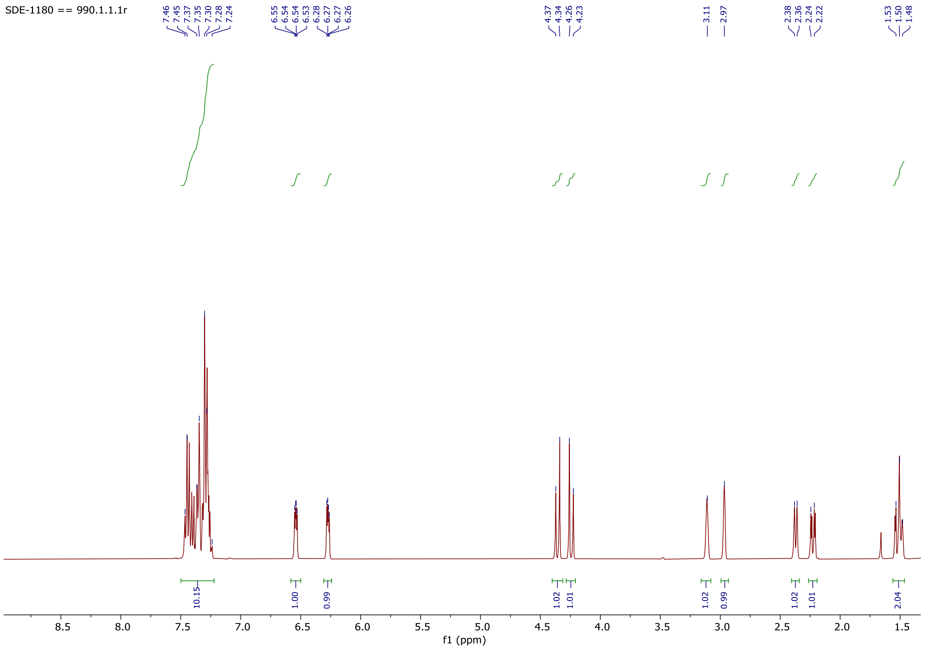

Figure S134. ^{1}H NMR spectra of compound **71.**

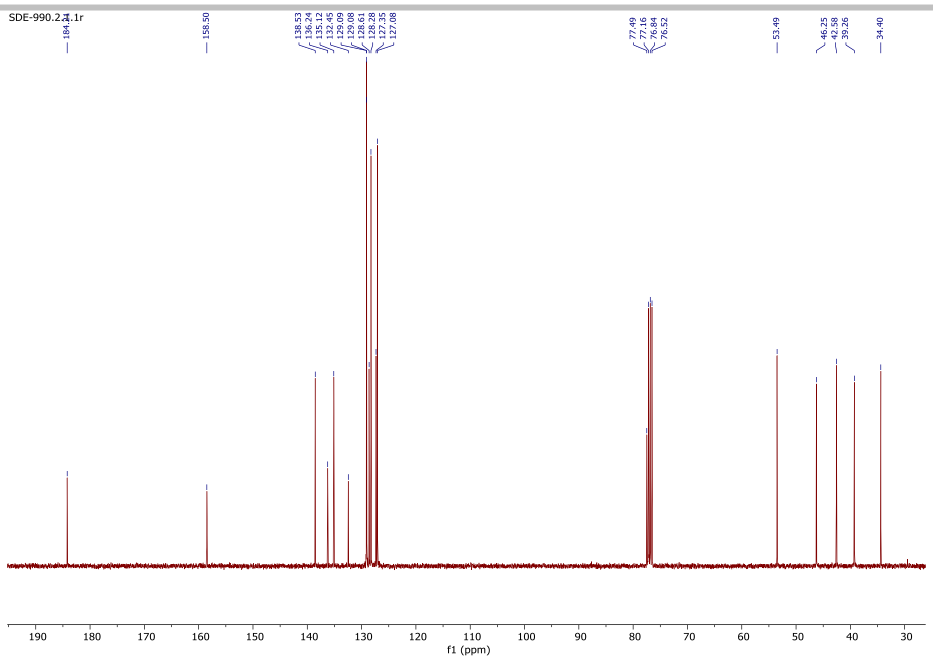

Figure S135. ^{13}C NMR spectra of compound **71.**

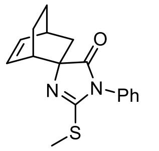

(1S,2S*,4S*)-2′-(Methylthio)-1′-phenylspiro[bicyclo[2.2.2]octane-2,4′-imidazol]-5-en-5′(1′H)-one* (**72**) (isolated using chloroform as eluent). From thiohydantoin **45a** (77 mg, 0.27 mmol), K_2CO_3 (112 mg, 0.81 mmol) and MeI (58 mg, 0.41 mmol) compound **72** (72 mg, 90%) was obtained as a light yellow amorphous solid.

^{1}H NMR (400 MHz, $CDCl_3$): δ 7.49-7.40 (m, 3H), 7.30-7.26 (m, 2H), 6.51-6.48 (m, 1H), 6.45-6.39 (m, 1H), 2.81-2.78 (m, 1H), 2.48-2.45 (m, 1H), 2.46 (s, 3H), 2.40-2.33 (m, 1H), 1.96 (dd, J_1 = 2.3 Hz, J_2 = 13.0 Hz, 1H), 1.76-1.69 (m, 1H), 1.62 (dt, J_1 = 3.2 Hz, J_2 = 13.0 Hz, 1H), 1.35-1.27 (m, 1H), 1.11-1.02 (m, 1H). **^{13}C NMR** (101 MHz, $CDCl_3$): δ 182.8, 158.7, 133.3, 133.2, 132.3, 128.9 (2C), 128.4, 127.0 (2C), 74.3, 38.3, 37.5, 29.7, 23.2, 18.2, 12.5. **HRMS** (ESI+) m/z calcd. for ($C_{17}H_{19}N_2OS$, M+H): 299.1213, found: (M+H): 299.1215.

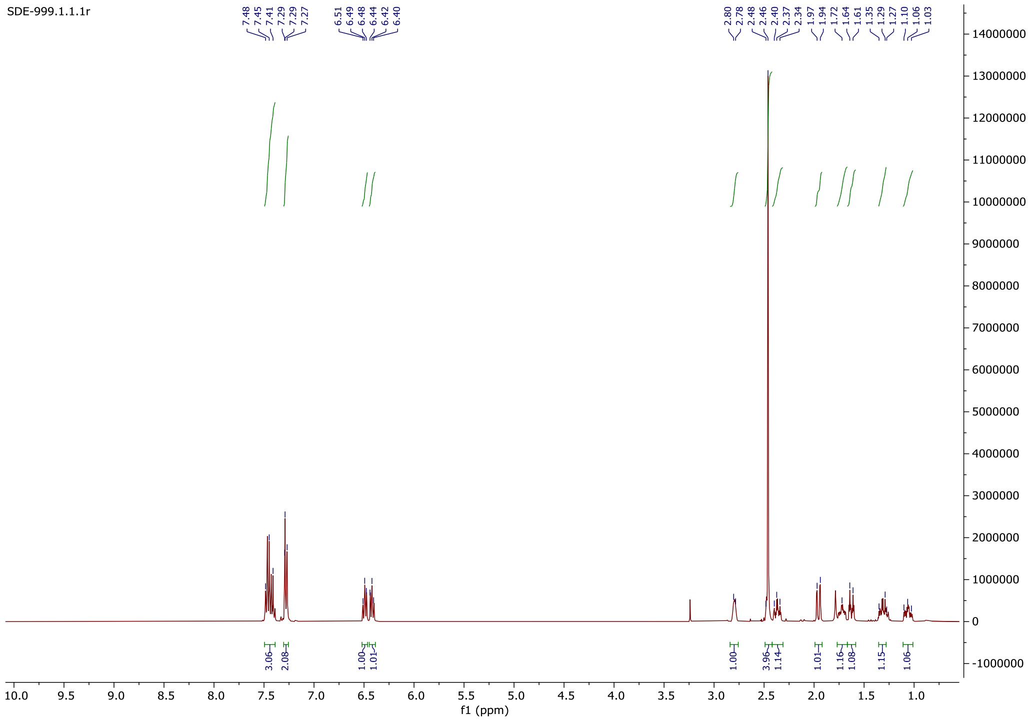

Figure S136. ^{1}H NMR spectra of compound **72.**

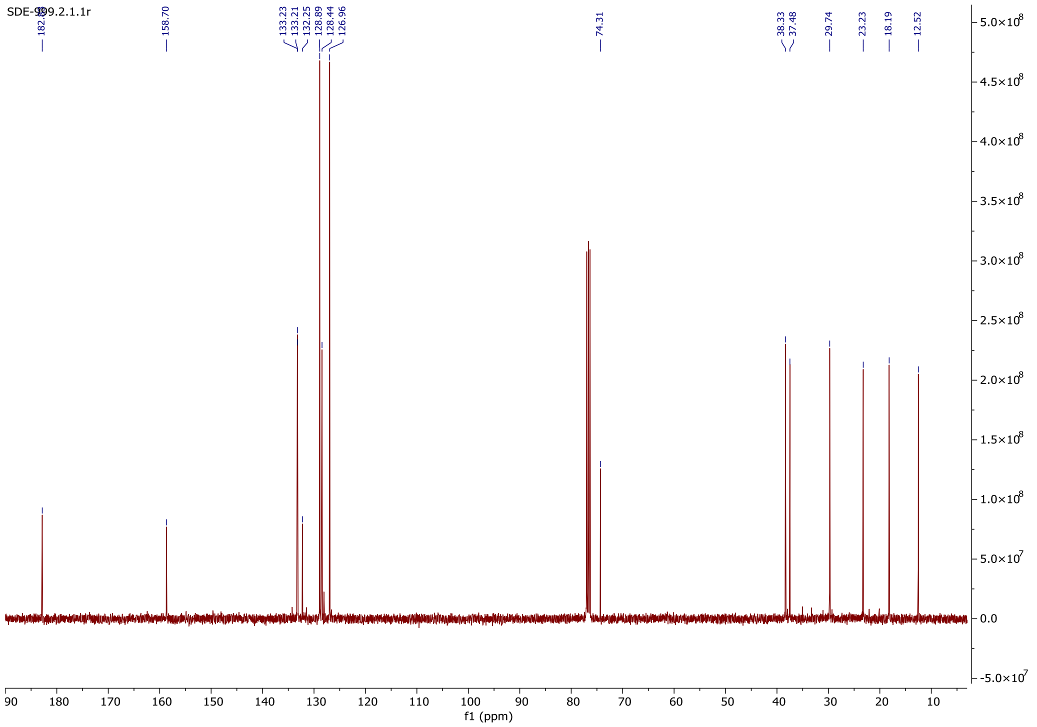

Figure S137. ^{13}C NMR spectra of compound **72.**

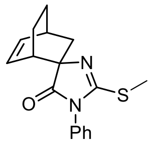

(1S,2R*,4S*)-2′-(Methylthio)-1′-phenylspiro[bicyclo[2.2.2]octane-2,4′-imidazol]-5-en-5′(1′H)-one* (**73**) (isolated using chloroform as eluent). From thiohydantoin **45b (**77 mg, 0.27 mmol), K_2CO_3 (112 mg, 0.81 mmol) and MeI (58 mg, 0.41 mmol) compound **73** (74 mg, 92%) was obtained as a light yellow amorphous solid.

^{1}H NMR (400 MHz, $CDCl_3$): δ 7.47-7.36 (m, 3H), 7.28-7.24 (m, 2H), 6.51-6.46 (m, 1H), 6.38-6.33 (m, 1H), 2.81-2.79 (m, 1H), 2.53 (s, 3H), 2.45-2.41 (m, 1H), 2.37-2.32 (m, 1H), 1.85-1.77 (m, 2H), 1.40-1.34 (m, 1H), 1.29-1.26 (m, 1H), 1.19-1.11 (m, 1H). **^{13}C NMR** (101 MHz, $CDCl_3$): δ 183.1, 158.7, 134.8, 132.3, 131.3, 128.8 (2C), 128.3, 126.8 (2C), 72.9, 38.6, 38.2, 29.7, 22.8, 21.7, 12.5. **HRMS** (ESI+) m/z calcd. for ($C_{17}H_{19}N_2OS$, M+H): 299.1213, found: (M+H): 299.1211.

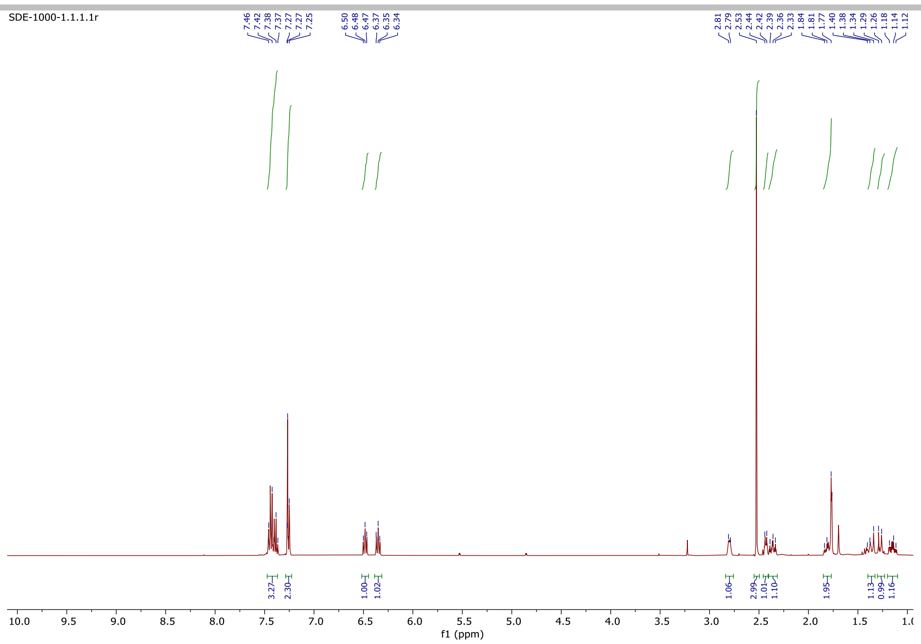

Figure S138. ^{1}H NMR spectra of compound **73.**

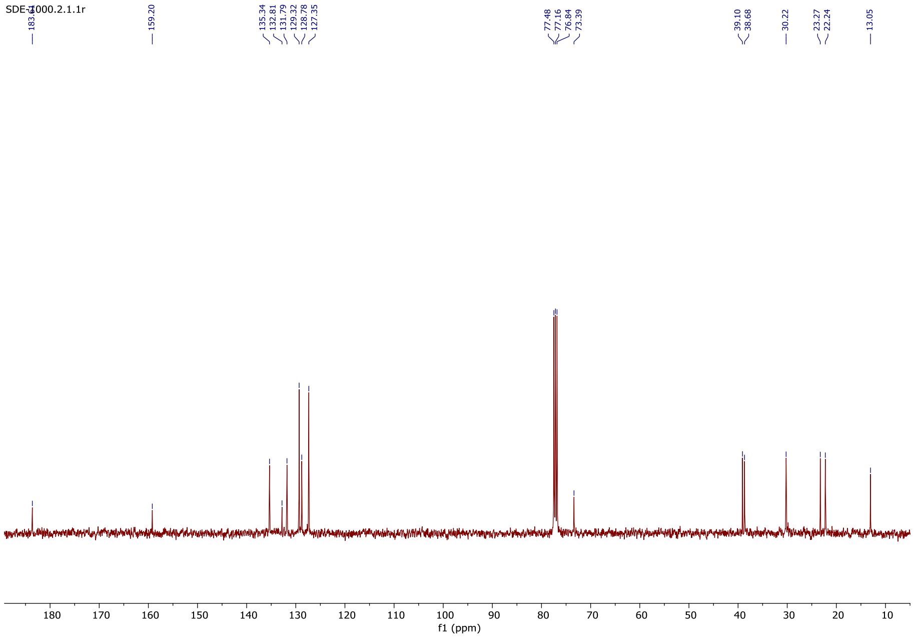

Figure S139. ^{13}C NMR spectra of compound **73.**

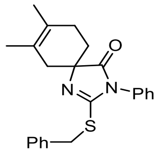

2-(Benzylthio)-7,8-dimethyl-3-phenyl-1,3-diazaspiro[4.5]deca-1,7-dien-4-one (**74**) (isolated using chloroform as eluent). From thiohydantoin **53** (77 mg, 0.27 mmol), K_2CO_3 (57 mg, 0.41 mmol) and $PhCH_2Cl$ (34 mg, 0.27 mmol) compound **74** (95 mg, 96%) was obtained as a light yellow amorphous solid.

^{1}H NMR (400 MHz, $CDCl_3$): δ 7.49-7.18 (m, 10H), 4.41-4.32 (m, 2H), 5.45-5.43 (m, 1H), 2.57 (d, J = 17.5 Hz, 1H), 2.53-2.42 (m, 1H), 2.11 (d, J = 17.5 Hz, 1H), 2.03-1.90 (m, 2H), 1.78 (s, 3H), 1.73 (s, 3H), 1.73-1.66 (m, 1H). **^{13}C NMR** (101 MHz, $CDCl_3$): δ 183.2, 158.4, 136.2, 132.1, 129.0 (2C), 128.9 (2C), 128.5, 128.1 (2C), 127.1, 126.9 (2C), 124.9, 121.2, 70.9, 38.5, 34.4, 30.2, 28.0, 18.7, 18.5. **HRMS** (ESI+) m/z calcd. for ($C_{23}H_{25}N_2OS$, M+H): 377.1682, found: (M+H): 377.1664.

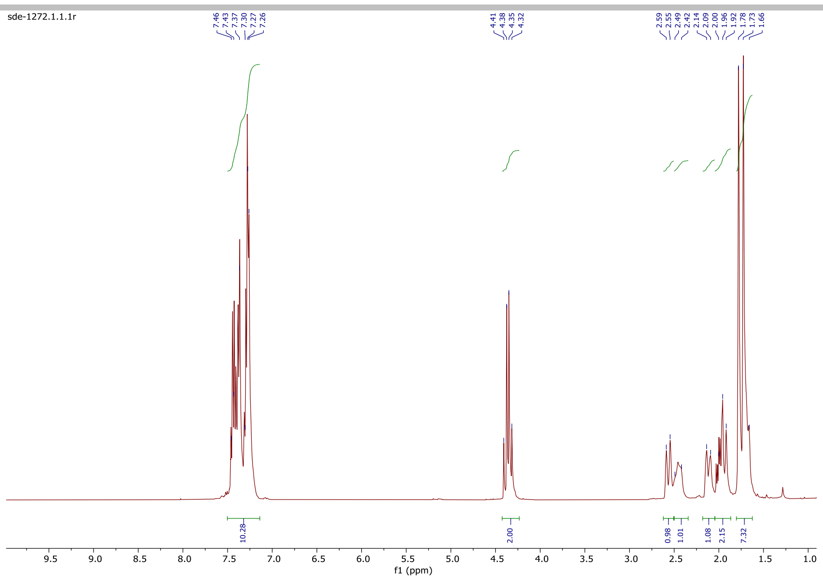

Figure S140. ^{1}H NMR spectra of compound **74.**

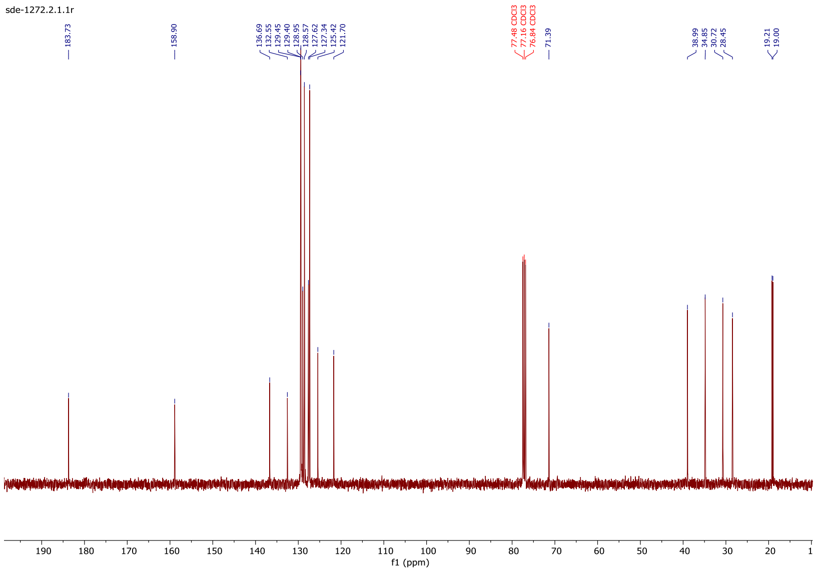

Figure S141. ^{13}C NMR spectra of compound **74.**

General procedure of acylation of hydantoins 28a, 44a.

To a solution of hydantoin (0.27 mmol) and Boc_2O (70 mg, 0.32 mmol) in chloroform (30 ml) triethylamine (32 mg, 0.32 mmol) and DMAP (33 mg, 0.27 mmol) were added and the mixture was refluxed for 10 h. After the solvent was removed in vacuo, the residue was purified by column chromatography on silica gel.

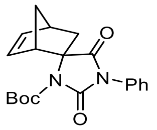

Tert-butyl (1S,2S*,4S*)-2′,5′-dioxo-1′-phenylspiro[bicyclo[2.2.1]heptane-2,4′-imidazolidin]-5-ene-3′-carboxylate* (**32a**) (isolated using chloroform as eluent). From hydantoin **28a** (69 mg, 0.27 mmol), Boc_2O (70 mg, 0.32 mmol), triethylamine (32 mg, 0.32 mmol) and DMAP (33 mg, 0.27 mmol) compound **32a** (86 mg, 86%) was obtained as a white crystalline solid.

^{1}H NMR (400 MHz, $CDCl_3$): δ 7.52-7.43 (m, 2H), 7.43-7.34 (m, 3H), 6.50 (dd, J_1 = 3.1 Hz, J_2 = 5.6 Hz, 1H), 6.10 (dd, J_1 = 3.0 Hz, J_2 = 5.6 Hz, 1H), 3.21-3.17 (m, 1H), 3.13 (s, 1H), 2.72 (dd, J_1 = 3.6 Hz, J_2 = 11.9 Hz, 1H), 2.39-2.30 (m, 2H), 1.53 (s, 9H), 1.53-1.40 (m, 1H). **^{13}C NMR** (101 MHz, $CDCl_3$): δ 174.4, 152.1, 149.6, 144.2, 131.3, 130.8, 129.1 (2C), 128.5, 126.5 (2C), 84.4, 71.3, 55.6, 47.3, 43.3, 35.1, 27.7 (3C). **HRMS** (ESI+) m/z calcd. for ($C_{20}H_{23}N_2O_4$, M+H): 355.1652, found: (M+H): 355.1648.

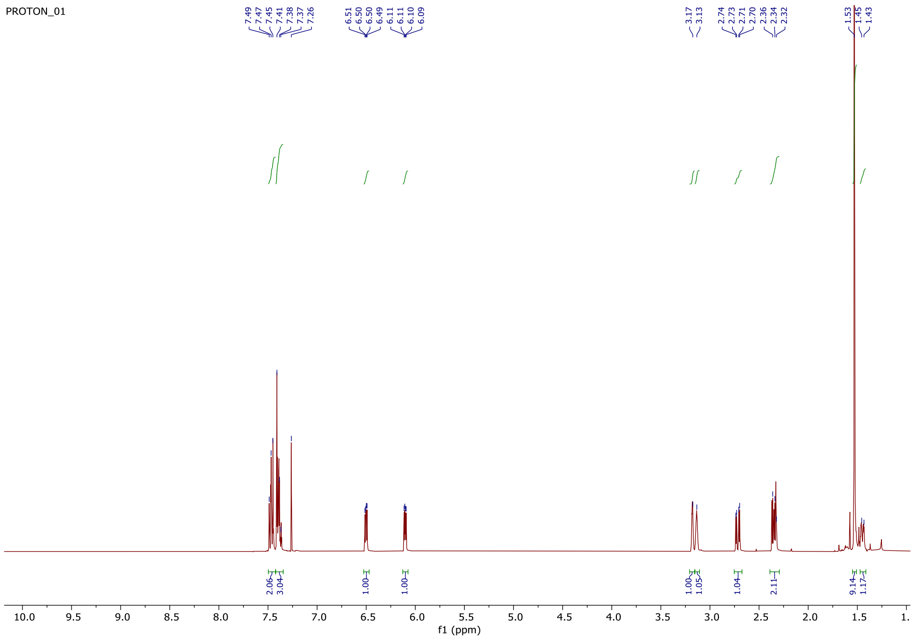

Figure S142. ^{1}H NMR spectra of compound **32a.**

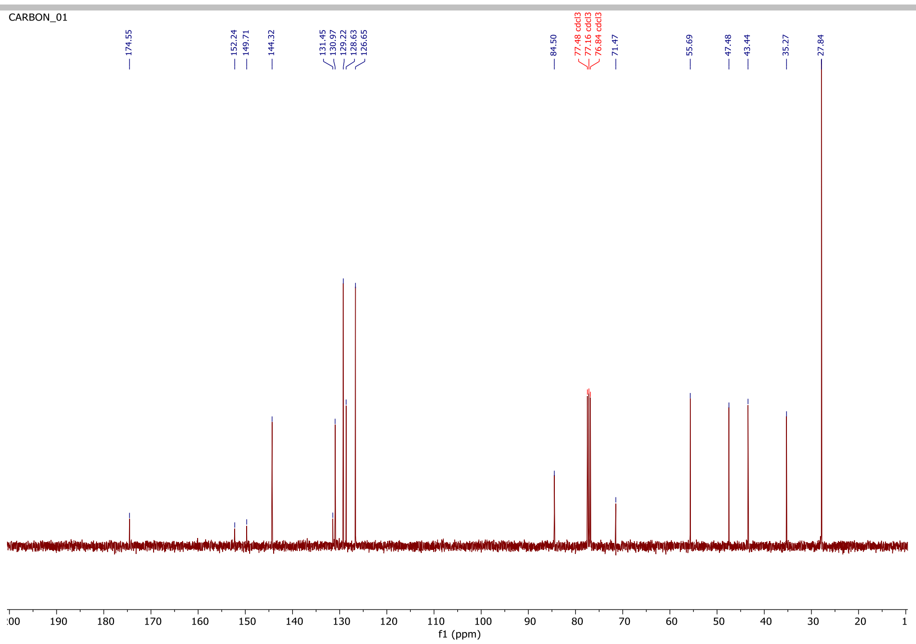

Figure S143. ^{13}C NMR spectra of compound **32a.**

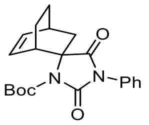

Tert-butyl (1S,2S*,4S*)-2′,5′-dioxo-1′-phenylspiro[bicyclo[2.2.2]octane-2,4′-imidazolidin]-5-ene-3′-carboxylate* (**70**) (isolated using chloroform as eluent). From hydantoin **44a** (72 mg, 0.27 mmol), Boc_2O (70 mg, 0.32 mmol), triethylamine (32 mg, 0.32 mmol) and DMAP (33 mg, 0.27 mmol) compound **70** (82 mg, 83%) was obtained as a white crystalline solid.

^{1}H NMR (400 MHz, $CDCl_3$): δ 7.49-7.43 (m, 2H), 7.41-7.35 (m, 3H), 6.50-6.43 (m, 1H), 6.29-6.23 (m, 1H), 2.90-2.85 (m, 1H), 2.84-2.79 (m, 1H), 2.70 (dt, J_1 = 3.3 Hz, J_2 = 13.6 Hz, 1H), 2.24-2.16 (m, 1H), 2.14 (dd, J_1 = 2.0 Hz, J_2 = 13.7 Hz, 1H), 1.77-1.69 (m, 1H), 1.54 (s, 9H), 1.31-1.27 (m, 1H), 1.13-1.05 (m, 1H). **^{13}C NMR** (101 MHz, $CDCl_3$): δ 173.1, 151.8, 149.6, 137.7, 131.3, 129.3, 129.0 (2C), 128.4, 126.5 (2C), 84.3, 68.9, 40.5, 34.8, 30.5, 27.8 (3C), 23.3, 20.7. **HRMS** (ESI+) m/z calcd. for ($C_{21}H_{25}N_2O_4$, M+H): 391.1628, found: (M+H): 391.1635.

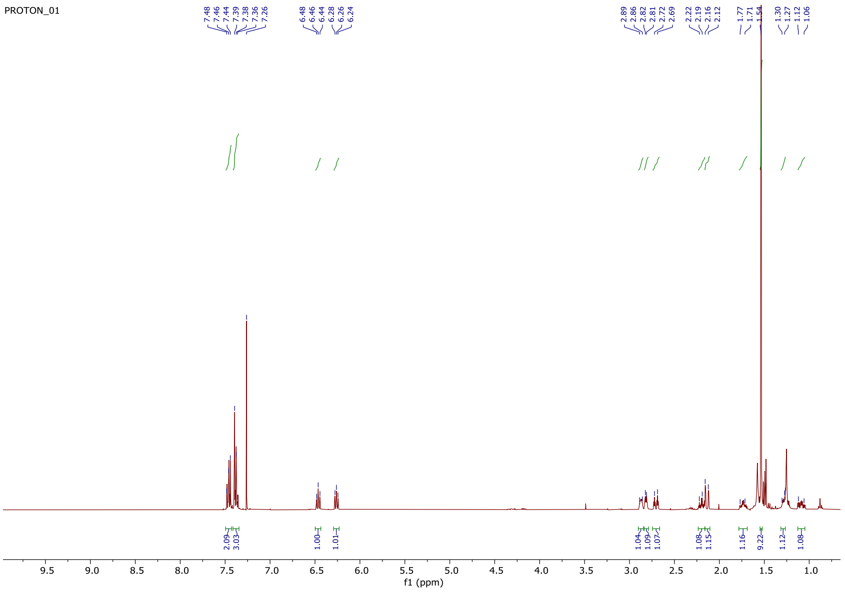

Figure S144. ^{1}H NMR spectra of compound **70.**

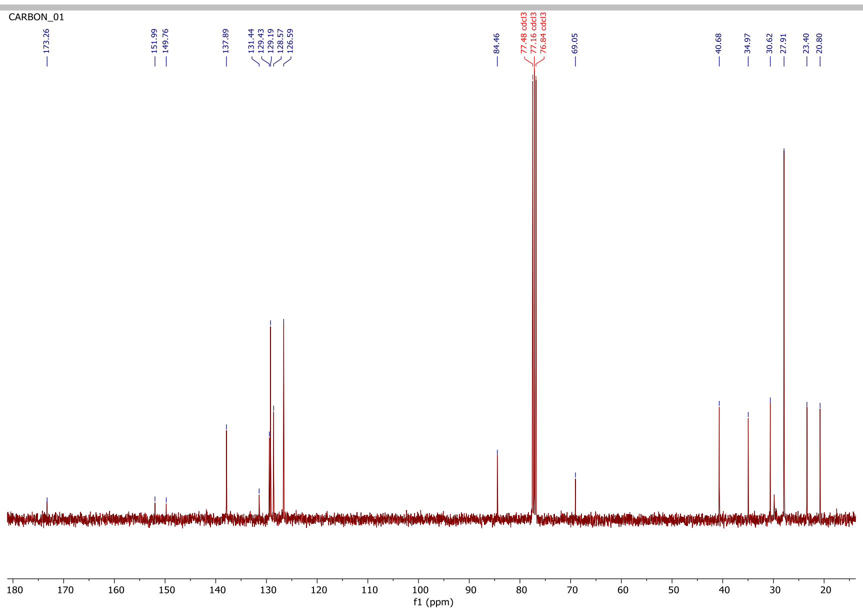

Figure S145. ^{13}C NMR spectra of compound **70.**

General procedure of desulfurization of thiohydantoins 34a, 45a with hydrogen peroxide.

To a solution of thiohydantoin (0.19 mmol) in methanol (30 ml) 30% aqueous H_2O_2 (100 ◎l) was added and the mixture was stirred for 2 days. After the solvent was removed in vacuo, the residue was purified by column chromatography on silica gel.

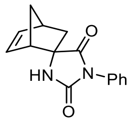

(1S,2S*,4S*)-1′-Phenylspiro[bicyclo[2.2.1]heptane-2,4′-imidazolidin]-5-ene-2′,5′-dione* (**28a**) (isolated using methanol/chloroform (1:200) as eluent). From thiohydantoin **34a** (51 mg, 0.19 mmol) and 30% aqueous H_2O_2 (100 ◎l) compound **28a** (29 mg, 60%) was obtained as a white crystalline solid.

The spectral data are consistent with the literature data [1].

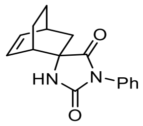

(1S,2S*,4S*)-1′-Phenylspiro[bicyclo[2.2.2]octane-2,4′-imidazolidin]-5-ene-2′,5′-dione* (**44a**) (isolated using methanol/chloroform (1:200) as eluent). From thiohydantoin **45a** (54 mg, 0.19 mmol) and 30% aqueous H_2O_2 (100 ◎l) compound **44a** (33 mg, 65%) was obtained as a white crystalline solid.

Spectral data are described above in Figures S55-S58.

General procedure of desulfurization of thiohydantoins 34a, 36a, 37a, 53, 57, 62 with 1,3-dipolar cycloaddition of nitrile oxides using diffusion mixing technique.

A mixture of thiohydantoin (0.19 mmol) and 4-chloro-N-hydroxybenzimidoyl chloride (0.19 mmol) in 3 ml of chloroform was placed to a 15 ml vial **1** (diameter 1.3 cm) and closed with a glass stopper with holes. The vial **1** was then placed in closed 50 ml vial **2** (diameter 3.5 cm) containing triethylamine (35.85 mmol, 5 ml) and the reaction mixture was stirred at room temperature for two days (TLC or NMR control). When the reaction was completed, the mixture from the inner vial was diluted with 10 ml of chloroform, transferred to a separating funnel and washed with 2% aqueous HCl (2 x 10 ml). The organic phase was dried over anhydrous Na_2SO_4, then mixture was boiling for 2 h. After that the solvent was removed under reduced pressure, and the residue was purified by column chromatography on silica gel.

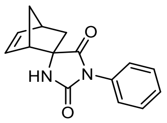

(1S,2S*,4S*)-1′-Phenylspiro[bicyclo[2.2.1]heptane-2,4′-imidazolidin]-5-ene-2′,5′-dione* (**28a**) (isolated using methanol/chloroform (1:200) as eluent). From thiohydantoin **34a** (51 mg, 0.19 mmol) and 4-chloro-N-hydroxybenzimidoyl chloride (36 mg, 0.19 mmol) compound **28a** (43 mg, 90%) was obtained as a white crystalline solid.
The spectral data are consistent with the literature data [1].

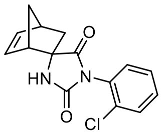

(1S,2S*,4S*)-1′-(2-Chlorophenyl)spiro[bicyclo[2.2.1]heptane-2,4′-imidazolidin]-5-ene-2′,5′-dione* (**75**) (isolated using methanol/chloroform (1:200) as eluent). From thiohydantoin **36a** (58 mg, 0.19 mmol) and 4-chloro-N-hydroxybenzimidoyl chloride (36 mg, 0.19 mmol) compound **75** (52 mg, 95%) was obtained as a white crystalline solid.
^{1}H NMR (400 MHz, $CDCl_3$): δ 7.57-7.50 (m, 1H), 7.41-7.31 (m, 3H), 6.55-6.51 (m, 1H), 6.25-6.19 (m, 1H), 5.94 (bs, 1H), 3.18-3.08 (m, 1H), 3.05 (m, 1H), 2.52-2.43 (m, 1H), 2.30-2.23 (m, 1H), 1.58-1.51 (m, 1H), 1.44-1.36 (m, 1H). **^{13}C NMR** (101 MHz, $CDCl_3$): (two sets of signals for C(Ar)-N-rotamers) δ 175.7, 175.5, 154.4, 154.4, 142.2, 142.2, 132.8, 132.7, 132.7, 132.6, 130.3, 130.2, 130.2, 130.1, 130.0, 129.9, 129.2, 129.1, 127.3, 127.3, 67.1, 67.0, 52.2, 51.6, 47.3, 47.2, 42.2, 42.1, 41.2, 40.4. **HRMS** (ESI+) m/z calcd. for (C15H14ClN2O2, M+H): 289.0738, found: (M+H): 289.0736.

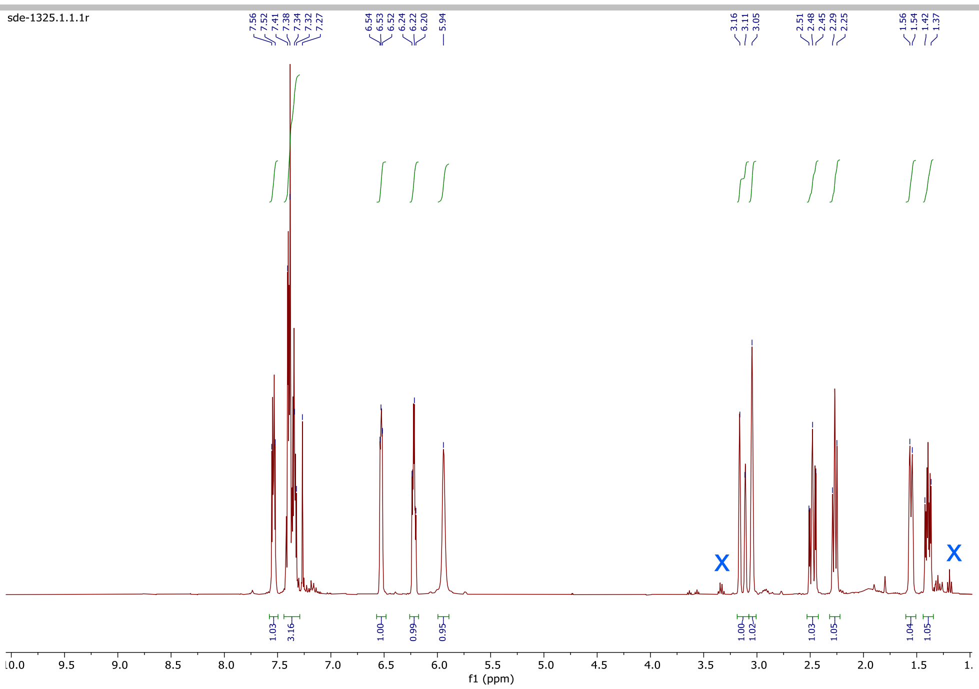

Figure S146. ^{1}H NMR spectra of compound **75** (*the sign "x" marks the residual signals of Et_3N*).

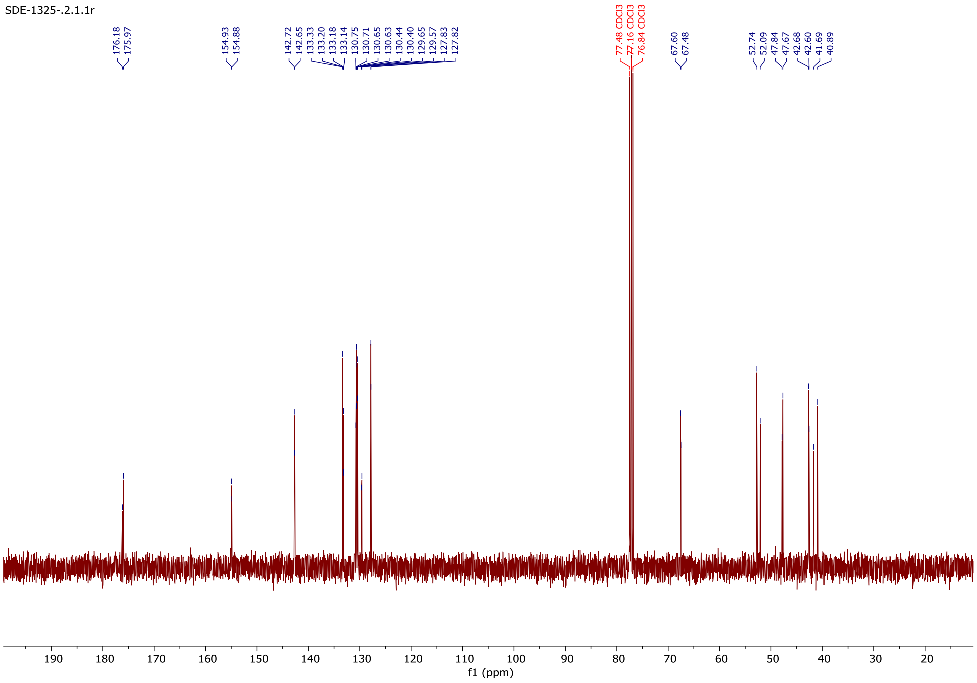

Figure S147. ^{13}C NMR spectra of compound **75**.

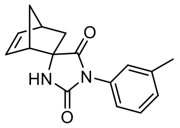

(1S,2S*,4S*)-1′-(m-Tolyl)spiro[bicyclo[2.2.1]heptane-2,4′-imidazolidin]-5-ene-2′,5′-dione* (**76**) (isolated using methanol/chloroform (1:200) as eluent). From thiohydantoin **37a** (54 mg, 0.19 mmol) and 4-chloro-N-hydroxybenzimidoyl chloride (36 mg, 0.19 mmol) compound **76** (51 mg, 98%) was obtained as a white crystalline solid.

^{1}H NMR (400 MHz, $CDCl_3$): δ 7.35 (t, J = 7.7 Hz, 1H), 7.26-7.15 (m, 3H), 6.56 (dd, J_1 = 3.1 Hz, J_2 = 5.7 Hz, 1H), 6.23 (dd, J_1 = 3.0 Hz, J_2 = 5.8 Hz, 1H), 5.77 (bs, 1H), 3.12-3.04 (m, 2H), 2.46 (dd, J_1 = 3.5 Hz, J_2 = 12.3 Hz, 1H), 2.40 (s, 3H), 2.30 (d, J = 9.1 Hz, 1H), 1.58-1.54 (m, 1H), 1.38 (dd, J_1 = 3.6 Hz, J_2 = 12.3 Hz, 1H).

^{13}C NMR (101 MHz, $CDCl_3$): δ 176.1, 155.1, 142.2, 138.6, 132.8, 131.2, 128.6, 128.4, 126.5, 123.0, 66.3, 51.9, 47.3, 42.2, 41.0, 21.0. **HRMS** (ESI+) m/z calcd. for ($C_{16}H_{17}N_2O_2$, M+H): 269.1285, found: (M+H): 269.1286.

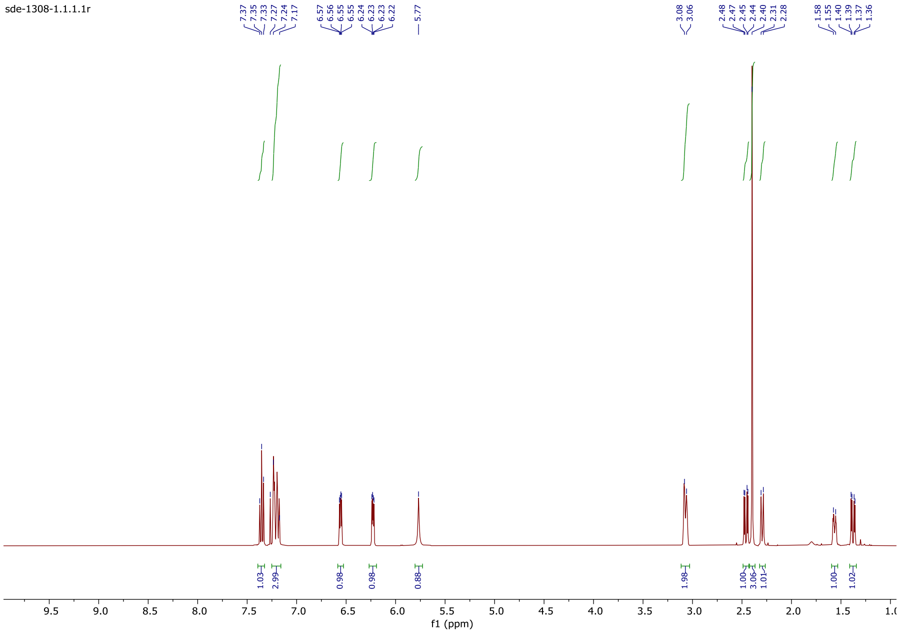

Figure S148. ^{1}H NMR spectra of compound **76**.

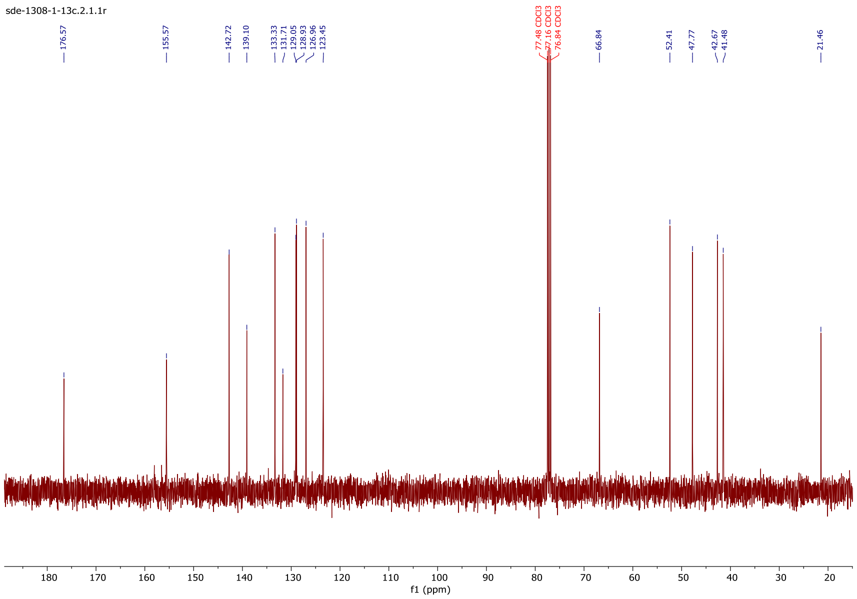

Figure S149. ^{13}C NMR spectra compound **76**.

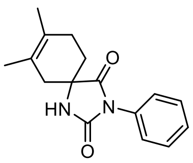

7,8-dimethyl-3-phenyl-1,3-diazaspiro[4.5]dec-7-ene-2,4-dione (**52**) (isolated using methanol/chloroform (1:200) as eluent). From thiohydantoin **53** (54 mg, 0.19 mmol) and 4-chloro-N-hydroxybenzimidoyl chloride (36 mg, 0.19 mmol) compound **52** (46 mg, 90%) was obtained as a white crystalline solid.
Spectral data are described above in Figures S91, S92.

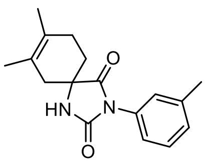

7,8-Dimethyl-3-(m-tolyl)-1,3-diazaspiro[4.5]dec-7-ene-2,4-dione (**77**) (isolated using methanol/chloroform (1:200) as eluent). From thiohydantoin **58** (57 mg, 0.19 mmol)

and 4-chloro-N-hydroxybenzimidoyl chloride (36 mg, 0.19 mmol) compound **77** (49 mg, 90%) was obtained as a white crystalline solid.
^{1}H NMR (400 MHz, $CDCl_3$): δ 7.36-7.15 (m, 4H), 6.63 (bs, 1H), 2.73 (d, J = 17.2 Hz, 1H), 2.38 (s, 3H), 2.20-2.16 (m, 2H), 2.10-1.96 (m, 2H), 1.83-1.77 (m, 1H), 1.65 (s, 6H). **^{13}C NMR** (101 MHz, $CDCl_3$): δ 175.4, 155.5, 138.5, 131.1, 128.5, 128.3, 126.3, 124.9, 122.8, 121.5, 60.3, 39.2, 29.5, 27.3, 20.9, 18.7, 18.2. **HRMS** (ESI+) m/z calcd. for ($C_{17}H_{21}N_2O_2$, M+H): 285.1598, found: (M+H): 285.1601.

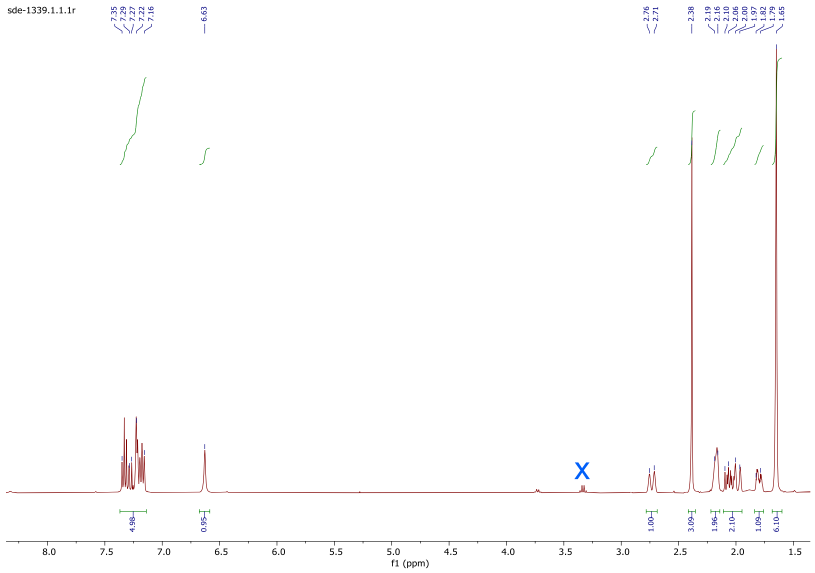

Figure S150. ^{1}H NMR spectra of compound **77** (*the sign "x" marks the residual signals of* Et_3N).

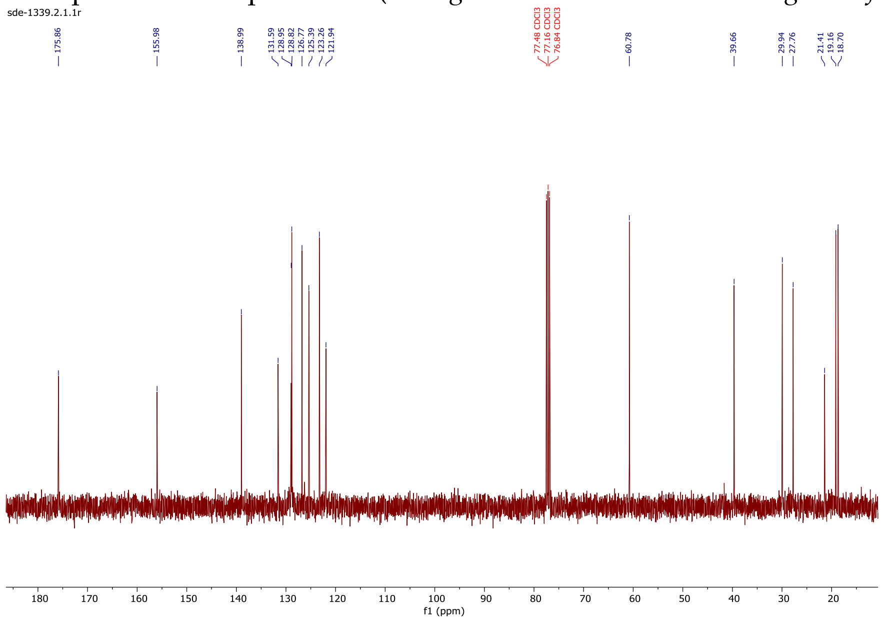

Figure S151. ^{13}C NMR spectra compound **77**.

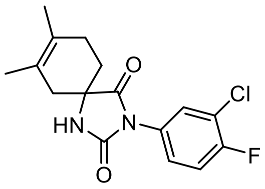

3-(3-chloro-4-fluorophenyl)-7,8-dimethyl-1,3-diazaspiro[4.5]dec-7-ene-2,4-dione (**78**) (isolated using methanol/chloroform (1:200) as eluent). From thiohydantoin **62** (61 mg, 0.19 mmol) and 4-chloro-N-hydroxybenzimidoyl chloride (36 mg, 0.19 mmol) compound **78** (47 mg, 81%) was obtained as a white crystalline solid.
^{1}H NMR (400 MHz, $CDCl_3$): δ 7.59-7.55 (m, 1H), 7.59-7.55 (m, 1H), 7.41-7.36 (m, 1H), 7.26-7.18 (m, 1H), 6.49 (bs, 1H), 2.73 (d, J = 16.9 Hz, 1H), 2.21-2.16 (m, 2H), 2.11-1.95 (m, 2H), 1.84-1.78 (m, 1H), 1.67 (s, 6H). **^{13}C NMR** (101 MHz, $CDCl_3$): δ 175.3 (s), 157.4 (d, J =

250.2 Hz), 155.2 (s), 128.9 (s), 128.3 (s), 125.8 (d, J = 7.7 Hz), 125.5 (s), 121.9 (s), 121.2 (s), 116.8 (d, J = 22.0 Hz), 60.9 (s), 39.7 (s), 30.0 (s), 27.7 (s), 19.2 (s), 18.8 (s). **^{19}F NMR** (376 MHz, $CDCl_3$): δ -115.44 (ddd, J_1 = 4.1 Hz, J_2 = 6.5 Hz, J_3 = 8.5 Hz). **HRMS** (ESI+) m/z calcd. for ($C_{16}H_{17}ClFN_2O_2$, M+H): 323.0963, found: (M+H): 323.0961.

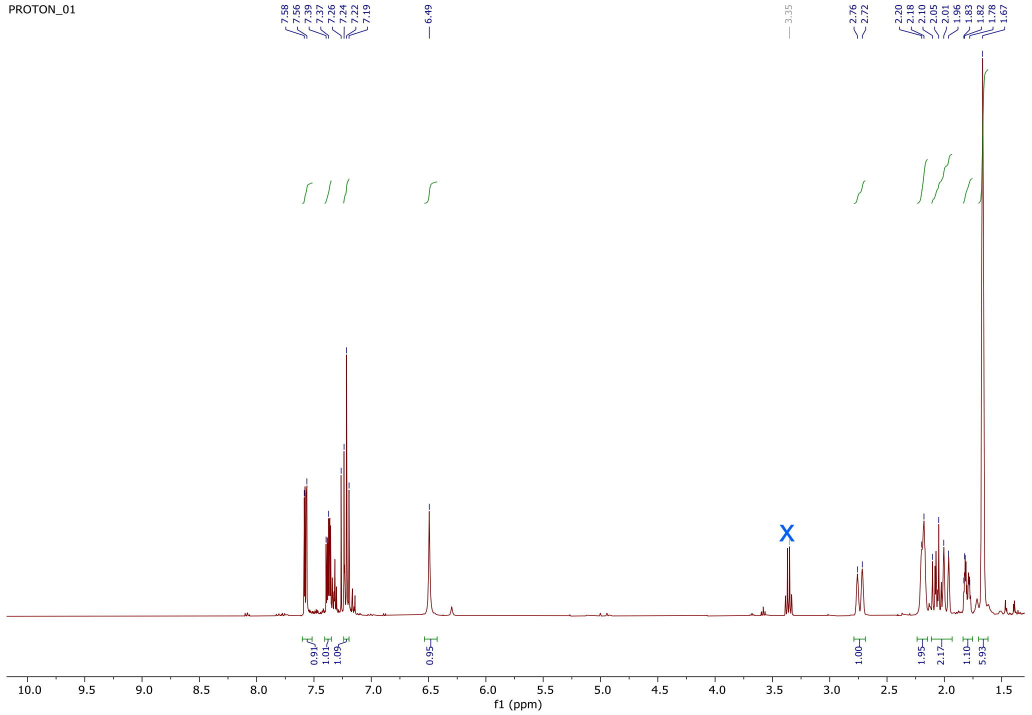

Figure S152. ^{1}H NMR spectra of compound **78** (*the sign "x" marks the residual signals of Et$_3$N*).

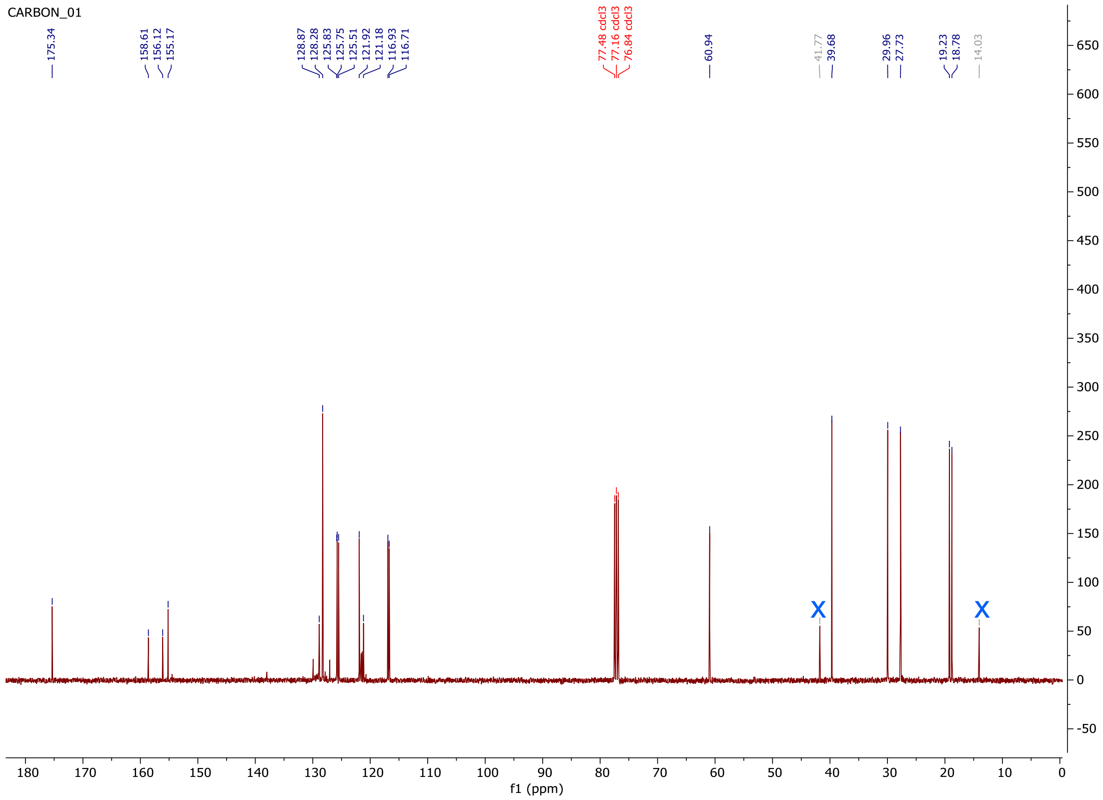

Figure S153. ^{13}C NMR spectra compound **78** (*the sign "x" marks the residual signals of Et$_3$N*).

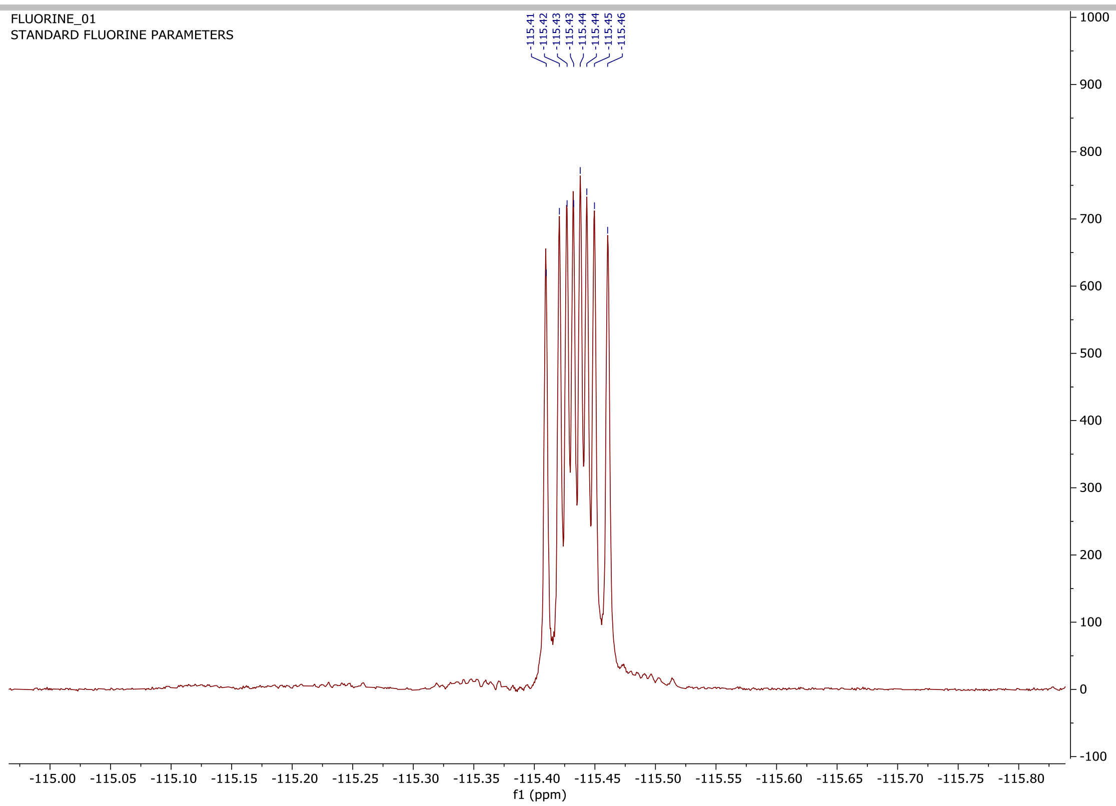

Figure S154. ^{19}F NMR spectra of compound **78**.

Cytotoxicity study: concentration-cytotoxicity dependencies (by MTT test)

28a

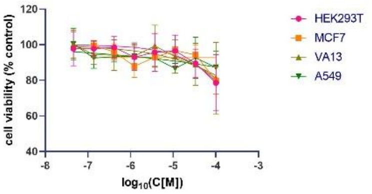

28b

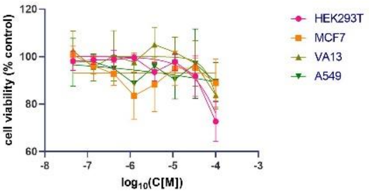

34a

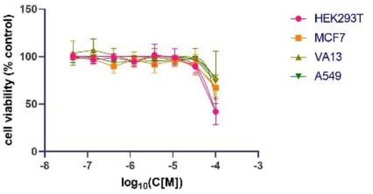

35b

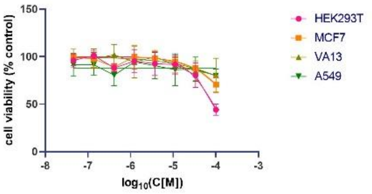

37a

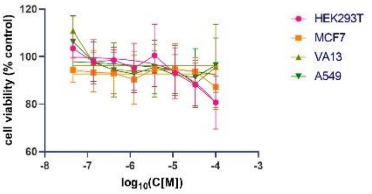

37b

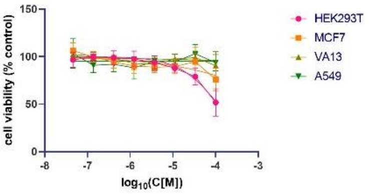

38a

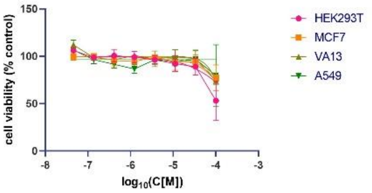

38b

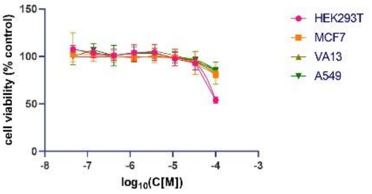

39b

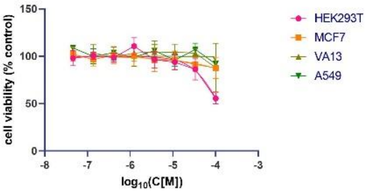

40a

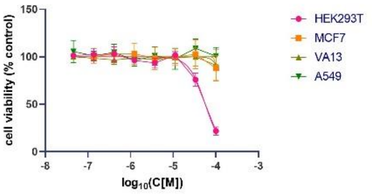

41a

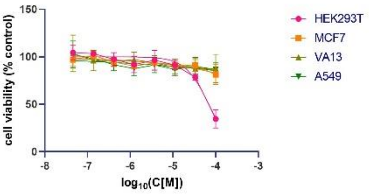

45a

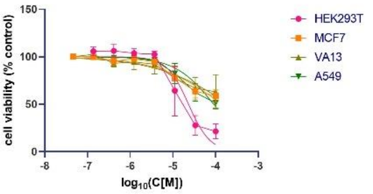

48a

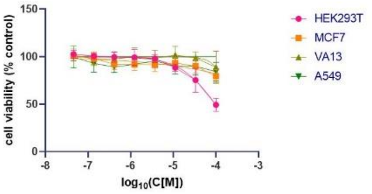

48b

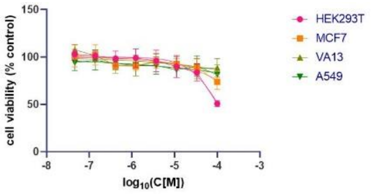

49a

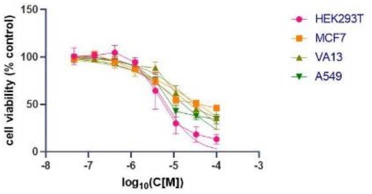

50a

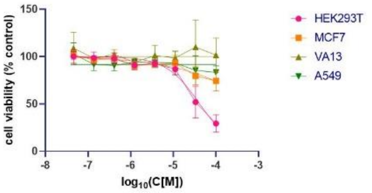

50b

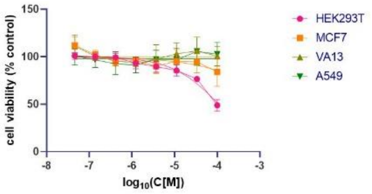

51a

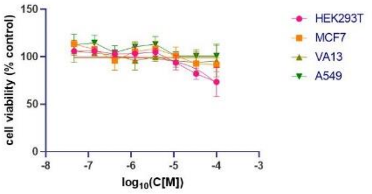

51b

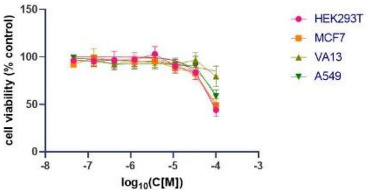

54

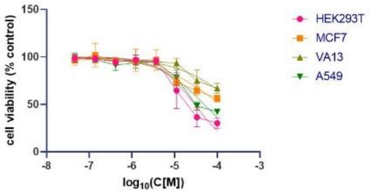

55

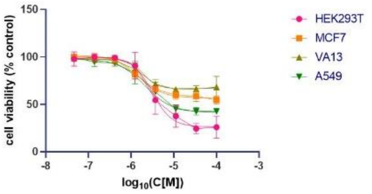

56

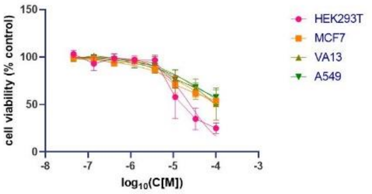

57

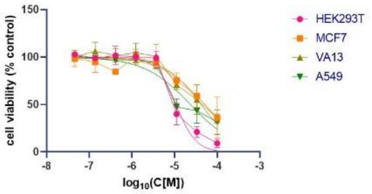

58

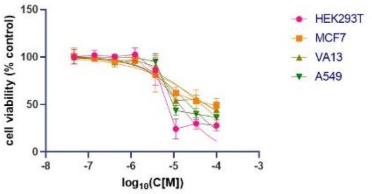

59

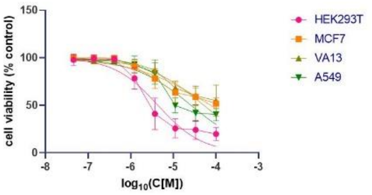

60

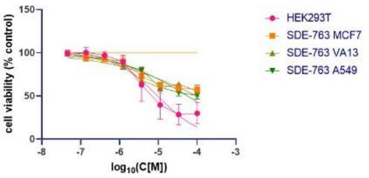

61

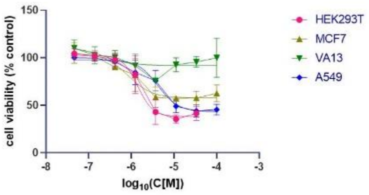

62

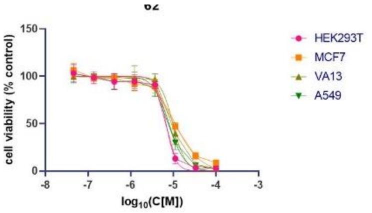

66a + 66b

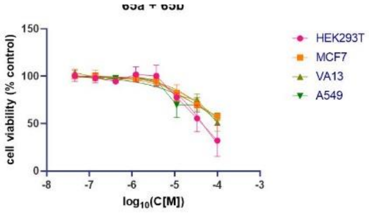

72

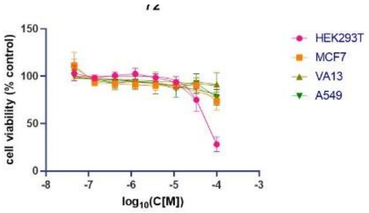

75

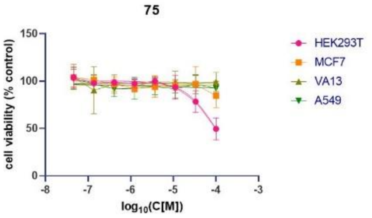

76

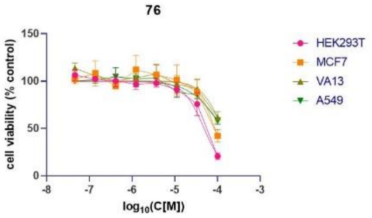

Figure S155. Concentration-response dependencies of compounds' cytotoxicity for HEK293T, MCF7, VA13 and A549 cell lines.

Table S1. Cytotoxicity (IC_{50} values) for the compounds **55** and **61** for HEK293T, MCF7, VA13 and A549 cell lines **(**μM)

Compound	HEK293T	MCF7	VA13	A549
**55**	3,1±0,4	1,5±0,2	1,9±0,3	2,1±0,2
**61**	1,4±0,2	0,9±0,2	0,4±0,3	4,2±0,5

X-Ray data

Table S2. Crystal data and structure refinement for compound **51a**.

Empirical formula	C18 H20 N2 O S	
Formula weight	312.42	
Temperature	296(2) K	
Wavelength	1.54186 Å	
Crystal system	Monoclinic	
Space group	P 21/n	
Unit cell dimensions	a = 12.7761(8) Å	a= 90°.
	b = 21.1240(10) Å	b= 110.120(8)°.
	c = 12.9820(8) Å	g = 90°.
Volume	3289.8(4) Å^{3}	
Z	8	
Density (calculated)	1.262 Mg/m^{3}	
Absorption coefficient	1.763 mm^{-1}	
F(000)	1328	
Theta range for data collection	4.186 to 66.755°.	
Index ranges	-15<=h<=13, -25<=k<=24, -9<=l<=15	
Reflections collected	20955	
Independent reflections	5707	
Completeness to theta = 66.755°	97.7 %	
Refinement method	Full-matrix least-squares on F^{2}	
Data / restraints / parameters	5707 / 0 / 410	
Goodness-of-fit on F^{2}	0.722	
Final R indices [I>2sigma(I)]	R1 = 0.0854, wR2 = 0.1890	
R indices (all data)	R1 = 0.2665, wR2 = 0.2423	
Extinction coefficient	0.00107(19)	
Largest diff. peak and hole	0.476 and -0.506 e. Å^{-3}	

Table S3. Hydrogen bonds for compound **51a** [Å and °].

D-H...A	d(D-H)	d(H...A)	d(D...A)	<(DHA)
N(2A)-H(2A)...S(1B)	0.97(7)	2.41(7)	3.330(7)	160(5)
N(2B)-H(2B)...S(1A)	1.03(7)	2.39(7)	3.388(7)	165(5)

Symmetry transformations used to generate equivalent atoms:

References

[1]. Shybanov, D.E.; Kukushkin, M.E.; Tafeenko, V.A.; Zyk, N. V.; Grishin, Y.K.; Roznyatovsky, V.A.; Beloglazkina, E.K. Different Addition Modes of Cyclopentadiene and Furan at Methylidene(Thio)Hydantoins. *Mendeleev Commun.* **2021**, *31*, 246–247, doi:10.1016/j.mencom.2021.03.034.
